# Supplementary material for: Asymmetric α-benzylation of cyclic ketones enabled by concurrent chemical aldol condensation and biocatalytic reduction
Source: Nat Commun. 2024 Jan 2;15:71. doi: 10.1038/s41467-023-44452-z (PMC10761851; doi:10.1038/s41467-023-44452-z)
Supplement: Supplementary file 1 — Supplementary Information [file 41467_2023_44452_MOESM1_ESM.pdf]

Supplementary Information for:

Asymmetric α-Benzylation of Cyclic Ketones Enabled by Concurrent Chemical Aldol Condensation and Biocatalytic Reduction

Yunting Liu[a], Teng Ma[a], Zhongxu Guo[a], Liya Zhou[a], Guanhua Liu[a], Ying He[a], Li Ma[a], Jing Gao[a], Jing Bai[b], Frank Hollmann*,[c], and Yanjun Jiang*,[a]

[a] School of Chemical Engineering and Technology, Hebei University of Technology, Tianjin 300130, China.

[b] College of Food Science and Biology, Hebei University of Science & Technology, Shijiazhuang, 050018, China.

[c] Department of Biotechnology, Delft University of Technology, 2629 HZ Delft, The Netherlands.

*E-mail: f.hollmann@tudelft.nl; yanjunjiang@hebut.edu.cn

Supplementary Methods

1. General Information

Cyclopentanone, cyclohexanone and cycloheptanone were purchased from Aladdin Reagents Co. Ltd (Shanghai, China). 2-methylbenzaldehyde, 3-methylbenzaldehyde, 4-methylbenzaldehyde, 2-methoxybenzaldehyde, 3-methoxybenzaldehyde, and 4-methoxybenzaldehyde were obtained from J&K Chemical Co. Ltd. (Beijing, China). Unless otherwise stated, other reagents are purchased from Aladdin Reagents Co. Ltd (Shanghai, China).

For thin-layer chromatography (TLC) analysis throughout this work, Merck precoated TLC plates (silica gel 60 GF254, 0.25 mm) were used. The products were purified by preparative column chromatography on silica gel E. Merck 9385. NMR spectra were recorded on a Bruker AV 400 spectrometer at 400 MHz (^{1}H NMR and ^{13}C NMR). Chemical shifts were reported in ppm relative to internal TMS for 1H NMR data, respectively. Data are presented in the following space: chemical shift, multiplicity, coupling constant in hertz (Hz), and signal area integration in natural numbers. The conversion and enantiomeric excess were determined by gas chromatography (GC) analysis on a Shimadzu-2010 GC with a flame ionization detector (FID) using nitrogen as carrier gas. Agilent J&W CP-Chiralsil-DEX CB capillary column (25 m × 0.25 mm × 0.25 μm) was used for determining the conversions and ee values, respectively. In the cases of using high-performance liquid chromatography (HPLC) (Shimadzu) for determining the ee values, Chiralpak AD-3 column or Chiralpak OD column were used.

2. Experimental Section

2.1 Mutagenesis

The site-directed mutagenesis was conducted using recombinant plasmids containing YqjM genes (Supplementary Table 1) as templates with the following program: 98 °C for 30 s, (98 °C for 10 s, 58 °C for 30 s, 68 °C for 1 min) with 30 cycles, 68 °C for 5 min. 50 μL PCR reaction mixture contains 1.5 μL each of forward and reverse primer (2 μM), 5 μL dNTP (2 mM), 5 μL 10 * KOD buffer, 2 μL $MgSO_4$ (25 mM), 1 μL KOD DNA polymerase, and adequate plasmid DNA. The PCR products were digested by *Dpn* I and transformed into *E. coli.* BL21 (DE3) cells. The sequences were verified by DNA sequencing, and the reduction activity of YqjM variants was detected via spectrophotometric assay. The primers used in this work were shown in Supplementary Table 2.

2.2 Construction of plasmid

Plasmid pET28b(+) (Novagen, Germany) was used as the expression vector for the wild-type YqjM and the mutants constructed in this study. The gene YqjM encoding for the wild-type YqjM was codon optimized for expression in *E. coli* and synthesized from GENWIZ (Suzhou, China). The gene YqjM was cloned into the plasmid pET28b(+) between Nde I and Hind III (N-terminal 6×His tag) and confirmed by sequencing. The obtaining plasmid pET28b(+)-YqjM was used as the template for mutagenesis. The YqjM mutants were generated using the KOD mutagenesis kit (TOYOBO, China) with the corresponding mutagenic primers (Supplementary Table 2).

2.3 Protein expression and purification

For YqjM expression, the plasmid pET28b-YqjM was transformed to *E. coli* BL21 (DE3). Single colonies were inoculated in 10 mL LB culture containing 50 μg mL^{-1} Kanamycin at 37 °C. 500 μL of the overnight culture were added to 50 mL TB culture containing 50 μg mL^{-1} Kanamycin and grown to an OD_{600} of 0.6–0.8 at 37 °C. The culture was induced by isopropyl-β-D-1-thiogalactopyranoside (IPTG) with a final concentration of 0.1 mM and incubated at 30 °C on a rotary shaker for 12 h. The cell pellets, collected by centrifugation at 5180 g for 10 min, were lysed using cell crusher. The YqjM enzyme was purified using Ni-NTA affinity chromatography. The Ni-NTA column (GE, 5 mL) was pre-equilibrated with buffer A containing 50 mM PBS, 500 mM NaCl, and 10 mM imidazole at pH 7.5. Then, the supernatant containing the soluble YqjM was loaded into the pre-equilibrated Ni-NTA column, and subsequently gradient eluted by PBS buffer (500 mM NaCl, pH 7.5) containing 100, 300, and 500 mM imidazole for 5-10 column volumes, respectively. The fractions containing YqjM was verified by SDS-PAGE analysis and concentrated by ultrafiltration. The enzyme buffer was exchanged to 50 mM PBS buffer containing 150 mM NaCl, 1 mM DTT and the purified enzyme was stored at 4 °C. The protein concentration of purified enzyme was determined by Nanodrop 2000c spectrophotometer (Thermo Scientific) at 595 nm using the extinction coefficients predicted from the Expasy ProtParam Tool. SDS-page documenting the purity of the YqjMs is provided as a Source Data file.

2.4 Enzyme activity assay

The activity assay of YqjM was performed by measuring the decrease of NADPH absorbance at 340 nm. Briefly, the assays were performed in 1 mL of 100 mM phosphate buffer solution (PBS, pH 7.5) containing 2 mM NADPH at 30 °C. The substrate (*E*)-2-benzylidenecyclopentan-1-one (**1a**, 1 mM) and 10 μM YqjM were added. The change in absorption values at 340 nm was monitored using a UV spectrophotometer, and calculated according to Formula 1. Unless otherwise stated, each of the pilot trials in this experiment was repeated 3 times, and the mean was taken to eradicate any discrepancies.

$$\text{Enzyme activity (U/g)} = \frac{Ew * V * 1000}{6220 * 1} \quad (1)$$

Ew is the change in the absorption value at 340 nm for 1 min; *V* the total volume of the reaction system, in L; 6220: molar extinction coefficient, in L $mol^{-1}cm^{-1}$; and 1: optical path distance of the cuvette.

The formula for calculating specific enzyme activity is as follows:

$$\text{Specific activity (U/g)} = \frac{U}{m} \quad (2)$$

U is the amount of enzyme that catalyzed the conversion of 1 μmol substrate during 1 min; *m* is the amount (g) of enzyme added.

2.5 DFT calculations

The DFT region was extracted from the wt-YqjM structure obtained from PDB (PDB code: 1Z41 [https://doi.org/10.2210/pdb1z41/pdb]) and the YqjM mutant structure generated by SWISS-MODEL, consisting of the substrate, the flavin mononucleotide (FMN) truncated at the C1 position, and the side-chain atoms of Y169 or Y69. Additionally, the water molecule located between the substrate and Y69 was included in the DFT region when considering water as a proton donor. All DFT calculations were carried out using the Gaussian 09 program.[1] The geometry optimizations of intermediates and transition states were performed using the B3LYP+D3/6-31G(d) level.[2] The SMD[3] solvation model using the experimentally used solvent, water, was applied to estimate the effect of enzyme-surrounding by calculating single-point implicit solvation using a dielectric constant of $\varepsilon = 4$. Vibrational frequency calculations were performed for all stationary points to confirm if each optimized structure is a local minimum or a transition state structure. All optimized transition state structures have only one imaginary (negative) frequency, and all minima have no imaginary frequencies. To get more accurate energies, the single-point energy was calculated using the larger basis set 6-311++G(2d,p),[4] denoted as the B3LYP+D3/6-311++G(2d,p) (SMD, solvent = water)//B3LYP+D3/6-31G(d) (SMD, solvent = water) level.[5] It should be emphasized that although the Tyr residues were not constrained, the DFT optimization led to only negligible change in the mutual poses of the Tyr residues compared to those assumed in the enzyme, reflecting the reasonable real pocket environment.

2.6 Procedures for synthesizing exocyclic substrates

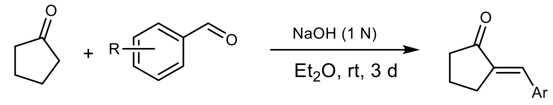

The synthesis of α-arylidene cyclopentanones[6]: The corresponding aryl aldehydes (5 mmol) and cyclopentanone (6 mmol) were taken in a round bottom flask and dissolved in 10 mL of diethyl ether. Then aqueous sodium hydroxide solution (1M, 5 mL) was added to it and stirred at the room temperature for 3 days. After complete consumption of benzaldehyde, water was added to the reaction mixture and organic part was extracted with diethyl ether. The organic layer was dried over anhydrous sodium sulfate and concentrated under reduced pressure. Finally, the product was purified by flash silica gel column chromatography (eluent-hexane:ethyl acetate= 95:5).

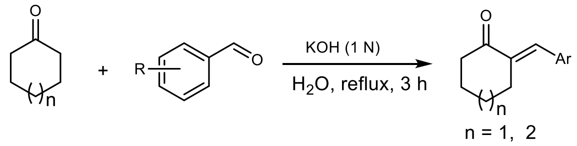

The synthesis of α-arylidene cyclohexanones[7]: The corresponding aryl aldehydes (5 mmol) and cyclohexanone (6 mmol) were taken in a 50 mL round bottom flask and aqueous potassium hydroxide solution (1M, 5 mL) was added to it. Then the reaction mixture was refluxed for 3h. After full consumption of benzaldehyde, it was extracted with diethyl ether and concentrated under vacuum. Finally, the desired product was isolated via silica gel flash column chromatography using hexane-ethyl acetate mixture (95:5) as eluent.

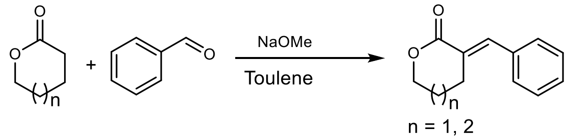

The synthesis of α-arylidene lactones[8]: To a solution of benzaldehyde (18.7 mmol) and lactone (37.4 mmol) in toluene (40 mL) was added sodium methoxide (NaOMe, 1.32 g, 24.5 mmol) at -10 °C. After being stirred at the same temperature for 5 min, the mixture was stirred at room temperature for 2 h. Then the mixture was diluted with AcOEt (30 mL) and added dropwise with concentrated H_2SO_4 (1.35 mL) and H_2O (20 mL) in an ice bath. The organic layer was washed subsequently with saturated $NaHCO_3$

solution, H_2O and brine, and then dried over Na_2SO_4. The solvent was removed under reduced pressure. The residue was recrystallized from *i*-Pr_2O or purified by silica gel chromatography to afford pure product.

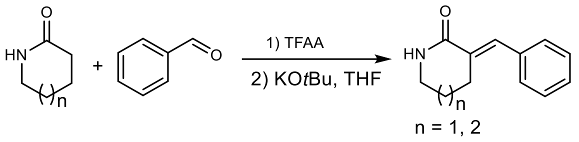

The synthesis of α-arylidene lactams[9]: The lactam (20 mmol) was dissolved in toluene (15 mL) and the solution was cooled to 5 °C, followed by the slow addition of trifluoroacetic anhydride (TFAA) (3 mL, 1.1 eq). The resulting solution was allowed to warm to room temperature, and was stirred further for 1 h at this temperature. Toluene and the residual TFAA was removed under vacuum. The resulting brownish oily product was mixed with the corresponding aldehyde (0.9 equiv), and the mixture was slowly charged to a mixture of potassium *t*-butoxide (KO*t*Bu, 1.2 equiv) in THF (25 mL) under an ice/water bath. The mixture was heated to 55°C and stirred therein for 1 h. The reaction mixture was concentrated to a slurry under vacuum, then water (100 mL) was added. The mixture was filtered, and the solid was washed with water and dried in vacuum oven overnight. The crude product was purified by recrystallization from ethyl acetate/petroleum ether or hexane.

2.7 Procedures for synthesizing racemic α-benzyl cyclic carbonyl compounds

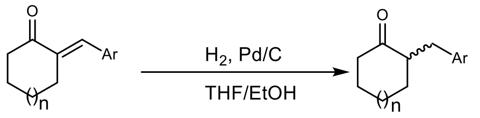

The synthesis of α-arylidene cyclic ketones[10]: 25 mg of Pd/C (containing 0.0235 mmol of Pd) and a magnetic bar were added into a reaction tube charged with N_2. A solution of 1.0 mmol of substrates in 10 mL of anhydrous THF/EtOH (volume ratio = 4:1) was injected into the reaction tube, which was then equipped with a balloon charged with H_2. The mixture was magnetically stirred at room temperature (25 °C) for 6 h. The solvent was evaporated under vacuum and the residue was separated by flash column chromatogram (eluent: petroleum ether-EtOAc 20:1) to give the corresponding products.

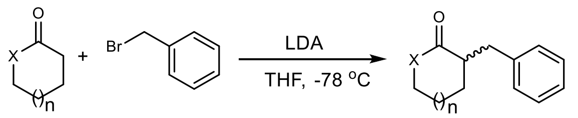

The synthesis of α-benzyl lactones and lactams.[11] Diisopropylamine (3.7 mL, 26.0 mmol, 1.0 equiv) in dry THF (130 mL) was cooled to -78 °C under Ar. *n*Butyl lithium (LDA, 2.5 M in hexane, 10.4 mL, 26.0 mmol, 1.0 equiv) was added dropwise and the mixture was stirred for 15 min. Lactones (26.0 mmol, 1.0 equiv) or lactams (13.0 mmol, 0.5 equiv) was dissolved in THF (5 mL), added dropwise, and the mixture was stirred for 30 min. 4-Bromobenzyl bromide (7.1 g, 28.6 mmol, 1.1 equiv) was dissolved in THF (5 mL) and was added slowly, the mixture was stirred at -78 to 23 °C for 16 h. Saturated ammonium chloride (aqueous, 600 mL) was added and the mixture was extracted with diethyl ether (2 x 60 mL). The combined organic layers were washed with brine (2 x 60 mL), dried over anhydrous Na_2SO_4, and concentrated in vacuo. The crude oil was purified by flash chromatography on SiO_2 to obtain pure products.

2.8 Procedure for preparing organobismuth catalyst

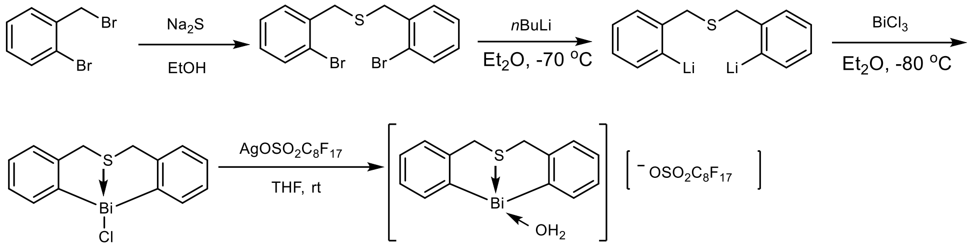

$S(CH_2\text{-}2\text{-}C_6H_4Br)_2$[12]**:** 2-Bromobenzyl bromide (23.3 g,0.092 mol) was dissolved in degassed absolute ethanol (300 mL), and sodium sulfide nonahydrate (11.2 g, 0.047 mol) was added, along with sodium hydroxide (four pellets). The reaction mixture was refluxed for 16 h, giving a cream-colored precipitate and an orange solution. The solvent was removed in vacuo, and the resulting solids were treated with a mixture of diethyl ether (200 mL) and degassed water (100 mL). The ether layer was removed and the remaining solution washed with more diethyl ether (60 mL). The combined organic extracts were dried over $MgSO_4$ before the majority of the solvent was removed in vacuo, precipitating a pale yellow crystalline solid.

$S(CH_2C_6H_4)_2BiCl$[13]: Bis(2-bromobenzyl) sulfide (14.88 g, 40.0 mmol) was dissolved in dried ethyl ether (150 mL), and then 32.6 mL (81.6 mmol, 2.5M in hexane) of *n*-butyllithium was added dropwise at -70 °C into the solution. The as-obtained mixture was stirred at -70 °C for 0.5 h, and subsequently stirred at room temperature for 3 h. After the addition of a solution of $BiCl_3$ (12.87 g, 40.8 mmol) in dried ethyl ether (120 mL) at -80 °C, the resulting mixture was stirred overnight with the temperature gradually rising to room temperature. After the removal of solvent under vacuum and toluene extraction, the in soluble material was filtered out, and the as-obtained organic layer was washed with deionized H_2O and dried using anhydrous $MgSO_4$. The solvent was removed under reduced pressure to leave an oily substance that was yellow in color. The yellow substance was dissolved in CH_2Cl_2 and recrystallized from CH_2Cl_2/hexane to give $S(CH_2C_6H_4)_2BiCl$ in the form of colorless crystals.

$[S(CH_2C_6H_4)_2Bi\ (OH_2)]^+[C_8F_{17}SO_3]^-$:[14] To a solution of $S(CH_2C_6H_4)_2BiCl$ (1.0 mmol) in THF (20 mL), a solution of $AgC_8F_{17}SO_3$ (1.0 mmol) in THF (10 mL) was added. After the mixture was stirred in darkness at RT for 3 h, it was subject to filtration. The filtrate was then mixed with hexane (1.0 mL), and after 24 h there was the formation of colorless crystals. Crystals suitable for X-ray diffraction analysis were obtained by recrystallization of $[S(CH_2C_6H_4)_2Bi\ (OH_2)]^+[C_8F_{17}SO_3]^-$ from THF–hexane solution.

2.9 Procedure for organobismuth-catalyzed aldol condensation[15]

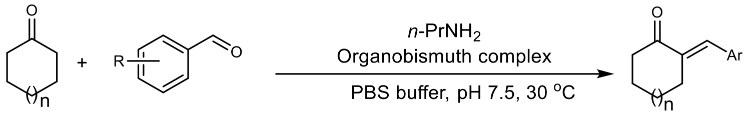

n = 0, 1, 2

To a 25-mL round-bottomed flask were added organobismuth catalyst (0.01 mmol), *n*-$PrNH_2$ (0.1 mmol), corresponding aryl aldehyde (1.0 mmol) and cyclic ketone (1.2 mmol), and PBS (100 mM, pH 7.5). Then the mixture was stirred at 30 °C as monitored by TLC analysis until the aldehyde as well as the intermediate imine obtained from aldehyde and *n*-$PrNH_2$ were consumed completely. Then the mixture was extracted with Et_2O (10 mL x 3), and the combined organic layer was evaporated under vacuum. The resulting residue was subject to column chromatography on silica gel (200–300 meshes) (petroleum ether/ethyl acetate=5/1, v/v).

2.10 Procedure for enzymatic reduction

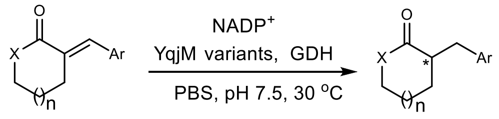

X = C, O, NH; n = 0, 1, 2

A 25-mL round-bottomed flask was charged with substrate (exocyclic α,β-unsaturated carbonyl compounds, 10 mM), YqjM variants (6.0 μM), GDH (12 μM), $NADP^+$ (50 μM), glucose (20 mM), and PBS (5 mL, 100 mM, pH 7.5) containing 10% v/v isooctane. The flask was capped tightly, and the reaction mixture was stirred at 30 °C for 24 h. Upon completion, the reaction mixture was extracted with Et_2O (5 mL x 3), and the organic phase was washed with saturated brine, and dried over anhydrous Na_2SO_4. Volatiles were removed under a vacuum. The crude product was purified by column chromatography using petroleum ether and ethyl acetate (8/1, v/v) as eluent to afford the desired product. The values of ee were determined by GC (Agilgent CP-Chirasil Dex CB (df = 0.25 μm, 0.32 mm i.d. × 25 m); carrier gas, N_2 (flow 30 mL/min); injection temp, 180 °C; initial column temperature 120 °C, 5 °C/min to 150 °C, hold for 2 min, then 1 °C/min to 200 °C, hold for 5 min).

2.11 One-pot concurrent chemoenzymatic cascade

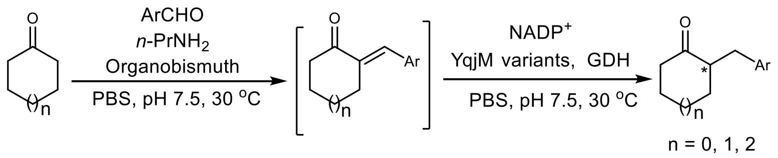

A 25-mL round-bottomed flask was charged with cyclopentanone (12 mM), benzaldehyde (10 mM), *n*-$PrNH_2$ (1.0 mM), organobismuth catalyst (0.1 mM), YqjM variants (6.0 μM), GDH (12 μM), $NADP^+$ (50 μM), glucose (20 mM), and PBS (5 mL, 100 mM, pH 7.5) containing 10% v/v isooctane. The flask was capped tightly, and the reaction mixture was stirred at 30 °C for 24 h. Upon completion, the reaction mixture was extracted with Et_2O (5 mL x 3), and the organic phase was washed with saturated brine, and dried over anhydrous Na_2SO_4. Volatiles were removed under a vacuum. The crude product was purified by column chromatography using petroleum ether and ethyl acetate (8/1, v/v) as eluent to afford the desired product. The values of ee were determined by GC (*vide infra*).

2.11 Procedure for multienzyme cascades[16]

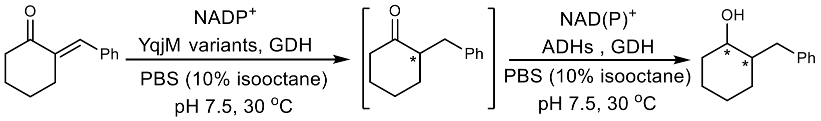

Substrate (10 mM), $NADP^+$ (50 μM) and glucose (20 mM), YqjM variants (6.0 μM) and GDH (12 μM), ADHs (0.5 mM) and PBS (5.0 mL, 100 mM, pH 7.5) containing 10% v/v isooctane were added to a 25-mL round-bottomed flask. In the system combining with ADA-A, additional NAD^+ (0.1 mM) was added. The mixture was stirred at 30 °C for 24 h. After the reaction was completed, the reaction solution was extracted with Et_2O (5 mL x 3), and the organic phase was dried using anhydrous Na_2SO_4. The solvent was concentrated in vacuo to obtain the crude products. The products were purified by column chromatography using petroleum ether and ethyl acetate (4/1, v/v) as eluent. The values of ee were determined by HPLC (Chiralpak OD column, UV detection at 254 nm, eluent: *n*-hexane/2-propanol =90:10, flow 1 mL/min, 30 °C).

2.12 Procedure for the synthesis of ***racemic-trans*-5**

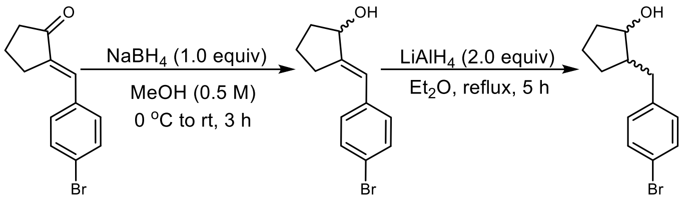

1g *trans*-**5**

The reduction of C=O double bond according to the procedure reported by Lautens:[17] A 50 mL round-bottomed flask equipped with a magnetic stir bar was charged with the exocyclic enone **1g** (5 mmol) and methanol (10 mL). At 0 °C, $NaBH_4$ (5 mmol) was added portion-wise. The mixture was stirred for 3 hours at room temperature or until full conversion is observed via TLC monitoring. The solution was then concentrated, redissolved in ethyl acetate or dichloromethane (15 mL), washed with water (10 mL), and brine (10 mL). The organic phase was then dried over $MgSO_4$, filtered, concentrated *in vacuo*, and carried on to the next step.

The reduction of C=C double bond according to the procedure reported by Lautens:[18] A solution of the allyl alcohol intermediate obtained in the first step in 10 mL Et_2O was added slowly to a 50 mL dry three-neck flask containing $LiAlH_4$ (10 mmol) and 10 mL dry Et_2O under N_2 atmosphere at 0 °C. The mixture was stirred at room temperature for 0.5 h, and then was refluxed for 5 h. After cooling to 0 °C the reaction was quenched with small amount of water, the reaction mixture was treated with 1N HCl (30 mL) and extracted with ethyl acetate (3 × 30 mL). The combined organic layer was washed with water, saturated $NaHCO_3$ solution in sequence, and dried over anhydrous Na_2SO_4. The solvent was removed in vacuum and the residual was purified by silica gel column chromatography (EtOAc/petroleum ether 1:4) to yield *racemic-trans*-**5**.

2.13 Multienzyme cascade for the synthesis of (1*S*,2*R*)-5

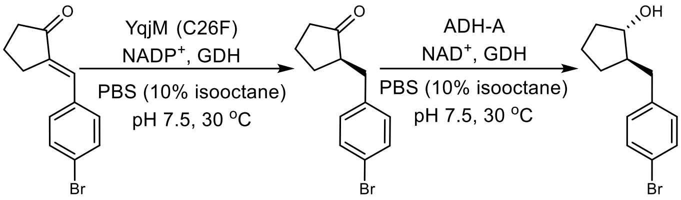

1g (*R*)-**1gp** (1*S*,2*R*)-**5**

Exocyclic enone **1g** (10 mM), $NADP^+$ (1 mM), NAD^+ (1 mM) and glucose (20 mM), YqjM (C26F, 0.1 mM) and GDH (0.2 mM), ADH-A (0.5 mM) and PBS (5.0 mL, 100 mM, pH 7.5) containing 10% v/v isooctane were added to a 25-mL round-bottomed flask. The mixture was stirred at 30 °C for 48 h. After the reaction was completed, the reaction solution was extracted with Et_2O (5 mL x 3), and the organic phase was dried using anhydrous Na_2SO_4. The solvent was concentrated in vacuo to obtain the crude products. The products were purified by column chromatography using petroleum ether and ethyl acetate (4/1, v/v) as eluent. The values of ee were determined by HPLC (Chiralpak AD-3 column, UV detection at 210 nm, eluent: *n*-hexane/2-propanol =80:20, flow 1 mL/min). ^{1}H NMR (400 MHz, $CDCl_3$) δ 7.40 (d, *J* = 8.3 Hz, 2H), 7.07 (d, *J* = 8.3 Hz, 2H), 3.88 (dd, *J* = 11.9, 5.8 Hz, 1H), 2.75 (dd, *J* = 13.6, 6.5 Hz, 1H), 2.45 (dd, *J* = 13.6, 8.7 Hz, 1H), 1.96 (tt, *J* = 12.7, 6.7 Hz, 2H), 1.85 – 1.75 (m, 1H), 1.74 – 1.66 (m, 1H), 1.63 – 1.53 (m, 2H), 1.40 (s, 1H), 1.24 (dd, *J* = 8.5, 5.2 Hz, 1H). ^{13}C NMR (100 MHz, $CDCl_3$) δ 140.31, 131.64, 130.80, 119.89, 78.59, 49.87, 39.30, 34.51, 29.82, 21.65.

1. NMR and GC/HPLC data

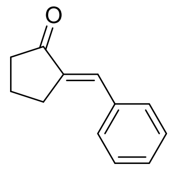

^{1}H NMR (400 MHz, CDCl$_3$) δ 7.56 (d, *J* = 7.2 Hz, 2H), 7.44 (s, 1H), 7.42 (s, 2H), 7.39 (s, 1H), 3.00 (t, *J* = 6.8 Hz, 2H), 2.43 (t, *J* = 7.9 Hz, 2H), 2.06 (p, *J* = 7.6 Hz, 2H). ^{13}C NMR (100 MHz, CDCl$_3$) δ 208.11, 136.14, 135.58, 132.30, 130.54, 129.37, 128.75, 37.82, 29.39, 20.23.

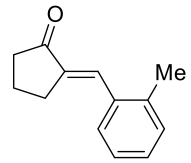

^{1}H NMR (400 MHz, CDCl$_3$) δ 7.64 (s, 1H), 7.46 (d, *J* = 7.0 Hz, 2H), 7.30 (d, *J* = 7.5 Hz, 2H), 2.93 (s, 2H), 2.45 – 2.44 (m, 3H), 2.03 (p, *J* = 7.5 Hz, 4H). ^{13}C NMR (100 MHz, CDCl$_3$) δ 208.05, 138.97, 136.91, 134.40, 130.60, 129.90, 129.21, 128.72, 125.85, 38.13, 29.47, 20.57, 20.07.

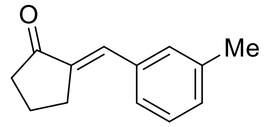

^{1}H NMR (400 MHz, CDCl$_3$) δ 7.60 (s, 1H), 7.42 (d, *J* = 5.7 Hz, 1H), 7.26 – 7.20 (m, 3H), 2.88 (td, *J* = 7.0, 2.5 Hz, 2H), 2.46 – 2.41 (m, 1H), 2.40 (s, 2H), 2.39 (d, *J* = 5.4 Hz, 2H), 2.03 – 1.94 (m, 2H). ^{13}C NMR (100 MHz, CDCl$_3$) δ 207.93, 138.90, 136.85, 134.33, 130.55, 129.79, 129.17, 128.67, 125.81, 38.08, 29.43, 20.53, 20.02.

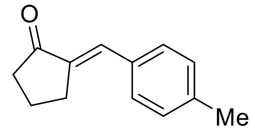

^{1}H NMR (400 MHz, CDCl$_3$) δ 7.43 (d, *J* = 8.1 Hz, 2H), 7.37 (t, *J* = 2.4 Hz, 1H), 7.22 (d, *J* = 8.0 Hz, 2H), 2.96 (td, *J* = 7.2, 2.8 Hz, 2H), 2.40 (d, *J* = 7.9 Hz, 2H), 2.38 (s, 3H), 2.02 (p, *J* = 7.6 Hz, 2H). ^{13}C NMR (100 MHz, CDCl$_3$) δ 208.17, 139.73, 135.11, 132.76, 132.37, 130.58, 129.48, 37.80, 29.37, 21.49, 20.19.

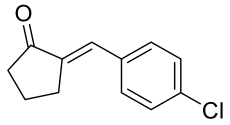

^{1}H NMR (400 MHz, CDCl$_3$) δ 7.46 (d, *J* = 8.5 Hz, 2H), 7.41 – 7.36 (m, 2H), 7.33 (t, *J* = 2.7 Hz, 1H), 2.95 (td, *J* = 7.2, 2.7 Hz, 2H), 2.42 (t, *J* = 7.9 Hz, 2H), 2.05 (p, *J* = 7.6 Hz, 2H). ^{13}C NMR (100 MHz, CDCl$_3$) δ 207.85, 136.53, 135.24, 134.02, 131.62, 130.89, 128.98, 37.74, 29.29, 20.14.

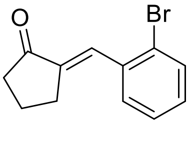

^{1}H NMR (400 MHz, CDCl$_3$) δ 7.67 – 7.64 (m, 1H), 7.64 – 7.60 (m, 1H), 7.48 (dd, *J* = 7.8, 1.4 Hz, 1H), 7.37 – 7.31 (m, 1H), 7.20 (td, *J* = 7.8, 1.5 Hz, 1H), 2.87 (td, *J* = 7.1, 2.7 Hz, 2H), 2.43 (t, *J* = 7.8 Hz,

2H), 2.01 (p, *J* = 7.5 Hz, 2H). ^{13}C NMR (100 MHz, $CDCl_3$) δ 207.45, 138.34, 135.47, 133.28, 130.73, 130.27, 130.04, 127.20, 126.28, 37.97, 29.19, 20.41.

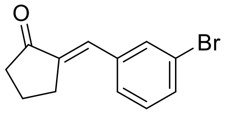

^{1}H NMR (400 MHz, $CDCl_3$) δ 7.69 – 7.64 (m, 1H), 7.64 – 7.58 (m, 1H), 7.48 (dd, *J* = 7.8, 1.7 Hz, 1H), 7.34 (td, *J* = 7.5, 1.3 Hz, 1H), 7.20 (td, *J* = 7.7, 1.7 Hz, 1H), 2.87 (td, *J* = 7.2, 2.8 Hz, 2H), 2.43 (t, *J* = 7.8 Hz, 2H), 2.01 (p, *J* = 7.5 Hz, 2H). ^{13}C NMR (100 MHz, $CDCl_3$) δ 207.47, 138.34, 135.47, 133.28, 130.73, 130.27, 130.04, 127.20, 126.28, 37.97, 29.19, 20.41.

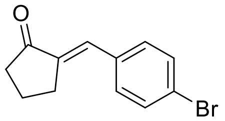

^{1}H NMR (400 MHz, $CDCl_3$) δ 7.54 (d, *J* = 2.0 Hz, 1H), 7.52 (s, 1H), 7.39 (s, 1H), 7.37 (s, 1H), 7.29 (t, *J* = 2.7 Hz, 1H), 2.92 (td, *J* = 7.2, 2.7 Hz, 2H), 2.40 (t, *J* = 7.9 Hz, 2H), 2.04 (p, *J* = 7.6 Hz, 2H). ^{13}C NMR (100 MHz, $CDCl_3$) δ 207.77, 136.70, 134.45, 131.94, 131.82, 130.91, 123.60, 37.73, 29.30, 20.13.

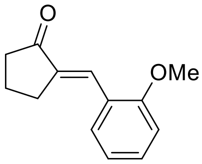

^{1}H NMR (400 MHz, $CDCl_3$) δ 7.84 (s, 1H), 7.50 (d, *J* = 7.6 Hz, 1H), 7.37 (t, *J* = 7.9 Hz, 1H), 7.01 (t, *J* = 7.6 Hz, 1H), 6.94 (d, *J* = 8.3 Hz, 1H), 3.89 (s, 3H), 3.02 – 2.88 (m, 2H), 2.43 (t, *J* = 7.7 Hz, 2H), 2.09 – 1.97 (m, 2H). ^{13}C NMR (126 MHz, $CDCl_3$) δ 208.09, 158.90, 136.02, 130.85, 129.67, 126.94, 124.53, 120.26, 110.77, 55.48, 37.92, 29.49, 20.40.

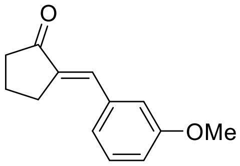

^{1}H NMR (400 MHz, $CDCl_3$) δ 7.37 (s, 1H), 7.34 (d, *J* = 8.2 Hz, 1H), 7.15 (d, *J* = 7.8 Hz, 1H), 7.08 (s, 1H), 6.94 (d, *J* = 8.0 Hz, 1H), 3.88 – 3.83 (m, 3H), 3.00 (t, *J* = 7.0 Hz, 2H), 2.43 (t, *J* = 8.1 Hz, 2H), 2.05 (t, *J* = 7.6 Hz, 2H). ^{13}C NMR (126 MHz, $CDCl_3$) δ 207.72, 159.62, 136.78, 136.28, 131.95, 129.60, 122.97, 115.68, 114.92, 55.12, 37.64, 29.27, 20.06.

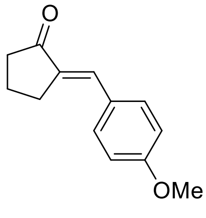

^{1}H NMR (400 MHz, $CDCl_3$) δ 7.36 (dd, *J* = 11.8, 5.4 Hz, 2H), 7.18 – 7.06 (m, 2H), 6.94 (d, *J* = 8.0 Hz, 1H), 3.85 (d, *J* = 2.5 Hz, 3H), 3.00 (t, *J* = 7.1 Hz, 2H), 2.43 (t, *J* = 8.1 Hz, 2H), 2.05 (p, *J* = 7.7 Hz, 2H). ^{13}C NMR (126 MHz, $CDCl_3$) δ 207.89, 160.52, 133.65, 132.27, 132.02, 128.16, 114.18, 55.27, 37.66, 29.20, 20.07.

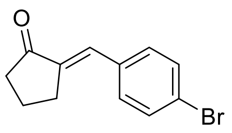

^{1}H NMR (400 MHz, $CDCl_3$) δ 7.54 (d, *J* = 8.4 Hz, 2H), 7.39 (d, *J* = 8.4 Hz, 2H), 7.31 (s, 1H), 2.94 (td, *J* = 7.2, 2.4 Hz, 2H), 2.41 (t, *J* = 7.8 Hz, 2H), 2.05 (p, *J* = 7.6 Hz, 2H). ^{13}C NMR (100 MHz, $CDCl_3$) δ 207.83, 136.70, 134.45, 131.95, 131.83, 130.94, 123.61, 37.74, 29.31, 20.13.

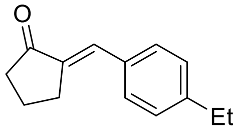

^{1}H NMR (400 MHz, $CDCl_3$) δ 7.50 (d, *J* = 7.6 Hz, 2H), 7.42 (s, 1H), 7.29 (d, *J* = 7.9 Hz, 2H), 3.15 – 2.91 (m, 2H), 2.71 (q, *J* = 7.5 Hz, 2H), 2.44 (t, *J* = 7.9 Hz, 2H), 2.06 (p, *J* = 7.6 Hz, 2H), 1.29 (t, *J* = 7.2 Hz, 3H). ^{13}C NMR (126 MHz, $CDCl_3$) δ 208.16, 145.99, 135.16, 133.01, 132.39, 130.72, 130.71, 128.29, 128.28, 37.76, 29.36, 28.81, 20.17, 15.36.

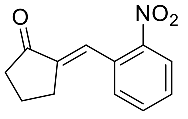

^{1}H NMR (400 MHz, $CDCl_3$) δ 8.07 (d, *J* = 8.0 Hz, 1H), 7.68 (d, *J* = 8.3 Hz, 1H), 7.65 (s, 1H), 7.55 (s, 1H), 7.52 (d, *J* = 7.7 Hz, 1H), 2.81 (td, *J* = 7.1, 2.8 Hz, 2H), 2.46 (t, *J* = 7.8 Hz, 2H), 2.03 (p, *J* = 7.5 Hz, 2H). ^{13}C NMR (100 MHz, $CDCl_3$) δ 206.79, 148.92, 139.55, 133.03, 131.31, 130.62, 129.39, 127.34, 124.94, 38.03, 28.81, 20.31.

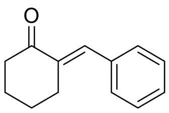

^{1}H NMR (400 MHz, $CDCl_3$) δ 7.54 (s, 1H), 7.47 – 7.38 (m, 4H), 7.37 – 7.27 (m, 1H), 2.87 (t, *J* = 6.6 Hz, 2H), 2.57 (t, *J* = 6.8 Hz, 2H), 1.96 (t, *J* = 6.1 Hz, 2H), 1.79 (t, *J* = 6.1 Hz, 2H). ^{13}C NMR (100 MHz, $CDCl_3$) δ 201.94, 136.72, 135.70, 135.66, 130.36, 128.60, 128.39, 40.39, 29.00, 23.93, 23.43.

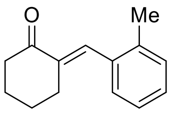

^{1}H NMR (400 MHz, $CDCl_3$) δ 7.55 (s, 1H), 7.30 – 7.04 (m, 4H), 2.76 – 2.65 (m, 2H), 2.58 (t, *J* = 6.7 Hz, 2H), 2.32 (s, 3H), 1.96 (dt, *J* = 13.1, 6.6 Hz, 2H), 1.76 (dd, *J* = 12.3, 6.3 Hz, 2H). ^{13}C NMR (100 MHz, $CDCl_3$) δ 202.17, 137.82, 137.49, 134.73, 134.08, 130.16, 129.02, 128.37, 125.35, 40.63, 28.85, 24.14, 23.78, 20.03.

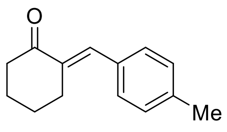

^{1}H NMR (400 MHz, $CDCl_3$) δ 7.43 (s, 1H), 7.25 (d, *J* = 7.8 Hz, 2H), 7.14 (s, 2H), 2.77 (t, *J* = 6.3 Hz, 2H), 2.46 (t, *J* = 6.7 Hz, 2H), 2.30 (s, 3H), 1.85 (q, *J* = 6.6, 6.2 Hz, 2H), 1.74 – 1.66 (m, 2H). ^{13}C NMR

(100 MHz, $CDCl_3$) δ 201.72, 138.81, 135.83, 132.82, 130.47, 129.12, 77.29, 40.28, 29.01, 23.88, 23.36, 21.39.

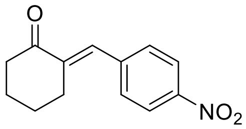

^1H NMR (400 MHz, $CDCl_3$) δ 8.25 (d, *J* = 8.8 Hz, 2H), 7.53 (d, *J* = 8.6 Hz, 2H), 7.47 (s, 1H), 2.83 (td, *J* = 6.5, 2.2 Hz, 2H), 2.59 (t, *J* = 6.7 Hz, 2H), 1.98 (qd, *J* = 6.6, 3.6 Hz, 2H), 1.88 – 1.73 (m, 2H). ^{13}C NMR (100 MHz, $CDCl_3$) δ 201.28, 147.18, 142.21, 139.96, 132.51, 130.70, 123.58, 40.47, 29.07, 23.77, 23.33.

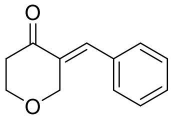

^1H NMR (400 MHz, $CDCl_3$) δ 7.65 (s, 1H), 7.46 – 7.37 (m, 3H), 7.30 (d, *J* = 6.7 Hz, 2H), 4.88 (d, *J* = 2.0 Hz, 2H), 4.10 (t, *J* = 6.1 Hz, 2H), 2.71 (t, *J* = 6.1 Hz, 2H). ^{13}C NMR (100 MHz, $CDCl_3$) δ 196.15, 136.11, 134.34, 133.26, 130.59, 129.50, 128.70, 68.70, 65.51, 39.77.

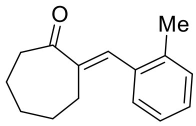

^1H NMR (400 MHz, $CDCl_3$) δ 7.62 (s, 1H), 7.20 (s, 2H), 7.15 (dd, *J* = 27.1, 5.8 Hz, 2H), 2.75 – 2.69 (m, 2H), 2.56 – 2.50 (m, 2H), 2.27 (s, 3H), 1.77 (dt, *J* = 6.2, 2.9 Hz, 4H), 1.72 – 1.65 (m, 2H). ^{13}C NMR (100 MHz, $CDCl_3$) δ 215.67, 138.22, 136.43, 130.36, 129.71, 126.22, 125.79, 52.34, 43.05, 34.80, 30.35, 29.38, 28.64, 24.39, 19.63. ^{13}C NMR (100 MHz, $CDCl_3$) δ 204.43, 140.87, 137.26, 135.56, 130.07, 128.49, 128.03, 125.51, 43.64, 31.34, 30.09, 27.74, 25.64, 20.03.

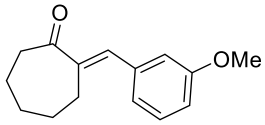

^1H NMR (400 MHz, $CDCl_3$) δ 7.48 (s, 1H), 7.33 – 7.27 (m, 1H), 6.94 (d, *J* = 7.7 Hz, 1H), 6.87 (d, *J* = 7.6 Hz, 2H), 3.82 (s, 3H), 2.71 (t, *J* = 9.6 Hz, 4H), 1.80 (s, 6H). ^{13}C NMR (100 MHz, $CDCl_3$) δ 204.91, 159.47, 141.03, 137.40, 135.53, 129.41, 121.75, 114.78, 113.75, 55.24, 43.46, 31.30, 29.98, 27.78, 25.46.

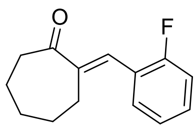

^1H NMR (400 MHz, $CDCl_3$) δ 7.52 (s, 1H), 7.32 – 7.23 (m, 2H), 7.17 – 7.06 (m, 2H), 2.72 (t, *J* = 5.5 Hz, 2H), 2.62 – 2.56 (m, 2H), 1.83 – 1.69 (m, 6H). ^{13}C NMR (100 MHz, $CDCl_3$) δ 204.04, 160.48 (d, *J* = 248 Hz), 142.64, 130.36 (d, *J* = 2.8 Hz), 129.93 (d, *J* = 8.3 Hz), 128.80 (d, *J* = 2.9 Hz), 123.85 (d, *J* = 3.6 Hz), 115.74 (d, *J* = 22 Hz), 43.48, 31.25, 29.64, 28.15, 25.57.

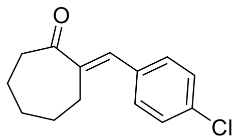

^{1}H NMR (400 MHz, $CDCl_3$) δ 7.44 (s, 1H), 7.37 – 7.32 (m, 2H), 7.30 – 7.20 (m, 2H), 2.68 (dd, *J* = 24.5, 10.0 Hz, 4H), 1.97 – 1.63 (m, 6H). ^{13}C NMR (100 MHz, $CDCl_3$) δ 204.59, 141.30, 134.46, 134.26, 134.02, 130.66, 128.66, 43.40, 31.25, 29.88, 27.66, 25.42.

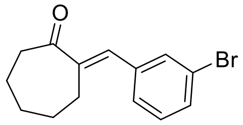

^{1}H NMR (400 MHz, $CDCl_3$) δ 7.48 (s, 1H), 7.46 – 7.33 (m, 2H), 7.26 (s, 1H), 7.25 (s, 1H), 2.81 – 2.69 (m, 2H), 2.66 (d, *J* = 9.5 Hz, 2H), 1.79 (d, *J* = 8.7 Hz, 6H). ^{13}C NMR (100 MHz, $CDCl_3$) δ 204.40, 141.98, 138.21, 133.86, 132.02, 130.99, 129.93, 127.87, 122.47, 43.40, 31.20, 29.88, 27.66, 25.45.

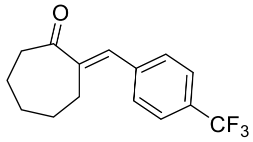

^{1}H NMR (400 MHz, $CDCl_3$) δ 7.63 (dd, *J* = 8.0, 4.1 Hz, 2H), 7.50 (d, *J* = 2.7 Hz, 1H), 7.42 (dd, *J* = 7.9, 2.4 Hz, 2H), 2.79 – 2.69 (m, 2H), 2.65 (d, *J* = 6.9 Hz, 2H), 1.85 – 1.75 (m, 6H). ^{13}C NMR (100 MHz, $CDCl_3$) δ 204.41, 204.37, 142.57, 139.75, 133.90 (d, *J* = 3.8 Hz), 129.49 (d, *J* = 5.5 Hz), 125.33 (dd, *J* = 5.9, 2.6 Hz), 122.67, 43.38 (d, *J* = 1.6 Hz), 31.21, 29.93, 27.68, 25.43.

2-benzylcyclopentan-1-one (1ap)

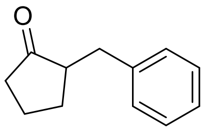

^{1}H NMR (400 MHz, $CDCl_3$) δ 7.35 – 7.18 (m, 5H), 3.19 (dd, *J* = 13.8, 4.0 Hz, 1H), 2.58 (dd, *J* = 13.9, 9.4 Hz, 1H), 2.46 – 2.31 (m, 2H), 2.22 – 2.13 (m, 1H), 2.13 – 1.51 (m, 4H). ^{13}C NMR (100 MHz, $CDCl_3$) δ 220.41, 140.07, 128.97, 128.50, 126.23, 51.11, 38.28, 35.66, 29.21, 20.61. GC conditions: Agilgent CP-Chirasil Dex CB (df = 0.25 μm, 0.32 mm i.d. × 25 m); carrier gas, N_2 (flow 30 mL/min); injection temp, 180 °C; initial column temperature 100 °C, then progress rate, 5 °C/min; final column temperature, 200 °C for 5min; t_R = 10.641 min and t_S = 10.334 min.

2-(2-methylbenzyl)cyclopentan-1-one (1bp)

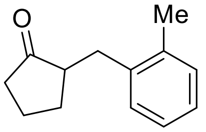

^{1}H NMR (400 MHz, $CDCl_3$) δ 7.22 – 7.10 (m, 4H), 3.27 (d, *J* = 8.0 Hz, 1H), 2.49 (d, *J* = 13.1 Hz, 1H), 2.46 – 2.40 (m, 1H), 2.38 (d, *J* = 2.6 Hz, 1H), 2.35 (s, 3H), 2.26 – 1.97 (m, 3H), 1.86 – 1.67 (m, 1H),

1.60 (dt, J = 12.2, 5.4 Hz, 1H). ^{13}C NMR (100 MHz, $CDCl_3$) δ 220.35, 138.42, 136.14, 130.39, 129.36, 126.32, 125.97, 50.04, 38.11, 32.83, 29.61, 20.62, 19.49. GC conditions: Agilgent CP-Chirasil Dex CB (df = 0.25 μm, 0.32 mm i.d. × 25 m); carrier gas, N_2 (flow 30 mL/min); injection temp, 180 °C; initial column temperature 100 °C, then progress rate, 5 °C/min; final column temperature, 200 °C for 5min; t_R = 10.95 min and t_S = 11.548 min.

2-(3-methylbenzyl)cyclopentan-1-one (1cp)

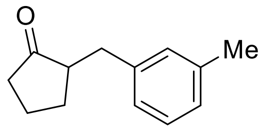

^{1}H NMR (400 MHz, $CDCl_3$) δ 7.43 (t, J = 7.5 Hz, 1H), 7.28 (d, J = 7.5 Hz, 1H), 3.38 (d, J = 13.8 Hz, 1H), 2.80 – 2.69 (m, 1H), 2.58 (d, J = 3.0 Hz, 5H), 2.36 (dd, J = 19.2, 9.1 Hz, 2H), 2.21 (d, J = 6.4 Hz, 1H), 1.99 (d, J = 10.2 Hz, 1H), 1.83 (dd, J = 19.0, 8.7 Hz, 1H). ^{13}C NMR (100 MHz, $CDCl_3$) δ 220.33, 140.00, 138.03, 129.70, 128.33, 126.92, 125.91, 51.07, 38.24, 35.56, 29.23, 21.42, 20.57, 49.65. GC conditions: Agilgent CP-Chirasil Dex CB (df = 0.25 μm, 0.32 mm i.d. × 25 m); carrier gas, N_2 (flow 30 mL/min); injection temp, 180 °C; initial column temperature 100 °C, then progress rate, 5 °C/min; final column temperature, 200 °C for 5 min; t_R = 14.396min (minor) and t_S = 14.24 min (major).

2-(4-methylbenzyl)cyclopentan-1-one (1dp)

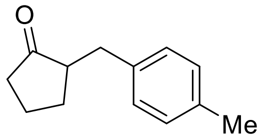

^{1}H NMR (400 MHz, $CDCl_3$) δ 7.07 (q, J = 8.0 Hz, 4H), 3.10 (dd, J = 13.9, 4.2 Hz, 1H), 2.51 (dd, J = 13.9, 9.5 Hz, 1H), 2.32 (s, 5H), 2.10 (ddd, J = 18.8, 10.3, 8.6 Hz, 2H), 1.95 (dt, J = 9.0, 2.7 Hz, 1H), 1.81 – 1.64 (m, 1H), 1.63 – 1.48 (m, 1H). ^{13}C NMR (100 MHz, $CDCl_3$) δ 220.41, 136.88, 135.66, 129.12, 128.80, 51.11, 38.28, 35.16, 29.15, 21.02, 20.57. GC conditions: Agilgent CP-Chirasil Dex CB (df = 0.25 μm, 0.32 mm i.d. × 25 m); carrier gas, N_2 (flow 30 mL/min); injection temp, 180 °C; initial column temperature 100 °C, then progress rate, 5 °C/min; final column temperature, 200 °C for 5 min; t_R = 16.519min and t_S = 16.114min.

2-(2-bromobenzyl)cyclopentan-1-one (1ep)

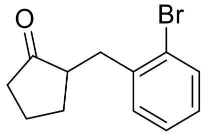

^{1}H NMR (400 MHz, $CDCl_3$) δ 7.53 (d, J = 7.4 Hz, 1H), 7.28 – 7.18 (m, 2H), 7.07 (td, J = 7.4, 6.5, 2.6 Hz, 1H), 3.34 (dd, J = 13.8, 4.4 Hz, 1H), 2.63 (dd, J = 13.8, 9.7 Hz, 1H), 2.56 – 2.42 (m, 1H), 2.36 (dd, J

= 17.5, 7.9 Hz, 1H), 2.22 – 1.94 (m, 3H), 1.83 – 1.66 (m, 1H), 1.58 (qd, *J* = 11.4, 11.0, 6.4 Hz, 1H). ^{13}C NMR (100 MHz, $CDCl_3$) δ 219.74, 139.56, 132.93, 131.02, 127.95, 127.45, 124.62, 49.68, 37.99, 35.61, 29.25, 20.55. GC conditions: Agilgent CP-Chirasil Dex CB (df = 0.25 μm, 0.32 mm i.d. × 25 m); carrier gas, N_2 (flow 30 mL/min); injection temp, 180 °C; initial column temperature 120 °C, 5 °C/min to 150 °C, then 2 °C/min to 170 °C and 1 °C/min to 200 °C, hold for 5 min; t_R = 39.63 min and t_S = 39.356 min.

2-(3-bromobenzyl)cyclopentan-1-one (1fp)

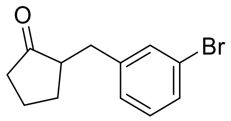

^{1}H NMR (400 MHz, $CDCl_3$) δ 7.43 – 7.31 (m, 2H), 7.21 – 7.08 (m, 2H), 3.15 (dd, *J* = 13.9, 4.2 Hz, 1H), 2.52 (dd, *J* = 13.9, 9.4 Hz, 1H), 2.46 – 2.22 (m, 2H), 2.21 – 2.07 (m, 2H), 2.00 (dddd, *J* = 15.3, 6.5, 4.8, 2.4 Hz, 1H), 1.85 – 1.69 (m, 1H), 1.60 – 1.49 (m, 1H). ^{13}C NMR (100 MHz, $CDCl_3$) δ 219.65, 142.41, 131.88, 130.02, 129.35, 127.57, 122.48, 50.83, 38.09, 35.22, 29.16, 20.52. GC conditions: Agilgent CP-Chirasil Dex CB (df = 0.25 μm, 0.32 mm i.d. × 25 m); carrier gas, N_2 (flow 30 mL/min); injection temp, 180 °C; initial column temperature 120 °C, 5 °C/min to 150 °C, then 2 °C/min to 170 °C and 1 °C/min to 200 °C, hold for 5 min; t_R = 27.013 min and t_S = 26.790 min.

2-(2-bromobenzyl)cyclopentan-1-one (1gp)

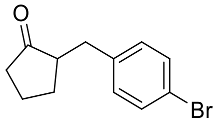

^{1}H NMR (400 MHz, $CDCl_3$) δ 7.25 (d, *J* = 7.8 Hz, 2H), 6.89 (d, *J* = 7.8 Hz, 2H), 2.93 (dd, *J* = 14.0, 4.3 Hz, 1H), 2.38 (dd, *J* = 14.0, 9.1 Hz, 1H), 2.20 (dd, *J* = 19.0, 8.6 Hz, 2H), 1.94 (dt, *J* = 19.2, 9.8 Hz, 2H), 1.80 (s, 1H), 1.66 – 1.53 (m, 1H), 1.38 (tt, *J* = 11.6, 5.8 Hz, 1H). ^{13}C NMR (100 MHz, $CDCl_3$) δ 219.76, 138.94, 131.50, 130.69, 120.02, 50.79, 38.14, 34.94, 29.03, 20.52. GC conditions: Agilgent CP-Chirasil Dex CB (df = 0.25 μm, 0.32 mm i.d. × 25 m); carrier gas, N_2 (flow 30 mL/min); injection temp, 180 °C; initial column temperature 120 °C, 5 °C/min to 150 °C, hold for 2 min, then 1 °C/min to 200 °C, hold for 5 min; t_R = 27.048 min and t_S = 26.83 min.

2-(4-chlorobenzyl)cyclopentan-1-one (1hp)

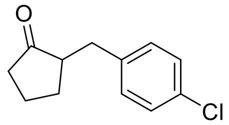

^{1}H NMR (400 MHz, $CDCl_3$) δ 7.24 (d, *J* = 8.4 Hz, 2H), 7.09 (d, *J* = 8.3 Hz, 2H), 3.09 (dd, *J* = 14.0, 4.3 Hz, 1H), 2.54 (dd, *J* = 14.0, 9.2 Hz, 1H), 2.35 (dd, *J* = 20.1, 7.9 Hz, 2H), 2.09 (ddd, *J* = 19.0,

10.6, 8.9 Hz, 2H), 1.96 (ddd, J = 12.8, 8.9, 6.5 Hz, 1H), 1.82 – 1.65 (m, 1H), 1.59 – 1.45 (m, 1H). ^{13}C NMR (100 MHz, $CDCl_3$) δ 219.89, 138.43, 132.00, 130.30, 128.56, 50.88, 38.17, 34.87, 29.03, 20.53. GC conditions: Agilgent CP-Chirasil Dex CB (df = 0.25 μm, 0.32 mm i.d. × 25 m); carrier gas, N_2 (flow 30 mL/min); injection temp, 180 °C; initial column temperature 120 °C, 5 °C/min to 150 °C, then 2 °C/min to 170 °C and 1 °C/min to 200 °C, hold for 5 min; t_R = 31.945 min and t_S = 31.545 min.

2-(2-methoxybenzyl)cyclopentan-1-one (1ip)

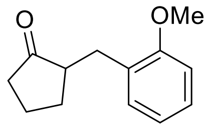

^{1}H NMR (400 MHz, $CDCl_3$) δ 7.19 (td, J = 7.8, 1.8 Hz, 1H), 7.11 (dd, J = 7.3, 1.7 Hz, 1H), 6.91 – 6.80 (m, 2H), 3.80 (s, 3H), 3.22 (d, J = 9.0 Hz, 1H), 2.51 – 2.39 (m, 2H), 2.39 – 2.26 (m, 1H), 2.13 (ddd, J = 18.8, 10.1, 8.6 Hz, 1H), 1.95 (dddd, J = 11.9, 9.0, 6.1, 3.0 Hz, 2H), 1.80 – 1.63 (m, 1H), 1.62 – 1.46 (m, 1H). ^{13}C NMR (100 MHz, $CDCl_3$) δ 220.87, 157.58, 130.52, 128.49, 127.47, 120.37, 110.21, 55.17, 49.60, 38.16, 30.20, 29.34, 20.57. GC conditions: Agilgent CP-Chirasil Dex CB (df = 0.25 μm, 0.32 mm i.d. × 25 m); carrier gas, N_2 (flow 30 mL/min); injection temp, 180 °C; initial column temperature 120 °C, 5 °C/min to 150 °C, hold for 2 min, then 1 °C/min to 200 °C, hold for 5 min; t_R = 25.872 min and t_S = 25.715 min.

2-(3-methoxybenzyl)cyclopentan-1-one (1jp)

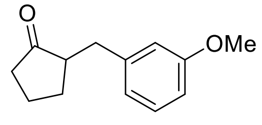

^{1}H NMR (400 MHz, $CDCl_3$) δ 7.20 (t, J = 7.8 Hz, 1H), 6.79 – 6.69 (m, 3H), 3.79 (s, 3H), 3.13 (dd, J = 13.8, 4.1 Hz, 1H), 2.50 (dd, J = 13.7, 9.6 Hz, 1H), 2.45 – 2.28 (m, 2H), 2.13 (dd, J = 10.4, 8.7 Hz, 1H), 2.10 – 2.02 (m, 1H), 1.96 (ddd, J = 12.7, 8.9, 6.5 Hz, 1H), 1.73 (dddd, J = 19.0, 12.7, 9.4, 7.4 Hz, 1H), 1.65 – 1.48 (m, 1H). ^{13}C NMR (100 MHz, $CDCl_3$) δ 220.27, 159.67, 141.68, 129.42, 121.29, 114.64, 111.44, 55.17, 51.01, 38.23, 35.67, 29.24, 20.56. GC conditions: Agilgent CP-Chirasil Dex CB (df = 0.25 μm, 0.32 mm i.d. × 25 m); carrier gas, N_2 (flow 30 mL/min); injection temp, 180 °C; initial column temperature 120 °C, 5 °C/min to 150 °C, hold for 2 min, then 1 °C/min to 200 °C, hold for 5 min; t_R = 30.405 min and t_S = 30.275 min.

2-(4-methoxybenzyl)cyclopentan-1-one (1kp)

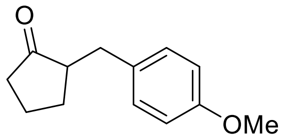

^{1}H NMR (400 MHz, $CDCl_3$) δ 7.08 (d, *J* = 8.6 Hz, 2H), 6.82 (d, *J* = 8.7 Hz, 2H), 3.78 (s, 3H), 3.06 (dd, *J* = 14.0, 4.2 Hz, 1H), 2.51 (dd, *J* = 14.0, 9.2 Hz, 1H), 2.39 – 2.25 (m, 2H), 2.15 – 2.03 (m, 2H), 1.95 (dtd, *J* = 12.3, 8.9, 7.6, 4.9 Hz, 1H), 1.76 – 1.51 (m, 2H). ^{13}C NMR (100 MHz, $CDCl_3$) δ 220.49, 158.04, 131.98, 129.88, 113.82, 55.26, 51.19, 38.33, 34.67, 29.06, 20.58. GC conditions: Agilgent CP-Chirasil Dex CB (df = 0.25 μm, 0.32 mm i.d. × 25 m); carrier gas, N_2 (flow 30 mL/min); injection temp, 180 °C; initial column temperature 120 °C, 5 °C/min to 150 °C, hold for 2 min, then 1 °C/min to 200 °C, hold for 5 min; t_R = 34.713 min and t_S = 34.616 min.

2-(4-ethylbenzyl)cyclopentan-1-one (1lp)

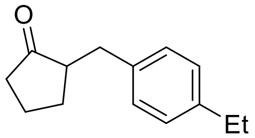

^{1}H NMR (400 MHz, $CDCl_3$) δ 7.21 – 7.04 (m, 4H), 3.14 (dd, *J* = 13.8, 4.1 Hz, 1H), 2.65 (q, *J* = 7.6 Hz, 2H), 2.54 (dd, *J* = 13.9, 9.5 Hz, 1H), 2.44 – 2.29 (m, 2H), 2.13 (ddd, *J* = 18.8, 10.3, 8.6 Hz, 2H), 2.04 – 1.91 (m, 1H), 1.75 (dd, *J* = 8.4, 2.1 Hz, 1H), 1.65 – 1.52 (m, 1H), 1.25 (t, *J* = 7.6 Hz, 3H). ^{13}C NMR (100 MHz, $CDCl_3$) δ 220.45, 142.08, 137.17, 128.86, 127.92, 51.13, 38.28, 35.20, 29.21, 28.45, 20.58, 15.62. GC conditions: Agilgent CP-Chirasil Dex CB (df = 0.25 μm, 0.32 mm i.d. × 25 m); carrier gas, N_2 (flow 30 mL/min); injection temp, 180 °C; initial column temperature 100 °C, then progress rate, 5 °C/min; final column temperature, 200 °C for 5 min; t_R = 20.112 min and t_S = 19.822 min.

2-(2-nitrobenzyl)cyclopentan-1-one (1mp)

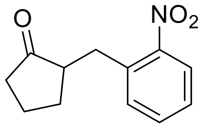

^{1}H NMR (400 MHz, $CDCl_3$) δ 7.91 (d, *J* = 8.2 Hz, 1H), 7.53 (t, *J* = 7.5 Hz, 1H), 7.37 (t, *J* = 7.9 Hz, 2H), 3.46 (dd, *J* = 13.8, 5.2 Hz, 1H), 2.83 (dd, *J* = 13.8, 8.6 Hz, 1H), 2.49 (dt, *J* = 14.0, 8.3 Hz, 1H), 2.36 (dd, *J* = 18.8, 8.5 Hz, 1H), 2.13 (dq, *J* = 18.4, 9.5, 8.8 Hz, 2H), 2.06 – 1.97 (m, 1H), 1.84 – 1.70 (m, 1H), 1.55 (qd, *J* = 11.6, 6.5 Hz, 1H). ^{13}C NMR (100 MHz, $CDCl_3$) δ 219.04, 149.52, 135.21, 132.93, 132.60, 127.45, 124.80, 50.23, 37.71, 32.32, 29.46, 20.45. GC conditions: Agilgent CP-Chirasil Dex CB (df = 0.25 μm, 0.32 mm i.d. × 25 m); carrier gas, N_2 (flow 30 mL/min); injection temp, 180 °C; initial column

temperature 120 °C, 2 °C/min to 130 °C , then 10 °C/min to 170 °C and 1 °C/min to 200 °C, hold for 5 min; t_R = 20.701 min and t_S = 20.535 min.

2-methylcyclopentan-1-one (1pp)

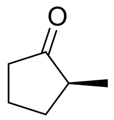

^{1}H NMR (400 MHz, $CDCl_3$) δ 1.07 – 1.16 (d, *J* = 7.0 Hz, 3H), 1.43 – 1.57 (dtd, *J* = 6.6, 10.7, 12.2 Hz, 1H), 1.72 – 1.89 (m, 1H), 1.89 – 2.04 (m, 1H), 2.04 – 2.14 (m, 1H), 2.14 – 2.21 (m, 1H), 2.21 – 2.30 (m, 1H), 2.30 – 2.46 (m, 1H). GC conditions: Agilgent CP-Chirasil Dex CB (df = 0.25 μm, 0.32 mm i.d. × 25 m); carrier gas, N_2 (flow 30 mL/min); injection temp, 180 °C; initial column temperature 100 °C, then progress rate, 5 °C/min; final column temperature, 200 °C for 5 min; t_R = 8.191 min and t_S = 7.864 min.

3-methylcyclopentan-1-one (1qp)

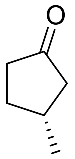

^{1}H NMR (400 MHz, $CDCl_3$) δ 1.06 – 1.21 (dd, *J* = 1.5, 6.6 Hz, 3H), 1.42 – 1.64 (m, 1H), 1.69 – 1.89 (ddd, *J* = 1.6, 9.3, 17.7 Hz, 1H), 2.06 – 2.23 (m, 2H), 2.23 – 2.48 (m, 3H). GC conditions: Agilgent CP-Chirasil Dex CB (df = 0.25 μm, 0.32 mm i.d. × 25 m); carrier gas, N_2 (flow 30 mL/min); injection temp, 180 °C; initial column temperature 100 °C, then progress rate, 5 °C/min; final column temperature, 200 °C for 5 min; t_R = 9.685 min and t_S = 9.518 min.

3-phenylcyclopentan-1-one (1rp)

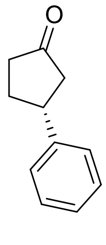

^{1}H NMR (400 MHz, $CDCl_3$) δ 7.34 (dd, *J* = 30.8, 7.4 Hz, 5H), 3.53 – 3.39 (m, 1H), 2.71 (dd, *J* = 18.3, 7.6 Hz, 1H), 2.51 (dd, *J* = 18.4, 8.7 Hz, 2H), 2.43 – 2.24 (m, 2H), 2.03 (p, *J* = 11.6, 11.2 Hz, 1H). ^{13}C NMR (100 MHz, $CDCl_3$) δ 218.46, 143.12, 128.75, 126.79, 45.86, 42.28, 38.94, 31.26. GC conditions: Agilgent CP-Chirasil Dex CB (df = 0.25 μm, 0.32 mm i.d. × 25 m); carrier gas, N_2 (flow 30 mL/min); injection temp, 180 °C; initial column temperature 100 °C, then progress rate, 5 °C/min; final column temperature, 200 °C for 5 min; t_R = 19.025 min and t_S = 18.058 min.

2-benzylcyclohexan-1-one (2ap)

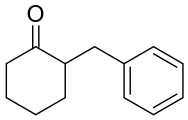

^{1}H NMR (400 MHz, $CDCl_3$) δ 7.30 (d, *J* = 6.9 Hz, 2H), 7.22 (dd, *J* = 15.9, 7.0 Hz, 3H), 3.28 (dd, *J* = 13.8, 3.7 Hz, 1H), 2.58 (d, *J* = 11.2 Hz, 1H), 2.48 (s, 1H), 2.47 – 2.34 (m, 2H), 2.08 (t, *J* = 11.9 Hz, 2H), 1.87 (d, *J* = 12.9 Hz, 1H), 1.69 (dt, *J* = 41.3, 12.3 Hz, 2H), 1.39 (q, *J* = 12.3 Hz, 1H). ^{13}C NMR (100 MHz, $CDCl_3$) δ 212.66, 140.44, 129.21, 128.36, 126.03, 52.55, 42.23, 35.53, 33.47, 28.12, 25.13. GC conditions: Agilgent CP-Chirasil Dex CB (df = 0.25 μm, 0.32 mm i.d. × 25 m); carrier gas, N_2 (flow 30 mL/min); injection temp, 180 °C; initial column temperature 100 °C, then progress rate, 5 °C/min; final column temperature, 200 °C for 5 min; t_R = 16.408 min and t_S = 15.676 min.

2-(2-methylbenzyl)cyclohexan-1-one (2bp)

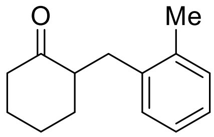

^{1}H NMR (400 MHz, $CDCl_3$) δ 7.14 (dt, *J* = 9.6, 4.3 Hz, 4H), 3.29 (dd, *J* = 14.1, 4.2 Hz, 1H), 2.56 (ddd, *J* = 12.1, 9.5, 5.0 Hz, 1H), 2.51 – 2.35 (m, 3H), 2.31 (s, 3H), 2.08 (tdd, *J* = 16.5, 5.6, 2.7 Hz, 2H), 1.94 – 1.81 (m, 1H), 1.76 – 1.55 (m, 2H), 1.42 (ddd, *J* = 24.9, 12.3, 3.5 Hz, 1H). ^{13}C NMR (101 MHz, $CDCl_3$) δ 212.62, 138.53, 136.27, 130.29, 129.91, 126.12, 125.75, 51.22, 42.22, 33.56, 32.46, 28.12, 25.20, 19.58. GC conditions: Agilgent CP-Chirasil Dex CB (df = 0.25 μm, 0.32 mm i.d. × 25 m); carrier gas, N_2 (flow 30 mL/min); injection temp, 180 °C; initial column temperature 100 °C, then progress rate, 5 °C/min; final column temperature, 200 °C for 5 min; t_R = 15.476 min and t_S = 14.815 min.

2-(4-methylbenzyl)cyclohexan-1-one (2dp)

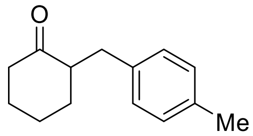

^{1}H NMR (400 MHz, $CDCl_3$) δ 7.06 (q, *J* = 8.1 Hz, 4H), 3.19 (dd, *J* = 13.9, 4.7 Hz, 1H), 2.57 – 2.45 (m, 1H), 2.43 – 2.33 (m, 2H), 2.31 (s, 3H), 2.11 – 1.96 (m, 2H), 1.88 – 1.76 (m, 1H), 1.74 – 1.51 (m, 2H), 1.42 – 1.23 (m, 1H). ^{13}C NMR (100 MHz, $CDCl_3$) δ 212.77, 137.26, 135.46, 129.05, 129.02, 52.61, 42.20, 35.03, 33.40, 28.10, 25.08, 21.04. GC conditions: Agilgent CP-Chirasil Dex CB (df = 0.25 μm, 0.32 mm i.d. × 25 m); carrier gas, N_2 (flow 30 mL/min); injection temp, 180 °C; initial column temperature 100 °C, then progress rate, 5 °C/min; final column temperature, 200 °C for 5 min; t_R = 17.822 min and t_S = 18.767 min.

2-(4-nitrobenzyl)cyclohexan-1-one (2fp)

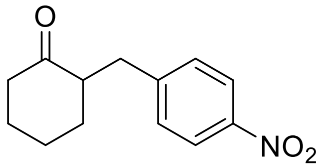

^1H NMR (400 MHz, $CDCl_3$) δ 8.18 – 8.09 (m, 2H), 7.35 (d, *J* = 8.7 Hz, 2H), 3.37 – 3.23 (m, 1H), 2.69 – 2.52 (m, 2H), 2.50 – 2.42 (m, 1H), 2.35 (td, *J* = 12.7, 6.0 Hz, 1H), 2.19 – 1.98 (m, 2H), 1.89 (ddd, *J* = 9.6, 3.3, 1.6 Hz, 1H), 1.71 – 1.60 (m, 2H), 1.51 – 1.33 (m, 1H). ^{13}C NMR (100 MHz, $CDCl_3$) δ 211.44, 148.56, 146.46, 130.03, 129.94, 123.59, 123.48, 52.10, 42.19, 35.49, 33.83, 27.94, 25.17. GC conditions: Agilgent CP-Chirasil Dex CB (df = 0.25 μm, 0.32 mm i.d. × 25 m); carrier gas, N_2 (flow 30 mL/min); injection temp, 180 °C; initial column temperature 120 °C, 8 °C/min to 150 °C, then 10 °C/min to 170 °C and 1 °C/min to 200 °C, hold for 5 min; t_R = 20.524 min and t_S = 20.409 min.

3-benzyltetrahydro-4H-pyran-4-one (2gp)

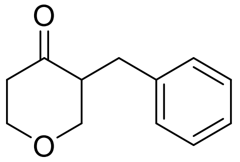

^1H NMR (400 MHz, $CDCl_3$) δ 7.28 (t, *J* = 7.6 Hz, 2H), 7.24 – 7.06 (m, 3H), 4.18 (d, *J* = 5.6 Hz, 1H), 4.04 (dd, *J* = 11.5, 6.0 Hz, 1H), 3.77 (t, *J* = 10.9 Hz, 1H), 3.43 (t, *J* = 10.5 Hz, 1H), 3.21 (dd, *J* = 14.4, 5.0 Hz, 1H), 2.84 (q, *J* = 7.5, 5.3 Hz, 1H), 2.63 (dd, *J* = 19.3, 12.3 Hz, 1H), 2.48 (dd, *J* = 13.6, 7.5 Hz, 2H). ^{13}C NMR (100 MHz, $CDCl_3$) δ 207.77, 138.88, 128.89, 128.57, 126.41, 72.18, 68.74, 53.04, 42.51, 31.83. GC conditions: Agilgent CP-Chirasil Dex CB (df = 0.25 μm, 0.32 mm i.d. × 25 m); carrier gas, N_2 (flow 30 mL/min); injection temp, 180 °C; initial column temperature 120 °C, 2 °C/min to 130 °C, then 10 °C/min to 170 °C and 1 °C/min to 200 °C, hold for 5 min; t_R = 20.718 min and t_S = 20.636 min.

2-methylcyclohexan-1-one (2jp)

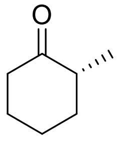

^1H NMR (400 MHz, $CDCl_3$) δ 2.61 – 2.22 (m, 3H), 2.21 – 2.01 (m, 2H), 1.95 – 1.76 (m, 1H), 1.78 – 1.54 (m, 2H), 1.52 – 1.31 (m, 1H), 1.04 (d, *J* = 6.6 Hz, 3H). ^{13}C NMR (100 MHz, $CDCl_3$) δ 213.41, 45.24, 41.74, 36.11, 27.88, 25.09, 14.62. GC conditions: Agilgent CP-Chirasil Dex CB (df = 0.25 μm, 0.32 mm i.d. × 25 m); carrier gas, N_2 (flow 30 mL/min); injection temp, 180 °C; initial column temperature 100 °C, then progress rate, 5 °C/min; final column temperature, 200 °C for 5 min; t_R = 13.989 min and t_S = 13.445 min.

3-methylcyclohexan-1-one (2kp)

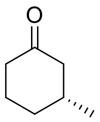

^{1}H NMR (400 MHz, $CDCl_3$) δ 2.54 – 2.31 (m, 2H), 2.31 – 2.14 (m, 1H), 2.12 – 1.98 (m, 2H), 1.98 – 1.79 (m, 2H), 1.78 – 1.56 (m, 1H), 1.35 (tdd, *J* = 13.4, 10.5, 3.7 Hz, 1H), 1.04 (d, *J* = 6.3 Hz, 3H). ^{13}C NMR (100 MHz, $CDCl_3$) δ 211.69, 49.88, 41.01, 34.11, 33.19, 25.21, 21.97. GC conditions: Agilgent CP-Chirasil Dex CB (df = 0.25 μm, 0.32 mm i.d. × 25 m); carrier gas, N_2 (flow 30 mL/min); injection temp, 180 °C; initial column temperature 100 °C, then progress rate, 5 °C/min; final column temperature, 200 °C for 5 min; t_R = 11.857 min and t_S = 11.615 min.

3-phenylcyclohexan-1-one (2lp)

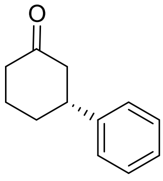

^{1}H NMR (400 MHz, $CDCl_3$) δ 7.31 (dd, *J* = 39.8, 7.2 Hz, 5H), 3.05 (t, *J* = 11.8 Hz, 1H), 2.61 (dd, *J* = 22.5, 11.4 Hz, 2H), 2.53 – 2.34 (m, 2H), 2.15 (dd, *J* = 24.7, 11.7 Hz, 2H), 1.87 (dq, *J* = 22.2, 12.5 Hz, 2H). ^{13}C NMR (100 MHz, $CDCl_3$) δ 211.09, 144.42, 128.76, 126.71 (d, *J* = 11.4 Hz), 49.01, 44.81, 41.26, 32.84, 25.61. GC conditions: Agilgent CP-Chirasil Dex CB (df = 0.25 μm, 0.32 mm i.d. × 25 m); carrier gas, N_2 (flow 30 mL/min); injection temp, 180 °C; initial column temperature 100 °C, then progress rate, 5 °C/min; final column temperature, 200 °C for 5 min; t_R = 23.009 min and t_S = 21.959 min.

2-(2-methylbenzyl)cycloheptan-1-one (3ap)

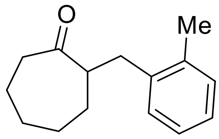

^{1}H NMR (400 MHz, $CDCl_3$) δ 7.11 (tt, *J* = 8.7, 3.9 Hz, 4H), 3.08 (dd, *J* = 14.1, 5.5 Hz, 1H), 2.80 (d, *J* = 9.1 Hz, 1H), 2.59 – 2.44 (m, 3H), 2.31 (s, 3H), 1.92 – 1.74 (m, 4H), 1.65 (s, 1H), 1.35 (q, *J* = 9.7, 8.2 Hz, 3H). ^{13}C NMR (100 MHz, $CDCl_3$) δ 215.67, 138.22, 136.43, 130.36, 129.71, 126.22, 125.79, 52.34, 43.05, 34.80, 30.35, 29.38, 28.64, 24.39, 19.63. GC conditions: Agilgent CP-Chirasil Dex CB (df = 0.25 μm, 0.32 mm i.d. × 25 m); carrier gas, N_2 (flow 30 mL/min); injection temp, 180 °C; initial column temperature 120 °C, 8 °C/min to 150 °C, then 10 °C/min to 170 °C and 1 °C/min to 200 °C, hold for 5 min; t_R = 27.847 min and t_S = 27.749 min.

2-(3-methoxybenzyl)cycloheptan-1-one (3bp)

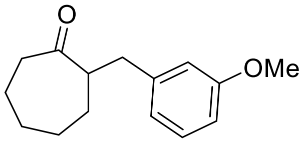

^{1}H NMR (400 MHz, $CDCl_3$) δ 7.21 (t, *J* = 7.8 Hz, 1H), 6.87 – 6.68 (m, 3H), 3.82 (s, 3H), 3.08 (dd, *J* = 13.7, 5.7 Hz, 1H), 2.85 (s, 1H), 2.61 – 2.46 (m, 3H), 1.97 – 1.78 (m, 4H), 1.68 (s, 1H), 1.46 – 1.31 (m, 3H). ^{13}C NMR (100 MHz, $CDCl_3$) δ 215.67, 159.57, 141.66, 129.28, 121.57, 114.93, 111.32, 55.16, 53.51, 43.24, 37.91, 30.35, 29.33, 28.68, 24.27. GC conditions: Agilgent CP-Chirasil Dex CB (df = 0.25 μm, 0.32 mm i.d. × 25 m); carrier gas, N_2 (flow 30 mL/min); injection temp, 180 °C; initial column temperature 120 °C, 8 °C/min to 150 °C, then 10 °C/min to 170 °C and 1 °C/min to 200 °C, hold for 5 min; t_R = 24.754 min and t_S = 24.661 min.

2-(2-fluorobenzyl)cycloheptan-1-one (3cp)

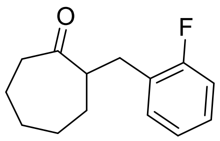

^{1}H NMR (400 MHz, $CDCl_3$) δ 7.24 (t, *J* = 7.1 Hz, 2H), 7.20 – 6.99 (m, 2H), 3.12 (dd, *J* = 13.8, 6.1 Hz, 1H), 2.95 (s, 1H), 2.73 (dd, *J* = 13.8, 8.2 Hz, 1H), 2.55 (t, *J* = 4.7 Hz, 2H), 1.90 (dd, *J* = 19.4, 8.6 Hz, 4H), 1.73 (dd, *J* = 15.9, 8.1 Hz, 1H), 1.43 (td, *J* = 11.5, 10.9, 6.2 Hz, 3H). ^{13}C NMR (100 MHz, $CDCl_3$) δ 215.14, 161.34 (d, *J* = 244 Hz), 131.68 (d, *J* = 5.1 Hz), 127.88 (d, *J* = 8.0 Hz), 126.92 (d, *J* = 15.8 Hz), 123.90 (d, *J* = 3.4 Hz), 115.22 (d, *J* = 22 Hz) 52.21, 43.09, 31.10, 30.46, 29.24, 28.62, 24.16. GC conditions: Agilgent CP-Chirasil Dex CB (df = 0.25 μm, 0.32 mm i.d. × 25 m); carrier gas, N_2 (flow 30 mL/min); injection temp, 180 °C; initial column temperature 120 °C, 2 °C/min to 130 °C, then 10 °C/min to 170 °C and 1 °C/min to 200 °C, hold for 5 min; t_R = 25.000 min and t_S = 24.906 min.

2-(4-chlorobenzyl)cycloheptan-1-one (3dp)

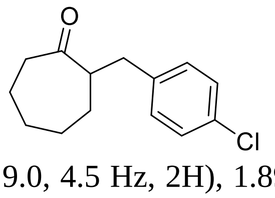

^{1}H NMR (400 MHz, $CDCl_3$) δ 7.28 – 7.21 (m, 2H), 7.10 (d, *J* = 8.4 Hz, 2H), 3.06 (dd, *J* = 13.8, 6.2 Hz, 1H), 2.80 (ddt, *J* = 10.1, 6.2, 3.3 Hz, 1H), 2.55 (dd, *J* = 13.8, 8.0 Hz, 1H), 2.46 (dd, *J* = 9.0, 4.5 Hz, 2H), 1.89 – 1.76 (m, 4H), 1.68 – 1.60 (m, 1H), 1.39 – 1.27 (m, 3H). ^{13}C NMR (100 MHz, $CDCl_3$) δ 215.21, 138.54, 131.85, 130.51, 128.44, 53.49, 43.32, 37.20, 30.50, 29.21, 28.73, 24.12. GC conditions: Agilgent CP-Chirasil Dex CB (df = 0.25 μm, 0.32 mm i.d. × 25 m); carrier gas, N_2 (flow 30 mL/min); injection temp, 180 °C; initial column temperature 120 °C, 8 °C/min to 150 °C, then 5 °C/min to 170 °C and 1 °C/min to 200 °C, hold for 5 min; t_R = 22.058 min and t_S = 22.449 min.

2-(3-bromobenzyl)cycloheptan-1-one (3ep)

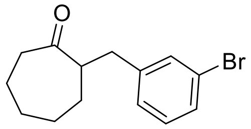

^{1}H NMR (400 MHz, $CDCl_3$) δ 7.32 (d, *J* = 6.7 Hz, 2H), 7.11 (dt, *J* = 16.6, 7.8 Hz, 2H), 3.05 (dd, *J* = 13.8, 6.1 Hz, 1H), 2.91 – 2.74 (m, 1H), 2.65 – 2.32 (m, 3H), 1.91 – 1.70 (m, 4H), 1.64 (ddd, *J* = 23.2, 9.9, 4.4 Hz, 1H), 1.46 – 1.25 (m, 3H). ^{13}C NMR (100 MHz, $CDCl_3$) δ 214.99, 142.53, 132.10, 129.90, 129.24, 127.87, 122.40, 53.31, 43.29, 37.43, 30.49, 29.18, 28.72, 24.08. GC conditions: Agilgent CP-Chirasil Dex CB (df = 0.25 μm, 0.32 mm i.d. × 25 m); carrier gas, N_2 (flow 30 mL/min); injection temp, 180 °C; initial column temperature 120 °C, 8 °C/min to 150 °C, then 5 °C/min to 170 °C and 1 °C/min to 200 °C, hold for 5 min; t_R =21.544 min and t_S = 22.029 min.

2-(4-(trifluoromethyl)benzyl)cycloheptan-1-one (3fp)

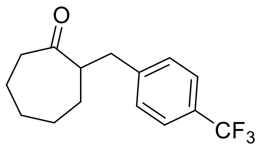

^{1}H NMR (400 MHz, $CDCl_3$) δ 7.52 (d, *J* = 8.0 Hz, 2H), 7.27 (d, *J* = 8.1 Hz, 2H), 3.14 (dd, *J* = 13.8, 6.3 Hz, 1H), 2.94 – 2.78 (m, 1H), 2.62 (dd, *J* = 13.8, 7.9 Hz, 1H), 2.53 – 2.40 (m, 2H), 1.81 (ddt, *J* = 29.5, 12.2, 5.4 Hz, 4H), 1.64 (tt, *J* = 14.7, 7.4 Hz, 1H), 1.49 – 1.27 (m, 3H). ^{13}C NMR (100 MHz, $CDCl_3$) δ 214.85, 144.33, 129.44, 125.23 (q, *J* = 3.8 Hz), 122.95, 53.22, 43.30, 37.61, 30.62, 29.13, 28.73, 24.02. GC conditions: Agilgent CP-Chirasil Dex CB (df = 0.25 μm, 0.32 mm i.d. × 25 m); carrier gas, N_2 (flow 30 mL/min); injection temp, 180 °C; initial column temperature 120 °C, 8 °C/min to 150 °C, then 10 °C/min to 170 °C and 1 °C/min to 200 °C, hold for 5 min; t_R = 25.485 min and t_S = 25.410 min.

(-)-Menthone

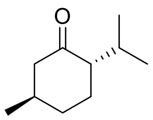

^{1}H NMR (400 MHz, $CDCl_3$) δ 2.37 (ddd, *J* = 12.9, 3.9, 2.3 Hz, 1H), 2.19 – 1.83 (m, 6H), 1.48 – 1.30 (m, 2H), 1.03 (d, *J* = 6.3 Hz, 3H), 0.93 (d, *J* = 6.9 Hz, 3H), 0.87 (d, *J* = 6.8 Hz, 3H). ^{13}C NMR (100 MHz, $CDCl_3$) δ 212.20, 55.79, 50.80, 35.41, 33.87, 27.82, 25.84, 22.22, 21.14, 18.64. GC conditions: Agilgent CP-Chirasil Dex CB (df = 0.25 μm, 0.32 mm i.d. × 25 m); carrier gas, N_2 (flow 30 mL/min); injection temp, 180 °C; initial column temperature 100 °C, then progress rate, 5 °C/min; final column temperature, 200 °C for 5 min; t_R = 5.267 min and t_S = 5.665 min.

Supplementary Figures

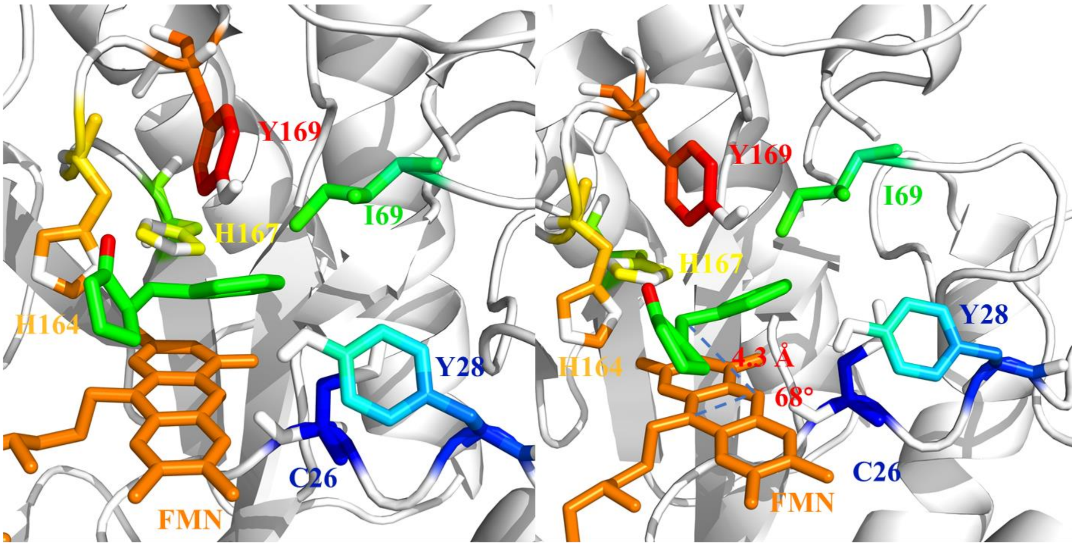

Supplementary Figure 1. Docking results of wt-YqjM with **1a**.

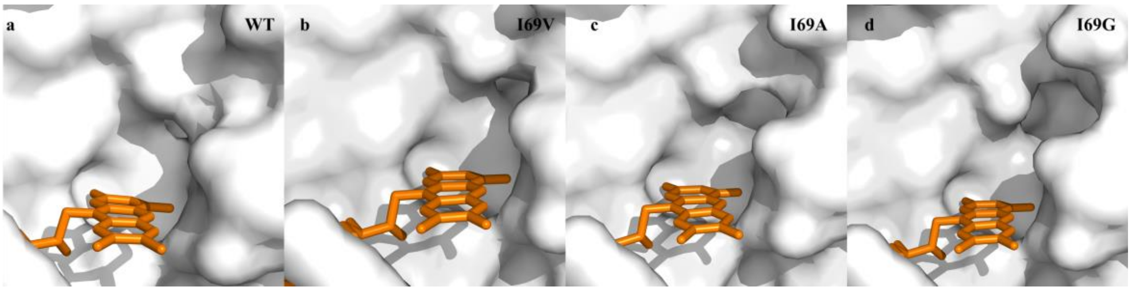

Supplementary Figure 2. Models of the active pocket. **a** wt-YqjM. **b** YqjM (I69V). **c** YqjM (I69A). **d** YqjM (I69G).

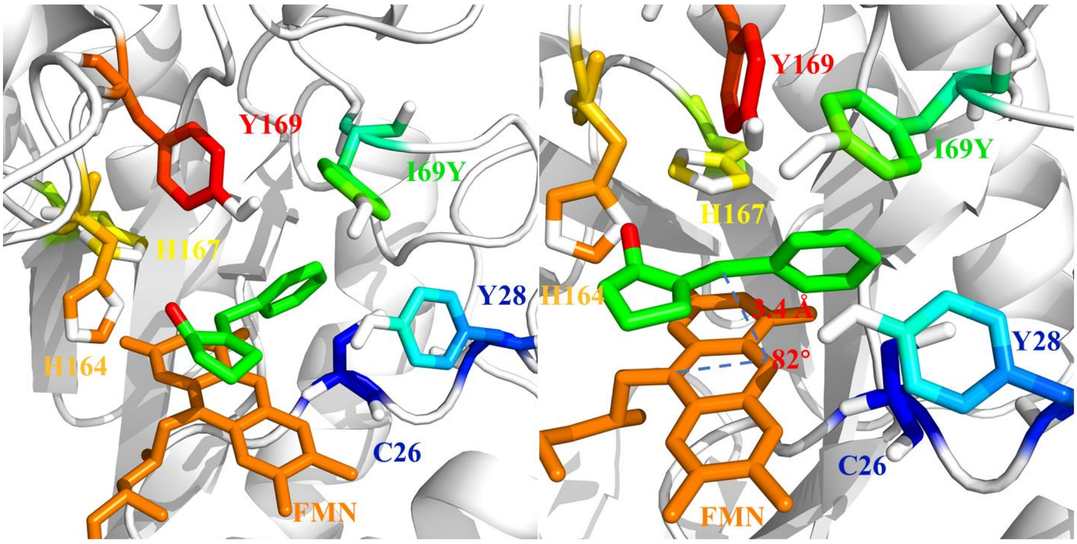

Supplementary Figure 3. Docking results of YqjM (I69Y) with **1a**.

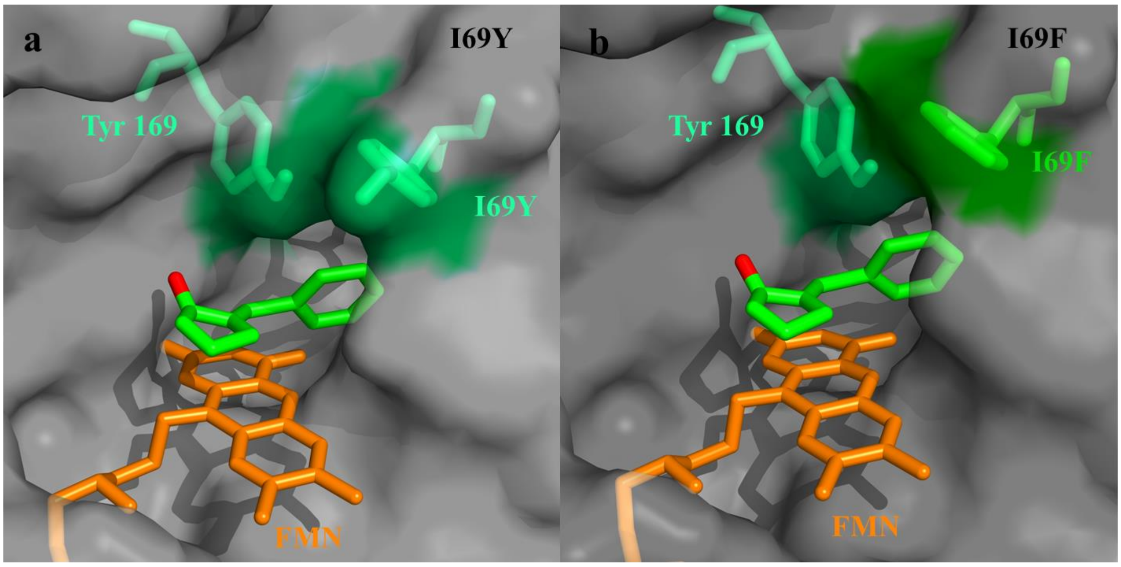

Supplementary Figure 4. Docking results of YqjM mutants with **1a**. **a** YqjM (I69F). **b** YqjM (I69Y).

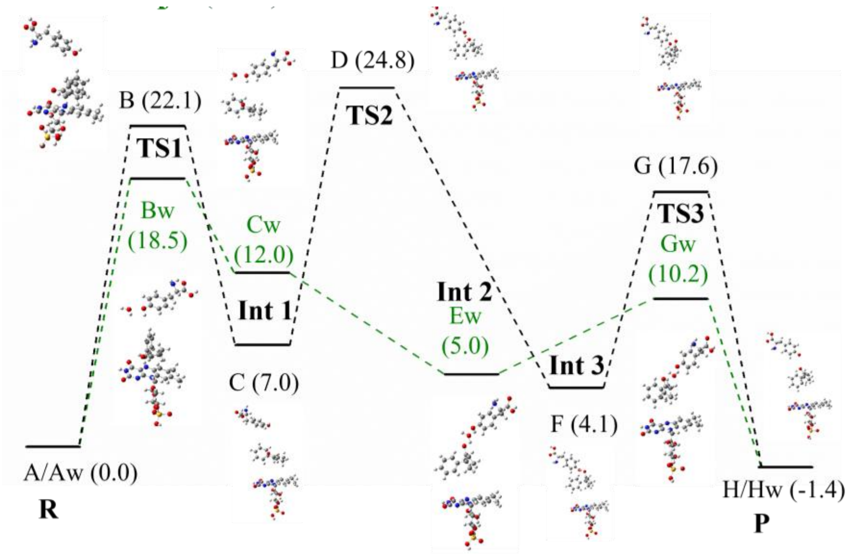

Supplementary Figure 5. Overall reaction scheme for reduction of **1a** by wt-YqjM (Black) and YqjM (I69Y/Y169F) (Green). Free energy profile for the reduction of **1a** by YqjM at the B3LYP+D3/6-311++G(2d,p) (SMD, solvent = water)//B3LYP+D3/6-31G(d) (SMD, solvent = water) level of theory. The values shown are free energies and energies (in brackets) in kcal/mol.

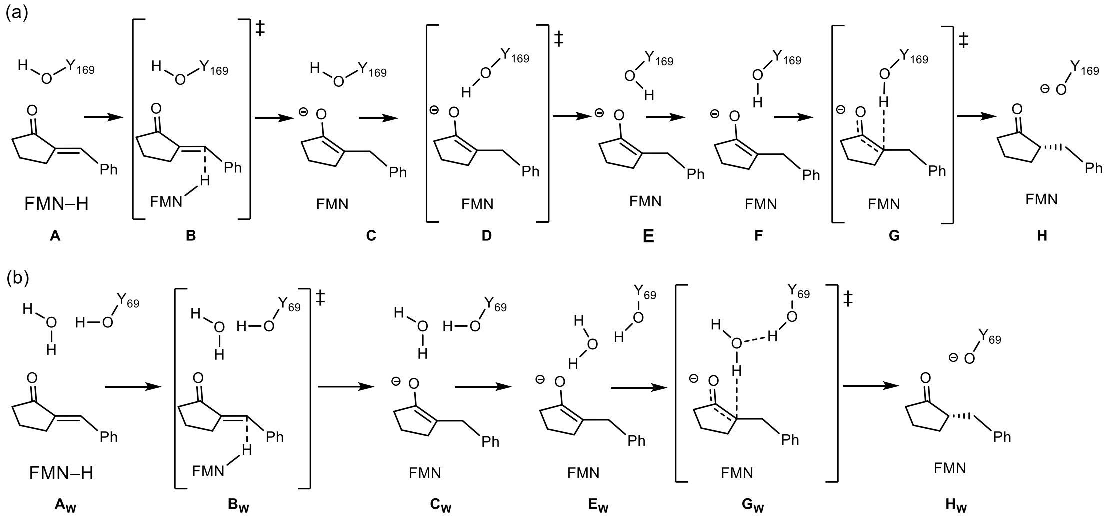

Supplementary Figure 6. Reduction mechanism of YqjM catalysis. Tyrosine protonates the substrate via a bridging water molecule (a) or directly (b)**.**

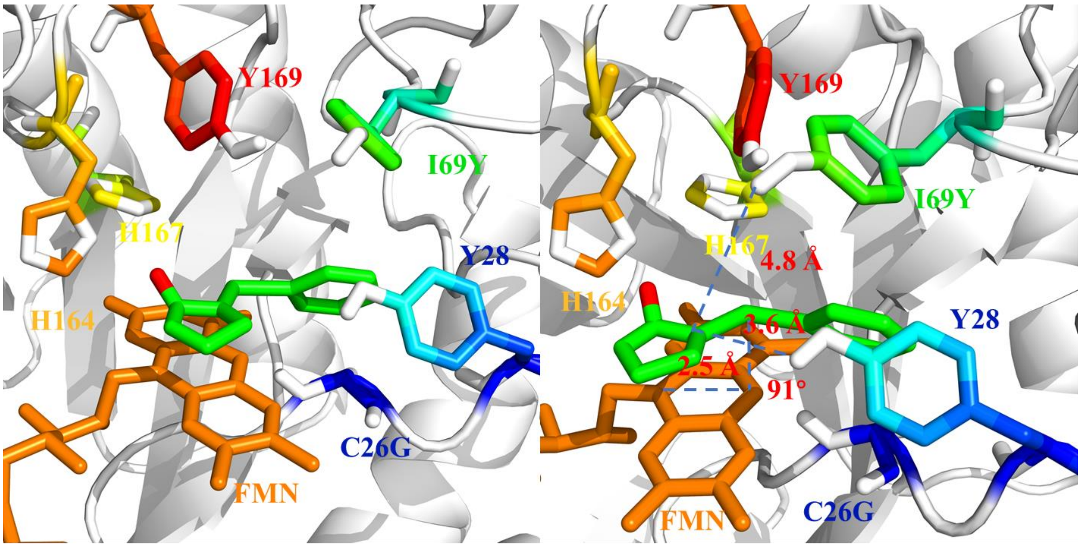

Supplementary Figure 7. Docking results of YqjM (C26G/I69Y) with **1a**.

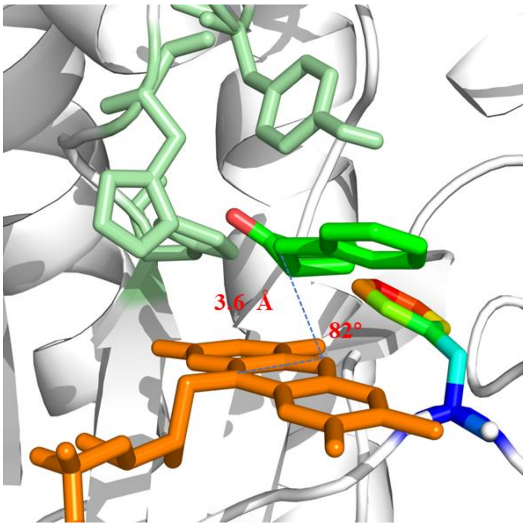

Supplementary Figure 8. Docking result of YqjM (C26F) with **1a**.

Kinetics parameters

For Supplementary Figures 9-22, *n* = 3. Error bars represent the standard deviations from three independent experiments; data are expressed as the mean ± SEM. Source data are provided as a Source data file.

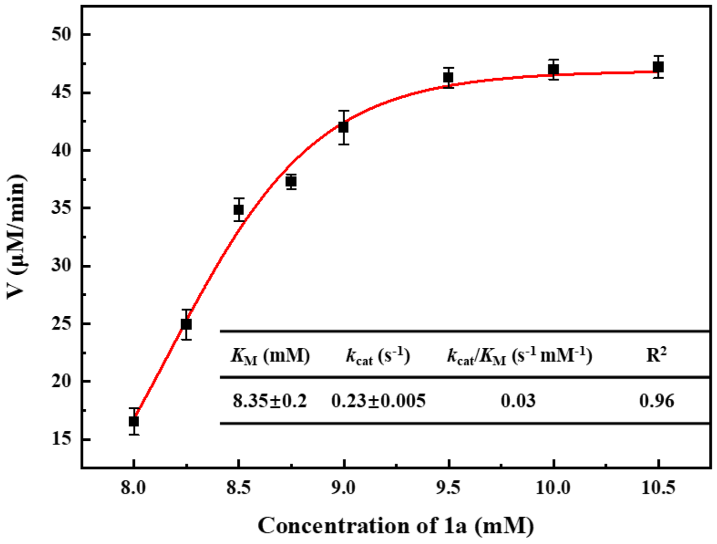

Supplementary Figure 9. Michaelis-Menten-plot for the reduction of **1a** by wt-YqjM.

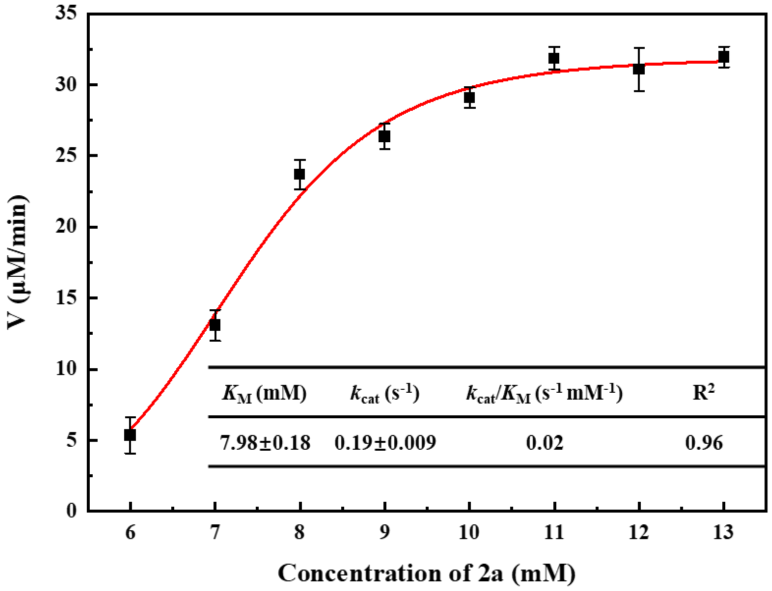

Supplementary Figure 10. Michaelis-Menten-plot for the reduction of **2a** by wt-YqjM.

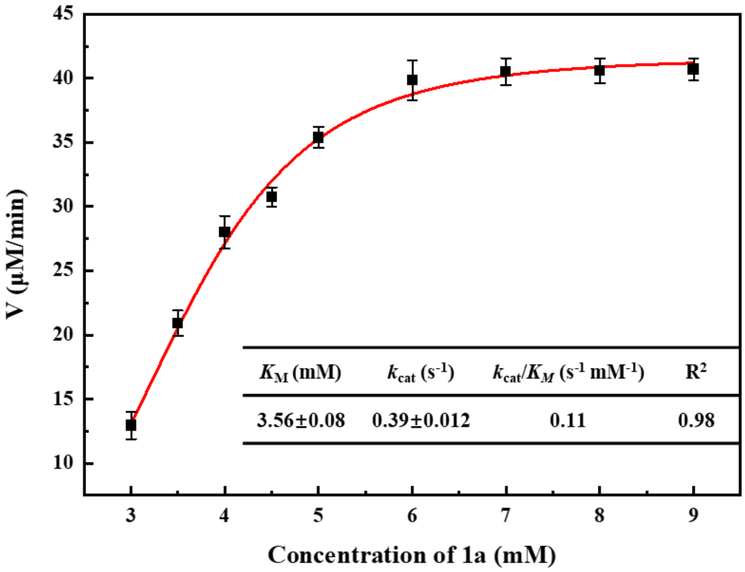

Supplementary Figure 11. Michaelis-Menten-plot for the reduction of **1a** by YqjM (I69G).

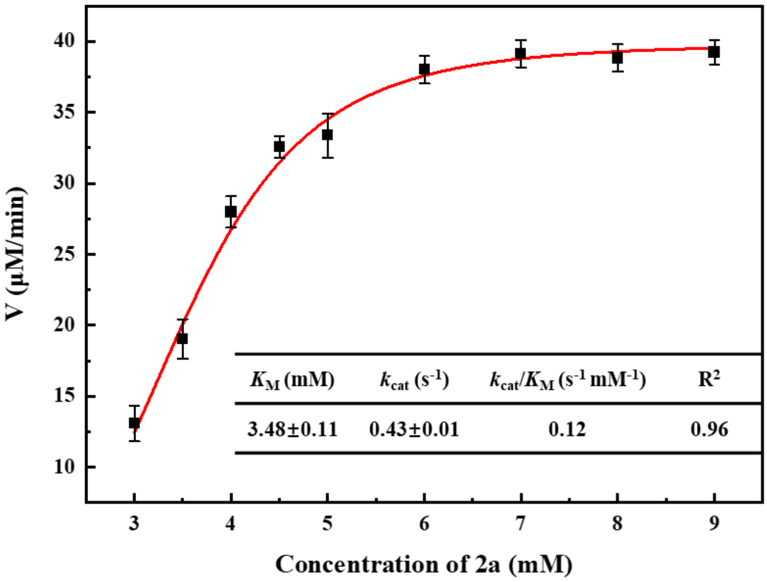

Supplementary Figure 12. Michaelis-Menten-plot for the reduction of **2a** by YqjM (I69G).

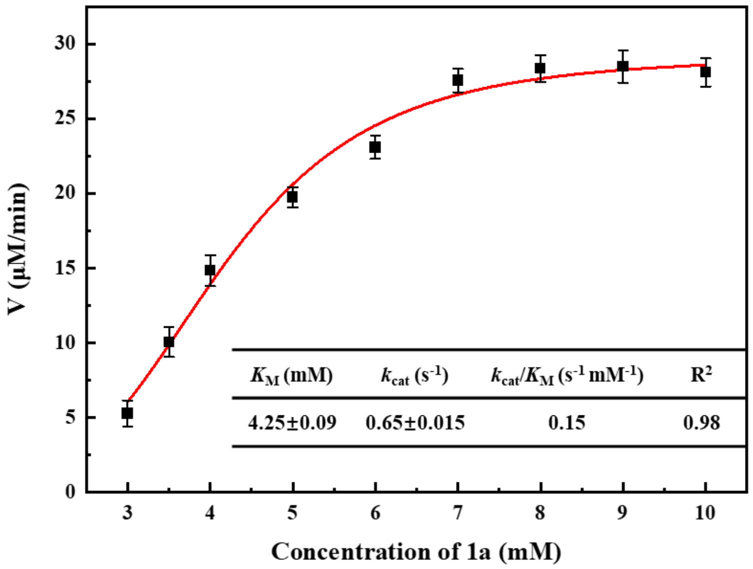

Supplementary Figure 13. Michaelis-Menten-plot for the reduction of **1a** by YqjM (I69Y).

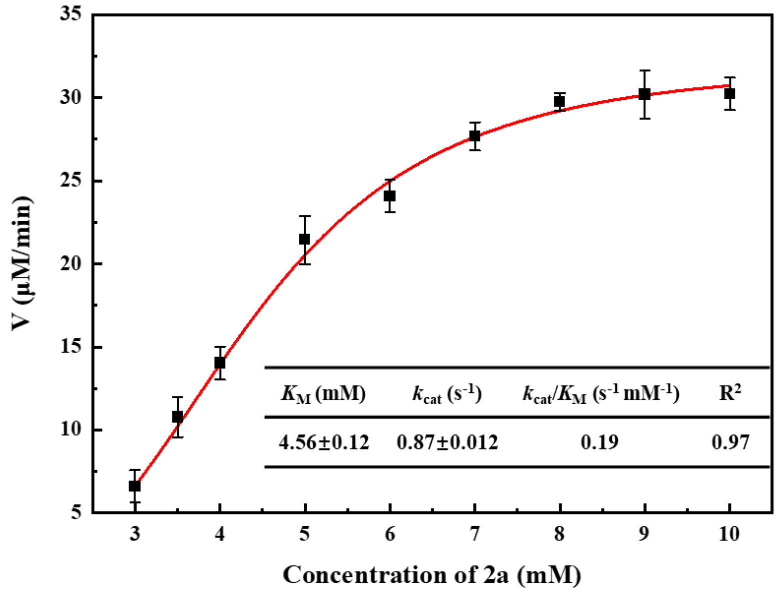

Supplementary Figure 14. Michaelis-Menten-plot for the reduction of **2a** by YqjM (I69Y).

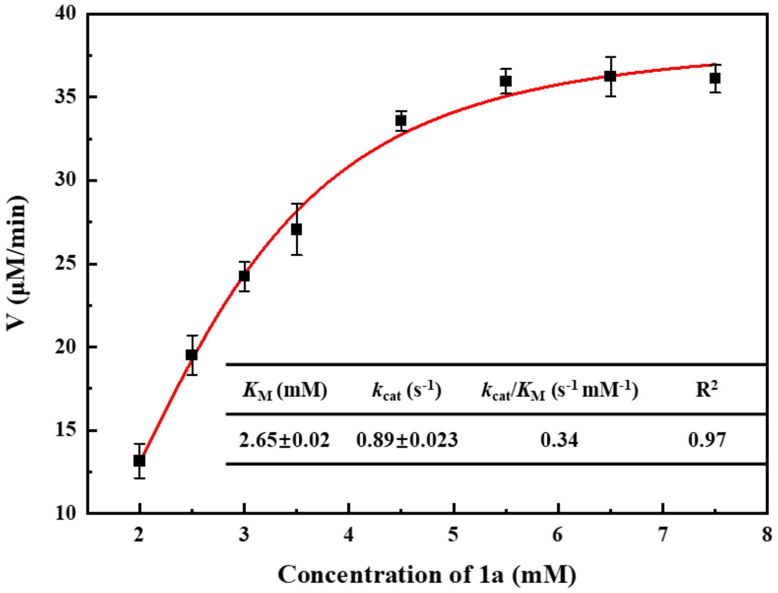

Supplementary Figure 15. Michaelis-Menten-plot for the reduction of **1a** by YqjM (I69Y/C26A).

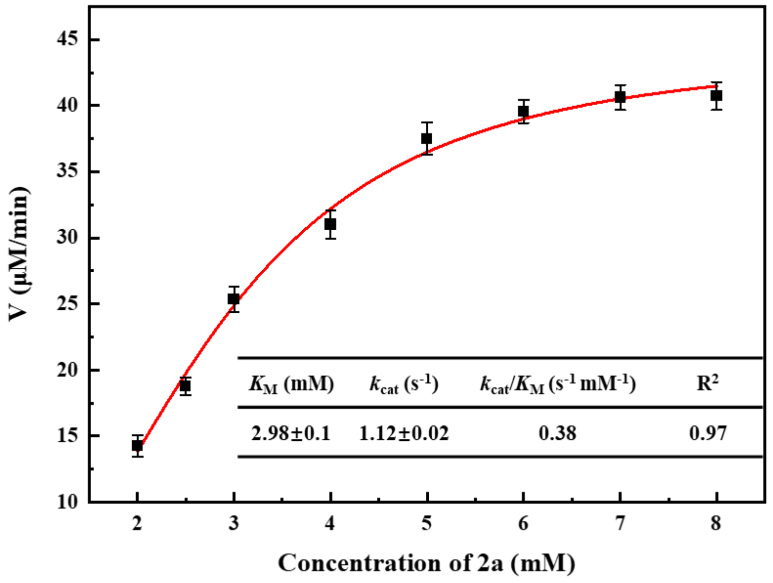

Supplementary Figure 16. Michaelis-Menten-plot for the reduction of **2a** by YqjM (I69Y/C26A).

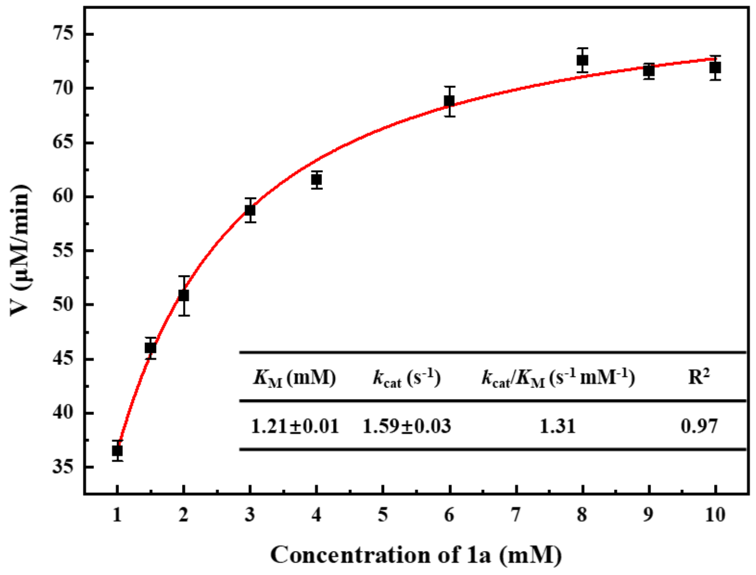

K_M (mM)	k_{cat} (s^{-1})	k_{cat}/K_M (s^{-1} mM^{-1})	R^2
1.21±0.01	1.59±0.03	1.31	0.97

Supplementary Figure 17. Michaelis-Menten-plot for the reduction of **1a** by YqjM (I69Y/C26G).

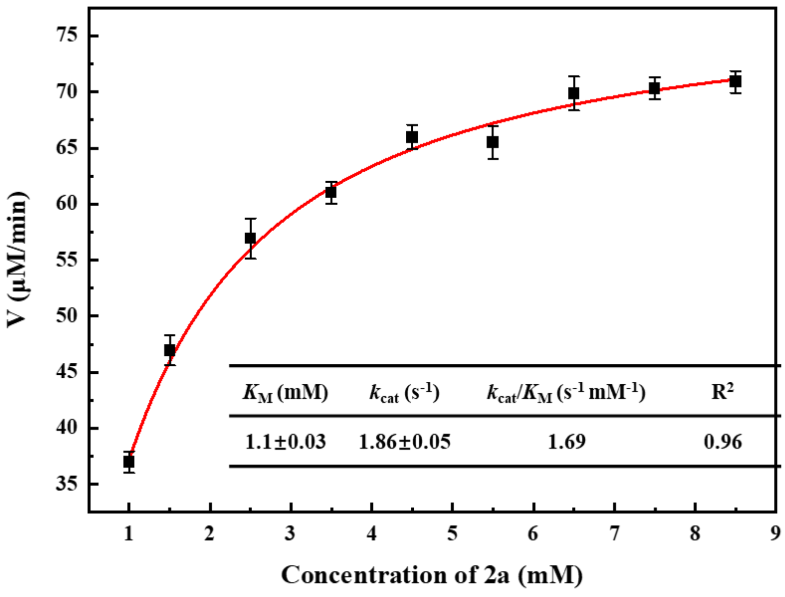

K_M (mM)	k_{cat} (s^{-1})	k_{cat}/K_M (s^{-1} mM^{-1})	R^2
1.1±0.03	1.86±0.05	1.69	0.96

Supplementary Figure 18. Michaelis-Menten-plot for the reduction of **2a** by YqjM (I69Y/C26G).

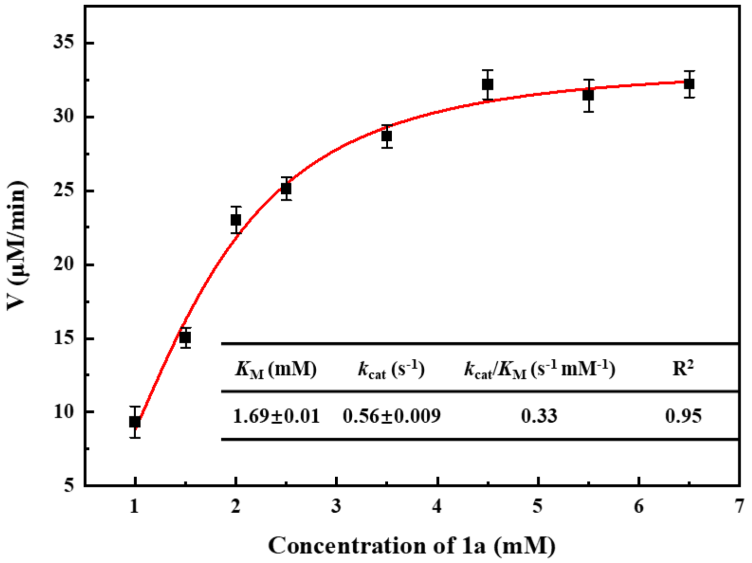

Supplementary Figure 19. Michaelis-Menten-plot for the reduction of **1a** by YqjM (I69Y/C26G/Y169F).

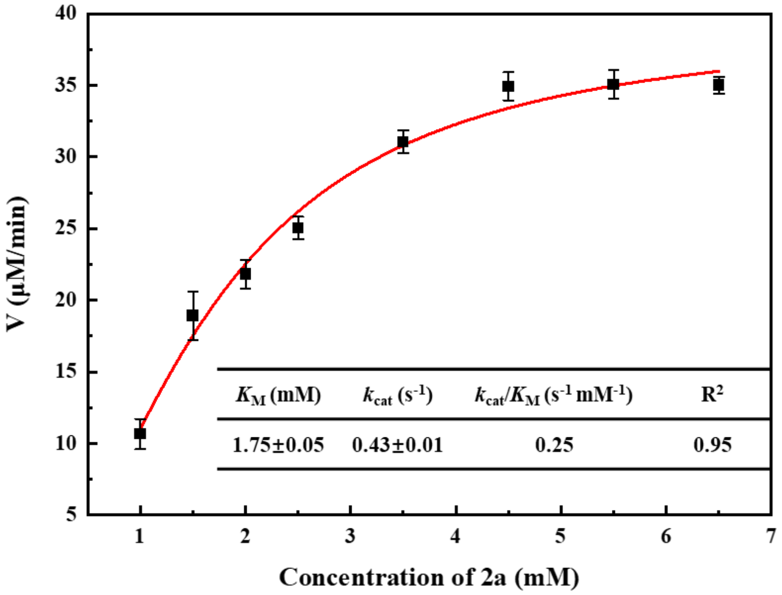

Supplementary Figure 20. Michaelis-Menten-plot for the reduction of **2a** by YqjM (I69Y/C26G/Y169F).

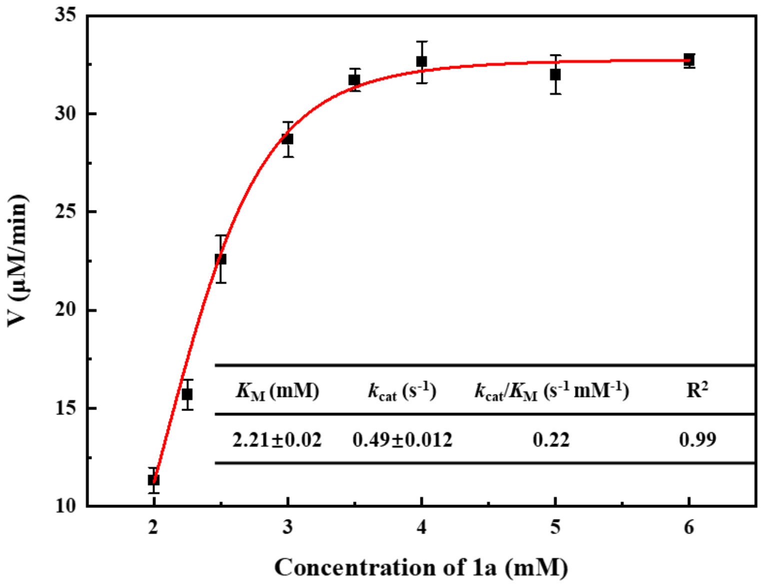

Supplementary Figure 21. Michaelis-Menten-plot for the reduction of **1a** by YqjM (I69Y/C26G/Y28F).

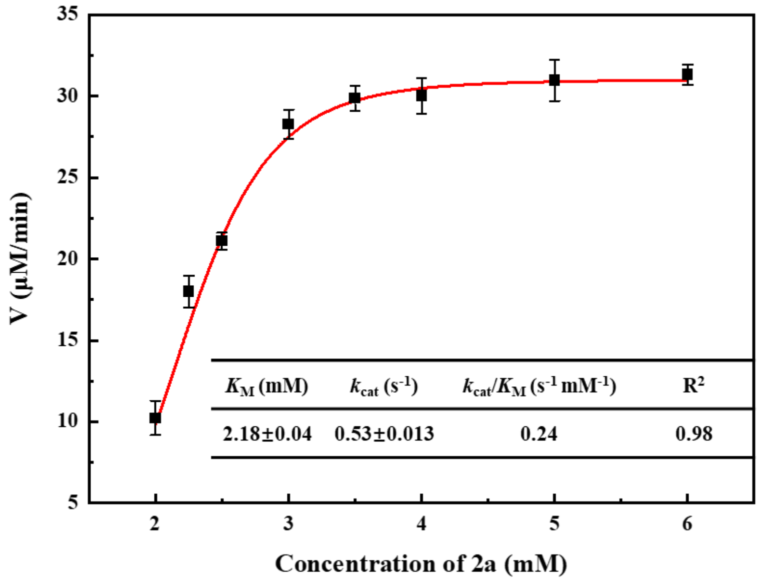

Supplementary Figure 22. Michaelis-Menten-plot for the reduction of **2a** by YqjM (I69Y/C26G/Y28F).

Enzymatic properties

For Supplementary Figures 23-33, n = 3. Error bars represent the standard deviations from three independent experiments; data are expressed as the mean ± SEM. Source data are provided as a Source data file.

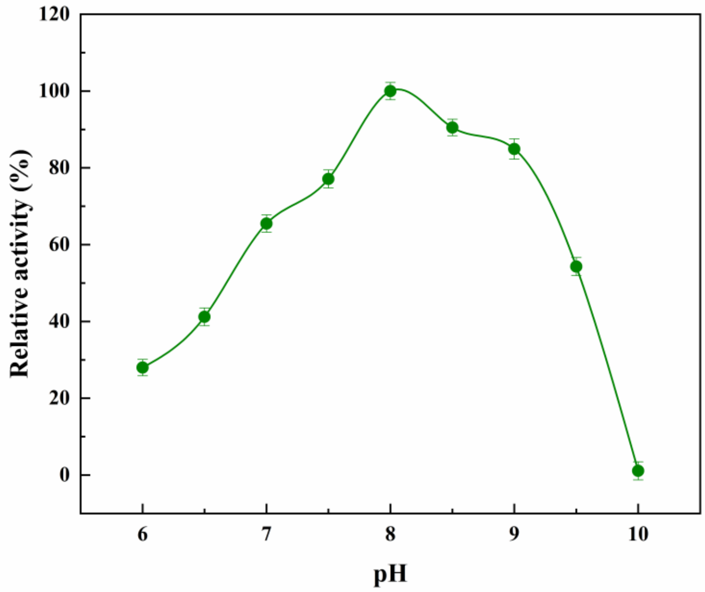

Supplementary Figure 23. Effect of pH on activity of YqjM (I69Y/C26G).

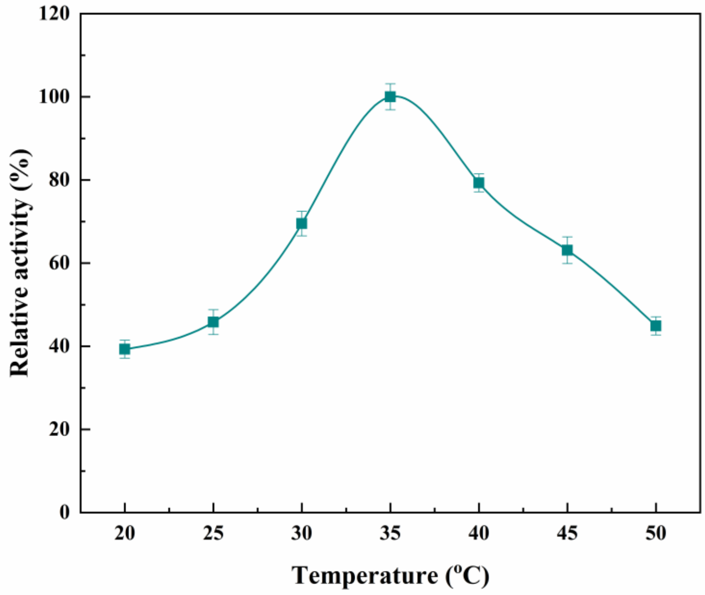

Supplementary Figure 24. Effect of temperature on activity of YqjM (I69Y/C26G).

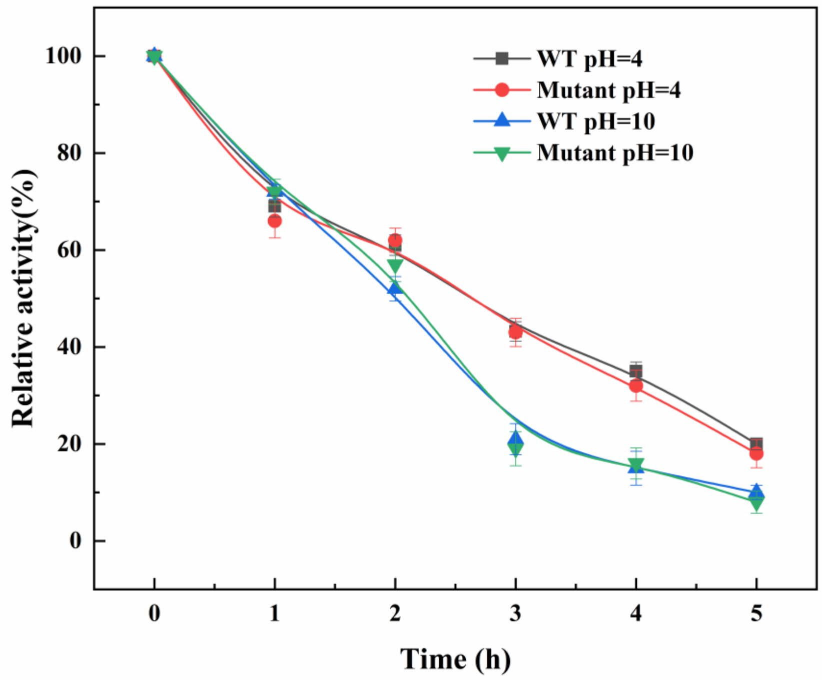

Supplementary Figure 25. pH stability of YqjM (I69Y/C26G).

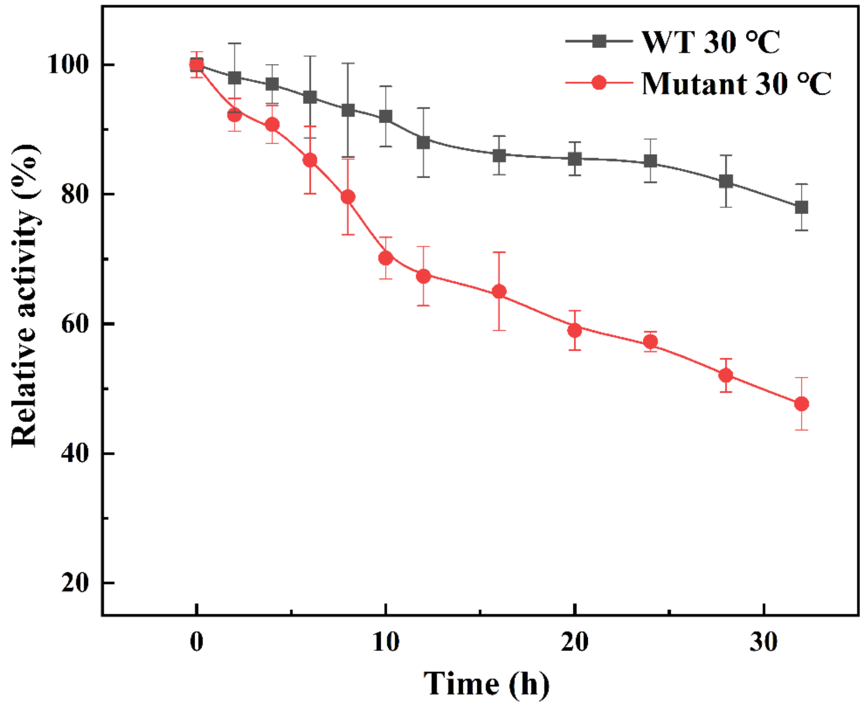

Supplementary Figure 26. Thermal stability of YqjM (I69Y/C26G).

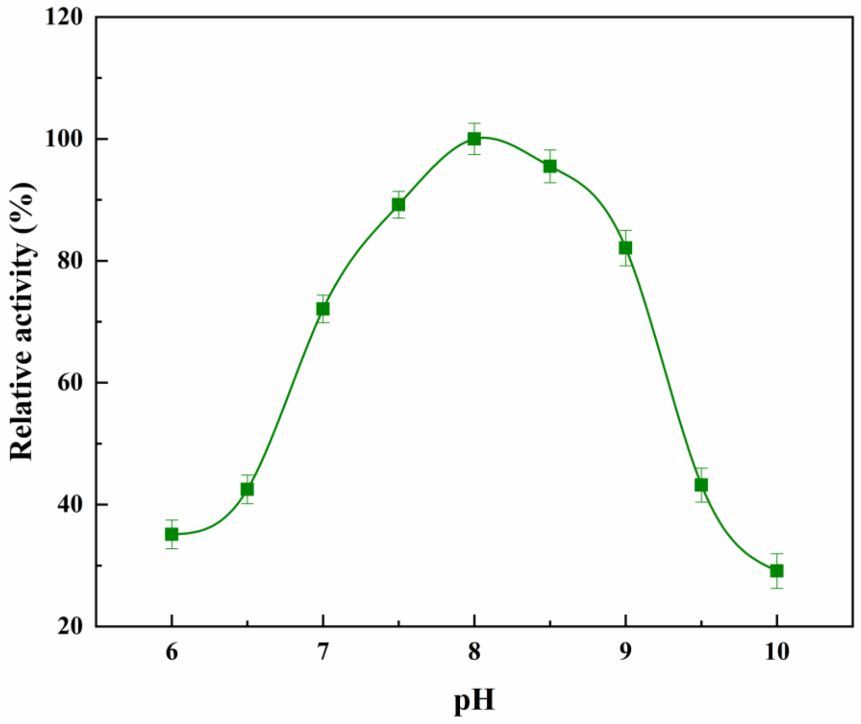

Supplementary Figure 27. Effect of pH on activity of YqjM (C26F).

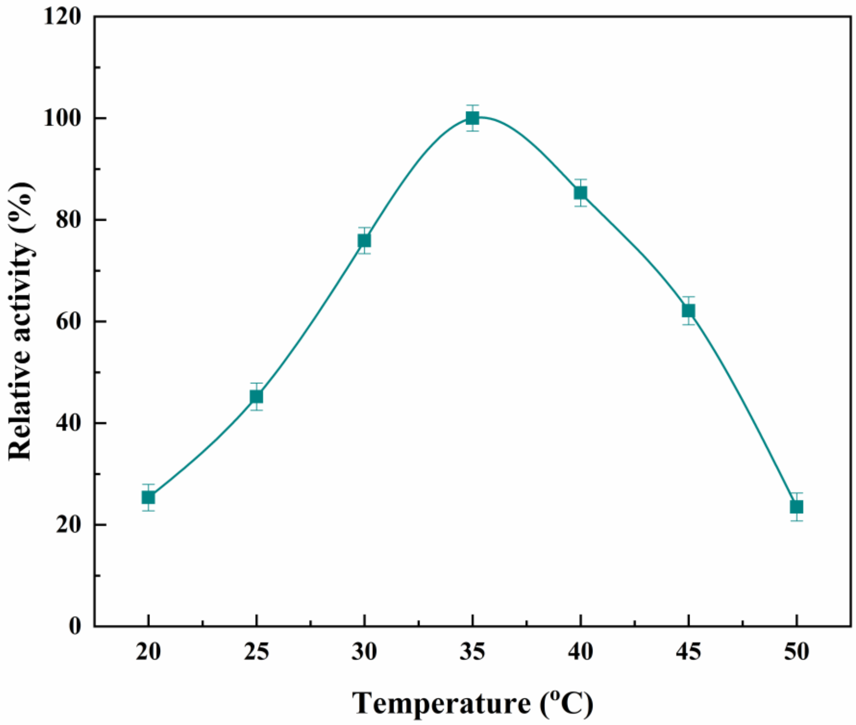

Supplementary Figure 28. Effect of temperature on activity of YqjM (C26F).

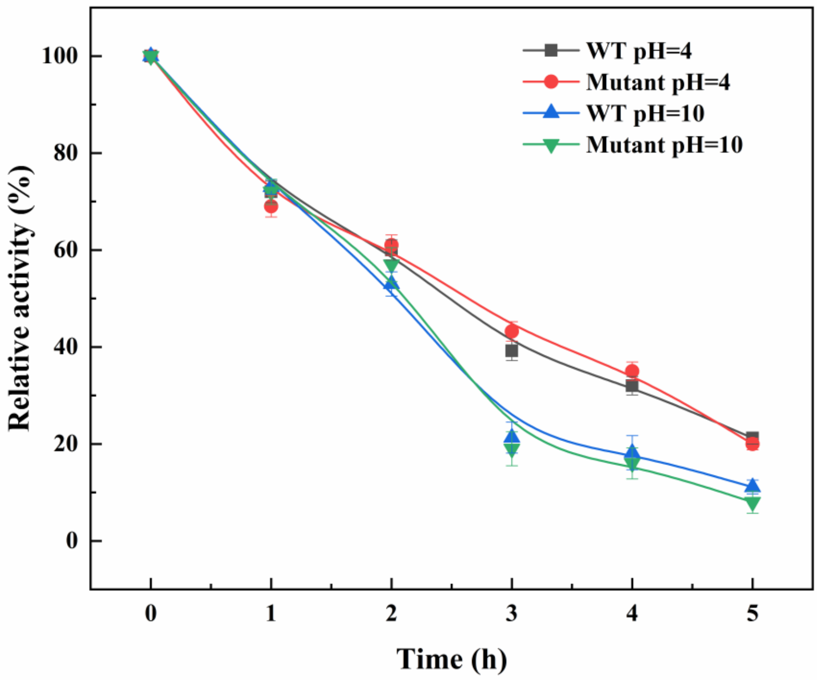

Supplementary Figure 29. pH stability of YqjM (C26F).

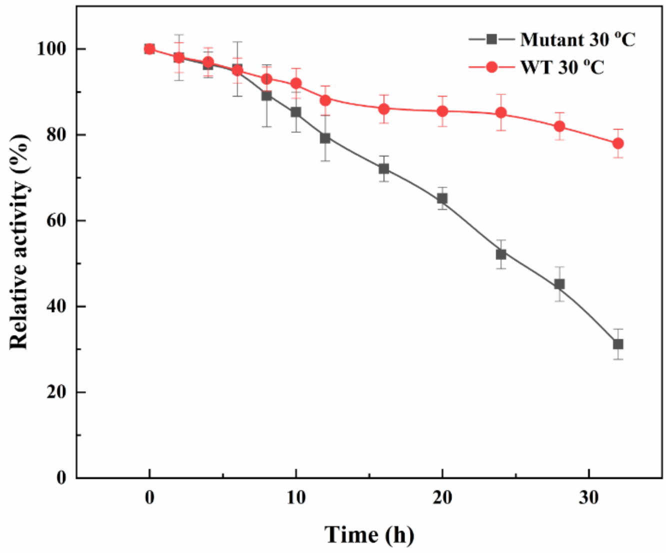

Supplementary Figure 30. Thermal stability (30 ºC) of YqjM (C26F).

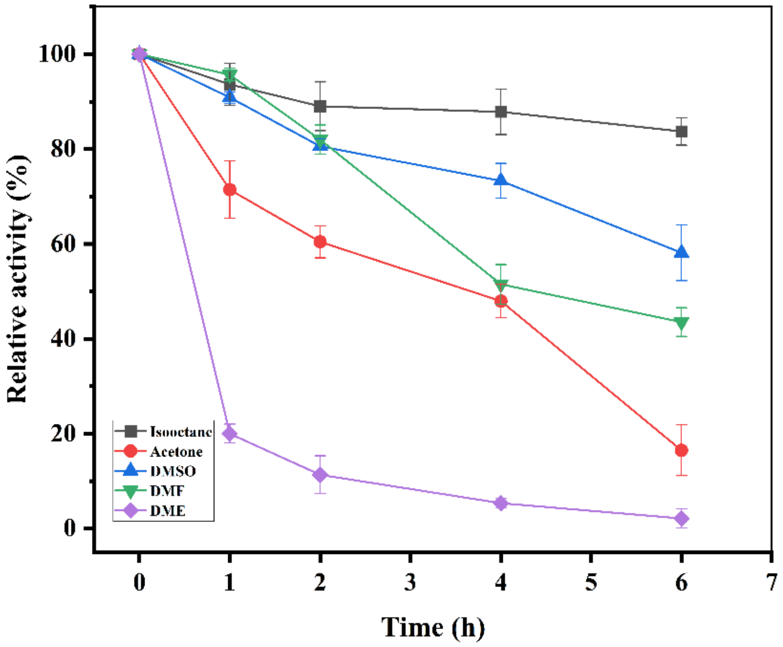

Supplementary Figure 31. Effect of co-solvents (5% v/v) on activity of YqjM (I69Y/C26G) in PBS (100 mM, pH 7.5) containing 10 mM **1a**.

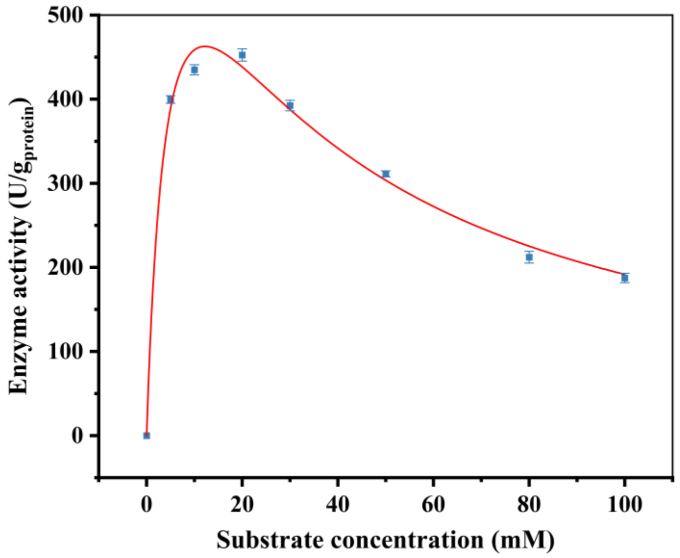

Supplementary Figure 32. Effect of substrate (**1a**) concentration on activity of YqjM (I69Y/C26G) in PBS (100 mM, pH 7.5) containing 10% v/v isooctane.

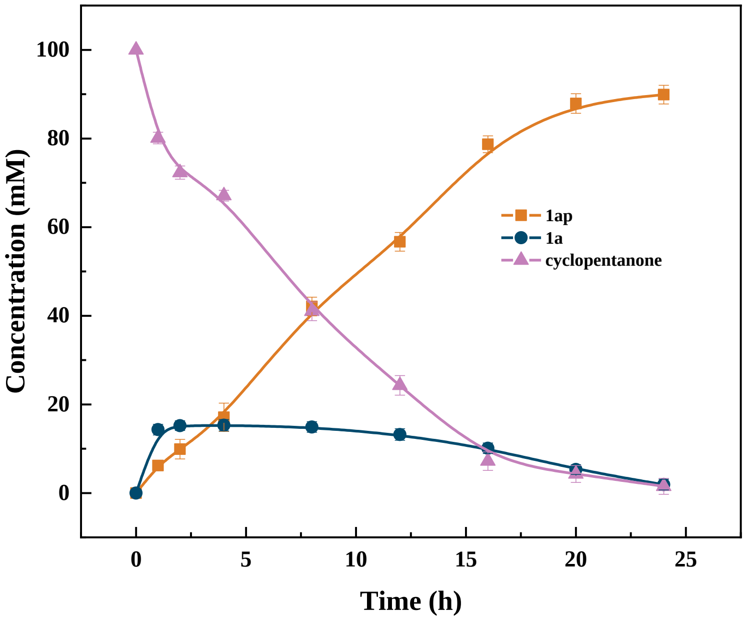

Supplementary Figure 33. Reaction time curves of chemoenzymatic cascade for α-benzylation of cyclic ketones.

Supplementary Tables

Supplementary Table 1. Gene sequence of wt-YqjM.

ATGGCCAGAAAATTATTTACACCTATTACAATTAAAGATATGACGTTAAAAAACCGCA
TTGTCATGTCGCCAATGTGCATGTATTCTTCTCATGAAAAGGACGGAAAATTAACACC
GTTCCACATGGCACATTACATATCGCGCGCAATCGGCCAGGTCGGACTGATTATTGTA
GAGGCGTCAGCGGTTAACCCTCAAGGACGAATCACTGACCAAGACTTAGGCATTTG
GAGCGACGAGCATATTGAAGGCTTTGCAAAACTGACTGAGCAGGTCAAAGAACAA
GGTTCAAAAATCGGCATTCAGCTTGCCCATGCCGGACGTAAAGCTGAGCTTGAAGG
AGATATCTTCGCTCCATCGGCGATTGCGTTTGACGAACAATCAGCAACACCTGTAGA
AATGTCAGCAGAAAAAGTAAAAGAAACGGTCCAGGAGTTCAAGCAAGCGGCTGCC
CGCGCAAAAGAAGCCGGCTTTGATGTGATTGAAATTCATGCGGCGCACGGATATTTA
ATTCATGAATTTTTGTCTCCGCTTTCCAACCATCGAACAGATGAATATGGCGGCTCAC
CTGAAAACCGCTATCGTTTCTTGAGAGAGATCATTGATGAAGTCAAACAAGTATGGG
ACGGTCCTTTATTTGTCCGTGTATCTGCTTCTGACTACACTGATAAAGGCTTAGACAT
TGCCGATCACATCGGTTTTGCAAAATGGATGAAGGAGCAGGGTGTTGACTTAATTGA
CTGCAGCTCAGGCGCCCTTGTTCACGCAGACATTAACGTATTCCCTGGCTATCAGGT
CAGCTTCGCTGAGAAAATCCGTGAACAGGCGGACATGGCTACTGGTGCCGTCGGCA
TGATTACAGACGGTTCAATGGCTGAAGAAATTCTGCAAAACGGACGTGCCGACCTC
ATCTTTATCGGCAGAGAGCTTTTGCGGGATCCATTTTTTGCAAGAACTGCTGCGAAA
CAGCTCAATACAGAGATTCCGGCCCCTGTTCAATACGAAAGAGGCTGGTAA

Supplementary Table 2. Primers used for site-directed mutagenesis.

Mutants	Primers
I69A-F	GCGGTTAACCCTCAAGGACGAGCGACTGACCAAGACTTAGGCATTTG
I69A-R	GCCTAAGTCTTGGTCAGTCGCTCGTCCTTGAGGGTTAACCGCTGAC
I69G-F	GCGGTTAACCCTCAAGGACGAGGCACTGACCAAGACTTAGGCATTTG
I69G-R	GCCTAAGTCTTGGTCAGTGCCTCGTCCTTGAGGGTTAACCGCTGAC
I69V-F	GCGGTTAACCCTCAAGGACGAGTAACTGACCAAGACTTAGGCATTTG
I69V-R	GCCTAAGTCTTGGTCAGTTACTCGTCCTTGAGGGTTAACCGCTGAC
I69Y-F	GCGGTTAACCCTCAAGGACGATATACTGACCAAGACTTAGGCATTTG
I69Y-R	GCCTAAGTCTTGGTCAGTATATCGTCCTTGAGGGTTAACCGCTGAC
C26A-F	CATTGTCATGTCGCCAATGGCAATGTATTCTTCTCATGAAAAGGACG
C26A-R	CTTTTCATGAGAAGAATACATTGCCATTGGCGACATGACAATGCGG
C26G-F	CATTGTCATGTCGCCAATGGGCATGTATTCTTCTCATGAAAAGGACG
C26G-R	CTTTTCATGAGAAGAATACATGCCCATTGGCGACATGACAATGCGG
Y28A-F	GTCATGTCGCCAATGTGCATGGCATCTTCTCATGAAAAGGACGG
Y28A-R	CCGTCCTTTTCATGAGAAGATGCCATGCACATTGGCGACATGACAATG
Y28I-F	GTCATGTCGCCAATGTGCATGATATCTTCTCATGAAAAGGACGG
Y28I-R	CCGTCCTTTTCATGAGAAGATGCCATATACATTGGCGACATGACAATG
Y28V-F	GTCATGTCGCCAATGTGCATGGTATCTTCTCATGAAAAGGACGG
Y28V-R	CCGTCCTTTTCATGAGAAGATGCCATACACATTGGCGACATGACAATG
Y169F-F	GAAATTCATGCGGCGCACGGATTCTTAATTCATGAATTTTTGTCTCCG
Y169F-R	GACAAAAATTCATGAATTAAGAATCCGTGCGCCGCATGAATTTC
C26W-F	CATTGTCATGTCGCCAATGTGGATGTATTCTTCTCATGAAAAGGACG
C26W-R	CTTTTCATGAGAAGAATACATCCACATTGGCGACATGACAATGCGG
C26F-F	CATTGTCATGTCGCCAATGTTCATGTATTCTTCTCATGAAAAGGACG
C26F-R	CTTTTCATGAGAAGAATACATGAACATTGGCGACATGACAATGCGG

Supplementary Table 3. Docking energies of different mutants with **1a**.

Entry	Mutants	Binding Energy (KJ/mol)[a]
1	wt	-1.13
2	I69W	-0.48
3	I69C	-0.56
4	I69M	-0.87
5	I69D	-1.18
6	I69R	-1.21
7	I69N	-1.21
8	I69H	-1.21
9	I69E	-1.22
10	I69K	-1.22
11	I69P	-1.28
12	I69Q	-1.32
13	I69S	-1.35
14	I69T	-1.52
15	I69L	-1.72
16	I69F	-1.82
17	I69A	-2.35
18	I69V	-2.35
19	I69G	-2.55
20	I69Y	-2.58

[a] Binding energy was calculated by molecular docking. AutoDock 4.2 was used for the docking of **1a** into the model structures. A grid box of 40 × 40 × 40 with spacing of 1.0 Å, which encompassed the active sites of YqjM mutants, was set as the search space to explore suitable substrate-protein binding modes and binding energies. The PDBQT format was employed for both the input and output of molecular structures.

Supplementary Table 4. Specific activity of different mutants toward **1a**.

Entry	Mutants	Specific activity (U/$g_{protein}$)[a]
1	wt	13.2
2	I69W	ND[b]
3	I69C	ND[b]
4	I69M	12.9
5	I69D	21.2
6	I69R	12.3
7	I69N	21.5
8	I69H	15.2
9	I69E	12.2
10	I69K	12.5
11	I69P	13.2
12	I69Q	25.8
13	I69S	23.5
14	I69T	53.2
15	I69L	15.6
16	I69F	35.6
17	I69A	45.2
18	I69V	33.4
19	I69G	58.5
20	I69Y	125.6

[a] Specific activity was calculated using the conversion at 1 min. U refers to the activity unit expressed as micromoles of substrate converted per min. Reactions were carried out in PBS (1.0 mL, 100 mM, pH 7.5) containing 1.0 mM **1a**, 2.0 mM NADPH, and 10 μM YqjM mutants. Data are obtained from three independent experiments and mean values are presented. Source data are provided as a Source data file. [b] ND means not detected.

Supplementary Table 5. DFT calculation results for wt-YqjM.[a]

	Structure	G(Ha)
A	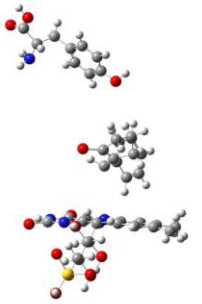	-3123.02413
B	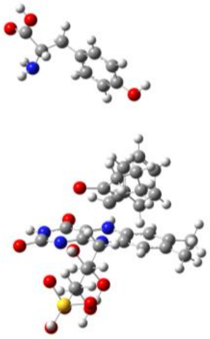	-3122.99412
C	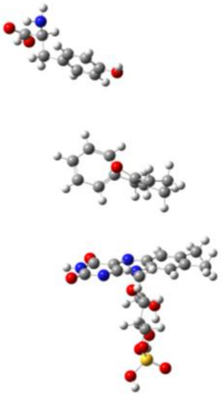	-3123.01294
D	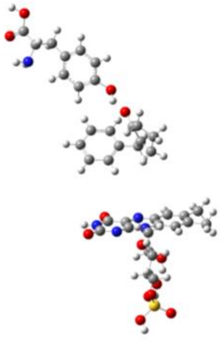	-3122.98468

F	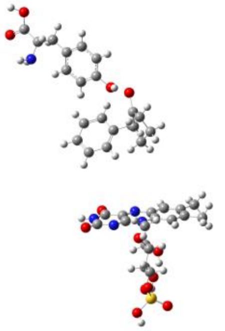	-3123.01763
G	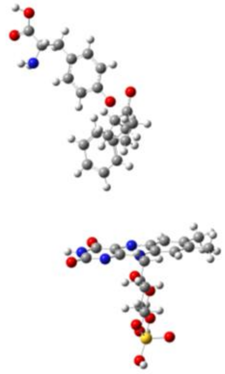	-3122.99612
H	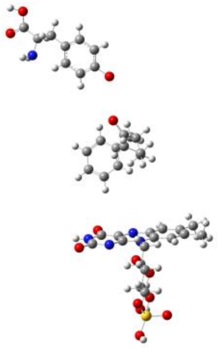	-3123.02637

[a] Free energy profile for the reduction of **1a** by YqjM at the B3LYP+D3/6-311++G(2d,p) (SMD, solvent = water)//B3LYP+D3/6-31G(d) (SMD, solvent = water) level of theory. The values shown are free energies and energies (in brackets) in kcal/mol.

Supplementary Table 6. DFT calculation results for YqjM (I69Y/Y169F).[a]

	Structure	G(Ha)
Aw	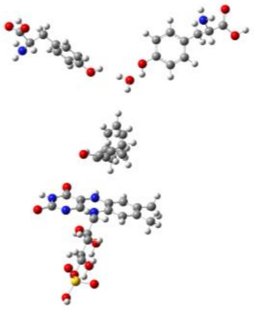	-3829.32006
Bw	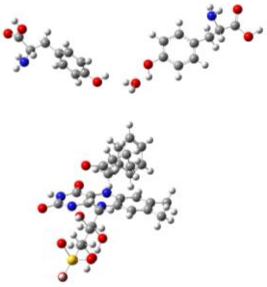	-3829.29005
Cw	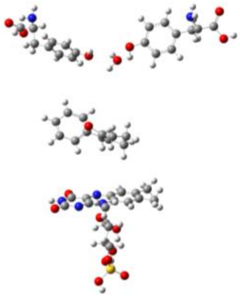	-3829.30890
Ew	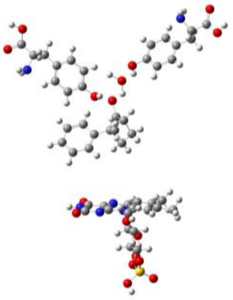	-3829.31448

Gw	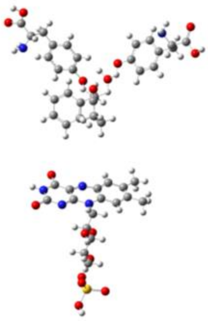	-3829.30588
Hw	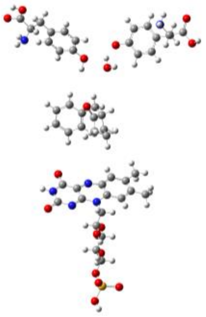	-3829.32229

[a] Free energy profile for the reduction of **1a** by YqjM at the B3LYP+D3/6-311++G(2d,p) (SMD, solvent = water)//B3LYP+D3/6-31G(d) (SMD, solvent = water) level of theory. The values shown are free energies and energies (in brackets) in kcal/mol.

Supplementary Table 7. Optimization of the enzymatic reduction conditions.[a]

Entry	Cosolvents	pH	Temperature (°C)	Time (h)	Conv. (%)[b]	ee (%)[b]
1	-	8	35	12	46	97
2	DMF (5%)	8	35	12	16	96
3	DME (5%)	8	35	12	ND[e]	ND
4	Acetone (5%)	8	35	12	11	95
5	DMSO (5%)	8	35	12	32	97
6	Isooctane (5%)	8	35	12	53	97
7	Isooctane (10%)	8	35	12	78	98
8	Isooctane (15%)	8	35	12	41	97
9	Isooctane (10%)	7.5	35	12	85	98
10	Isooctane (10%)	7	35	12	68	98
11	Isooctane (10%)	8.5	35	12	49	97
12	Isooctane (10%)	7.5	30	12	96	98
13	Isooctane (10%)	7.5	25	12	82	97
14	Isooctane (10%)	7.5	40	12	33	98
15[c]	Isooctane (10%)	7.5	30	24	49	98
16[d]	Isooctane (10%)	7.5	30	24	26	97

[a] Reactions were carried out in PBS (100 mM, pH 7.5) containing 10 mM **1a**, 0.1 mM $NADP^+$, 20 mM glucose, 0.1 mM YqjM variants, and 0.2 mM GDH. [b] The conversion and the values of ee were determined by GC. Mean values from triplicates are presented. Source data are provided as a Source data file. [c] Using 50 mM **1a**. [d] Using 100 mM **1a**. [e] ND means not detected.

Supplementary Table 8. Optimization of the concentrations of enzyme and cofactor.[a]

Entry	YqjM (μM)	$NADP^+$ (μM)	Conv. (%)[b]	ee (%)[b]	TON
1	2	50	30	98	1500
2	4	50	53	97	1325
3	6	50	91	98	1517
4	8	50	94	98	1175
5	10	50	92	97	920
6	6	10	32	98	534
7	6	20	52	97	867
8	6	30	67	98	1117
9	6	40	85	98	1417

[a] Reactions were carried out in PBS (100 mM, pH 7.5) containing10% v/v isooctane, 10 mM **1a**, 0.01-0.05 mM $NADP^+$, 20 mM glucose, 2-10 μM YqjM (I69Y/C26G), and 20 μM GDH at 30 °C for 24 h. [b] The conversion and the values of ee were determined by GC. Mean values from triplicates are presented. Source data are provided as a Source data file.

NMR spectra

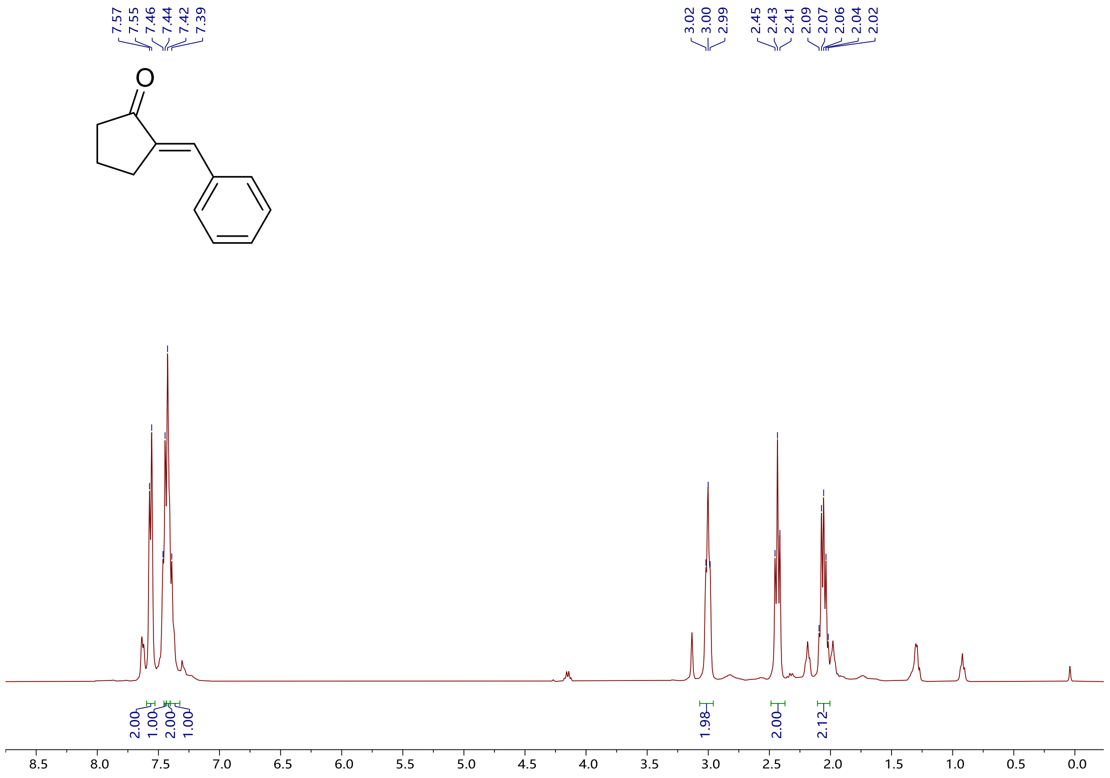
7.57
7.55
7.46
7.44
7.42
7.39
3.02
3.00
2.99
2.45
2.43
2.41
2.09
2.07
2.06
2.04
2.02
O
2.00
1.00
2.00
1.00
1.98
2.00
2.12
8.5
8.0
7.5
7.0
6.5
6.0
5.5
5.0
4.5
4.0
3.5
3.0
2.5
2.0
1.5
1.0
0.5
0.0

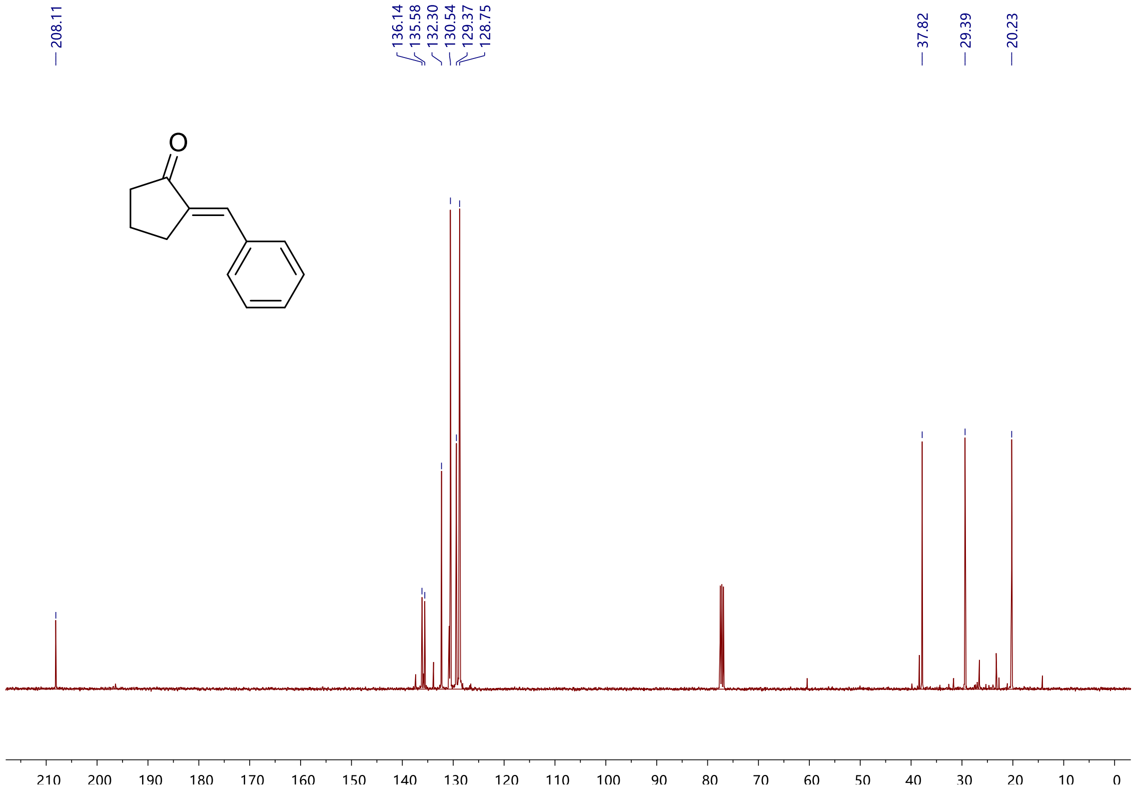
208.11
136.14
135.58
132.30
130.54
129.37
128.75
37.82
29.39
20.23
O
210
200
190
180
170
160
150
140
130
120
110
100
90
80
70
60
50
40
30
20
10
0

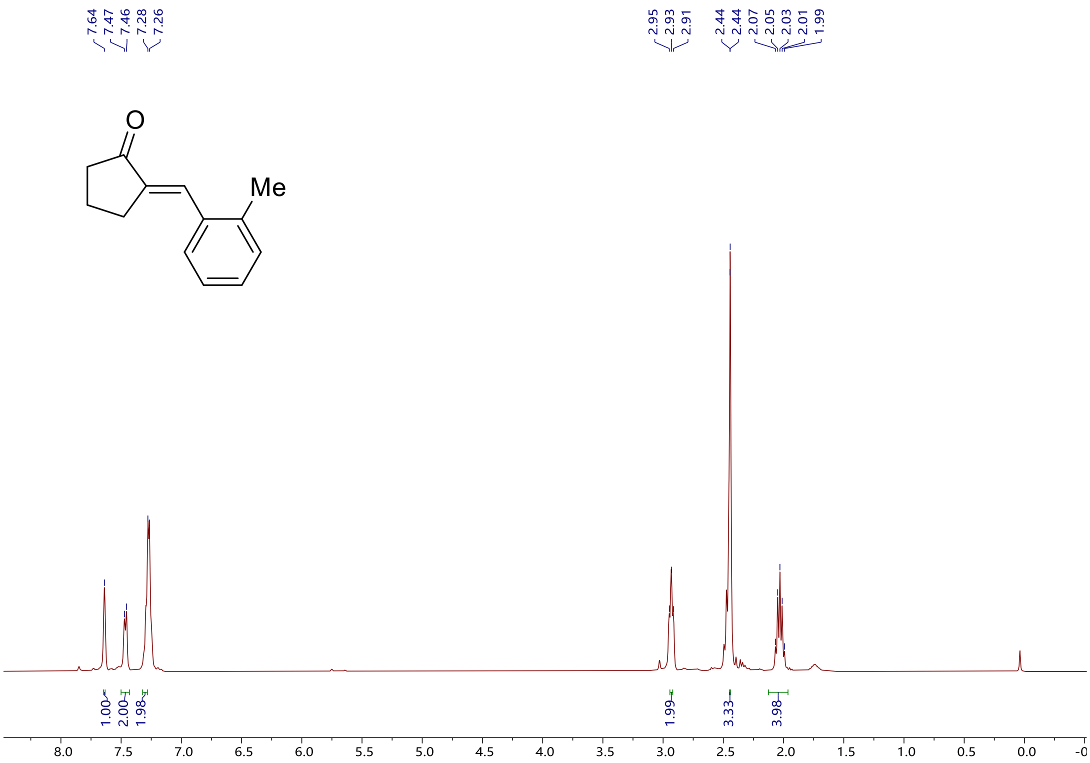

7.64
7.47
7.46
7.28
7.26
2.95
2.93
2.91
2.44
2.44
2.07
2.05
2.03
2.01
1.99
O
Me
1.00
2.00
1.98
1.99
3.33
3.98
8.0 7.5 7.0 6.5 6.0 5.5 5.0 4.5 4.0 3.5 3.0 2.5 2.0 1.5 1.0 0.5 0.0

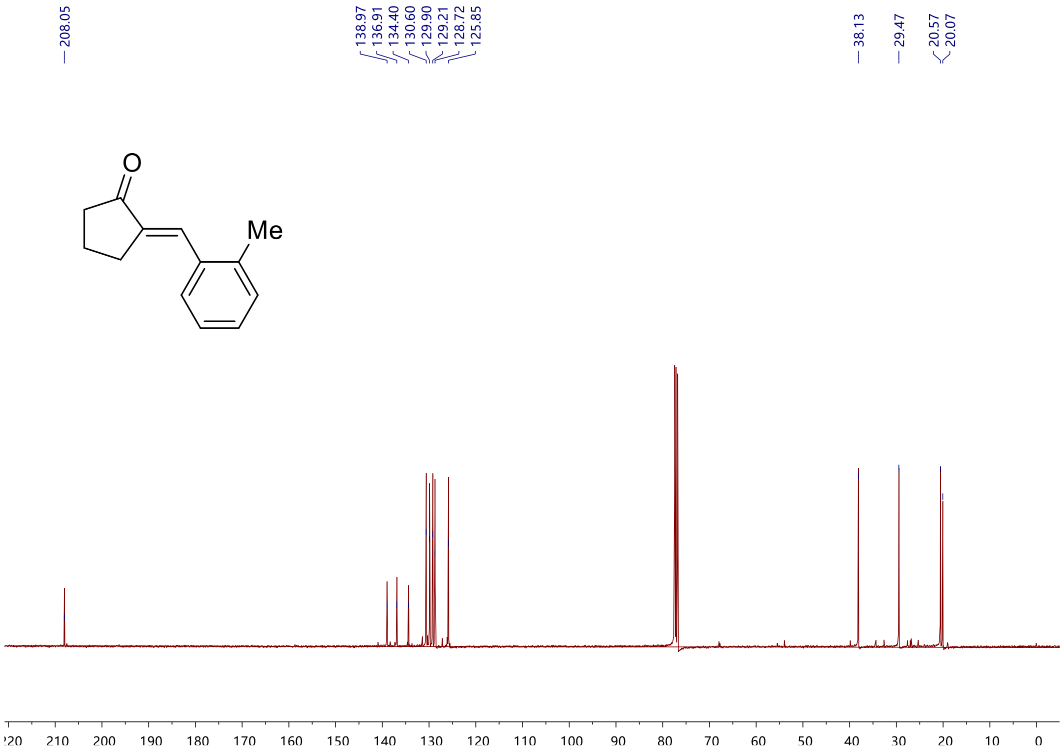

208.05
138.97
136.91
134.40
130.60
129.90
129.21
128.72
125.85
38.13
29.47
20.57
20.07
O
Me
210 200 190 180 170 160 150 140 130 120 110 100 90 80 70 60 50 40 30 20 10 0

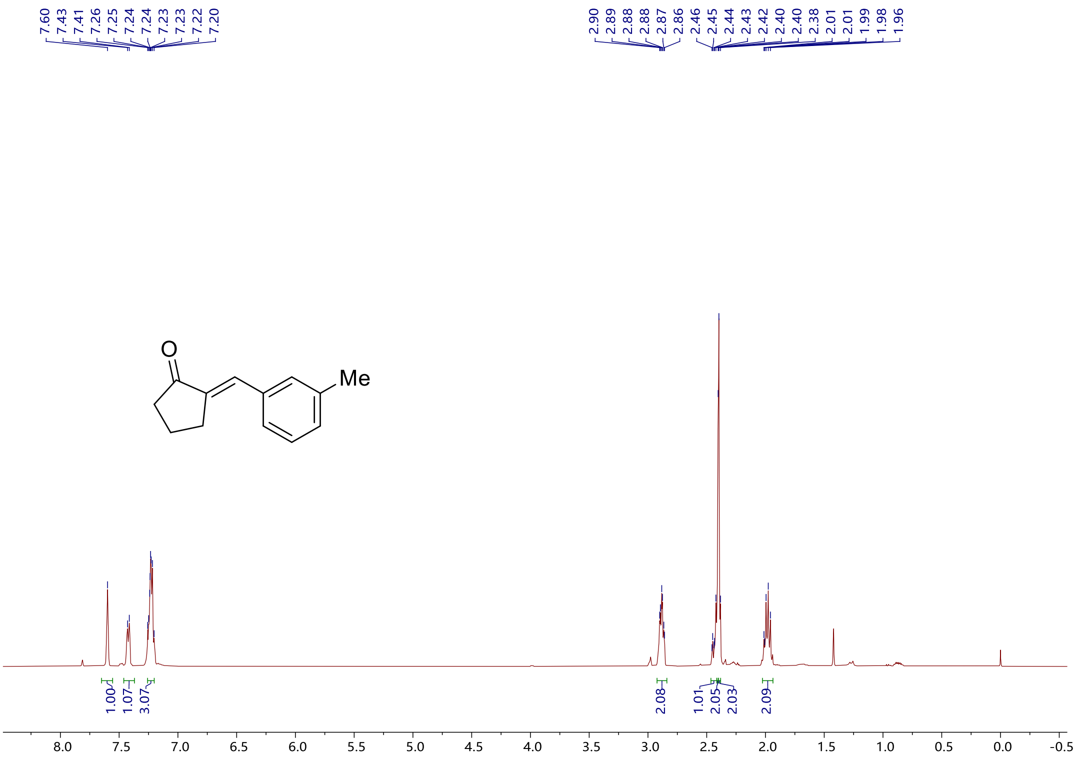
7.60
7.43
7.41
7.26
7.25
7.24
7.24
7.23
7.23
7.22
7.20
2.90
2.89
2.88
2.88
2.87
2.86
2.46
2.45
2.44
2.43
2.42
2.40
2.40
2.38
2.01
2.01
1.99
1.98
1.96
O
Me
1.00
1.07
3.07
2.08
1.01
2.05
2.03
2.09
8.0
7.5
7.0
6.5
6.0
5.5
5.0
4.5
4.0
3.5
3.0
2.5
2.0
1.5
1.0
0.5
0.0
-0.5

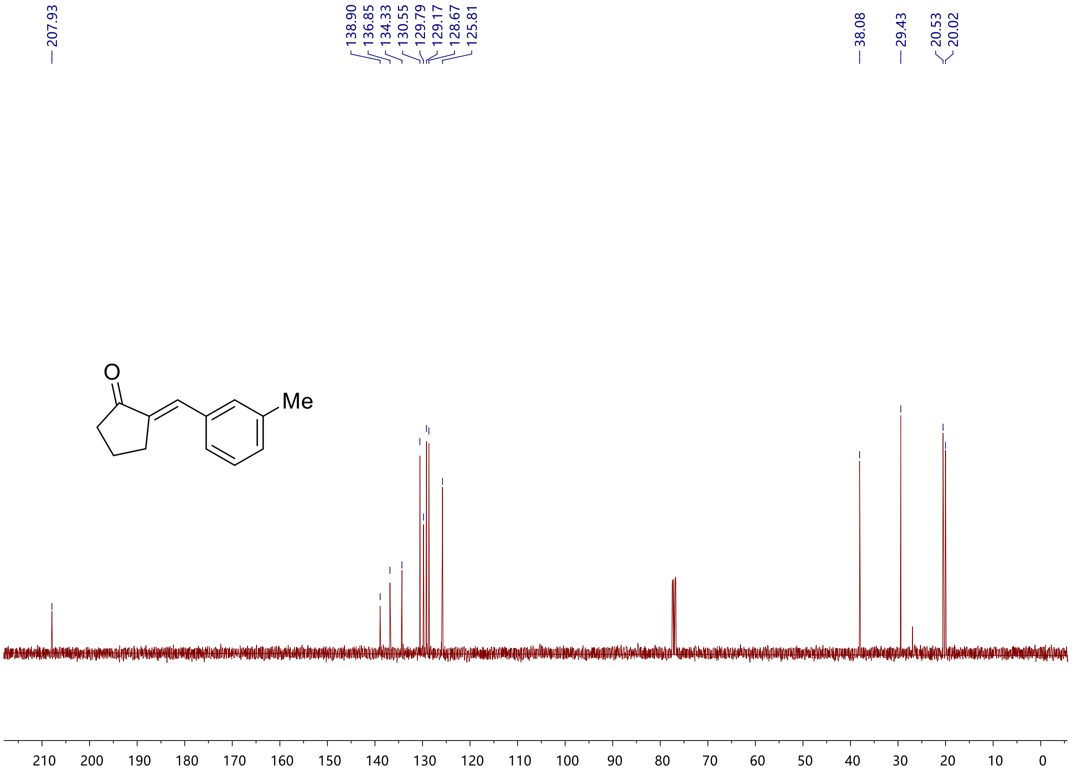
207.93
138.90
136.85
134.33
130.55
129.79
129.17
128.67
125.81
38.08
29.43
20.53
20.02
O
Me
210
200
190
180
170
160
150
140
130
120
110
100
90
80
70
60
50
40
30
20
10
0

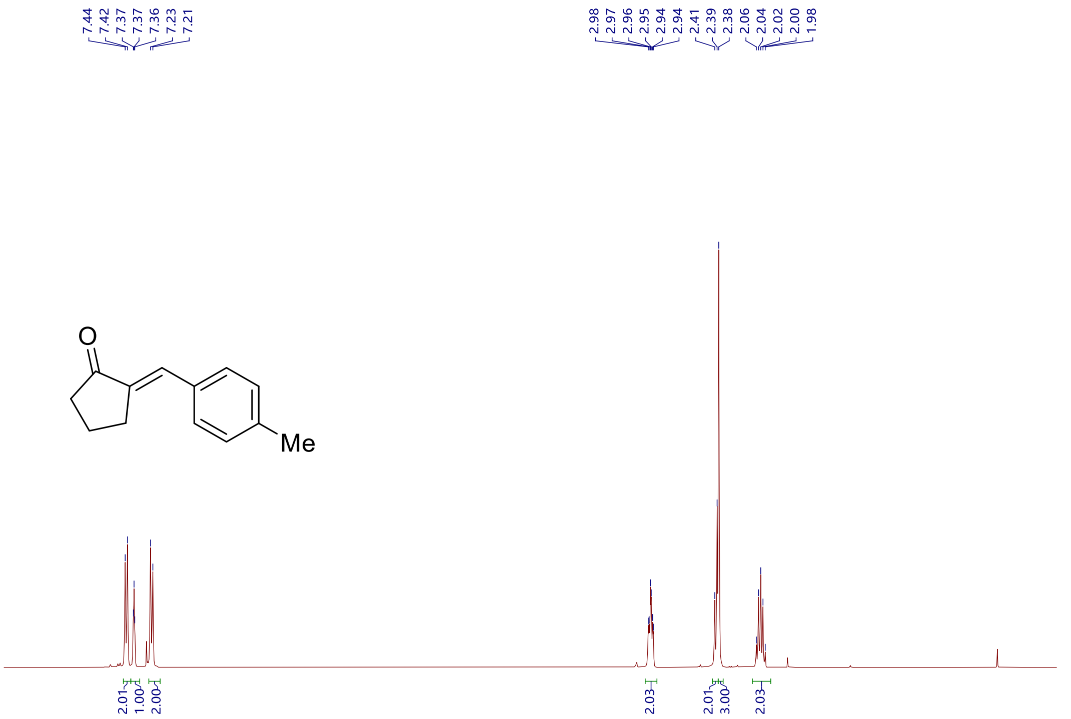

7.44
7.42
7.37
7.37
7.36
7.23
7.21
2.98
2.97
2.96
2.95
2.94
2.94
2.41
2.39
2.38
2.06
2.04
2.02
2.00
1.98
O
Me
2.01
1.00
2.00
2.03
2.01
3.00
2.03
8.0
7.5
7.0
6.5
6.0
5.5
5.0
4.5
4.0
3.5
3.0
2.5
2.0
1.5
1.0
0.5
0.0

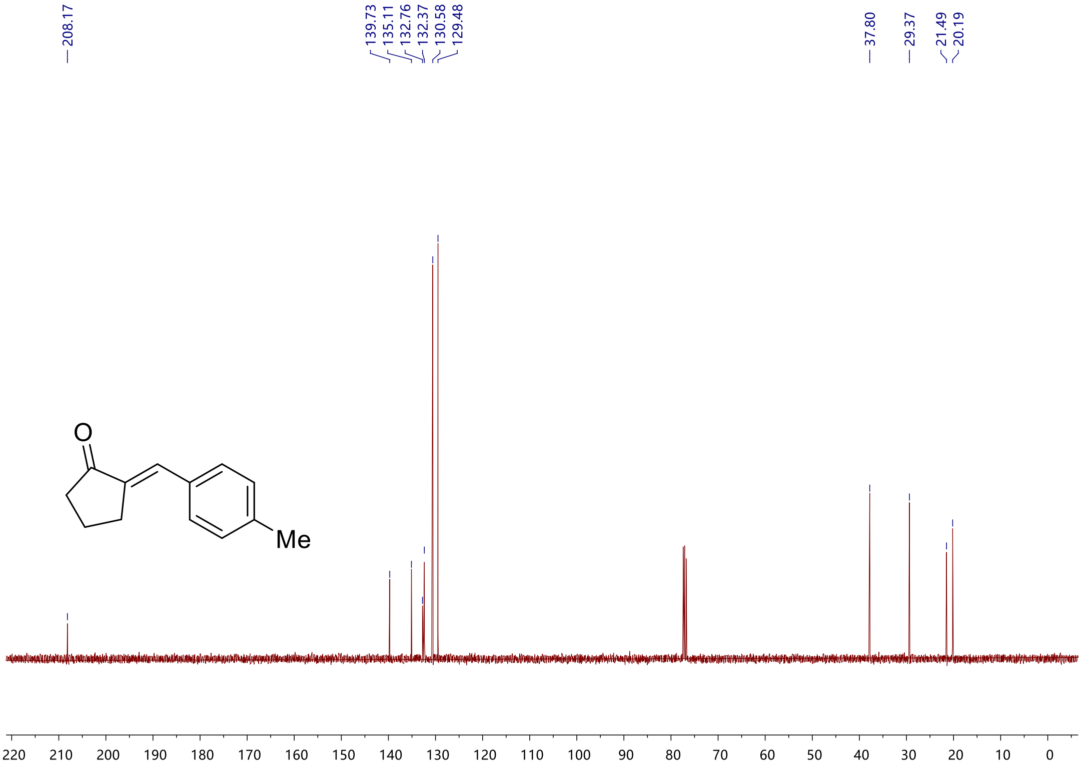

208.17
139.73
135.11
132.76
132.37
130.58
129.48
37.80
29.37
21.49
20.19
O
Me
220
210
200
190
180
170
160
150
140
130
120
110
100
90
80
70
60
50
40
30
20
10
0

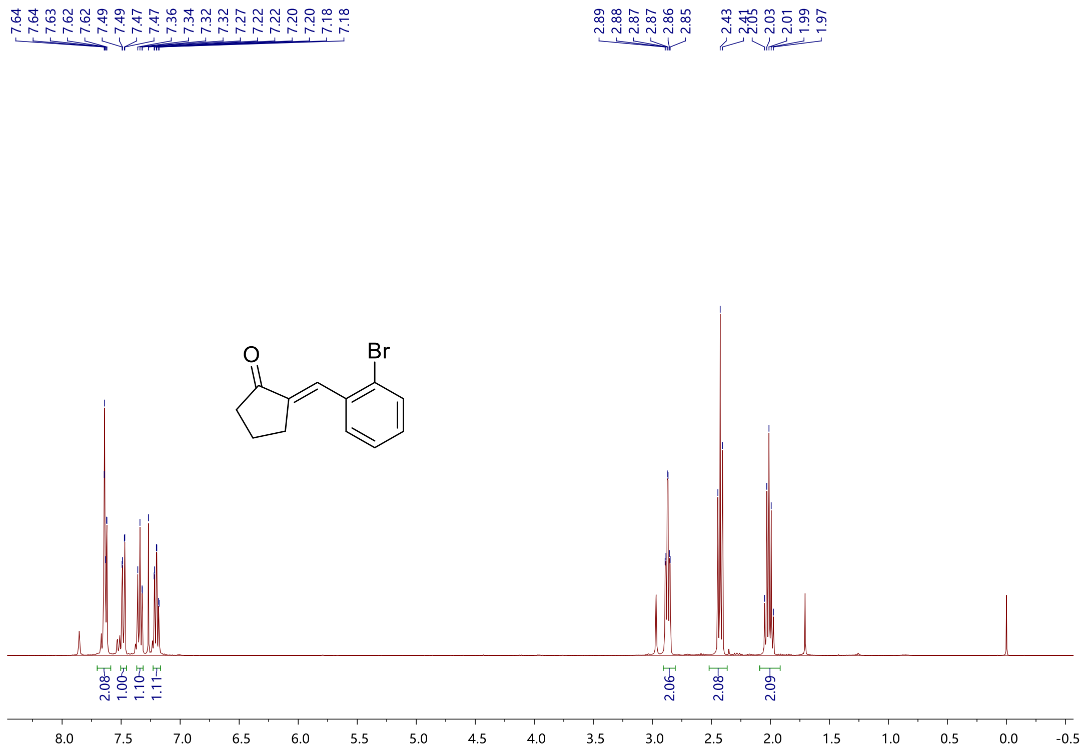
7.64
7.64
7.63
7.62
7.62
7.49
7.49
7.47
7.47
7.36
7.34
7.32
7.32
7.27
7.22
7.22
7.20
7.20
7.18
7.18
2.89
2.88
2.87
2.87
2.86
2.85
2.43
2.41
2.05
2.03
2.01
1.99
1.97
O
Br
2.08
1.00
1.10
1.11
2.06
2.08
2.09
8.0 7.5 7.0 6.5 6.0 5.5 5.0 4.5 4.0 3.5 3.0 2.5 2.0 1.5 1.0 0.5 0.0 -0.5

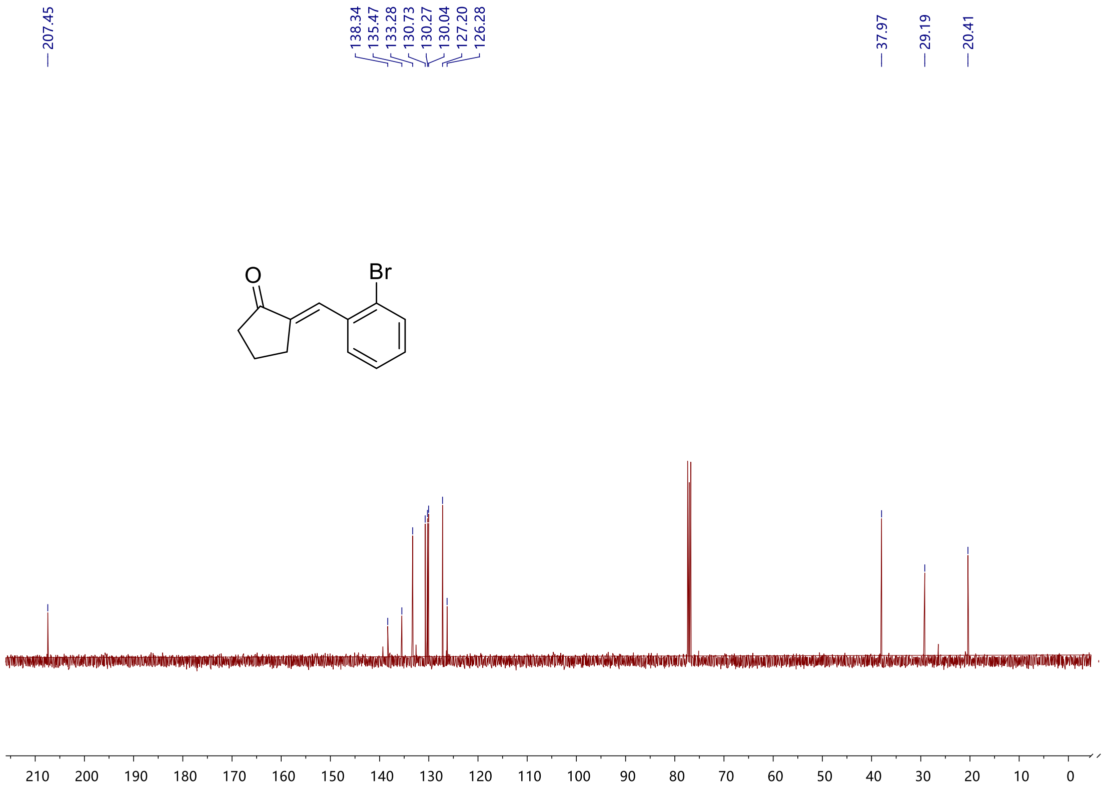
207.45
138.34
135.47
133.28
130.73
130.27
130.04
127.20
126.28
37.97
29.19
20.41
O
Br
210 200 190 180 170 160 150 140 130 120 110 100 90 80 70 60 50 40 30 20 10 0

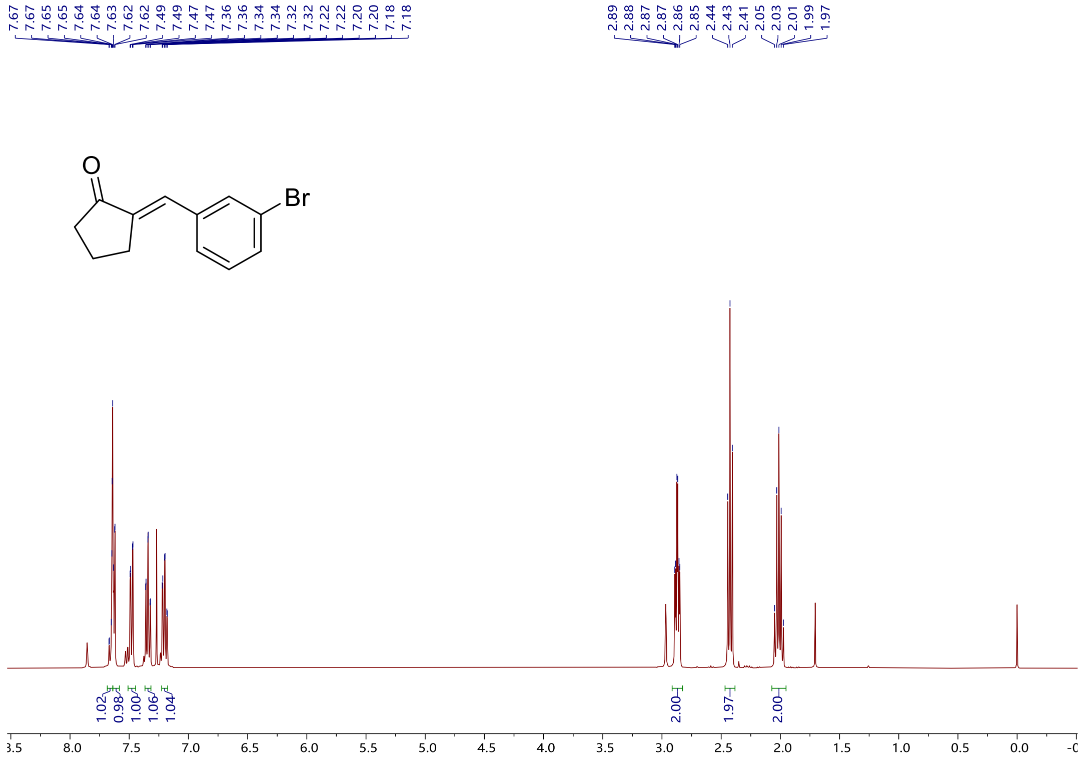
7.67 7.67 7.65 7.65 7.64 7.64 7.63 7.62 7.62 7.49 7.49 7.47 7.47 7.36 7.36 7.34 7.34 7.32 7.32 7.22 7.22 7.20 7.20 7.18 7.18
2.89 2.88 2.87 2.87 2.86 2.85 2.44 2.43 2.41 2.05 2.03 2.01 1.99 1.97
O
Br
1.02 0.98 1.00 1.06 1.04
2.00 1.97 2.00
8.5 8.0 7.5 7.0 6.5 6.0 5.5 5.0 4.5 4.0 3.5 3.0 2.5 2.0 1.5 1.0 0.5 0.0

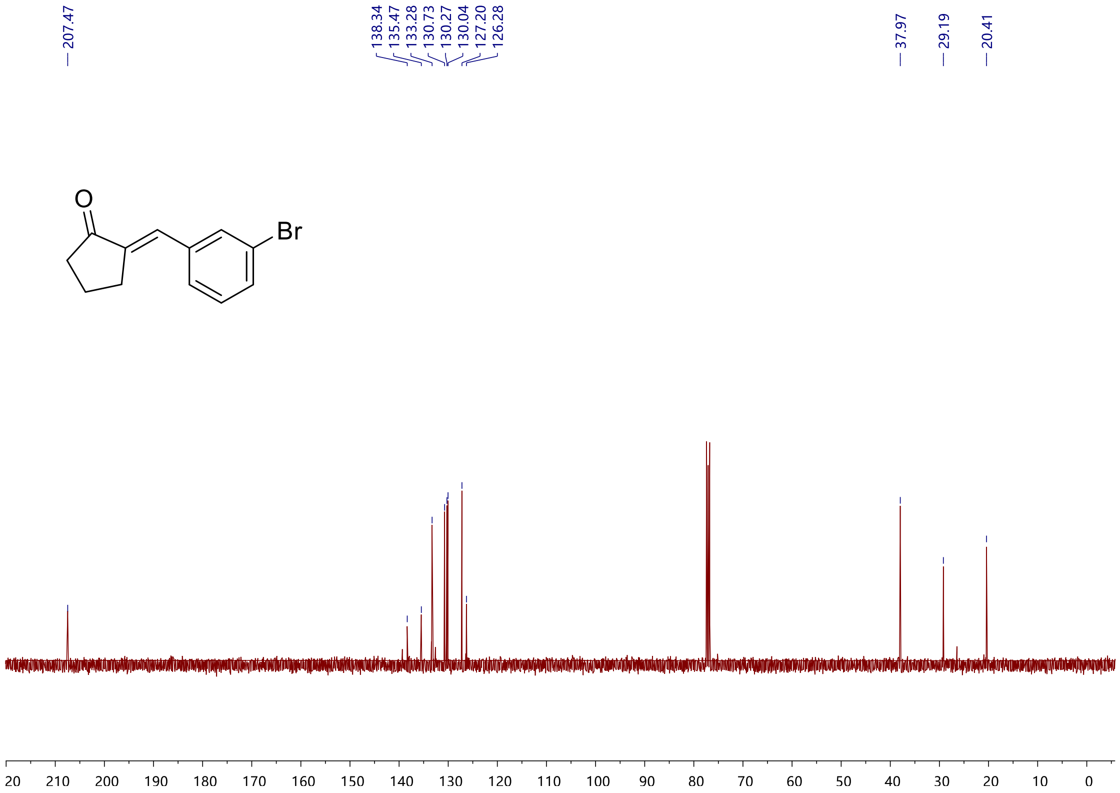
207.47
138.34 135.47 133.28 130.73 130.27 130.04 127.20 126.28
37.97
29.19
20.41
O
Br
220 210 200 190 180 170 160 150 140 130 120 110 100 90 80 70 60 50 40 30 20 10 0

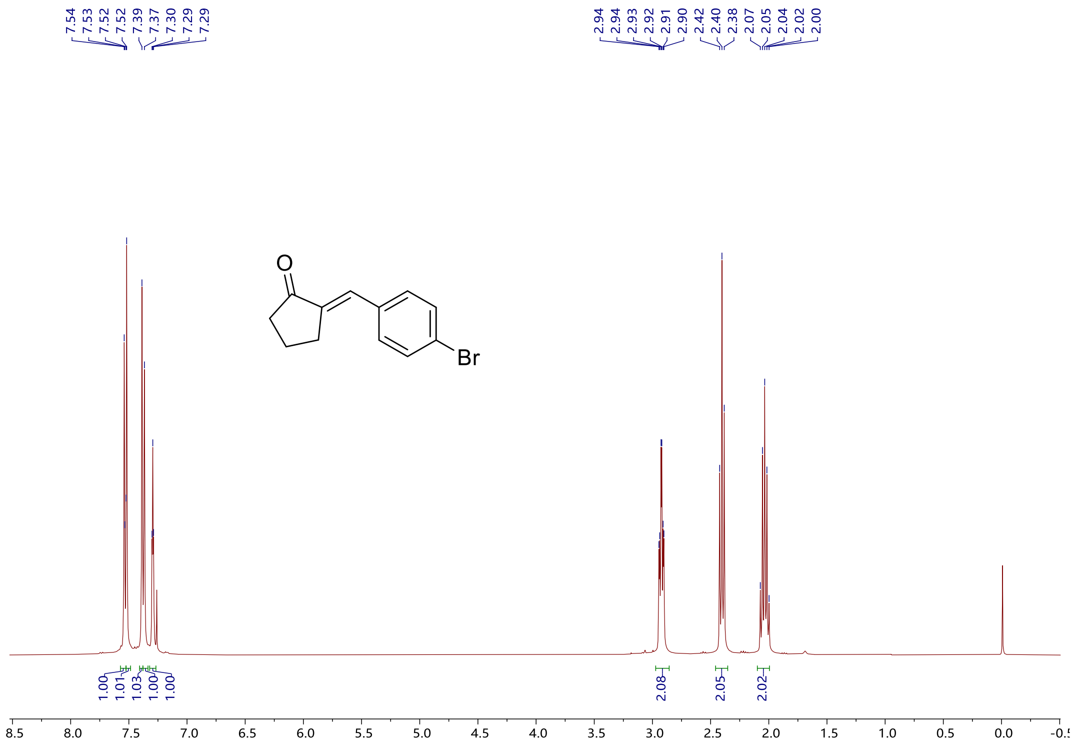
7.54
7.53
7.52
7.52
7.39
7.37
7.30
7.29
7.29
2.94
2.94
2.93
2.92
2.91
2.90
2.42
2.40
2.38
2.07
2.05
2.04
2.02
2.00
O
Br
1.00
1.01
1.03
1.00
1.00
2.08
2.05
2.02
8.5
8.0
7.5
7.0
6.5
6.0
5.5
5.0
4.5
4.0
3.5
3.0
2.5
2.0
1.5
1.0
0.5
0.0

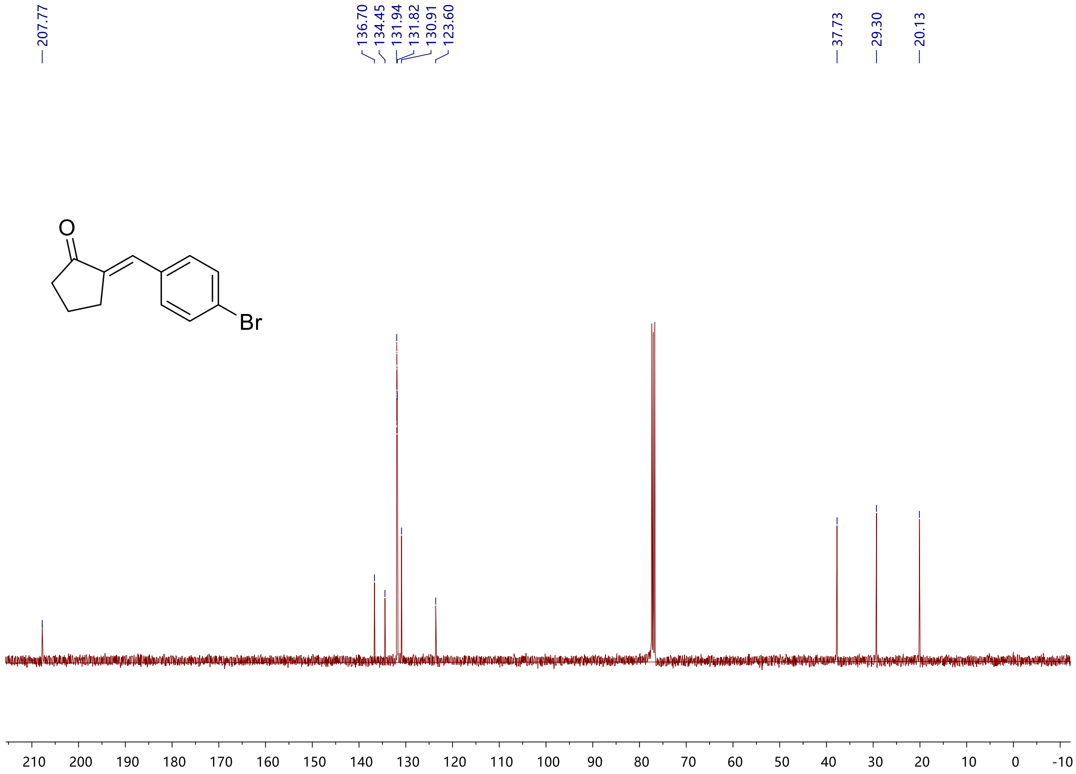
207.77
136.70
134.45
131.94
131.82
130.91
123.60
37.73
29.30
20.13
O
Br
210
200
190
180
170
160
150
140
130
120
110
100
90
80
70
60
50
40
30
20
10
0
-10

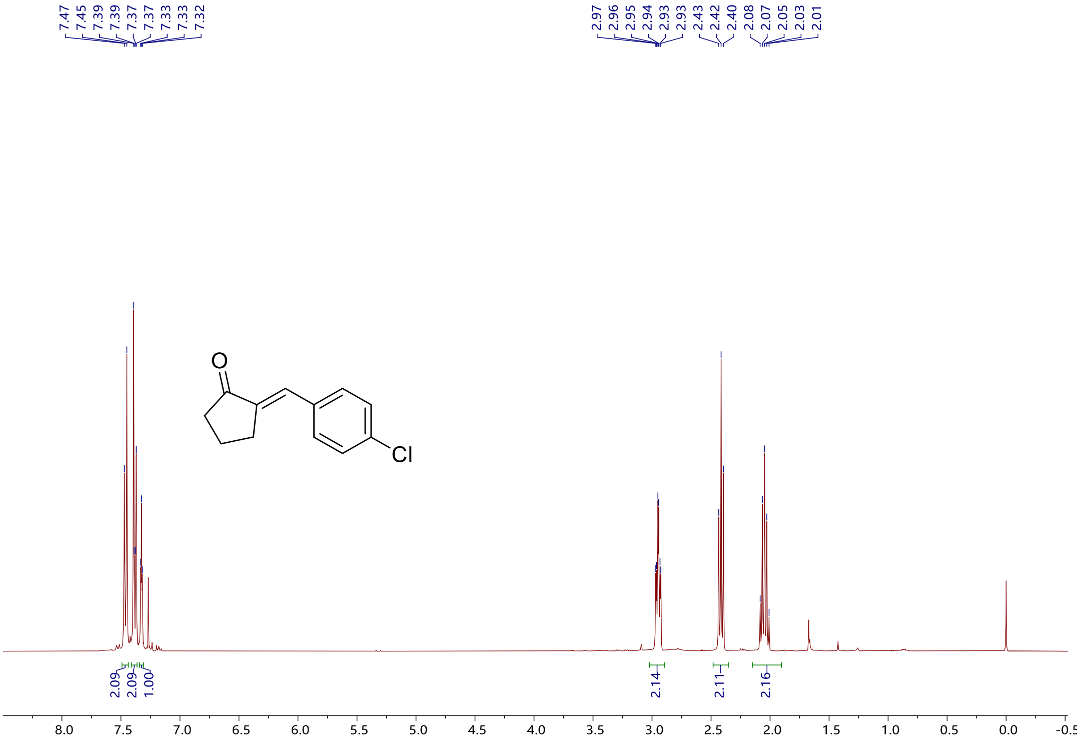
7.47
7.45
7.39
7.39
7.37
7.37
7.33
7.33
7.32
2.97
2.96
2.95
2.94
2.93
2.93
2.43
2.42
2.40
2.08
2.07
2.05
2.03
2.01
O
Cl
2.09
2.09
1.00
2.14
2.11
2.16
8.0
7.5
7.0
6.5
6.0
5.5
5.0
4.5
4.0
3.5
3.0
2.5
2.0
1.5
1.0
0.5
0.0
-0.5

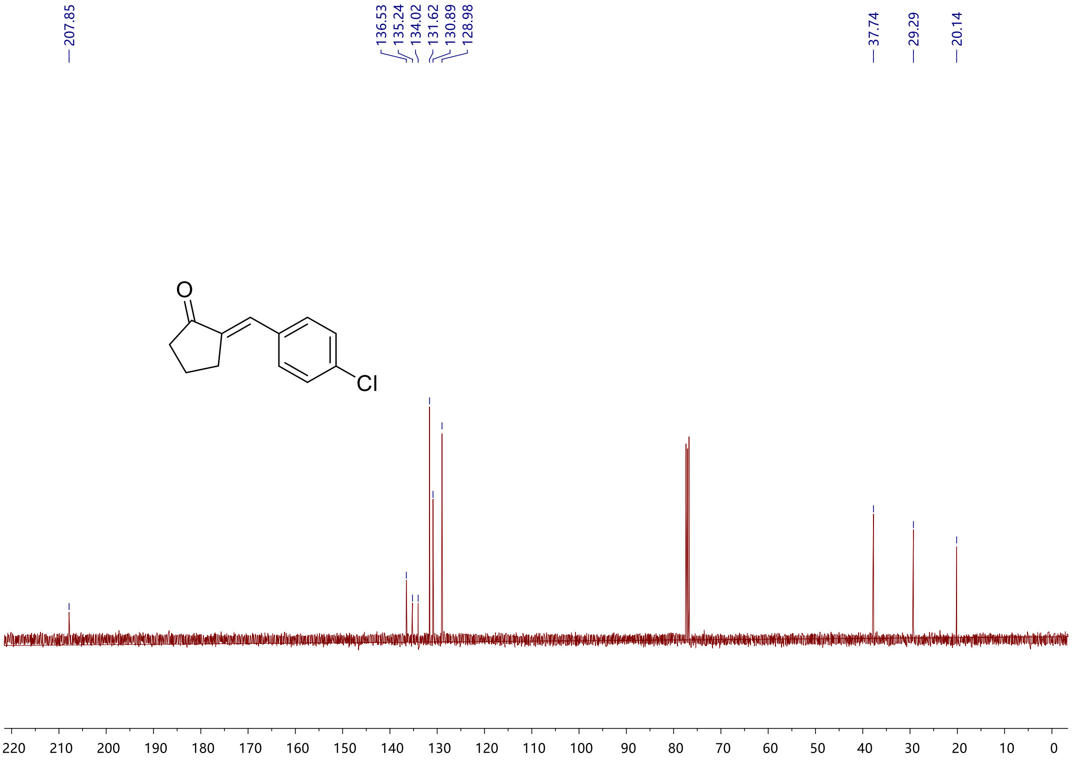
207.85
136.53
135.24
134.02
131.62
130.89
128.98
37.74
29.29
20.14
O
Cl
220
210
200
190
180
170
160
150
140
130
120
110
100
90
80
70
60
50
40
30
20
10
0

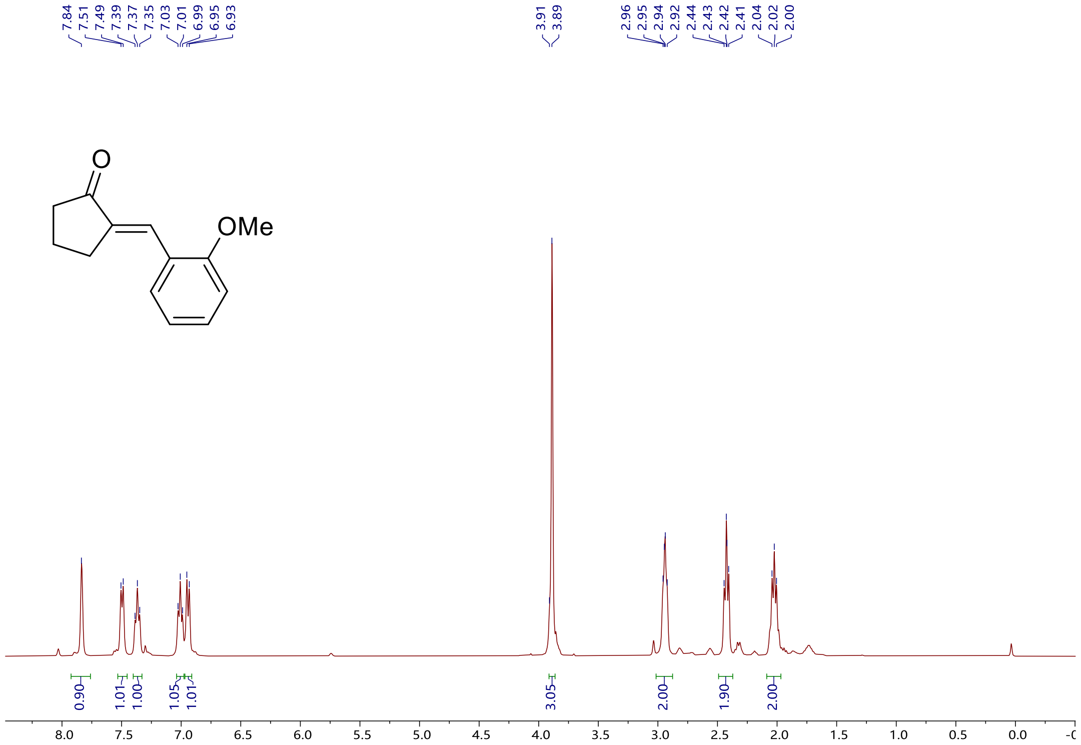
7.84
7.51
7.49
7.39
7.37
7.35
7.03
7.01
6.99
6.95
6.93
3.91
3.89
2.96
2.95
2.94
2.92
2.44
2.43
2.42
2.41
2.04
2.02
2.00
O
OMe
0.90
1.01
1.00
1.05
1.01
3.05
2.00
1.90
2.00

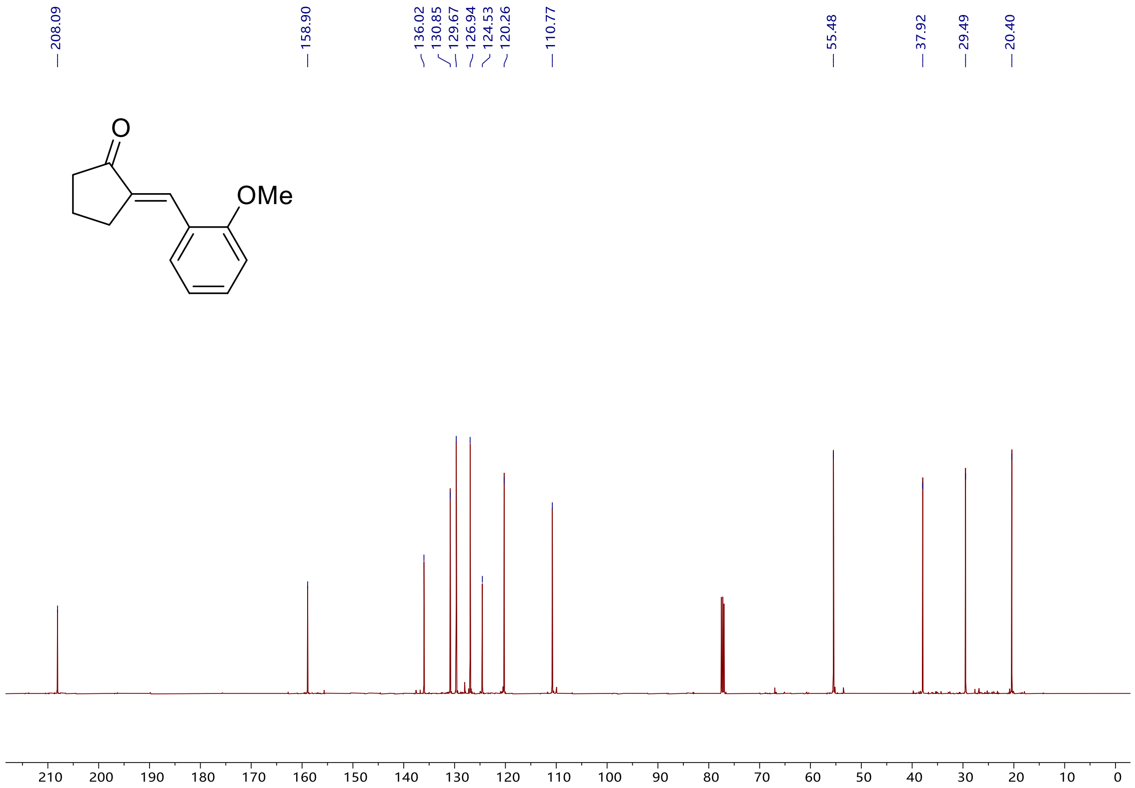
208.09
158.90
136.02
130.85
129.67
126.94
124.53
120.26
110.77
55.48
37.92
29.49
20.40
O
OMe

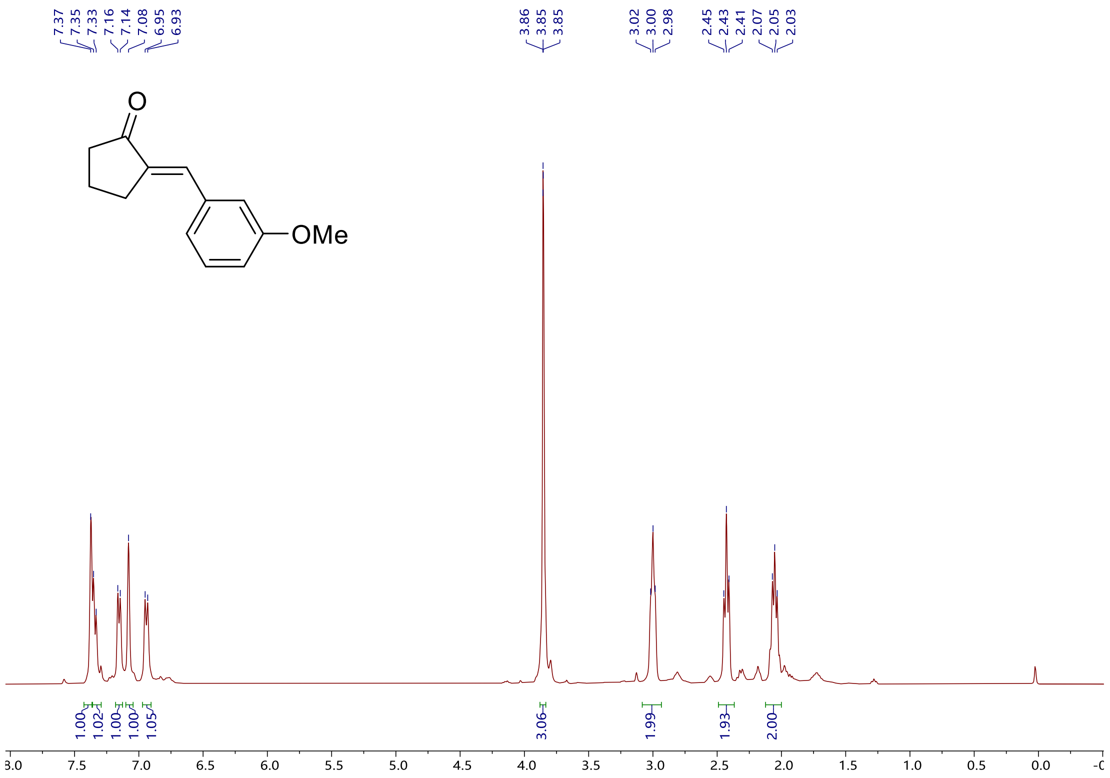
7.37 7.35 7.33 7.16 7.14 7.08 6.95 6.93
3.86 3.85 3.85
3.02 3.00 2.98
2.45 2.43 2.41 2.07 2.05 2.03
O
OMe
1.00 1.02 1.00 1.00 1.05
3.06
1.99
1.93
2.00
8.0 7.5 7.0 6.5 6.0 5.5 5.0 4.5 4.0 3.5 3.0 2.5 2.0 1.5 1.0 0.5 0.0

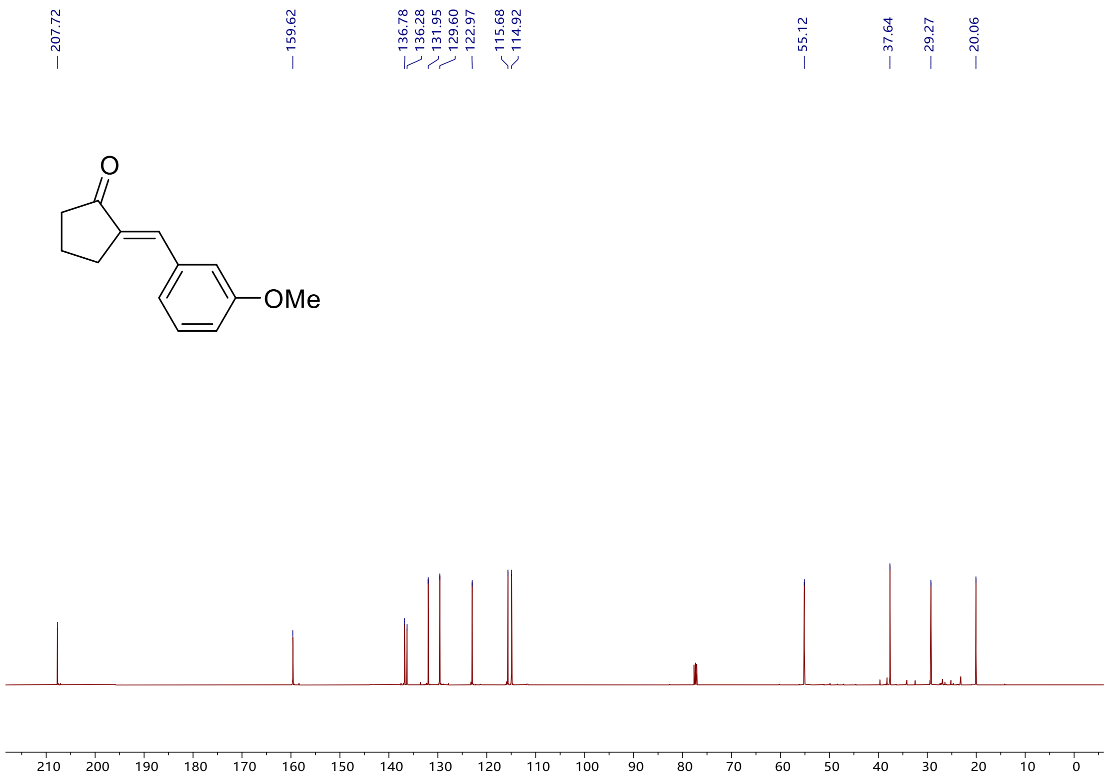
207.72
159.62
136.78 136.28 131.95 129.60 122.97 115.68 114.92
55.12
37.64
29.27
20.06
O
OMe
210 200 190 180 170 160 150 140 130 120 110 100 90 80 70 60 50 40 30 20 10 0

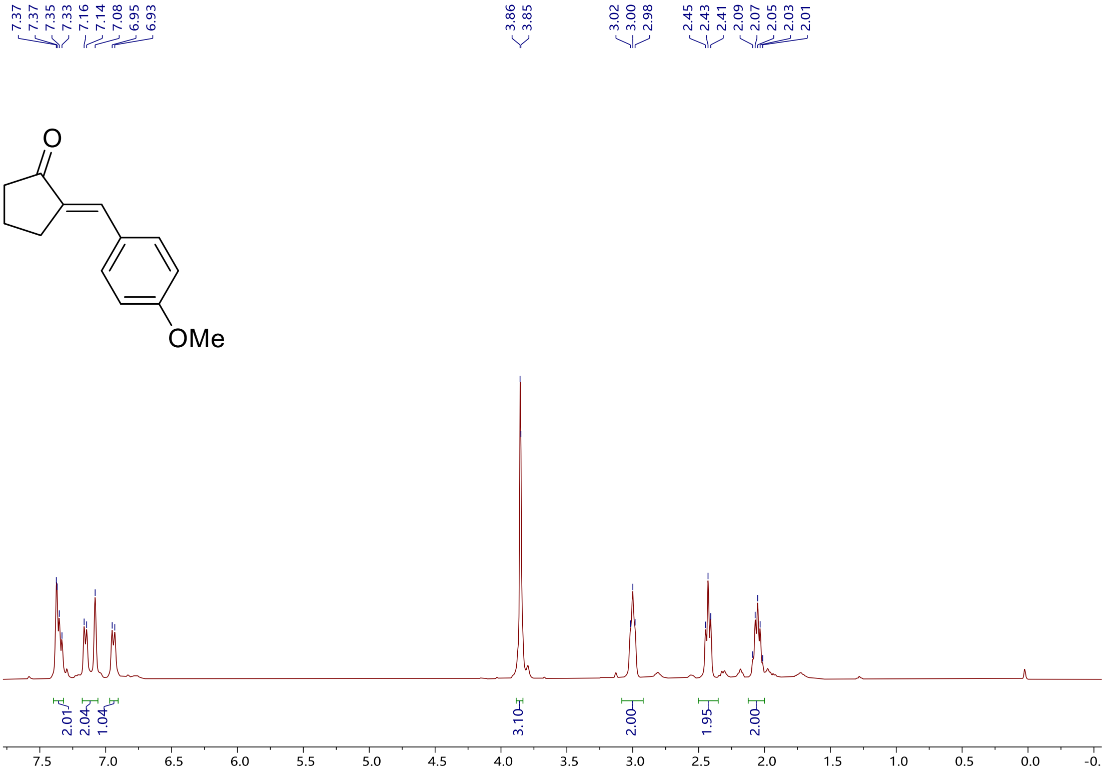
7.37
7.37
7.35
7.33
7.16
7.14
7.08
6.95
6.93
3.86
3.85
3.02
3.00
2.98
2.45
2.43
2.41
2.09
2.07
2.05
2.03
2.01
O
OMe
2.01
2.04
1.04
3.10
2.00
1.95
2.00

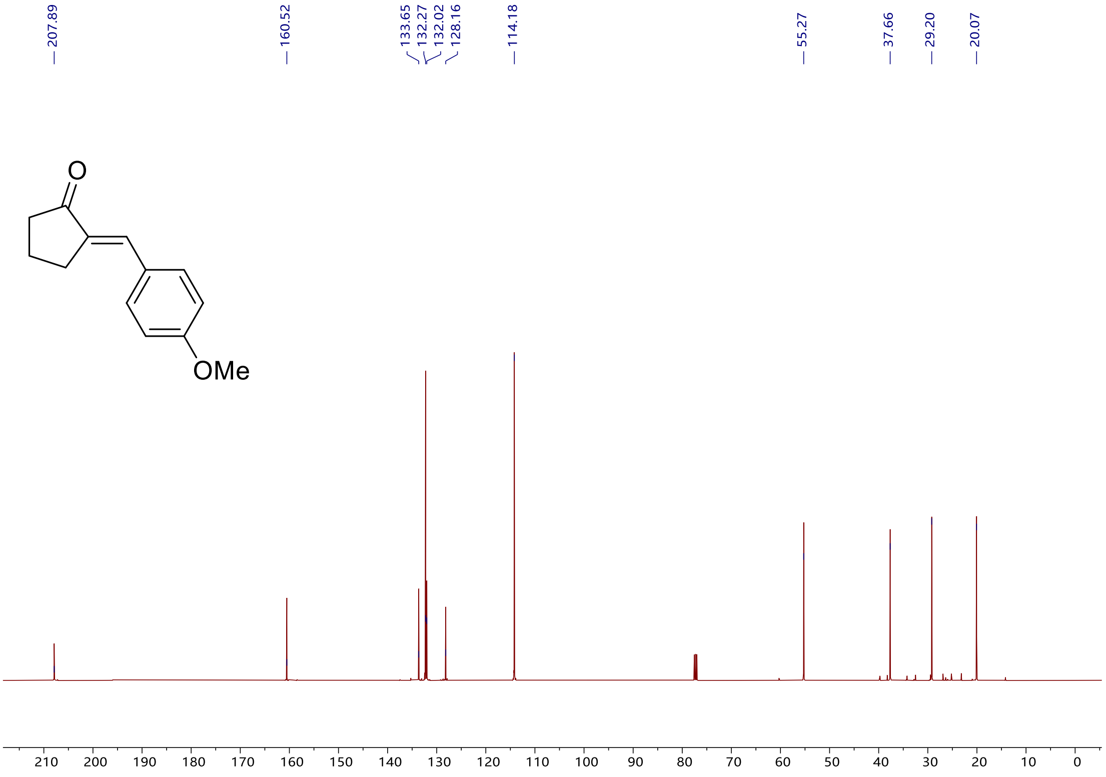
207.89
160.52
133.65
132.27
132.02
128.16
114.18
55.27
37.66
29.20
20.07
O
OMe

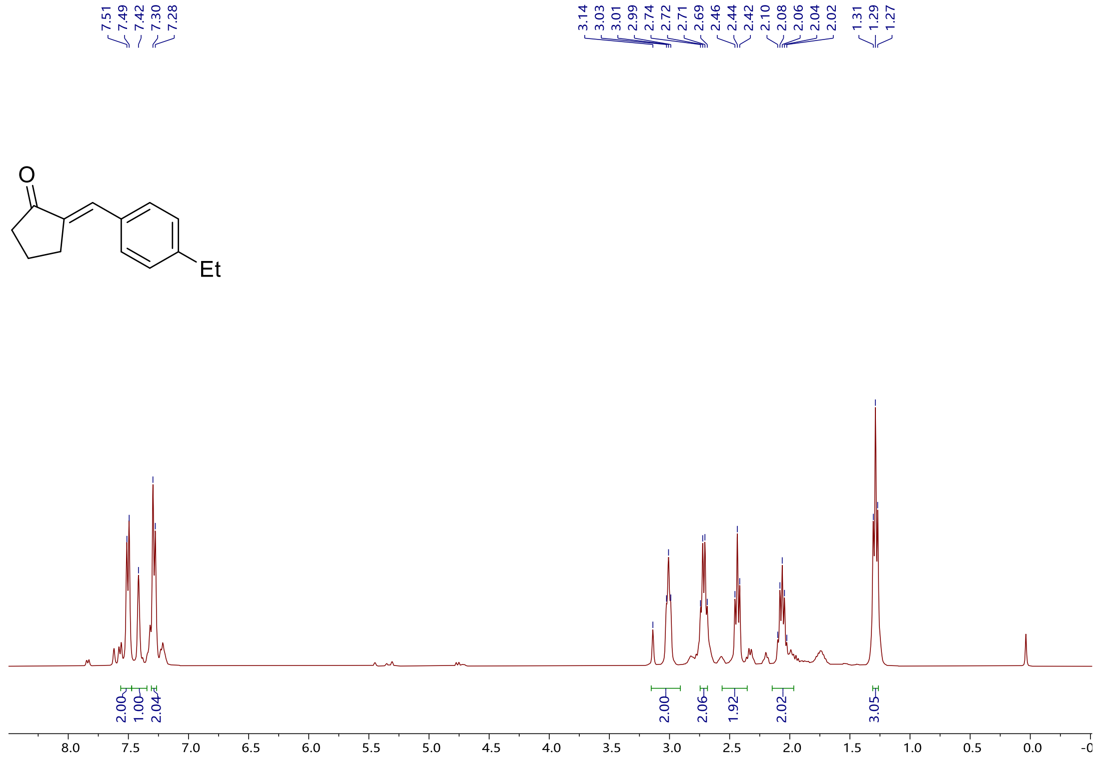

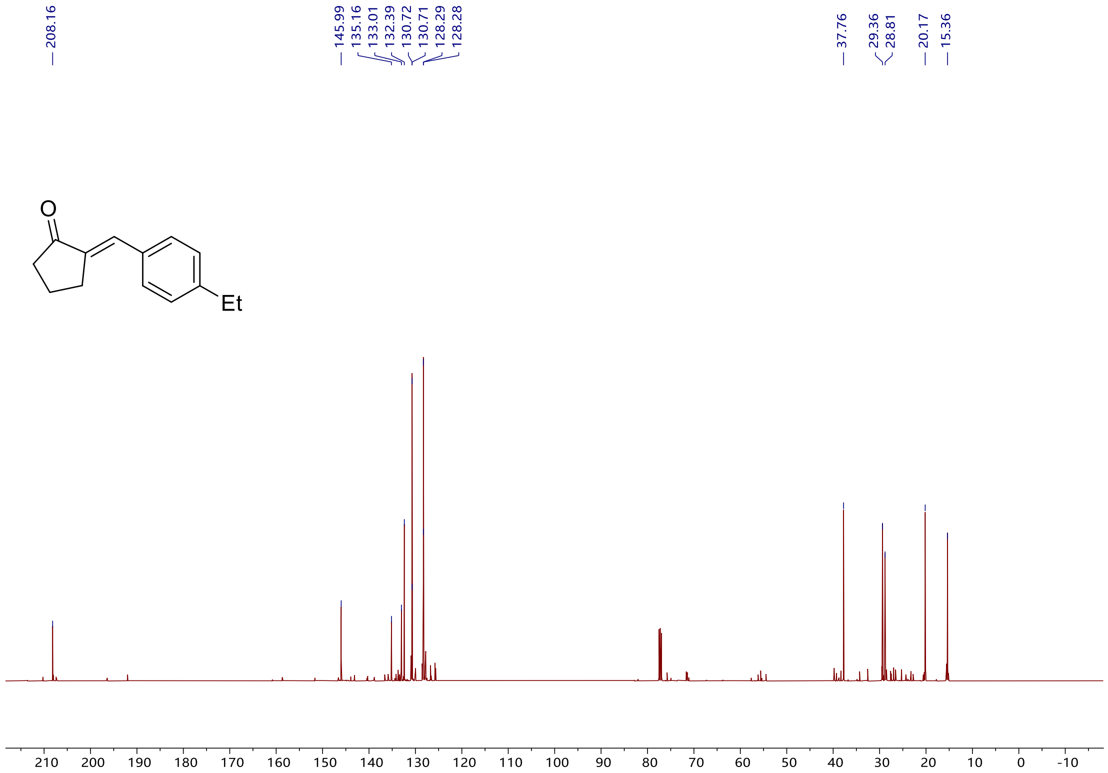

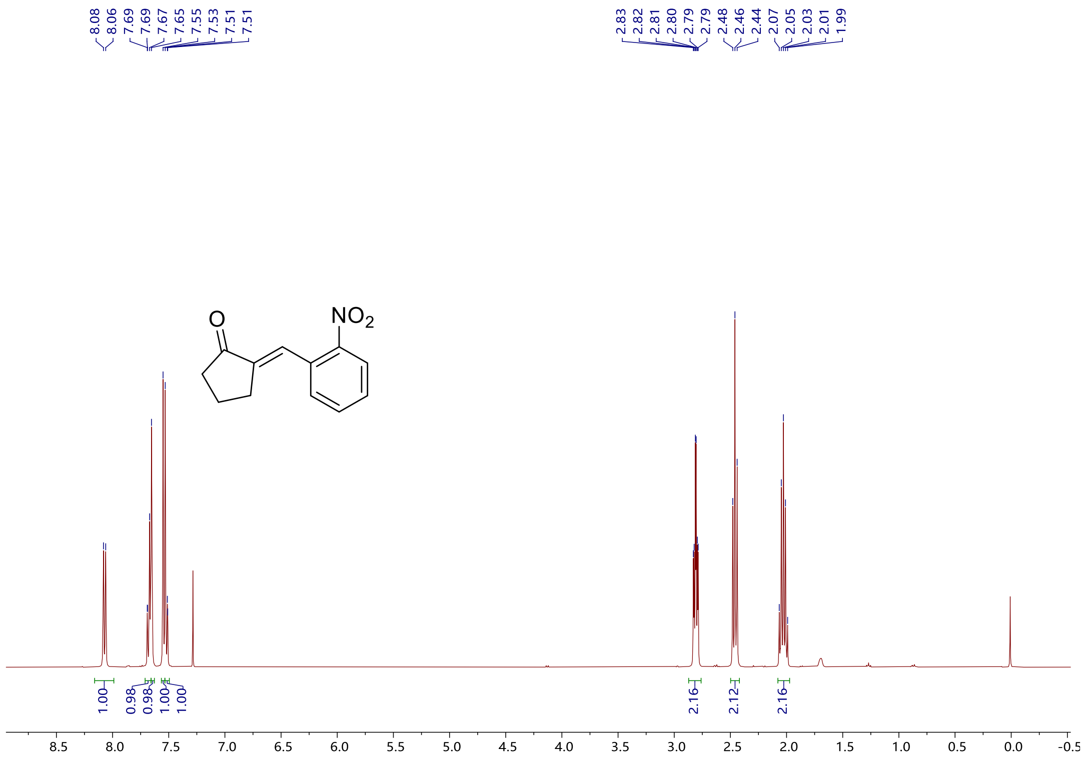
8.08
8.06
7.69
7.69
7.67
7.65
7.55
7.53
7.51
7.51
2.83
2.82
2.81
2.80
2.79
2.79
2.48
2.46
2.44
2.07
2.05
2.03
2.01
1.99
NO2
O
1.00
0.98
0.98
1.00
1.00
2.16
2.12
2.16

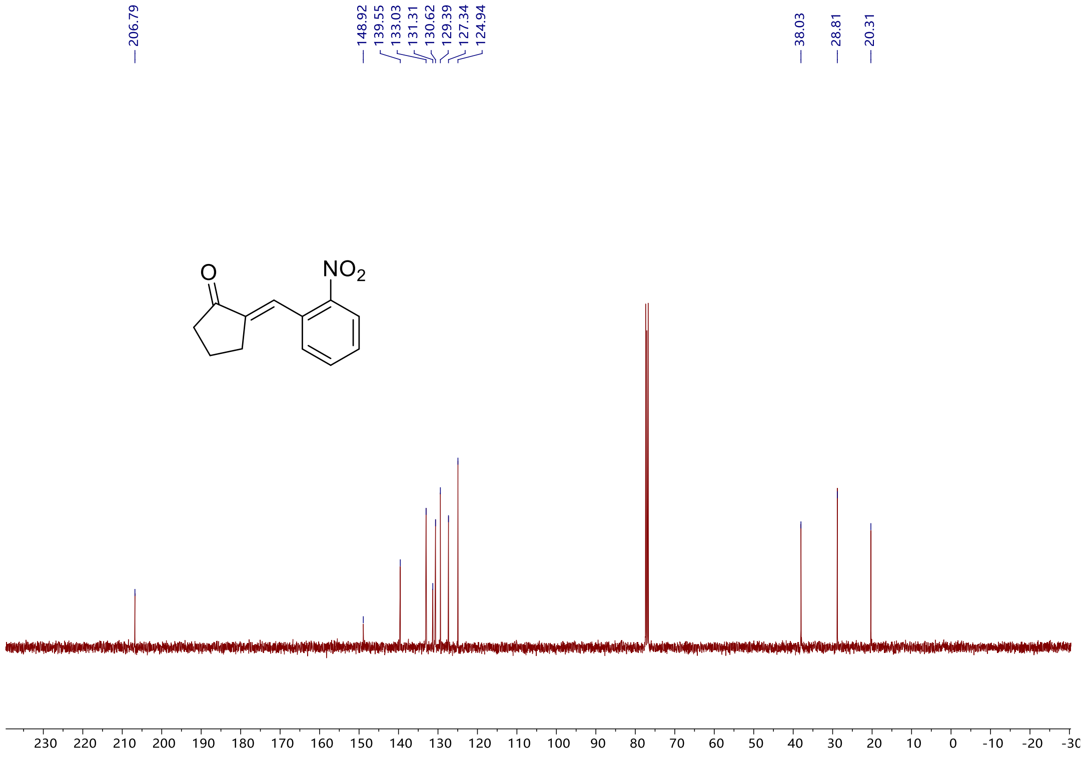
206.79
148.92
139.55
133.03
131.31
130.62
129.39
127.34
124.94
38.03
28.81
20.31
NO2
O

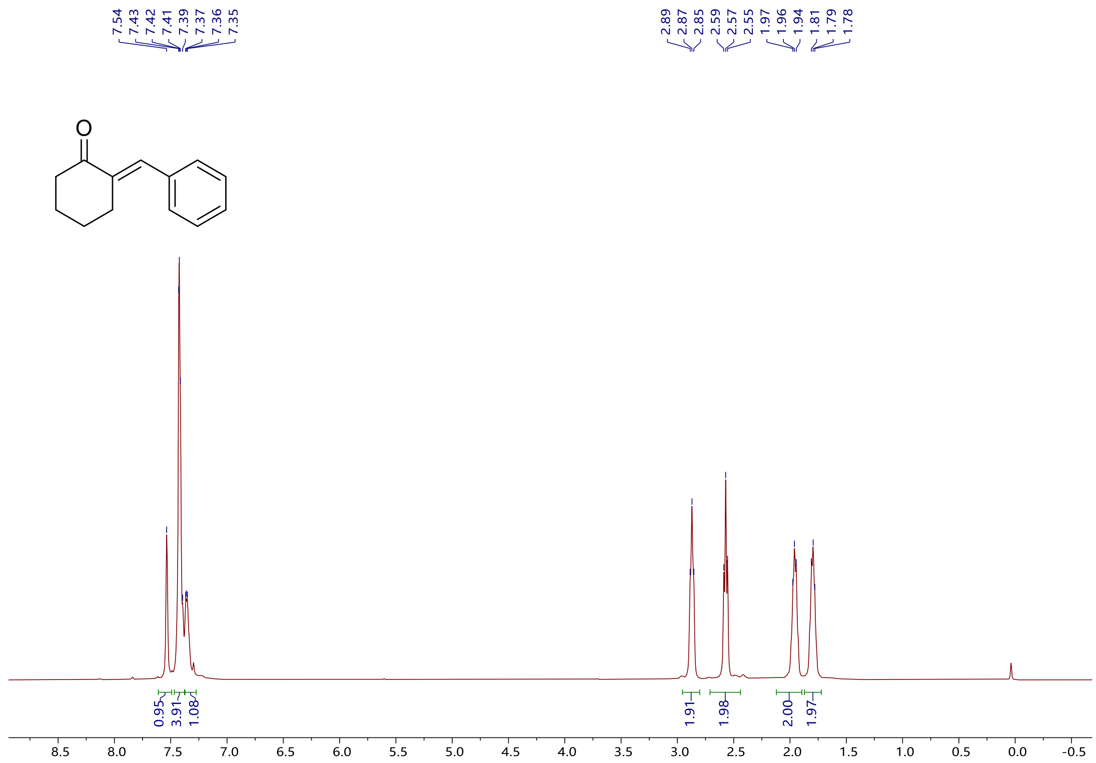
7.54
7.43
7.42
7.41
7.39
7.37
7.36
7.35
2.89
2.87
2.85
2.59
2.57
2.55
1.97
1.96
1.94
1.81
1.79
1.78
O
0.95
3.91
1.08
1.91
1.98
2.00
1.97
8.5
8.0
7.5
7.0
6.5
6.0
5.5
5.0
4.5
4.0
3.5
3.0
2.5
2.0
1.5
1.0
0.5
0.0
-0.5

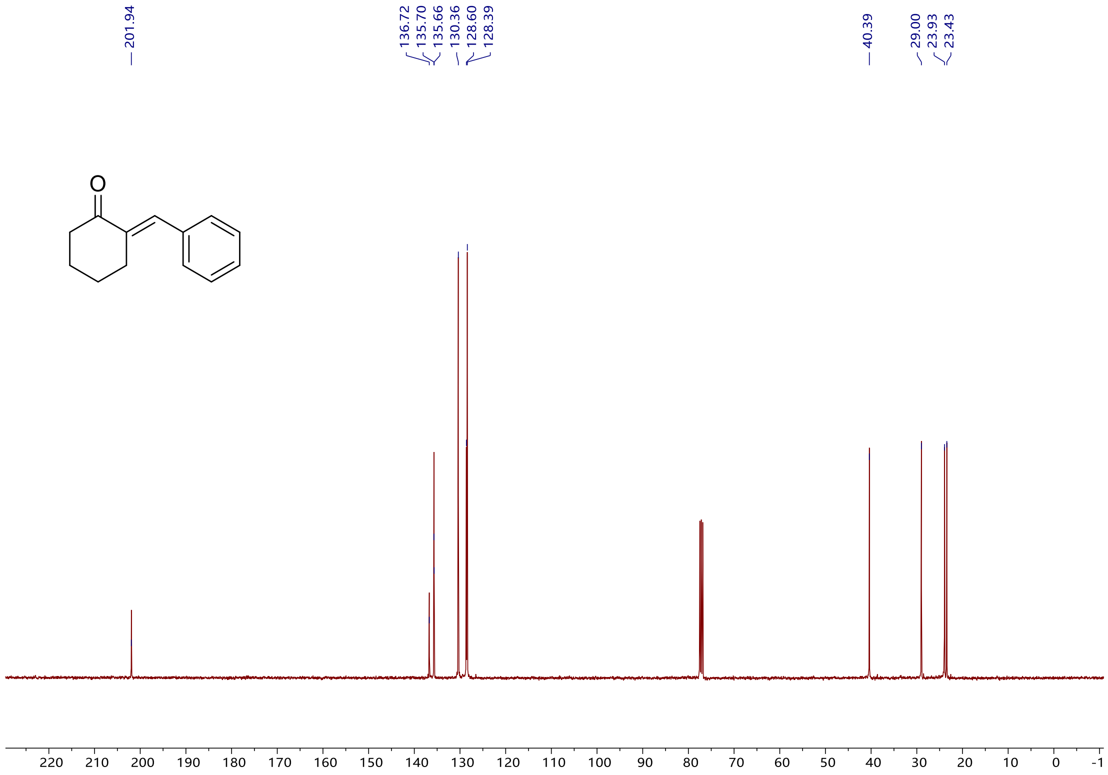
201.94
136.72
135.70
135.66
130.36
128.60
128.39
40.39
29.00
23.93
23.43
O
220
210
200
190
180
170
160
150
140
130
120
110
100
90
80
70
60
50
40
30
20
10
0
-1

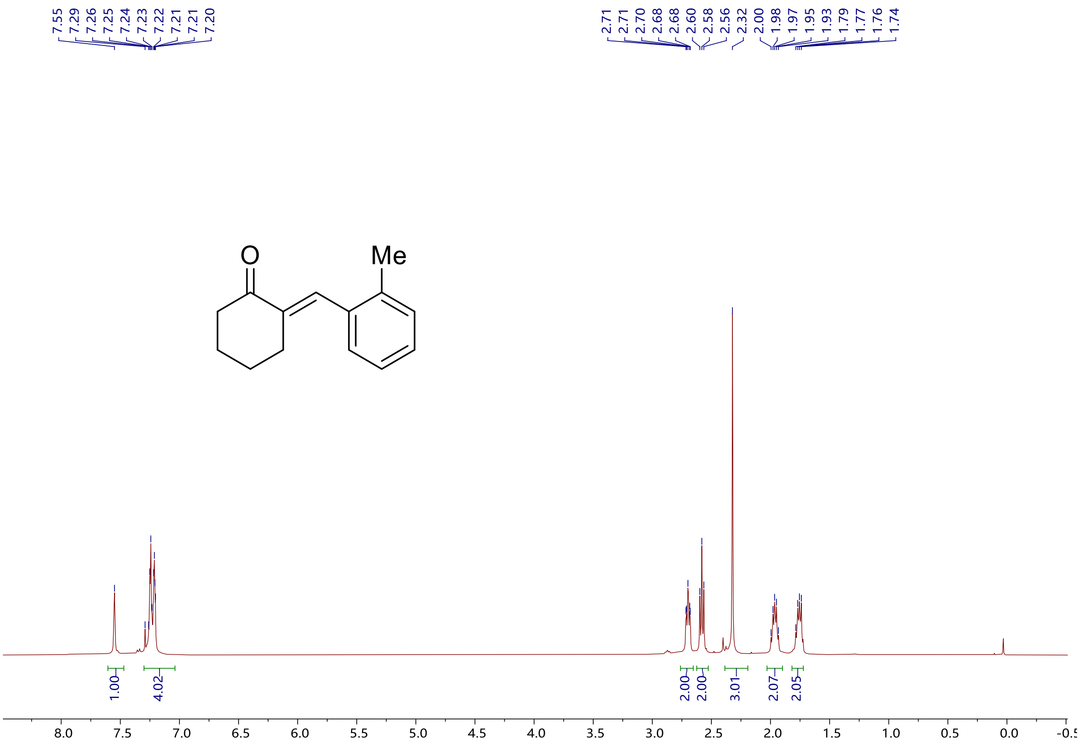
7.55
7.29
7.26
7.25
7.24
7.23
7.22
7.21
7.21
7.20
2.71
2.71
2.70
2.68
2.68
2.60
2.58
2.56
2.32
2.00
1.98
1.97
1.95
1.93
1.79
1.77
1.76
1.74
O
Me
1.00
4.02
2.00
2.00
3.01
2.07
2.05
8.0
7.5
7.0
6.5
6.0
5.5
5.0
4.5
4.0
3.5
3.0
2.5
2.0
1.5
1.0
0.5
0.0

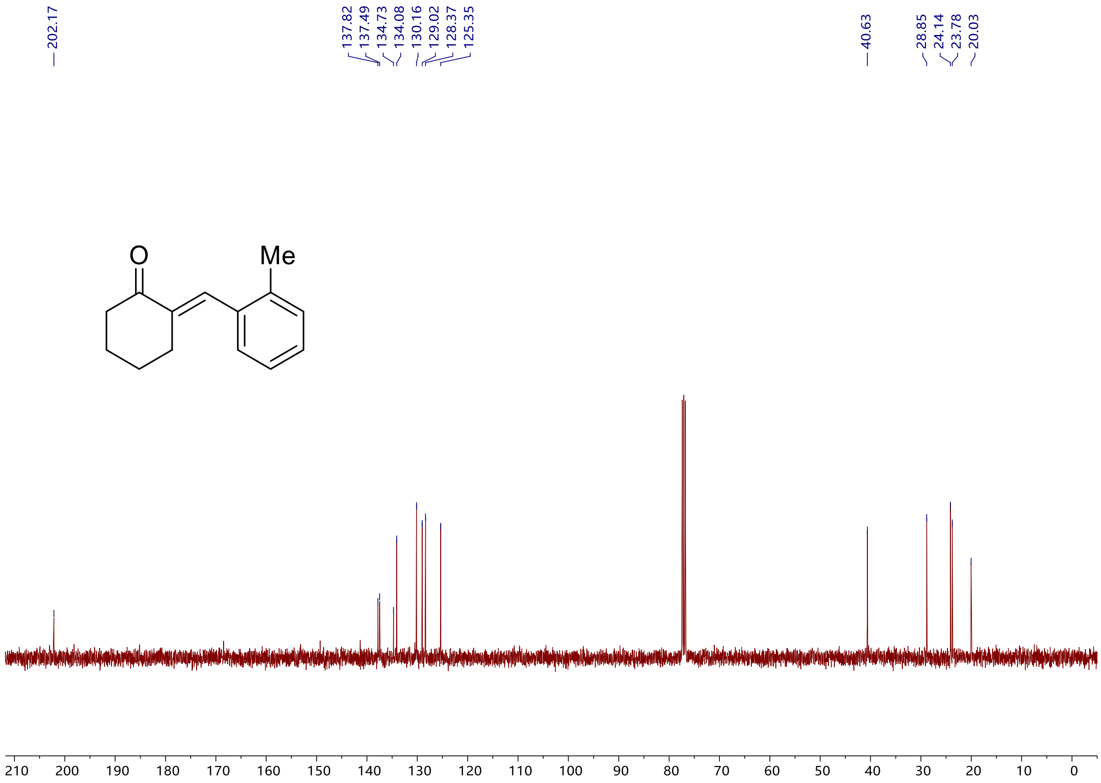
202.17
137.82
137.49
134.73
134.08
130.16
129.02
128.37
125.35
40.63
28.85
24.14
23.78
20.03
O
Me
210
200
190
180
170
160
150
140
130
120
110
100
90
80
70
60
50
40
30
20
10
0

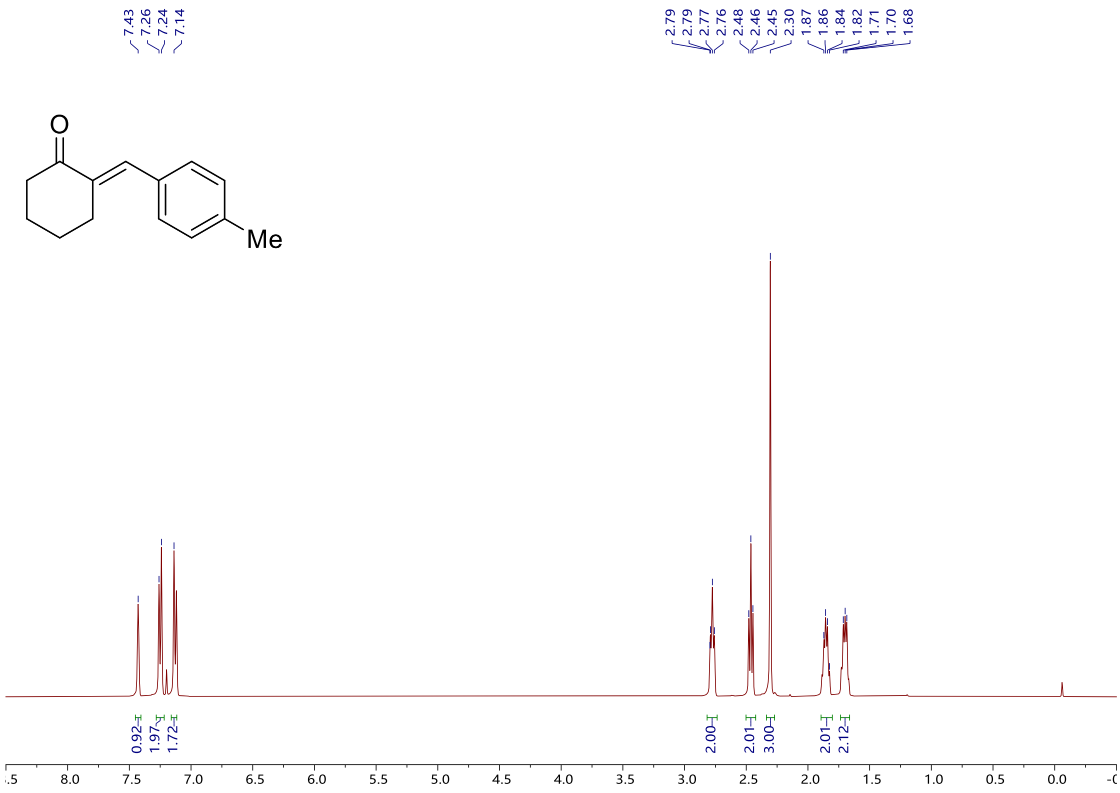
7.43
7.26
7.24
7.14
2.79
2.79
2.77
2.76
2.48
2.46
2.45
2.30
1.87
1.86
1.84
1.82
1.71
1.70
1.68
O
Me
0.92
1.97
1.72
2.00
2.01
3.00
2.01
2.12

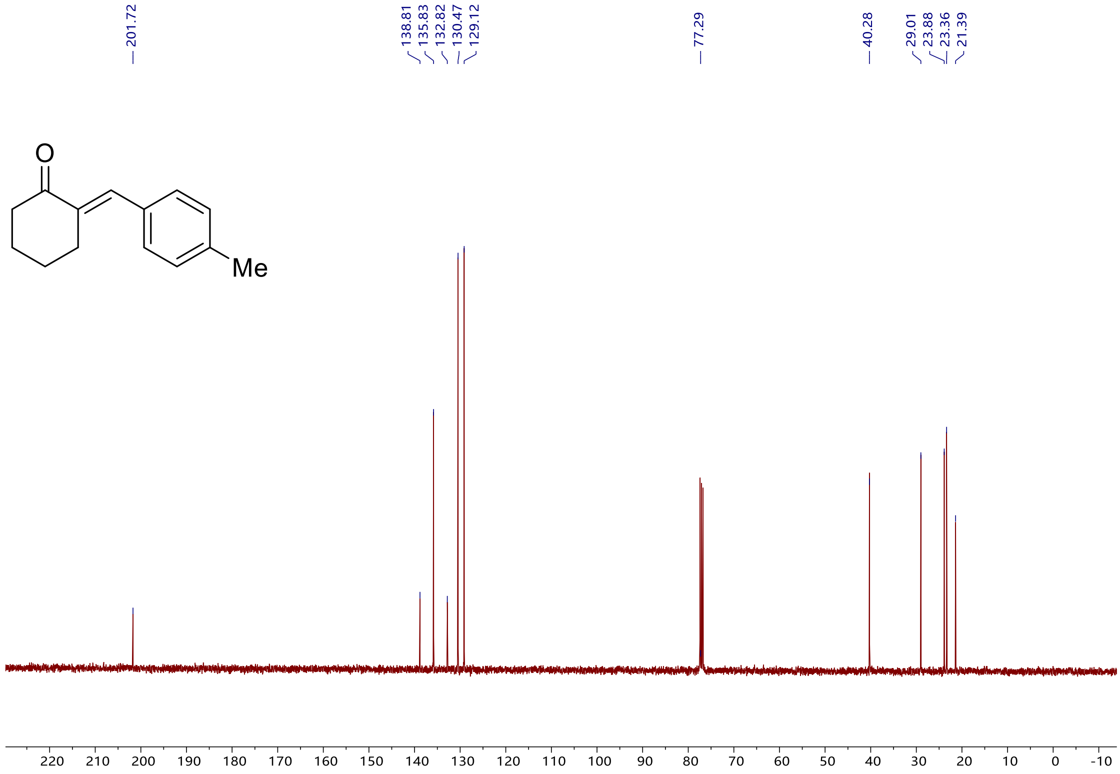
201.72
138.81
135.83
132.82
130.47
129.12
77.29
40.28
29.01
23.88
23.36
21.39
O
Me

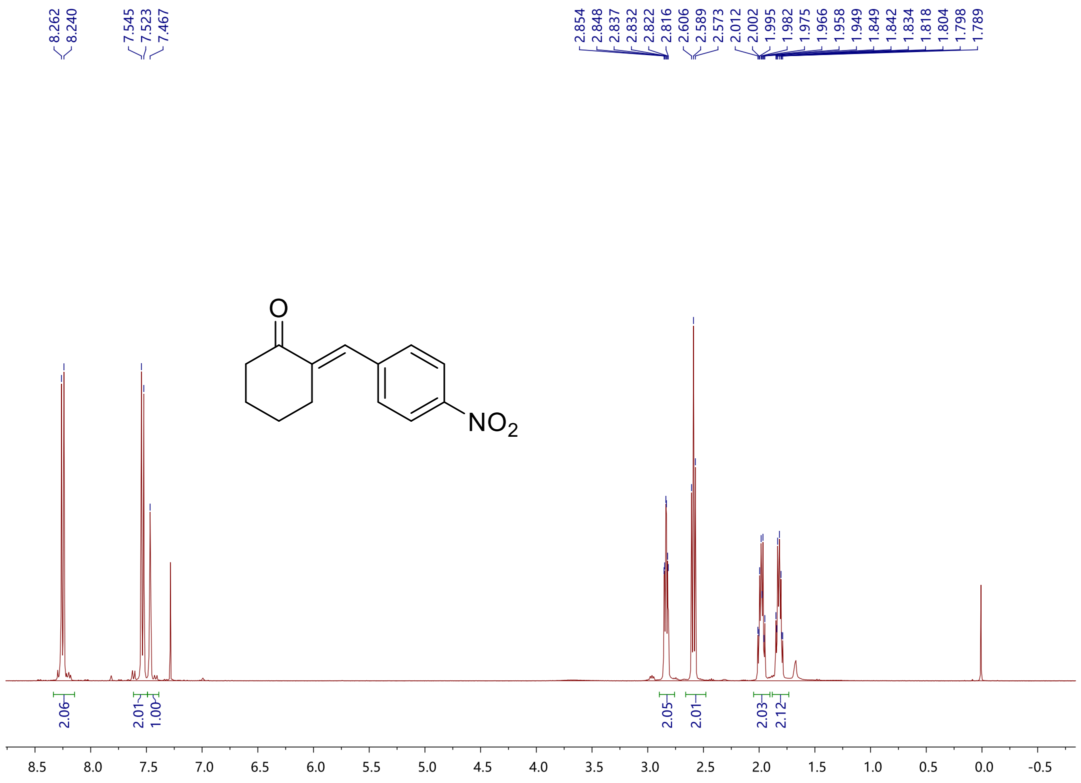
8.262
8.240
7.545
7.523
7.467
2.854
2.848
2.837
2.832
2.822
2.816
2.606
2.589
2.573
2.012
2.002
1.995
1.982
1.975
1.966
1.958
1.949
1.849
1.842
1.834
1.818
1.804
1.798
1.789
O
NO2
2.06
2.01
1.00
2.05
2.01
2.03
2.12
8.5 8.0 7.5 7.0 6.5 6.0 5.5 5.0 4.5 4.0 3.5 3.0 2.5 2.0 1.5 1.0 0.5 0.0 -0.5

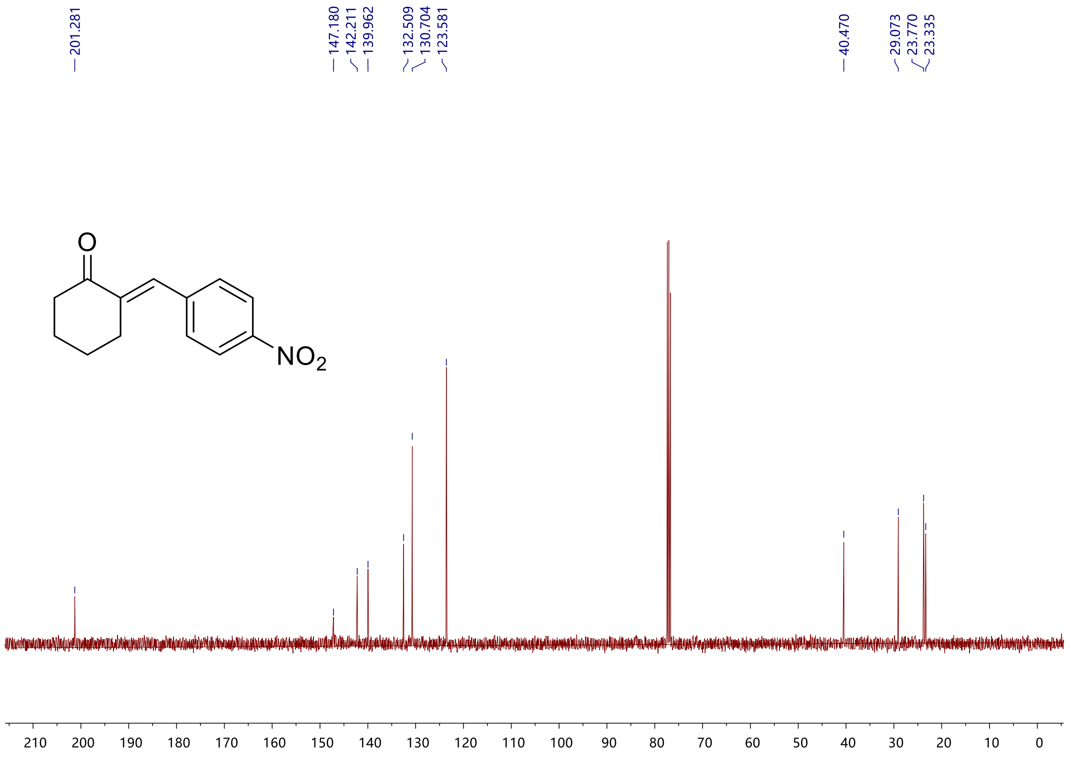
201.281
147.180
142.211
139.962
132.509
130.704
123.581
40.470
29.073
23.770
23.335
O
NO2
210 200 190 180 170 160 150 140 130 120 110 100 90 80 70 60 50 40 30 20 10 0

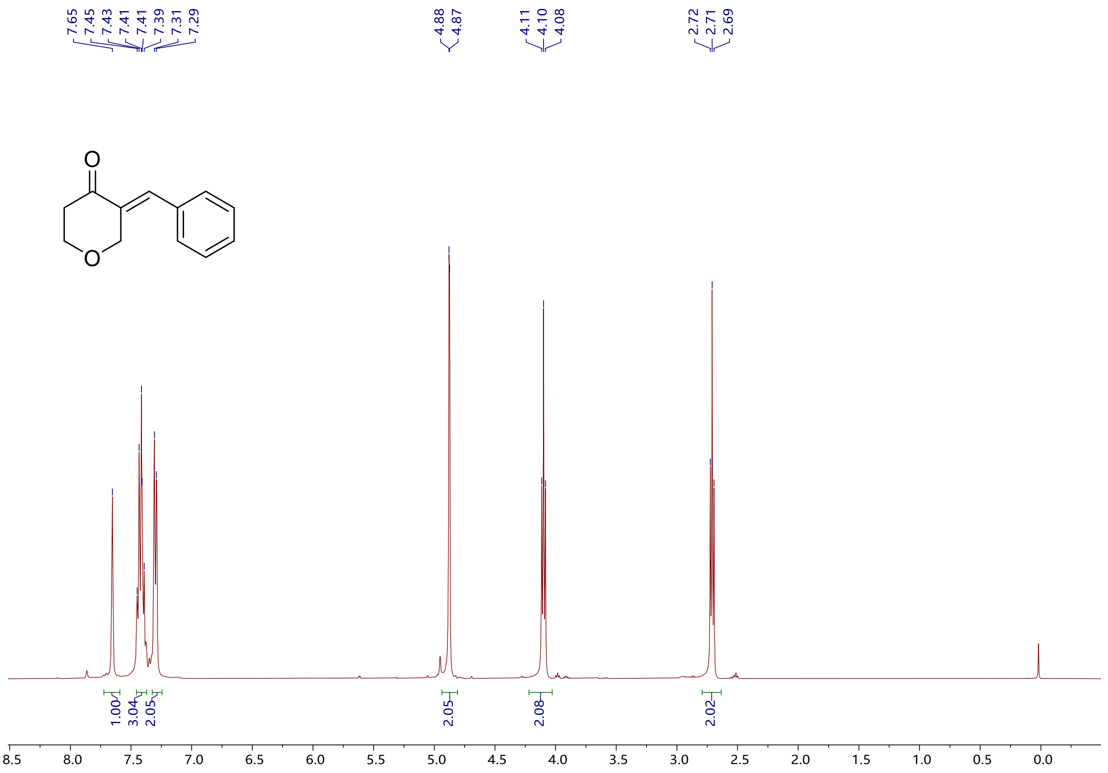
7.65
7.45
7.43
7.41
7.41
7.39
7.31
7.29
4.88
4.87
4.11
4.10
4.08
2.72
2.71
2.69
O
O
1.00
3.04
2.05
2.05
2.08
2.02
8.5
8.0
7.5
7.0
6.5
6.0
5.5
5.0
4.5
4.0
3.5
3.0
2.5
2.0
1.5
1.0
0.5
0.0

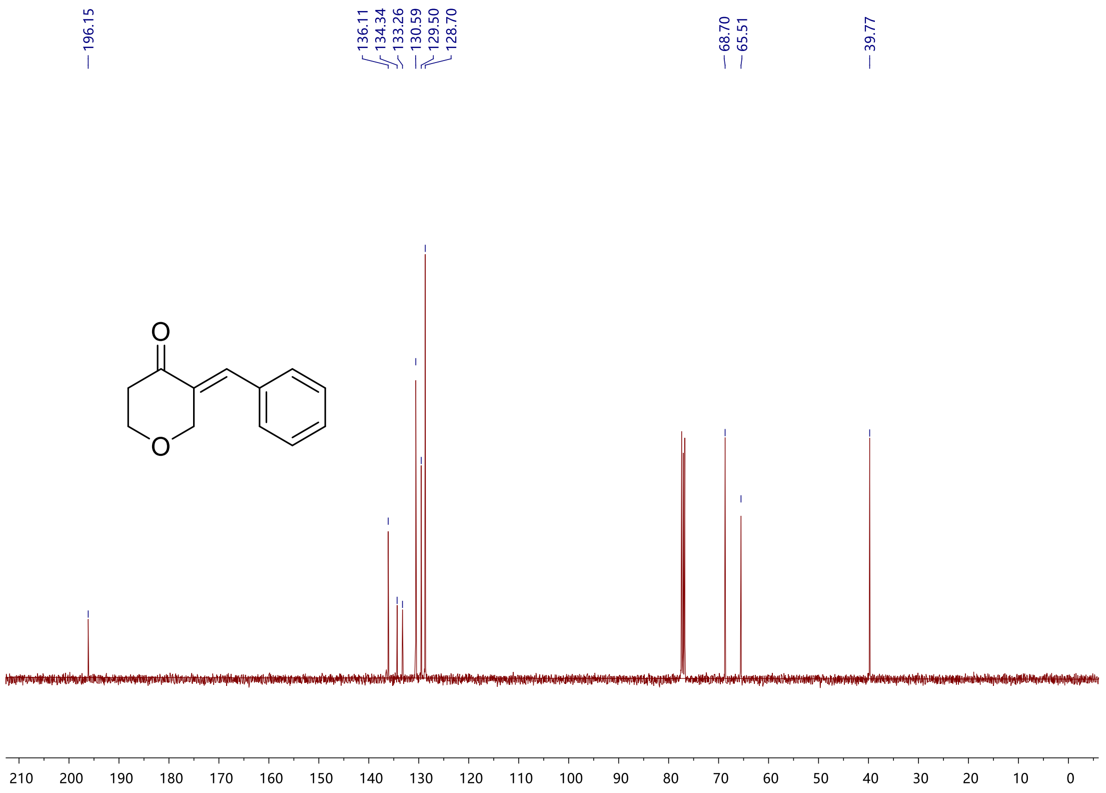
196.15
136.11
134.34
133.26
130.59
129.50
128.70
68.70
65.51
39.77
O
O
210
200
190
180
170
160
150
140
130
120
110
100
90
80
70
60
50
40
30
20
10
0

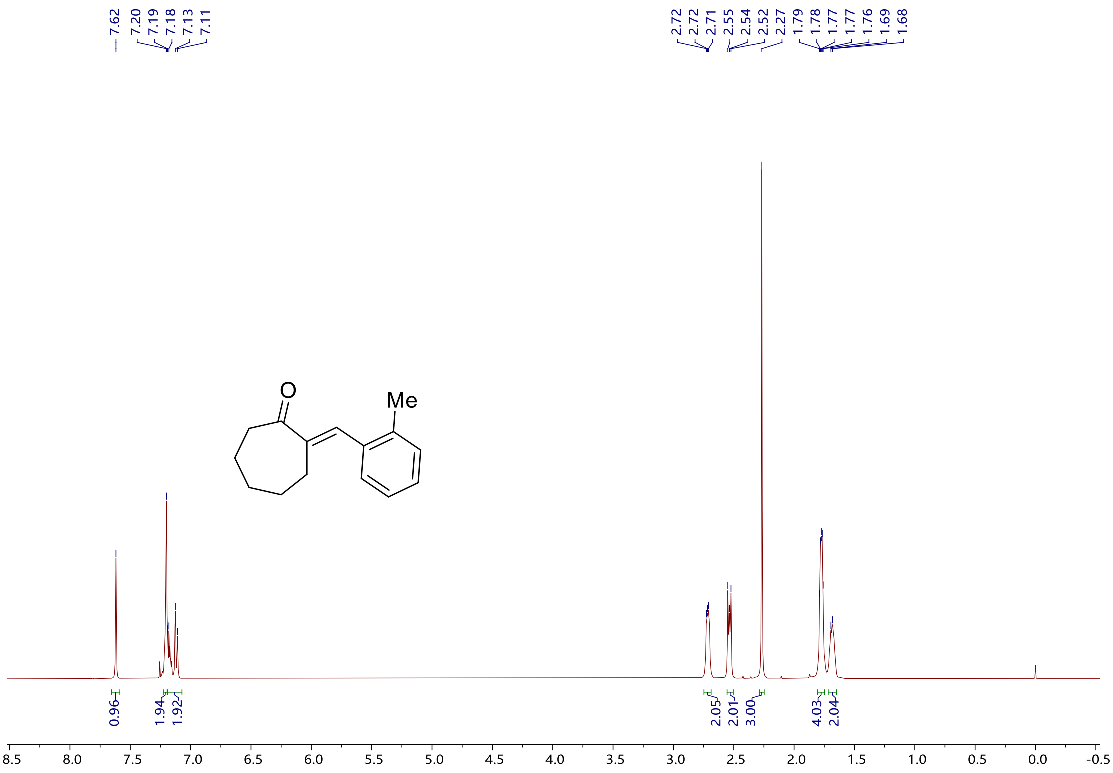
7.62
7.20
7.19
7.18
7.13
7.11
2.72
2.72
2.71
2.55
2.54
2.52
2.27
1.79
1.78
1.77
1.77
1.76
1.69
1.68
O
Me
0.96
1.94
1.92
2.05
2.01
3.00
4.03
2.04
8.5
8.0
7.5
7.0
6.5
6.0
5.5
5.0
4.5
4.0
3.5
3.0
2.5
2.0
1.5
1.0
0.5
0.0
-0.5

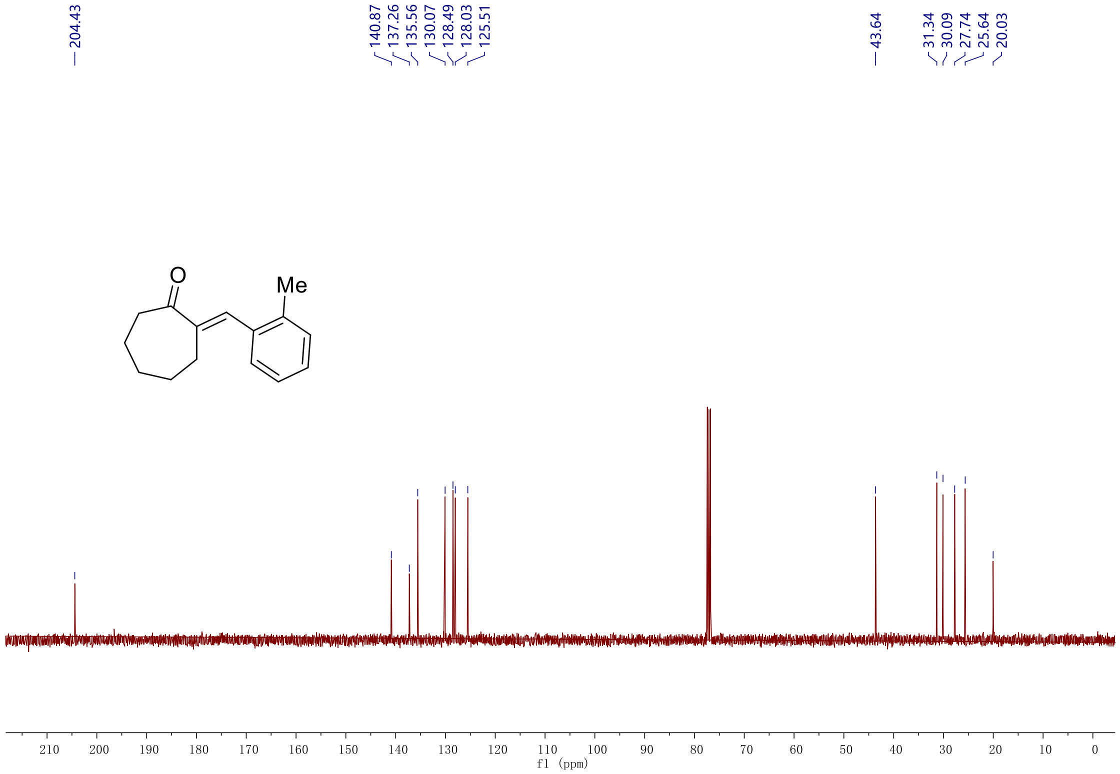
204.43
140.87
137.26
135.56
130.07
128.49
128.03
125.51
43.64
31.34
30.09
27.74
25.64
20.03
O
Me
210
200
190
180
170
160
150
140
130
120
110
100
90
80
70
60
50
40
30
20
10
0
f1 (ppm)

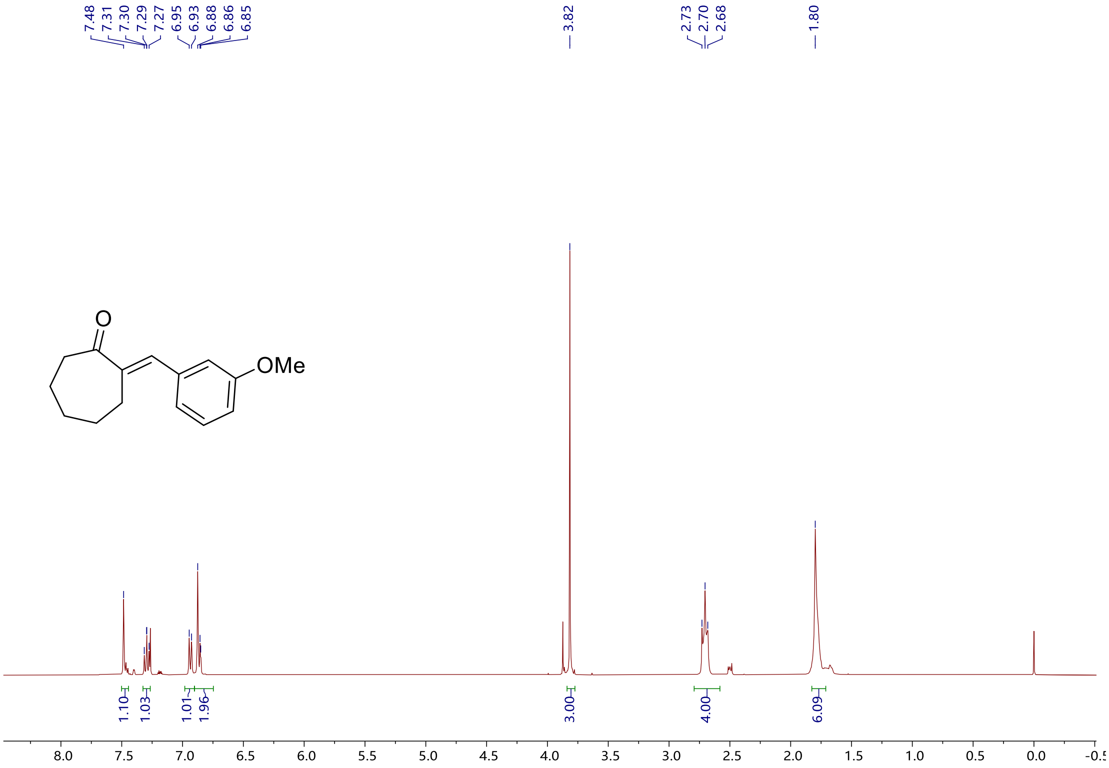
7.48
7.31
7.30
7.29
7.27
6.95
6.93
6.88
6.86
6.85
3.82
2.73
2.70
2.68
1.80
OMe
O
1.10
1.03
1.01
1.96
3.00
4.00
6.09
8.0
7.5
7.0
6.5
6.0
5.5
5.0
4.5
4.0
3.5
3.0
2.5
2.0
1.5
1.0
0.5
0.0

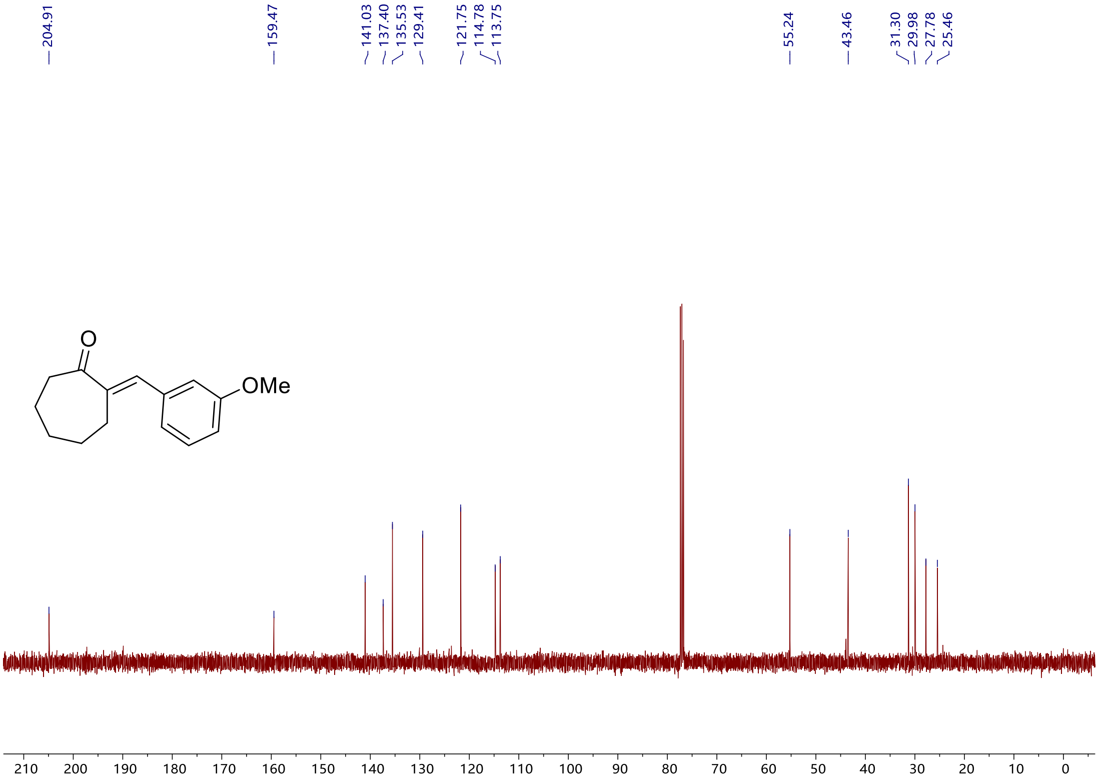
204.91
159.47
141.03
137.40
135.53
129.41
121.75
114.78
113.75
55.24
43.46
31.30
29.98
27.78
25.46
O
OMe
210
200
190
180
170
160
150
140
130
120
110
100
90
80
70
60
50
40
30
20
10
0

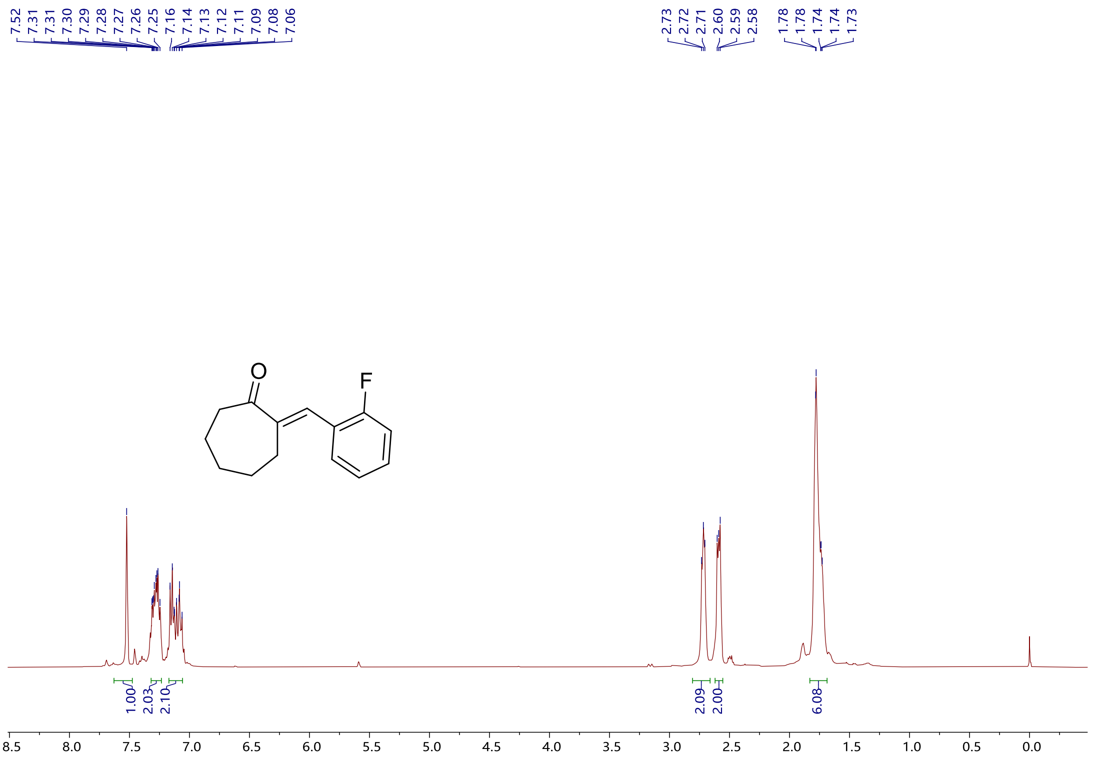
7.52 7.31 7.31 7.30 7.29 7.28 7.27 7.26 7.25 7.16 7.14 7.13 7.12 7.11 7.09 7.08 7.06
2.73 2.72 2.71 2.60 2.59 2.58
1.78 1.78 1.74 1.74 1.73
O
F
1.00 2.03 2.10
2.09 2.00
6.08
8.5 8.0 7.5 7.0 6.5 6.0 5.5 5.0 4.5 4.0 3.5 3.0 2.5 2.0 1.5 1.0 0.5 0.0

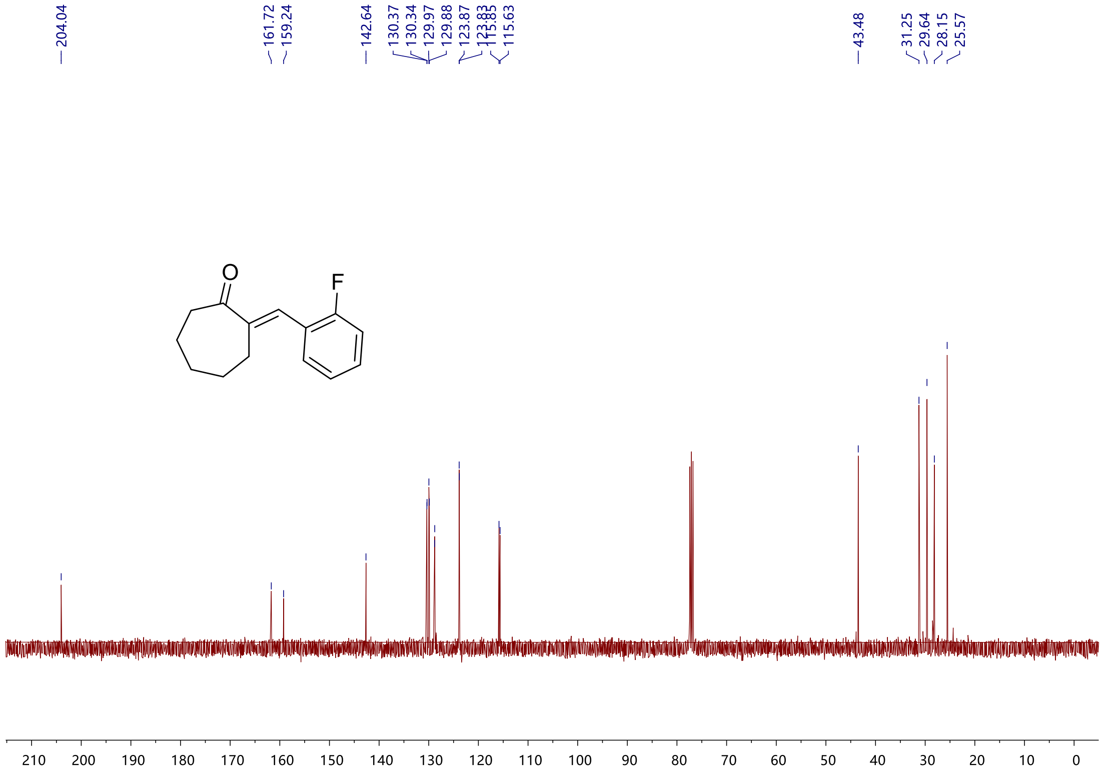
204.04
161.72 159.24
142.64
130.37 130.34 129.97 129.88 123.87 123.83 115.85 115.63
43.48
31.25 29.64 28.15 25.57
O
F
210 200 190 180 170 160 150 140 130 120 110 100 90 80 70 60 50 40 30 20 10 0

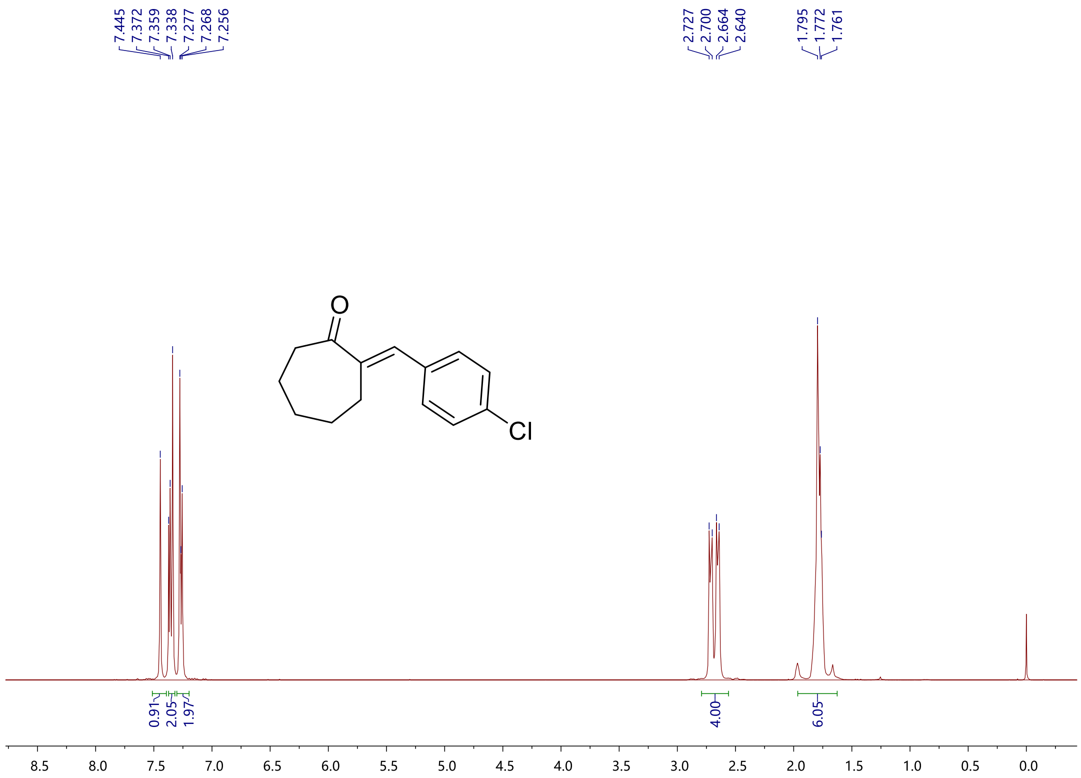

7.445
7.372
7.359
7.338
7.277
7.268
7.256
2.727
2.700
2.664
2.640
1.795
1.772
1.761
O
Cl
0.91
2.05
1.97
4.00
6.05
8.5 8.0 7.5 7.0 6.5 6.0 5.5 5.0 4.5 4.0 3.5 3.0 2.5 2.0 1.5 1.0 0.5 0.0

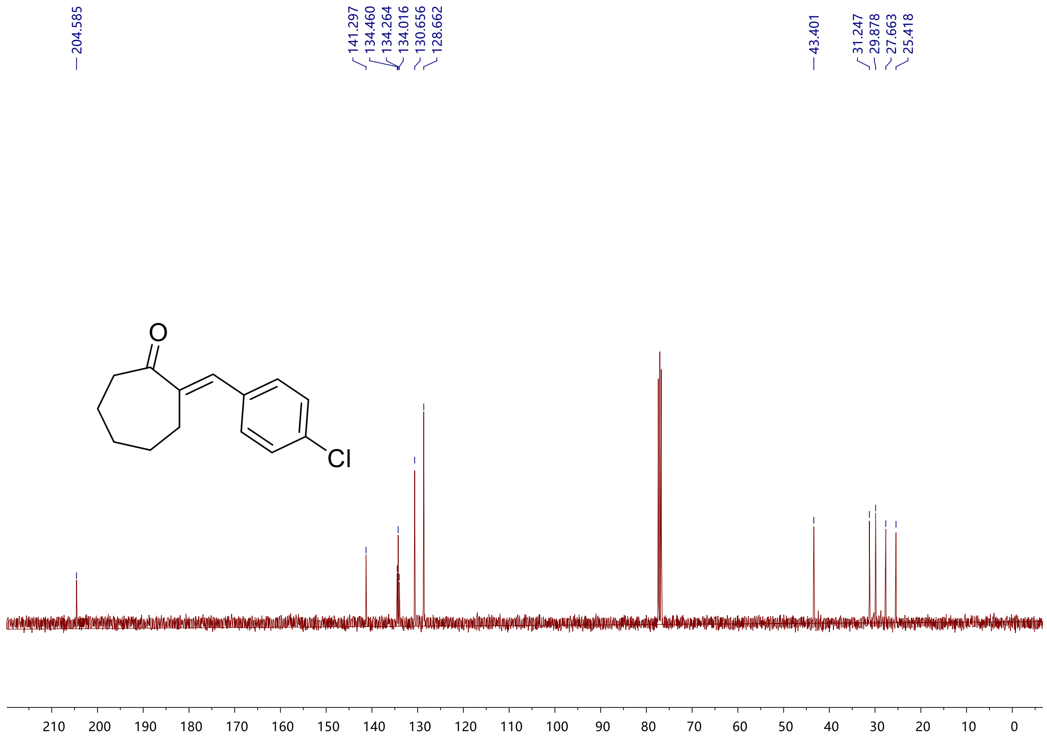

204.585
141.297
134.460
134.264
134.016
130.656
128.662
43.401
31.247
29.878
27.663
25.418
O
Cl
210 200 190 180 170 160 150 140 130 120 110 100 90 80 70 60 50 40 30 20 10 0

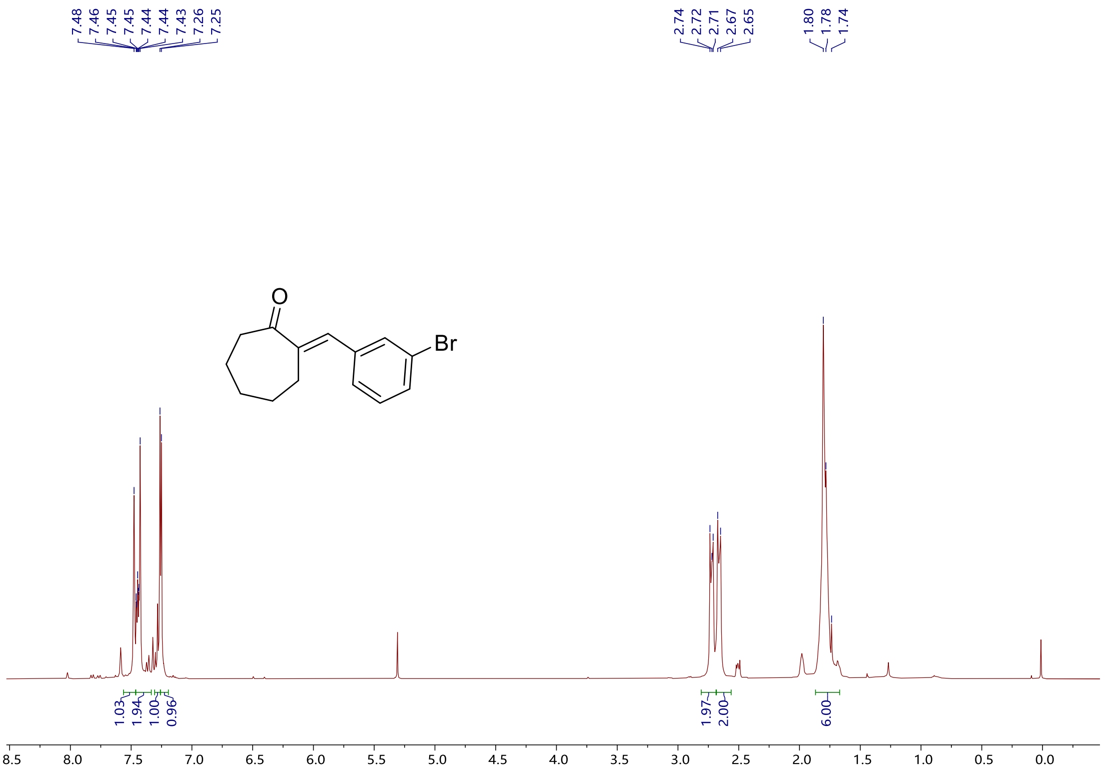
7.48
7.46
7.45
7.45
7.44
7.44
7.43
7.26
7.25
2.74
2.72
2.71
2.67
2.65
1.80
1.78
1.74
Br
1.03
1.94
1.00
0.96
1.97
2.00
6.00
8.5 8.0 7.5 7.0 6.5 6.0 5.5 5.0 4.5 4.0 3.5 3.0 2.5 2.0 1.5 1.0 0.5 0.0

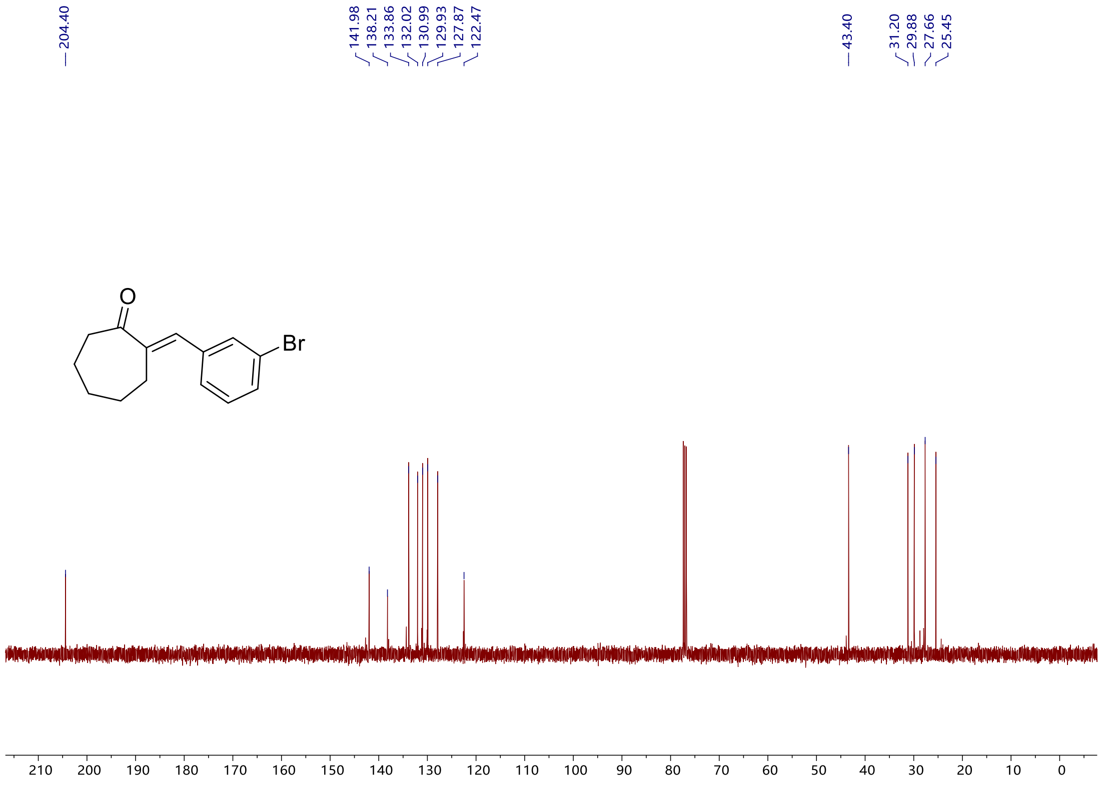
204.40
141.98
138.21
133.86
132.02
130.99
129.93
127.87
122.47
43.40
31.20
29.88
27.66
25.45
Br
210 200 190 180 170 160 150 140 130 120 110 100 90 80 70 60 50 40 30 20 10 0

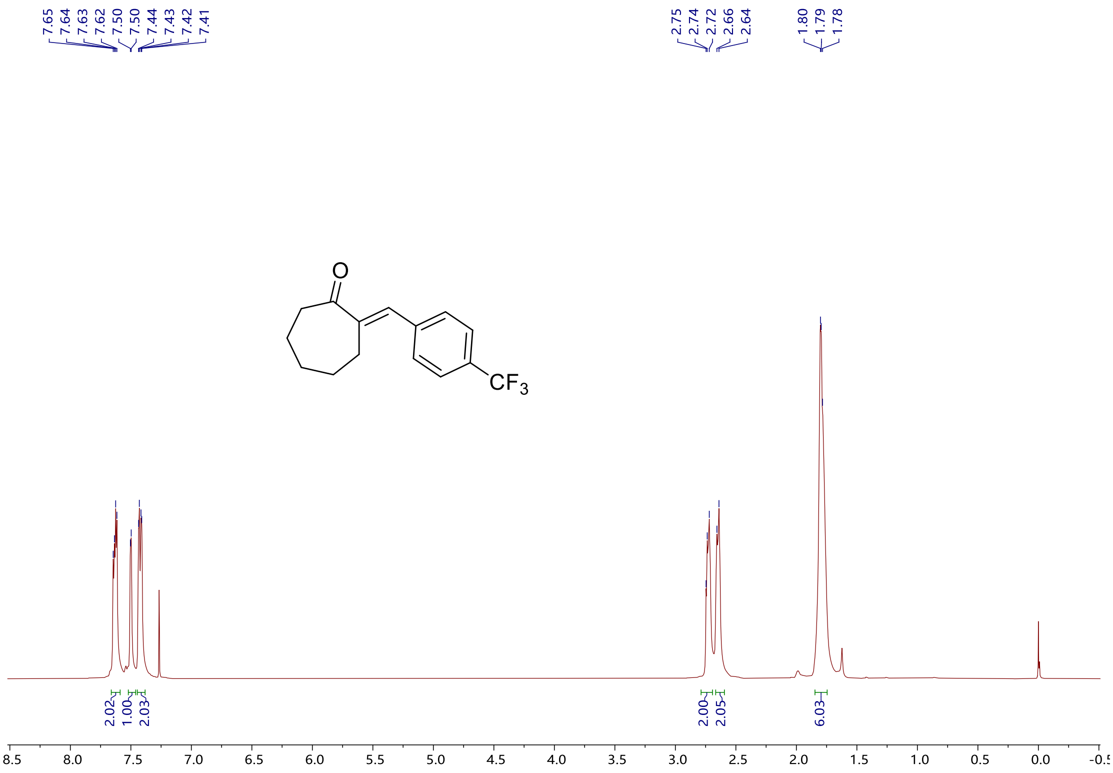
7.65
7.64
7.63
7.62
7.50
7.50
7.44
7.43
7.42
7.41
2.75
2.74
2.72
2.66
2.64
1.80
1.79
1.78
O
CF3
2.02
1.00
2.03
2.00
2.05
6.03
8.5 8.0 7.5 7.0 6.5 6.0 5.5 5.0 4.5 4.0 3.5 3.0 2.5 2.0 1.5 1.0 0.5 0.0

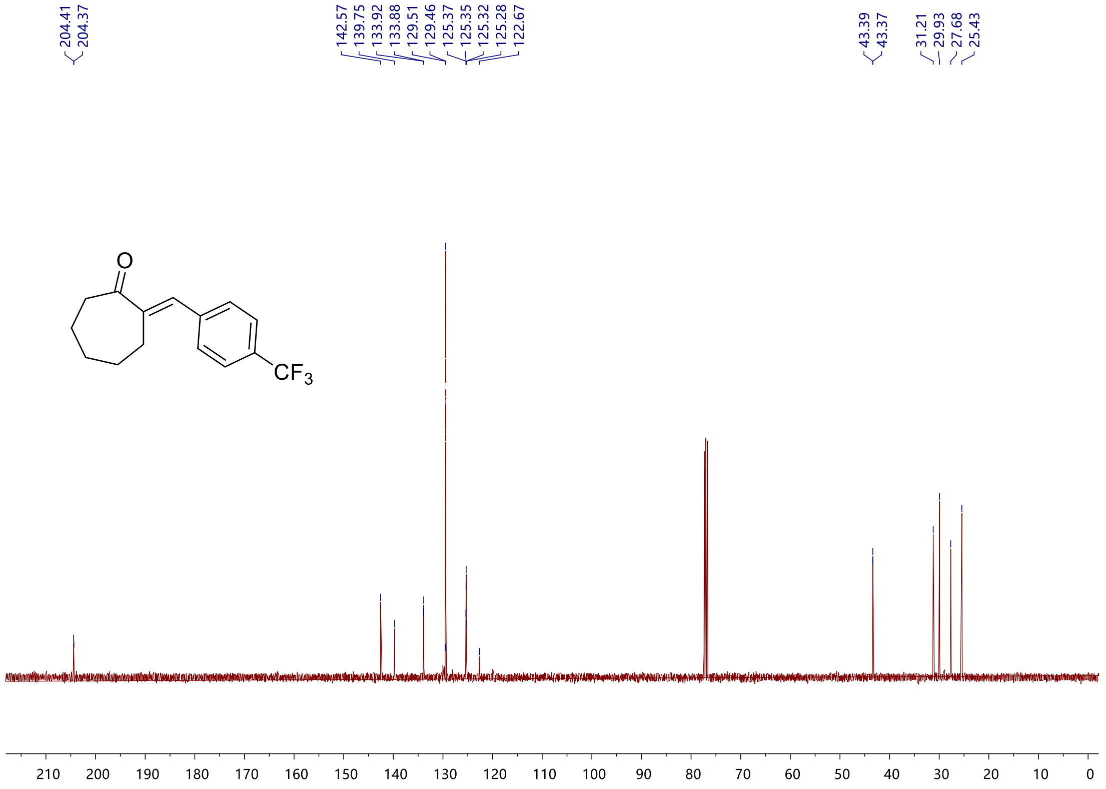
204.41
204.37
142.57
139.75
133.92
133.88
129.51
129.46
125.37
125.35
125.32
125.28
122.67
43.39
43.37
31.21
29.93
27.68
25.43
O
CF3
210 200 190 180 170 160 150 140 130 120 110 100 90 80 70 60 50 40 30 20 10 0

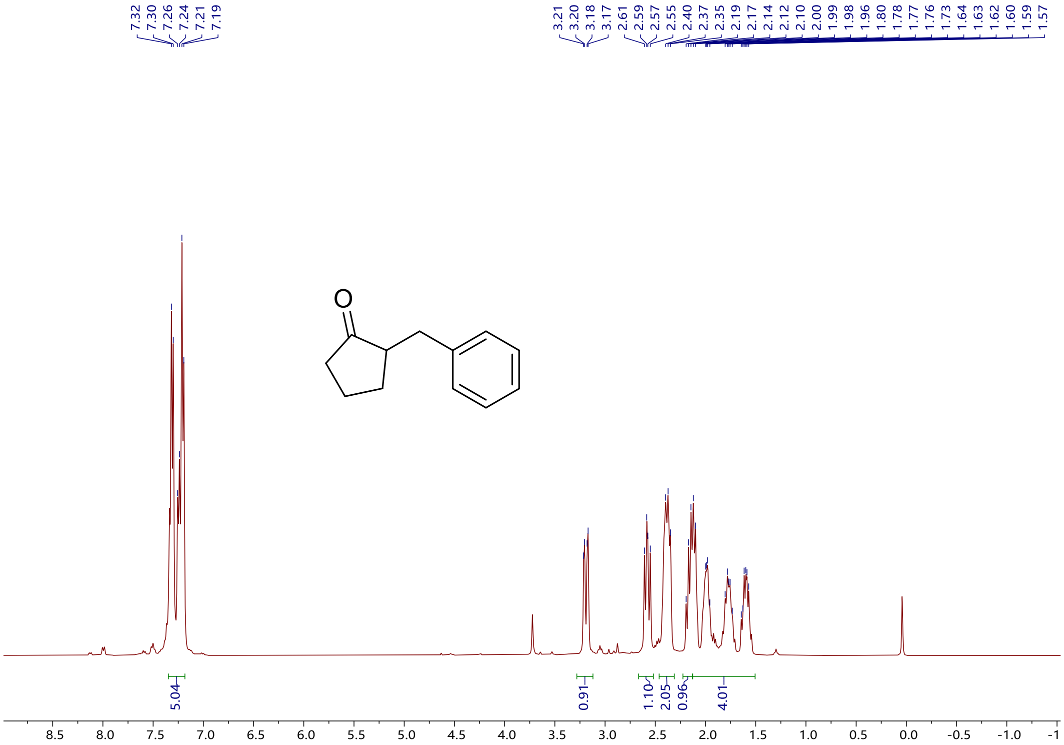

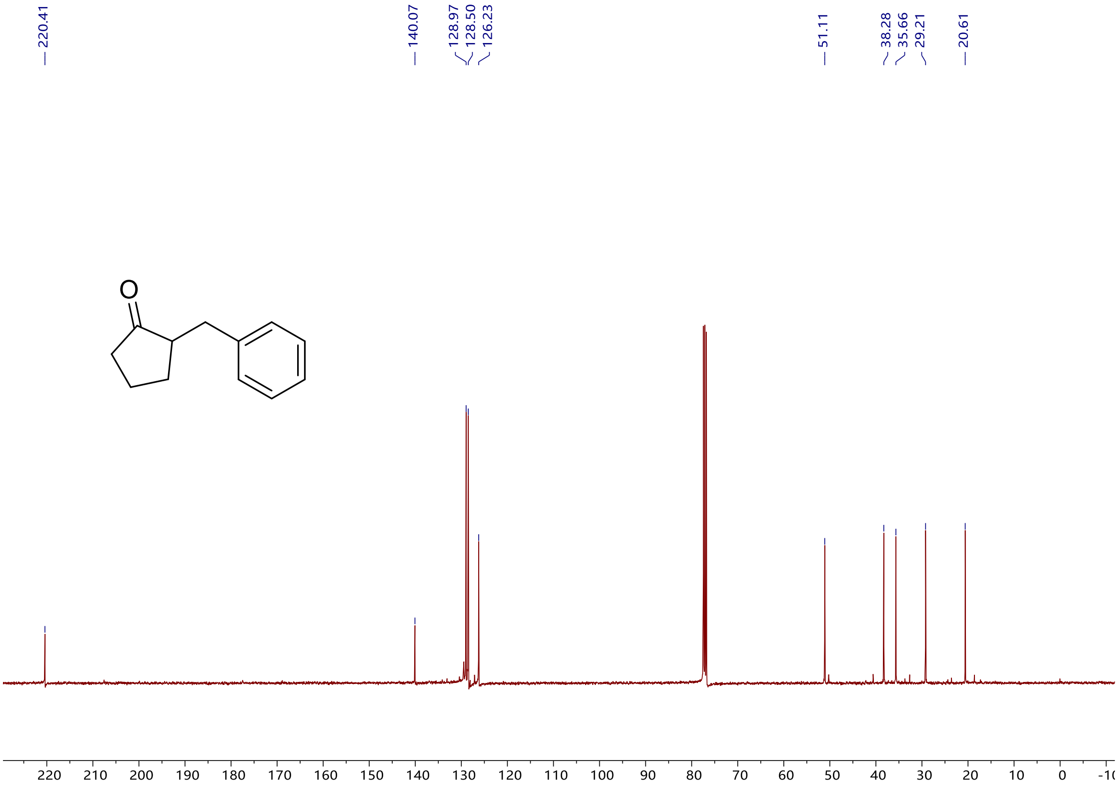

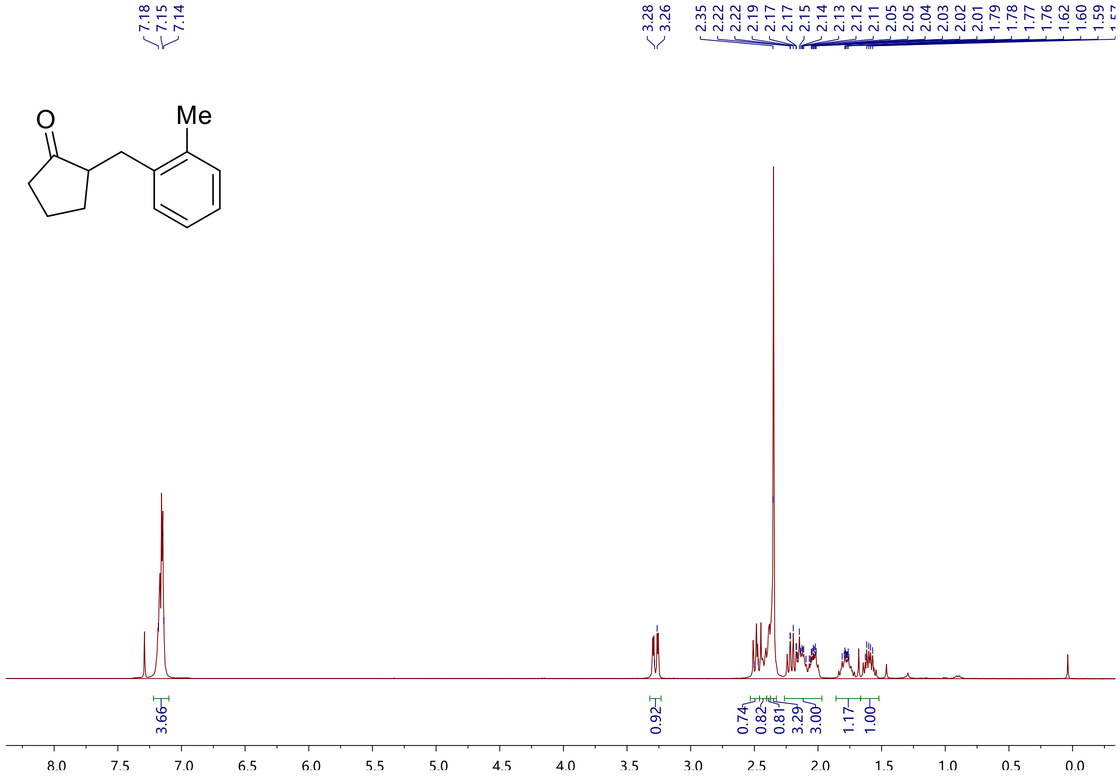
Me
O
7.18
7.15
7.14
3.28
3.26
3.66
0.92
8.0
7.5
7.0
6.5
6.0
5.5
5.0
4.5
4.0
3.5
3.0
2.5
2.0
1.5
1.0
0.5
0.0

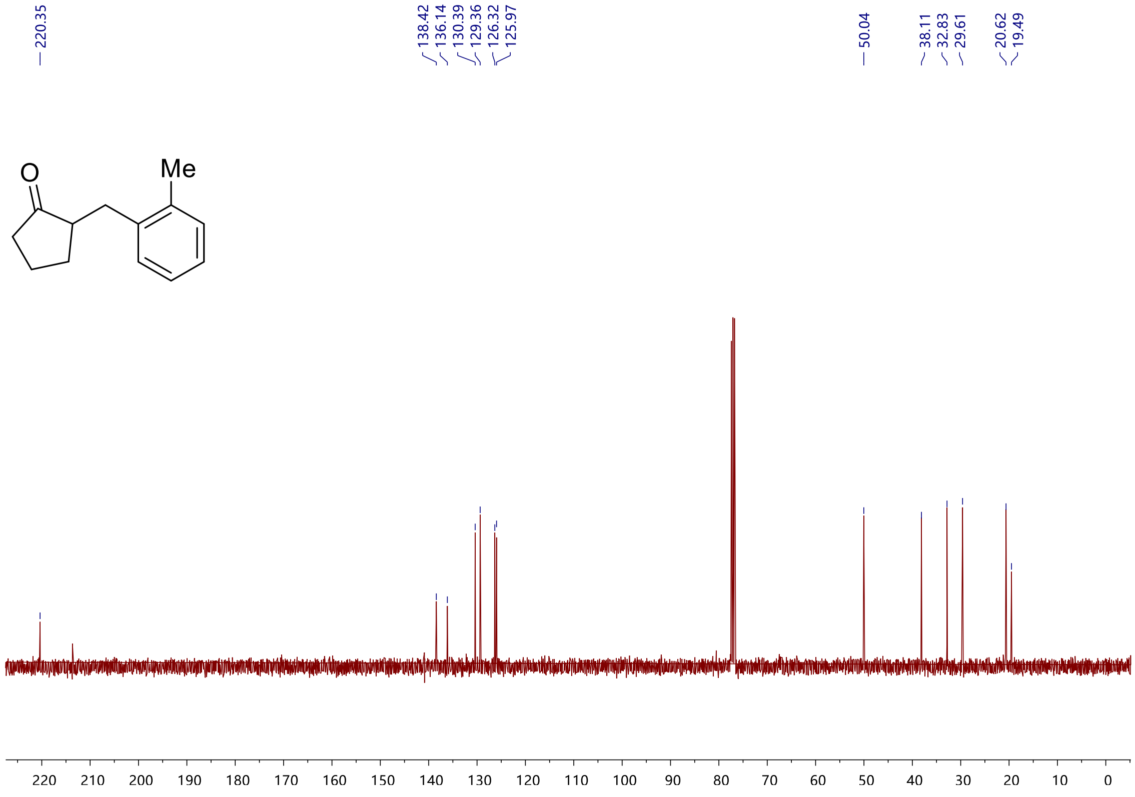
220.35
138.42
136.14
130.39
129.36
126.32
125.97
50.04
38.11
32.83
29.61
20.62
19.49
Me
O
220
210
200
190
180
170
160
150
140
130
120
110
100
90
80
70
60
50
40
30
20
10
0

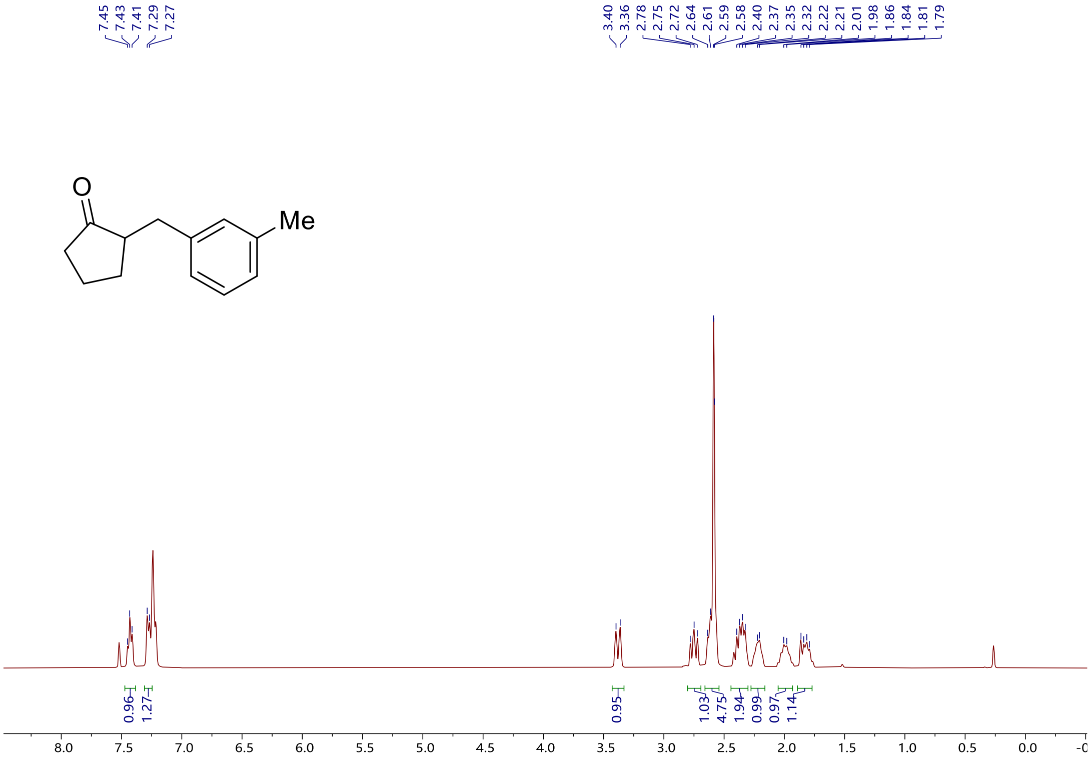

7.45
7.43
7.41
7.29
7.27
3.40
3.36
2.78
2.75
2.72
2.64
2.61
2.59
2.58
2.40
2.37
2.35
2.32
2.22
2.21
2.01
1.98
1.86
1.84
1.81
1.79
O
Me
0.96
1.27
0.95
1.03
4.75
1.94
0.99
0.97
1.14

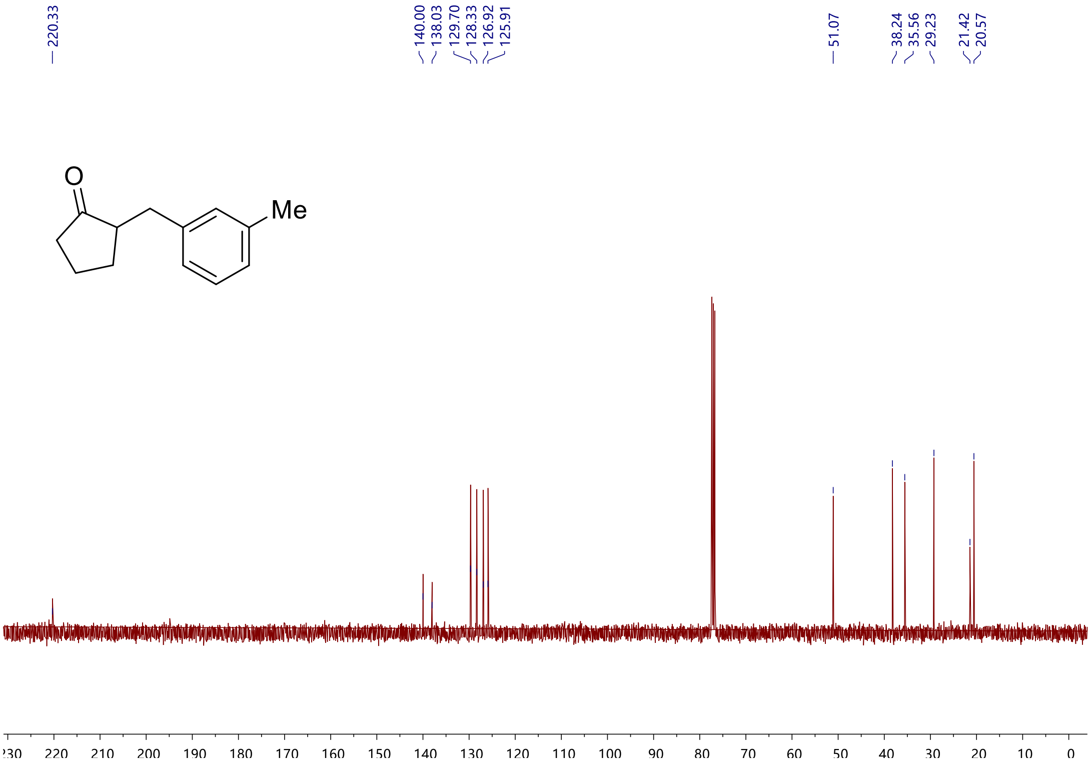

220.33
140.00
138.03
129.70
128.33
126.92
125.91
51.07
38.24
35.56
29.23
21.42
20.57
O
Me

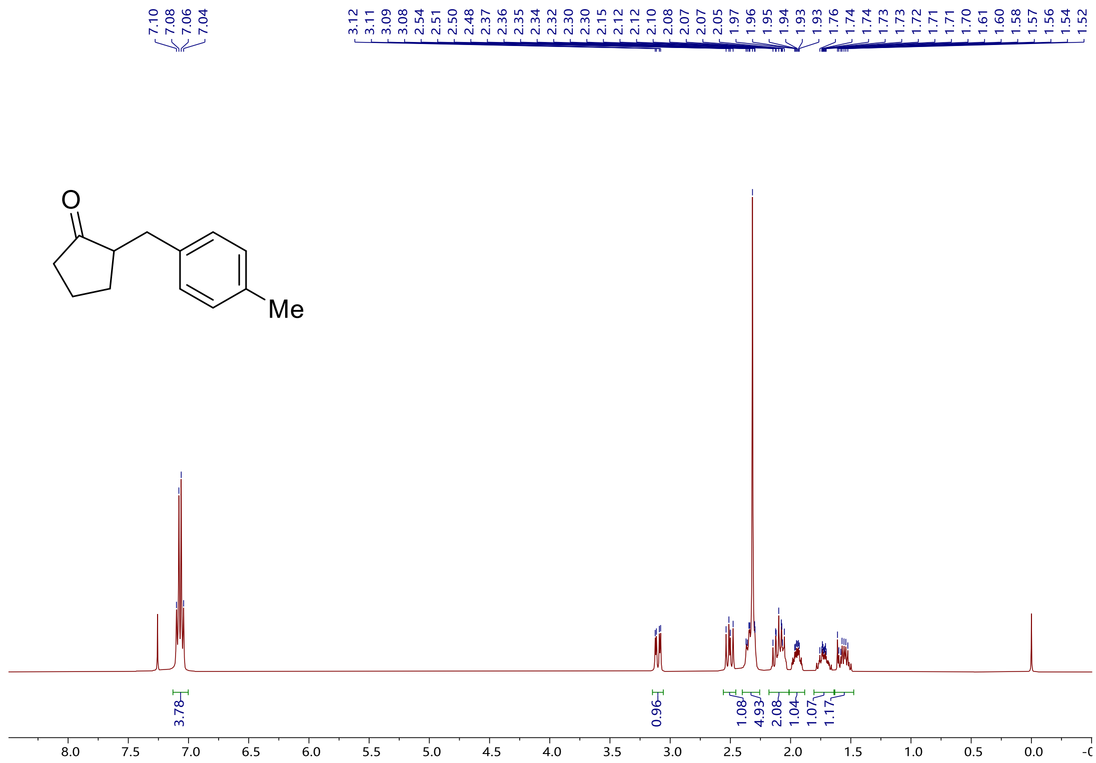

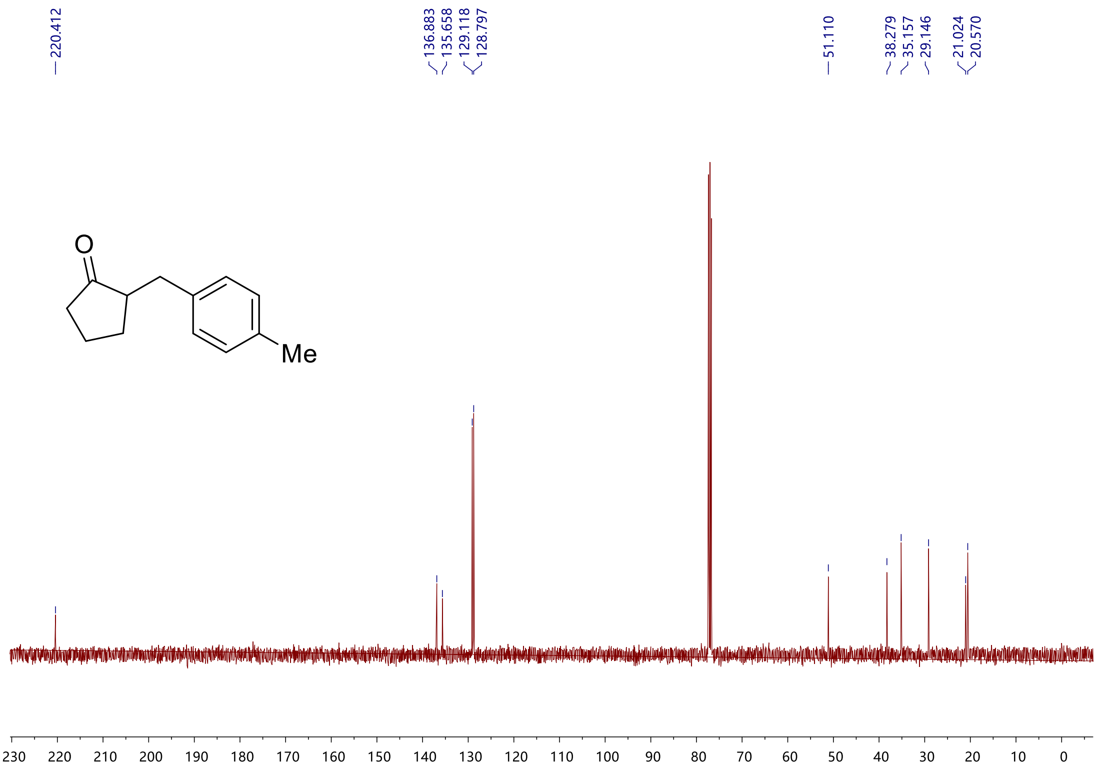

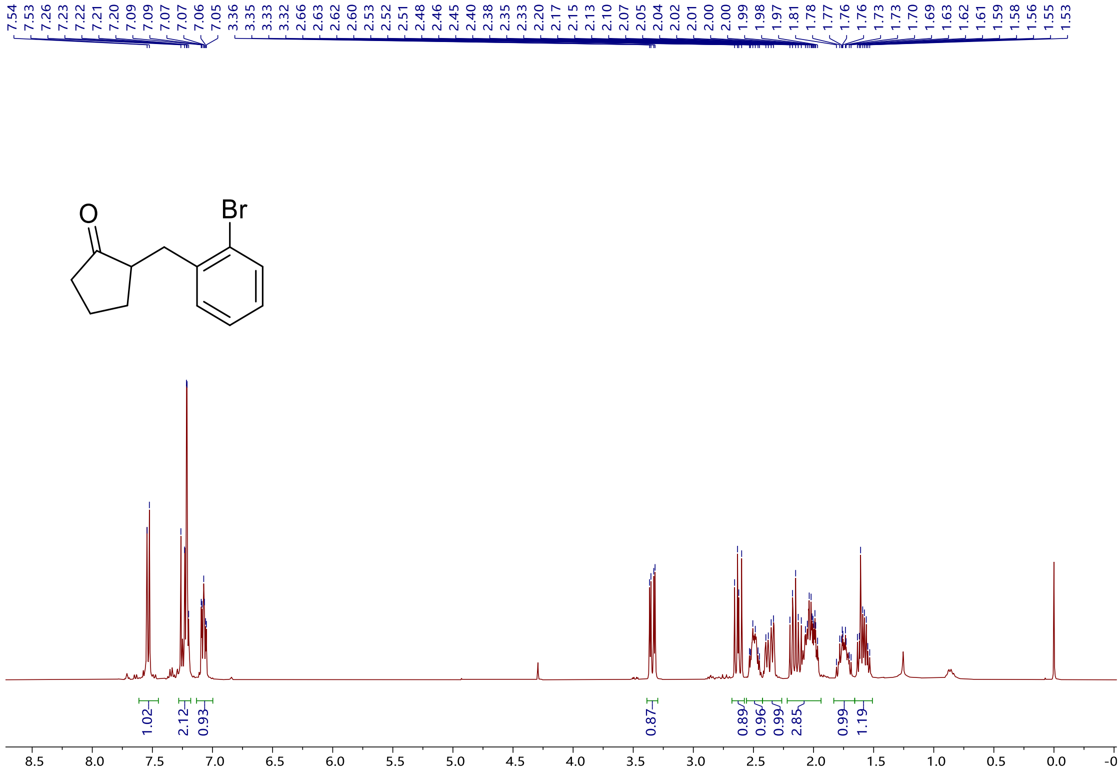
7.54 7.53 7.26 7.23 7.22 7.21 7.20 7.09 7.09 7.07 7.07 7.06 7.05 3.36 3.35 3.33 3.32 2.66 2.63 2.62 2.60 2.53 2.52 2.51 2.48 2.46 2.45 2.40 2.38 2.35 2.33 2.20 2.17 2.15 2.13 2.10 2.07 2.05 2.04 2.02 2.01 2.00 2.00 1.99 1.98 1.97 1.81 1.78 1.77 1.76 1.76 1.73 1.73 1.70 1.69 1.63 1.62 1.61 1.59 1.58 1.56 1.55 1.53
O
Br
1.02 2.12 0.93 0.87 0.89 0.96 0.99 2.85 0.99 1.19
8.5 8.0 7.5 7.0 6.5 6.0 5.5 5.0 4.5 4.0 3.5 3.0 2.5 2.0 1.5 1.0 0.5 0.0 -0

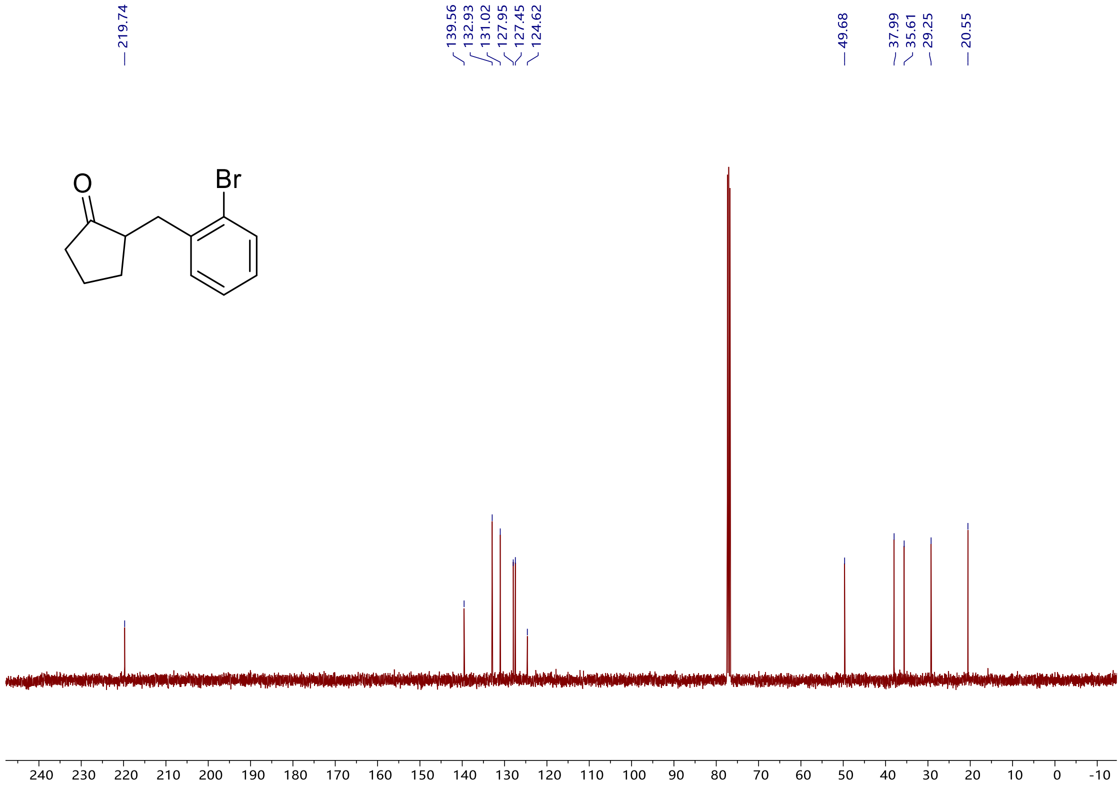
219.74
139.56 132.93 131.02 127.95 127.45 124.62
49.68
37.99 35.61 29.25
20.55
O
Br
240 230 220 210 200 190 180 170 160 150 140 130 120 110 100 90 80 70 60 50 40 30 20 10 0 -10

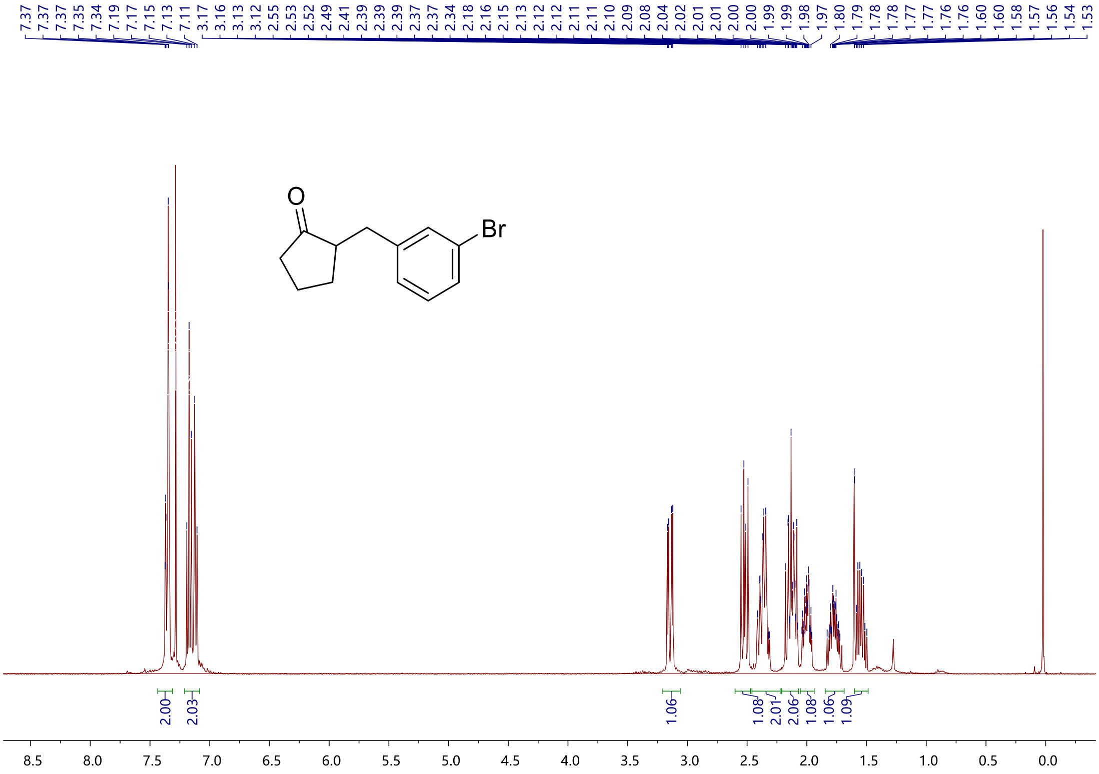
7.37
7.37
7.37
7.35
7.34
7.19
7.17
7.15
7.13
7.11
3.17
3.16
3.13
3.12
2.55
2.53
2.52
2.49
2.41
2.39
2.39
2.39
2.37
2.37
2.34
2.18
2.16
2.15
2.13
2.12
2.12
2.11
2.11
2.10
2.09
2.08
2.04
2.02
2.01
2.01
2.00
1.99
1.99
1.98
1.97
1.80
1.79
1.78
1.77
1.77
1.76
1.76
1.60
1.60
1.58
1.57
1.56
1.54
1.53
O
Br
2.00
2.03
1.06
1.08
2.01
2.06
1.08
1.06
1.09
8.5 8.0 7.5 7.0 6.5 6.0 5.5 5.0 4.5 4.0 3.5 3.0 2.5 2.0 1.5 1.0 0.5 0.0

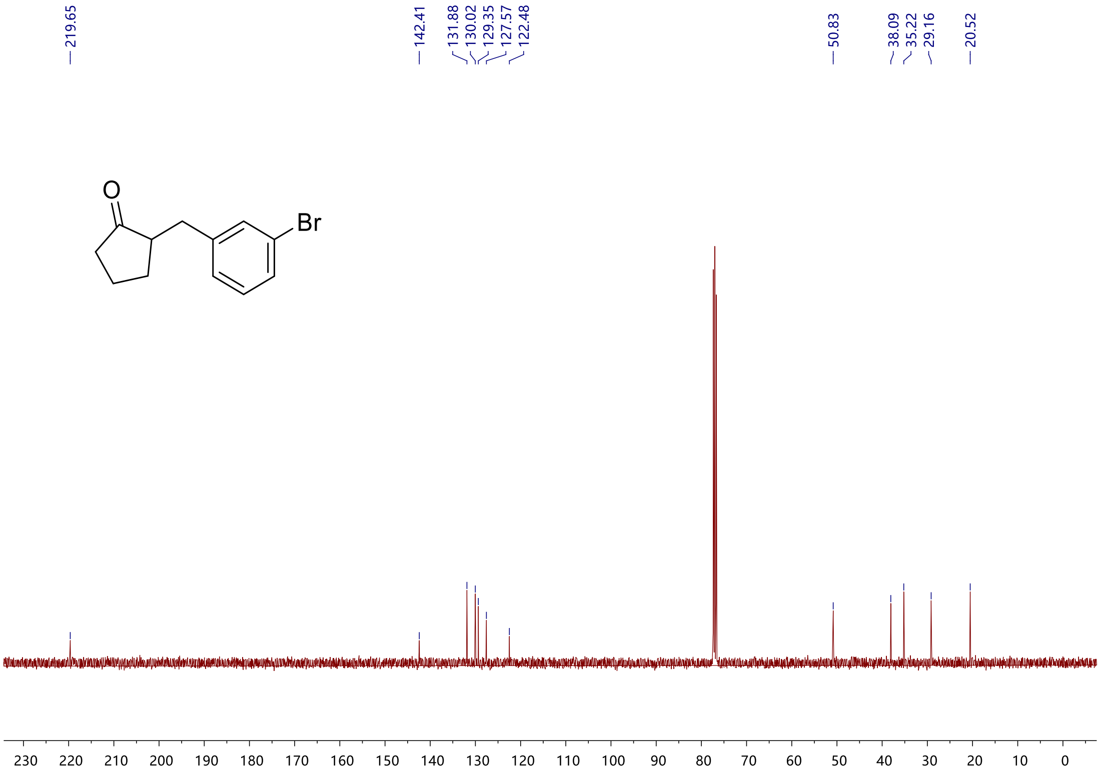
219.65
142.41
131.88
130.02
129.35
127.57
122.48
50.83
38.09
35.22
29.16
20.52
O
Br
230 220 210 200 190 180 170 160 150 140 130 120 110 100 90 80 70 60 50 40 30 20 10 0

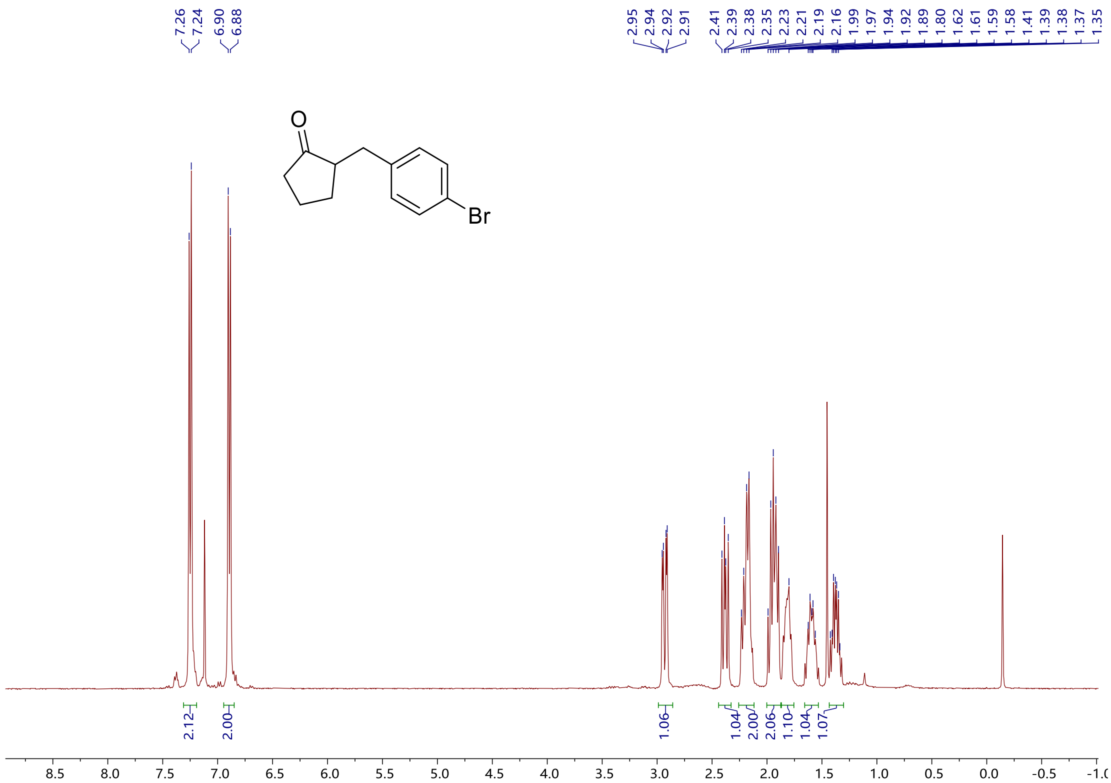
7.26
7.24
6.90
6.88
2.95
2.94
2.92
2.91
2.41
2.39
2.38
2.35
2.23
2.21
2.19
2.16
1.99
1.97
1.94
1.92
1.89
1.80
1.62
1.61
1.59
1.58
1.41
1.39
1.38
1.37
1.35
O
Br
2.12
2.00
1.06
1.04
2.00
2.06
1.10
1.04
1.07

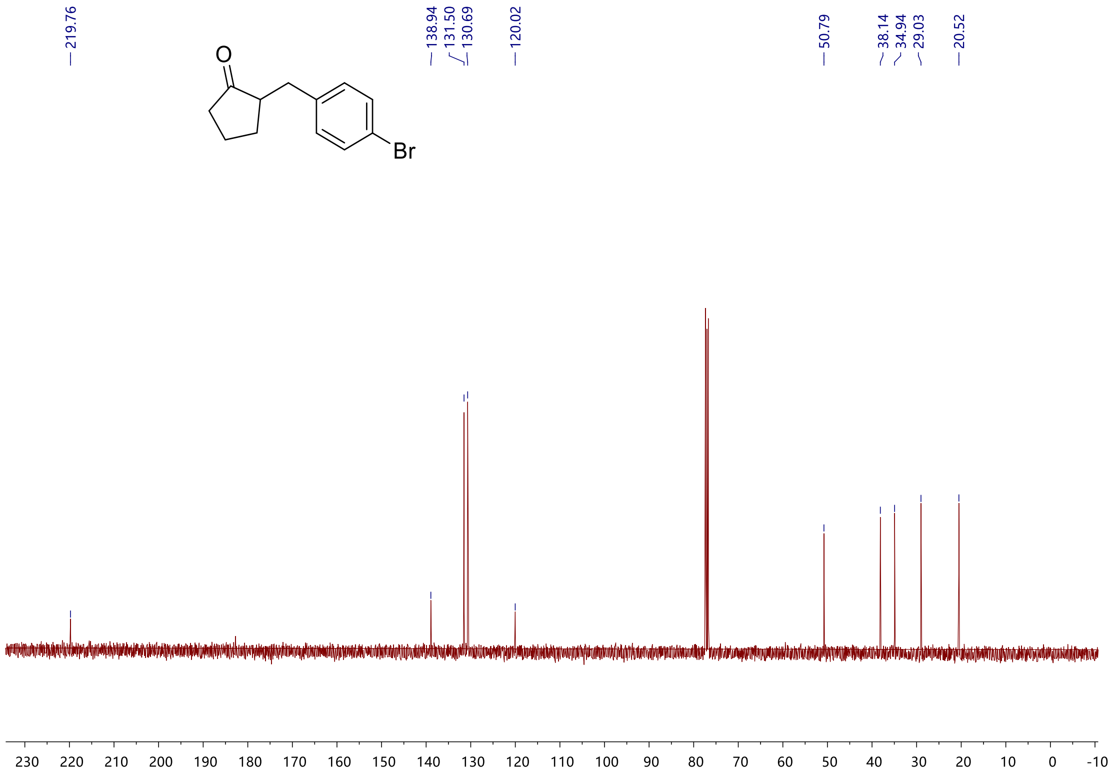
219.76
138.94
131.50
130.69
120.02
50.79
38.14
34.94
29.03
20.52
O
Br

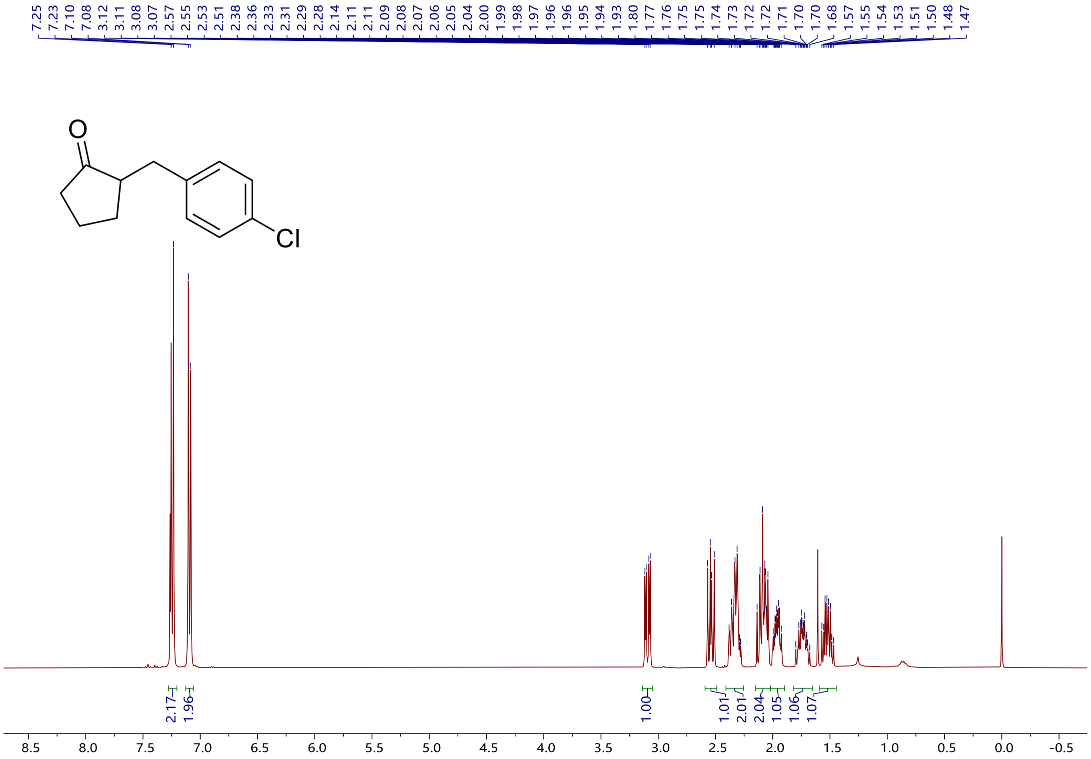
7.25
7.23
7.10
7.08
3.12
3.11
3.08
3.07
2.57
2.55
2.53
2.51
2.38
2.36
2.33
2.31
2.29
2.28
2.14
2.11
2.11
2.09
2.08
2.07
2.06
2.05
2.04
2.00
1.99
1.98
1.97
1.96
1.96
1.95
1.94
1.93
1.80
1.77
1.76
1.75
1.75
1.74
1.73
1.72
1.72
1.71
1.70
1.70
1.68
1.57
1.55
1.54
1.53
1.51
1.50
1.48
1.47
O
Cl
2.17
1.96
1.00
1.01
2.01
2.04
1.05
1.06
1.07
8.5 8.0 7.5 7.0 6.5 6.0 5.5 5.0 4.5 4.0 3.5 3.0 2.5 2.0 1.5 1.0 0.5 0.0 -0.5

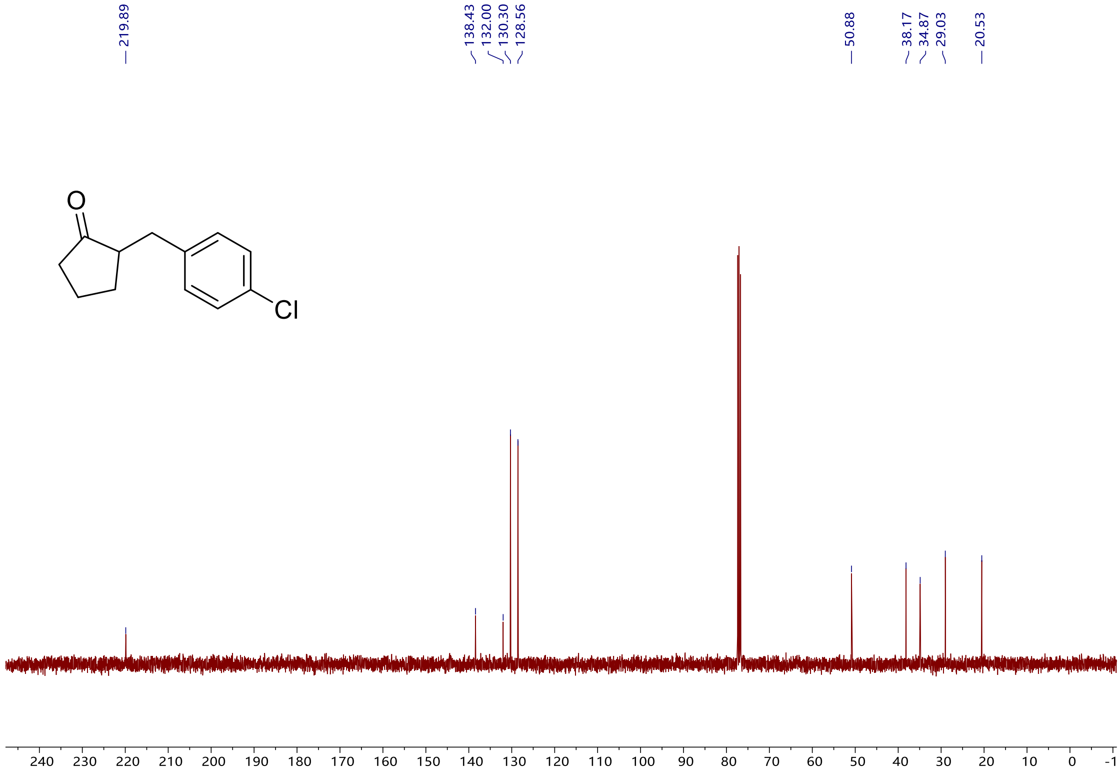
219.89
138.43
132.00
130.30
128.56
50.88
38.17
34.87
29.03
20.53
O
Cl
240 230 220 210 200 190 180 170 160 150 140 130 120 110 100 90 80 70 60 50 40 30 20 10 0 -1

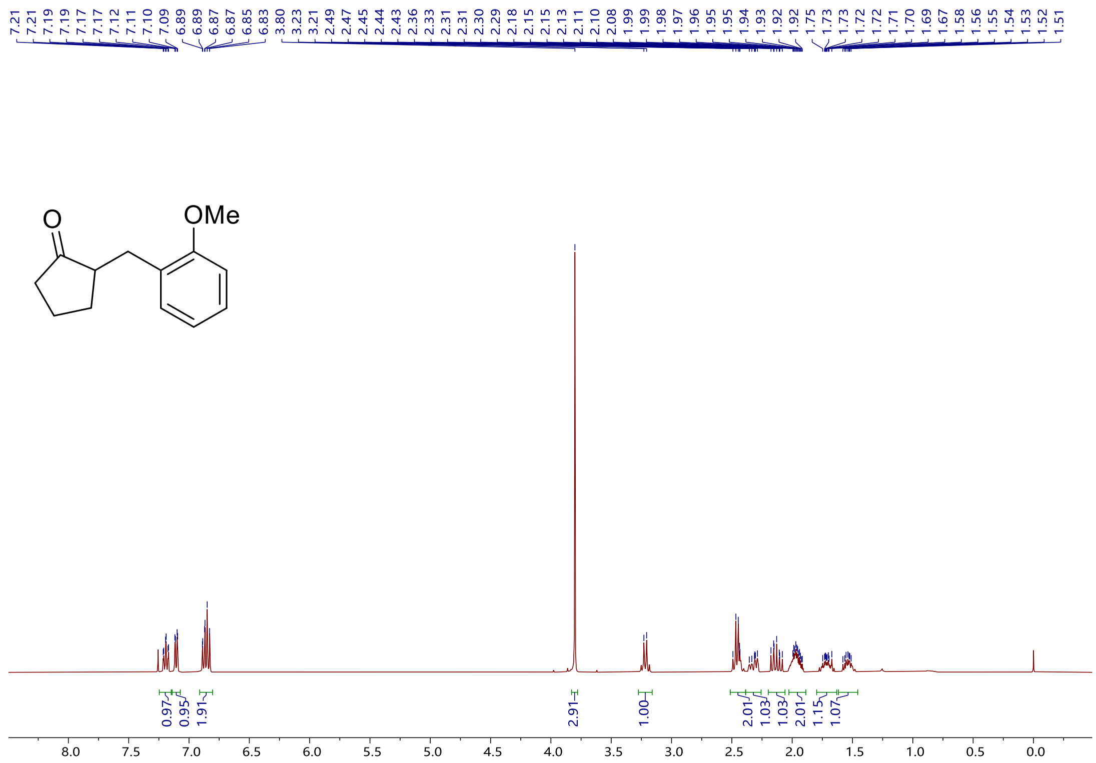

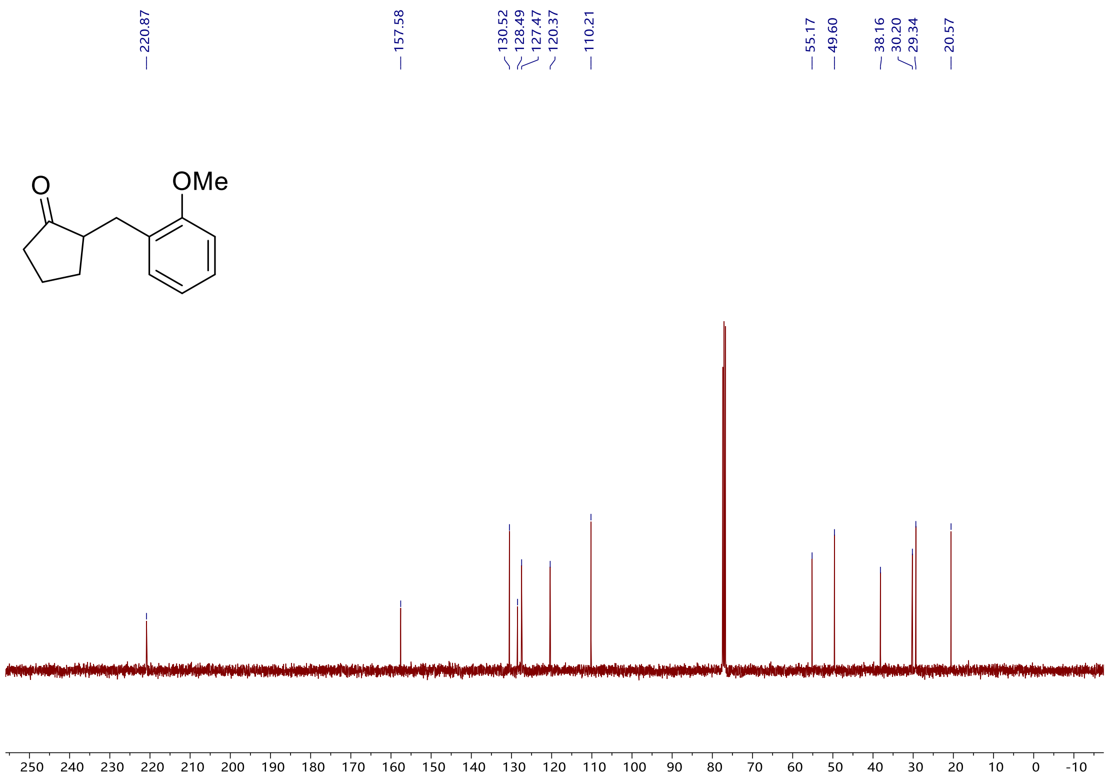

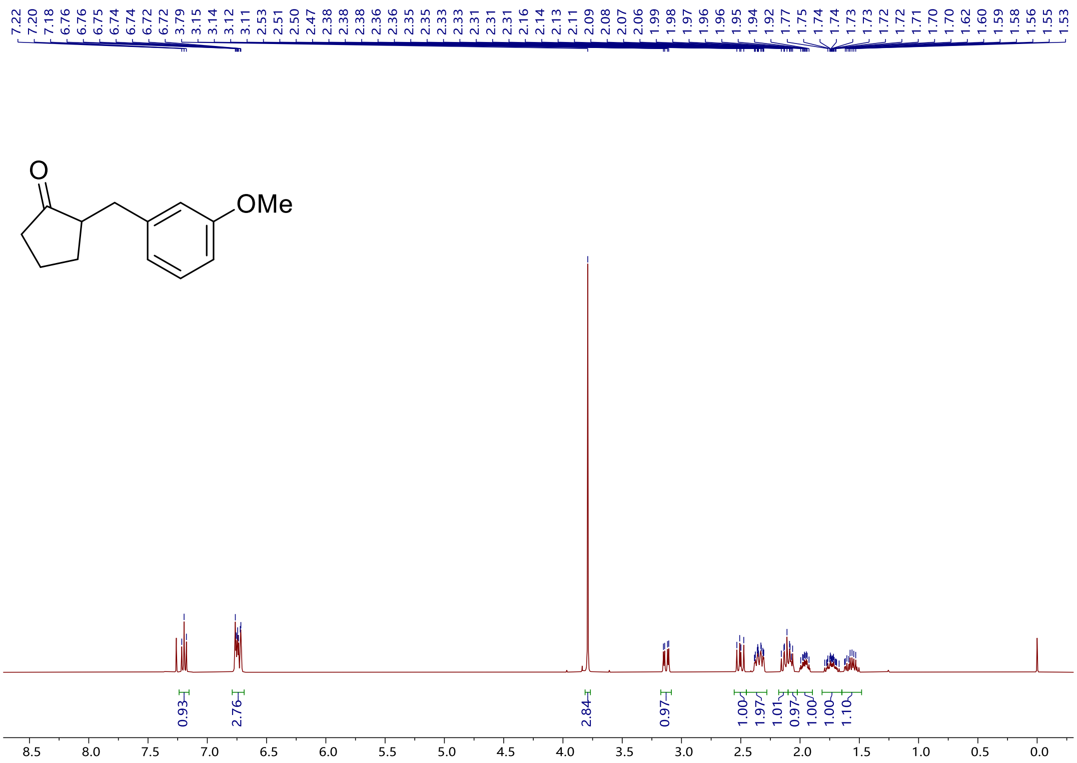

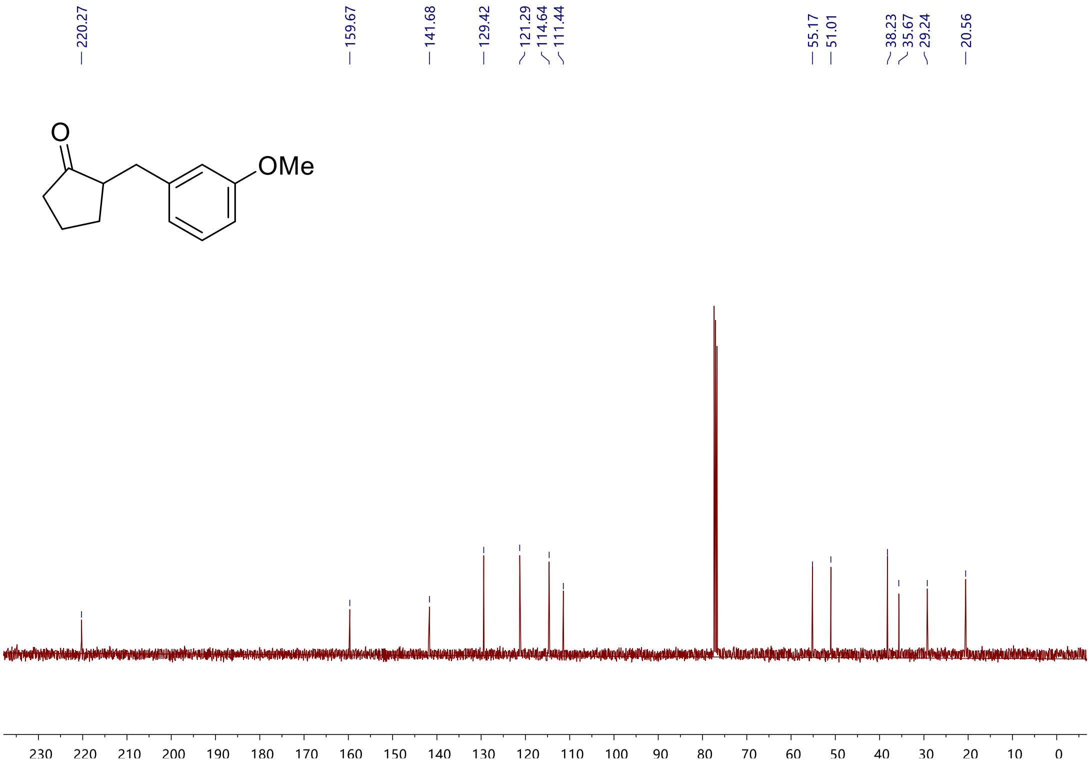

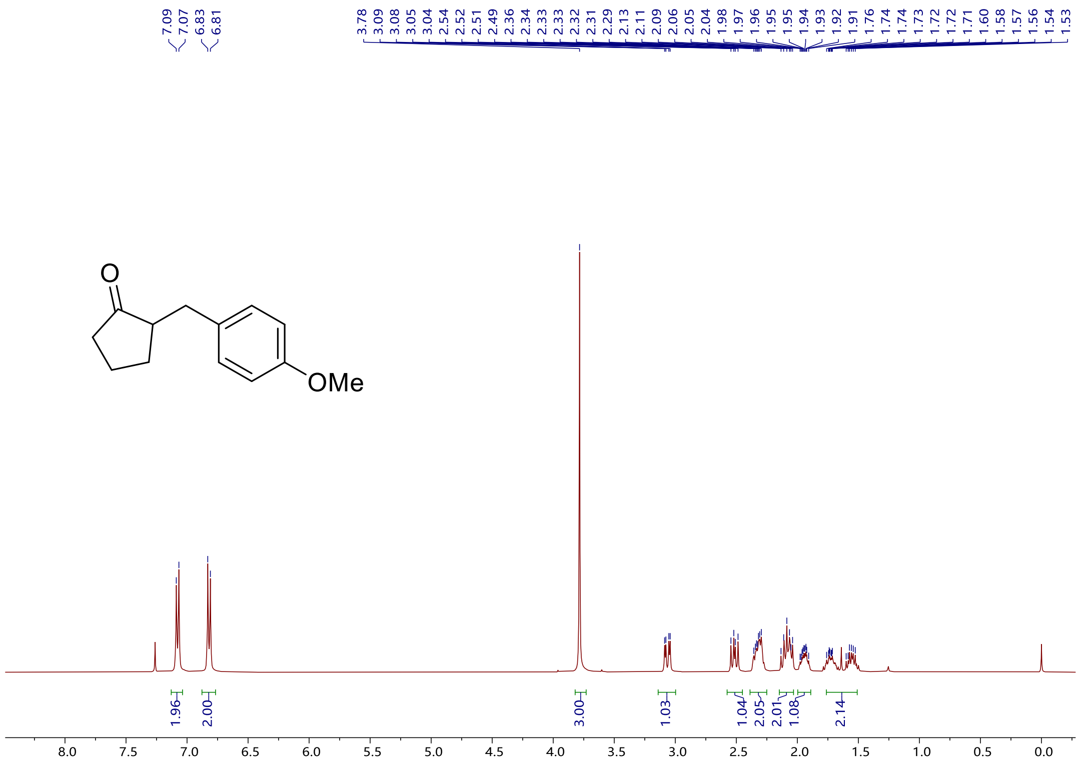
7.09
7.07
6.83
6.81
3.78
3.09
3.08
3.05
3.04
2.54
2.52
2.51
2.49
2.36
2.34
2.33
2.33
2.32
2.31
2.29
2.13
2.11
2.09
2.06
2.05
2.04
1.98
1.97
1.96
1.95
1.95
1.94
1.93
1.92
1.91
1.76
1.74
1.74
1.73
1.72
1.72
1.71
1.60
1.58
1.57
1.56
1.54
1.53
OMe
O
1.96
2.00
3.00
1.03
1.04
2.05
2.01
1.08
2.14
8.0 7.5 7.0 6.5 6.0 5.5 5.0 4.5 4.0 3.5 3.0 2.5 2.0 1.5 1.0 0.5 0.0

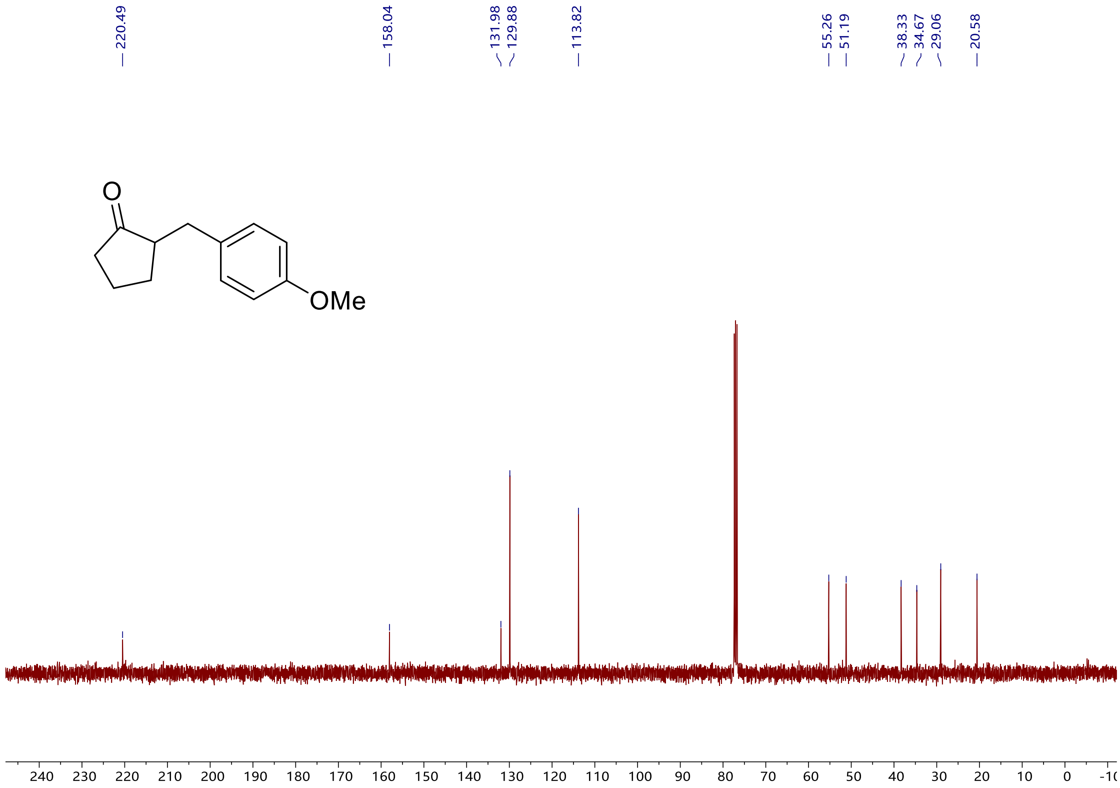
220.49
158.04
131.98
129.88
113.82
55.26
51.19
38.33
34.67
29.06
20.58
O
OMe
240 230 220 210 200 190 180 170 160 150 140 130 120 110 100 90 80 70 60 50 40 30 20 10 0

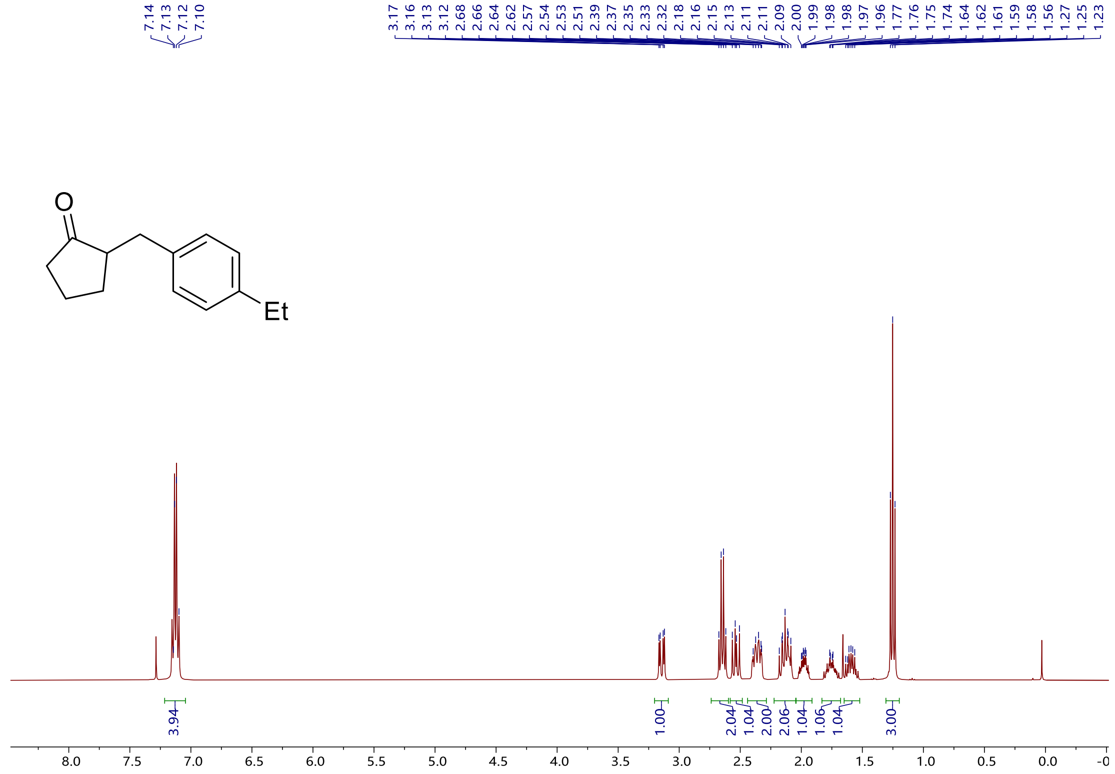
7.14
7.13
7.12
7.10
3.17
3.16
3.13
3.12
2.68
2.66
2.64
2.62
2.57
2.54
2.53
2.51
2.39
2.37
2.35
2.33
2.32
2.18
2.16
2.15
2.13
2.11
2.11
2.09
2.00
1.99
1.98
1.98
1.97
1.96
1.77
1.76
1.75
1.74
1.64
1.62
1.61
1.59
1.58
1.56
1.27
1.25
1.23
O
Et
3.94
1.00
2.04
1.04
2.00
2.06
1.04
1.06
1.04
3.00
8.0 7.5 7.0 6.5 6.0 5.5 5.0 4.5 4.0 3.5 3.0 2.5 2.0 1.5 1.0 0.5 0.0

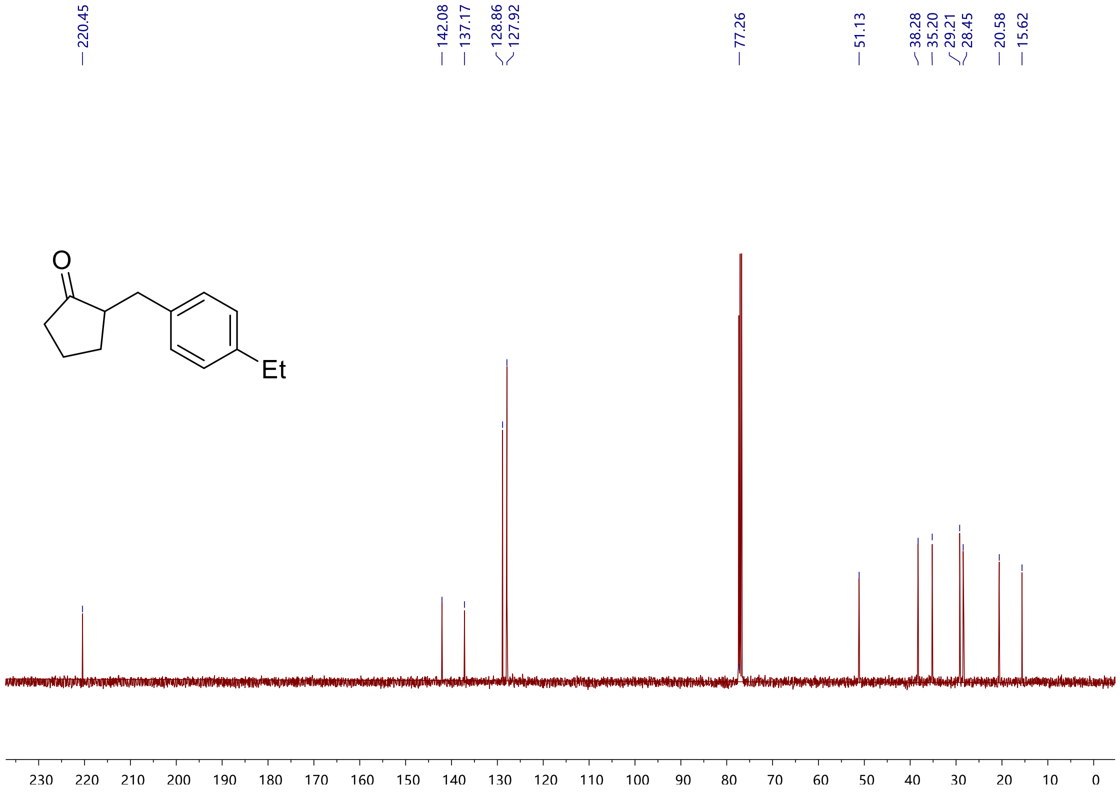
220.45
142.08
137.17
128.86
127.92
77.26
51.13
38.28
35.20
29.21
28.45
20.58
15.62
O
Et
230 220 210 200 190 180 170 160 150 140 130 120 110 100 90 80 70 60 50 40 30 20 10 0

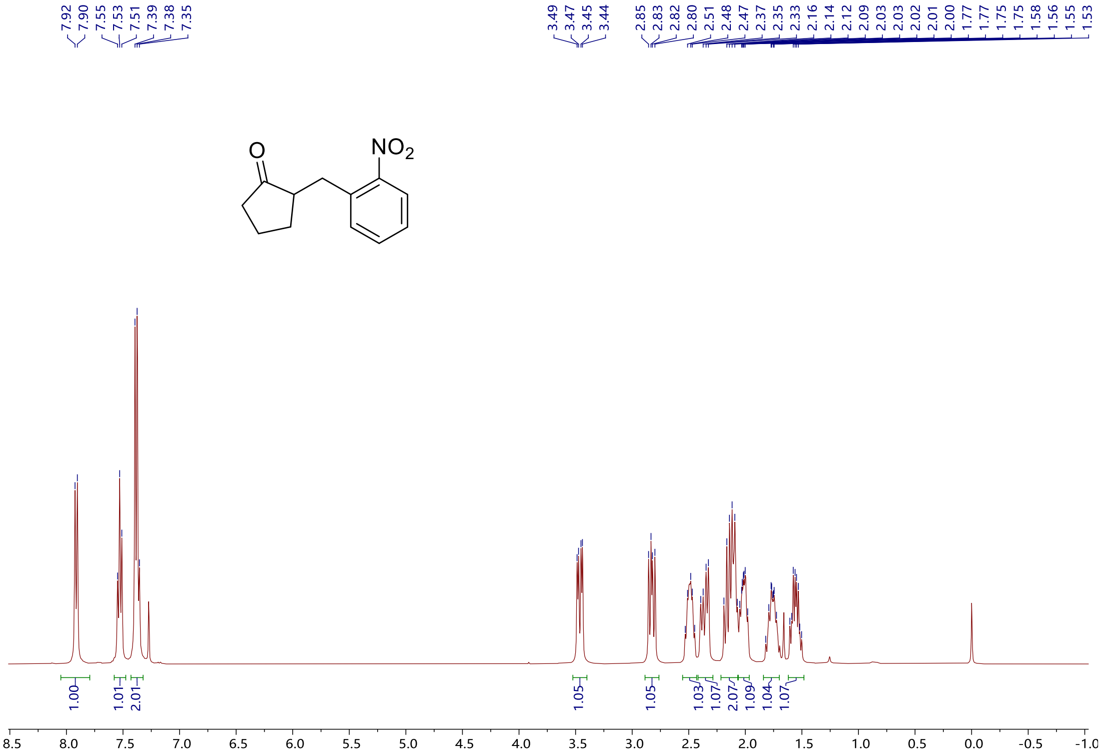
7.92
7.90
7.55
7.53
7.51
7.39
7.38
7.35
3.49
3.47
3.45
3.44
2.85
2.83
2.82
2.80
2.51
2.48
2.47
2.37
2.35
2.33
2.16
2.14
2.12
2.09
2.03
2.03
2.02
2.01
2.00
1.77
1.77
1.75
1.75
1.58
1.56
1.55
1.53
O
NO2
1.00
1.01
2.01
1.05
1.05
1.03
1.07
2.07
1.09
1.04
1.07
8.5 8.0 7.5 7.0 6.5 6.0 5.5 5.0 4.5 4.0 3.5 3.0 2.5 2.0 1.5 1.0 0.5 0.0 -0.5 -1.0

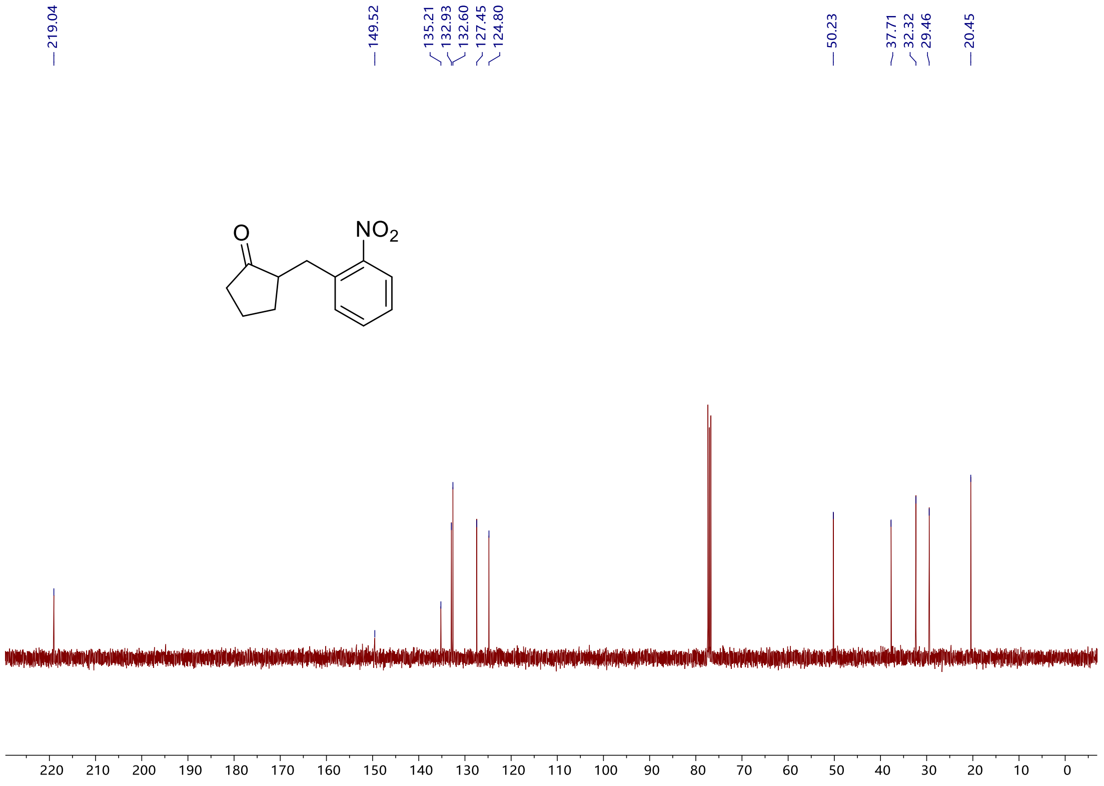
219.04
149.52
135.21
132.93
132.60
127.45
124.80
50.23
37.71
32.32
29.46
20.45
O
NO2
220 210 200 190 180 170 160 150 140 130 120 110 100 90 80 70 60 50 40 30 20 10 0

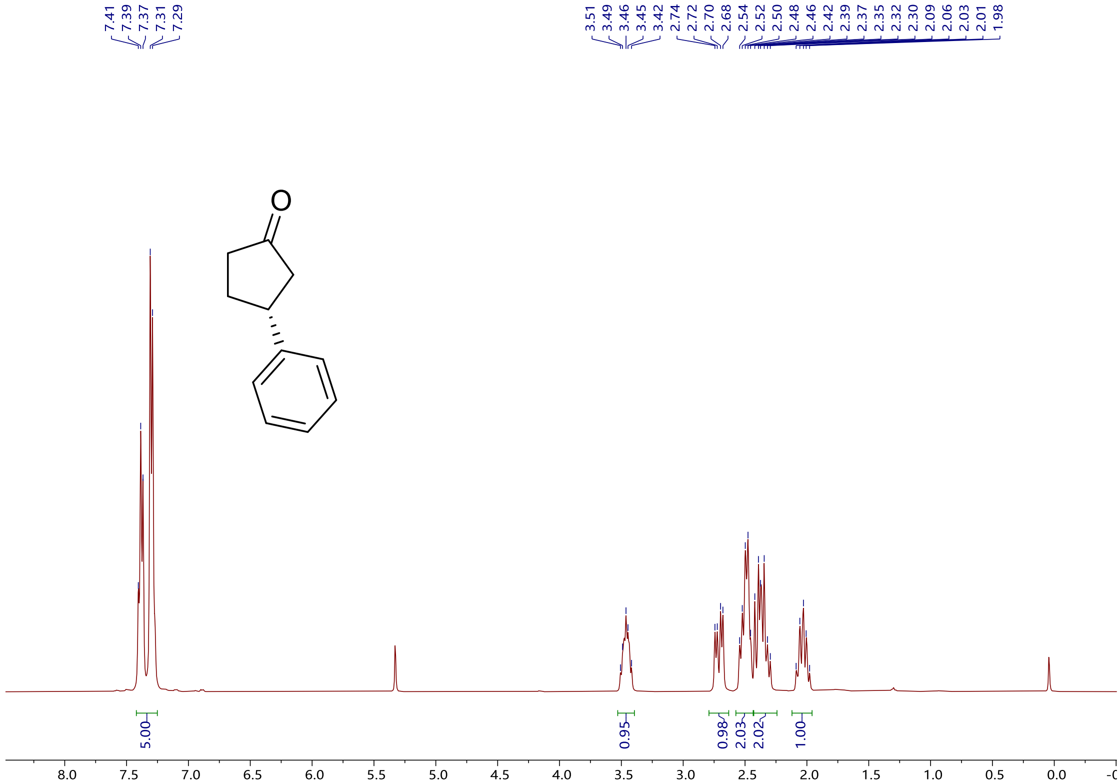
7.41
7.39
7.37
7.31
7.29
3.51
3.49
3.46
3.45
3.42
2.74
2.72
2.70
2.68
2.54
2.52
2.50
2.48
2.46
2.42
2.39
2.37
2.35
2.32
2.30
2.09
2.06
2.03
2.01
1.98
O
5.00
0.95
0.98
2.03
2.02
1.00
8.0
7.5
7.0
6.5
6.0
5.5
5.0
4.5
4.0
3.5
3.0
2.5
2.0
1.5
1.0
0.5
0.0

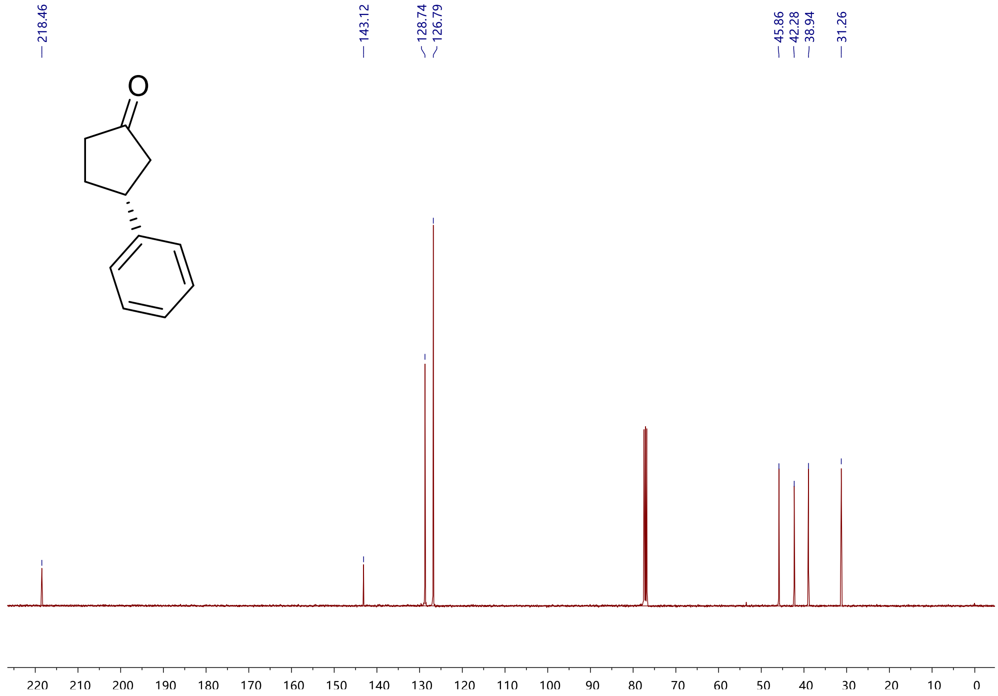
218.46
143.12
128.74
126.79
45.86
42.28
38.94
31.26
O
220
210
200
190
180
170
160
150
140
130
120
110
100
90
80
70
60
50
40
30
20
10
0

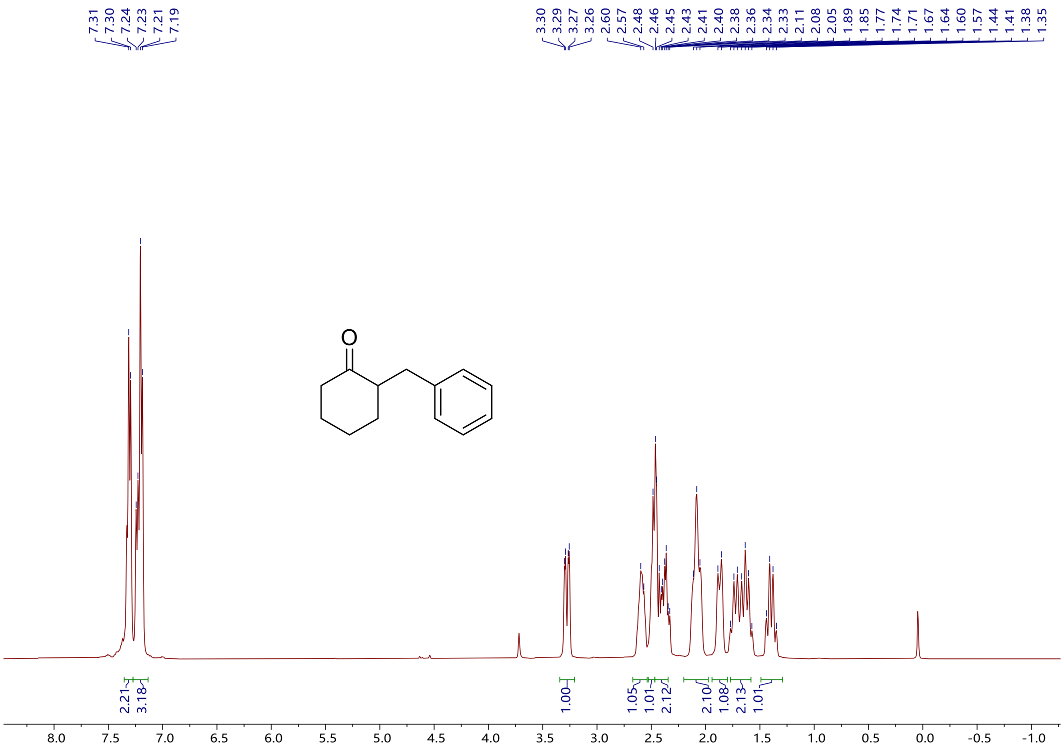
7.31
7.30
7.24
7.23
7.21
7.19
3.30
3.29
3.27
3.26
2.60
2.57
2.48
2.46
2.45
2.43
2.41
2.40
2.38
2.36
2.34
2.33
2.11
2.08
2.05
1.89
1.85
1.77
1.74
1.71
1.67
1.64
1.60
1.57
1.44
1.41
1.38
1.35
O
2.21
3.18
1.00
1.05
1.01
2.12
2.10
1.08
2.13
1.01
8.0
7.5
7.0
6.5
6.0
5.5
5.0
4.5
4.0
3.5
3.0
2.5
2.0
1.5
1.0
0.5
0.0
-0.5
-1.0

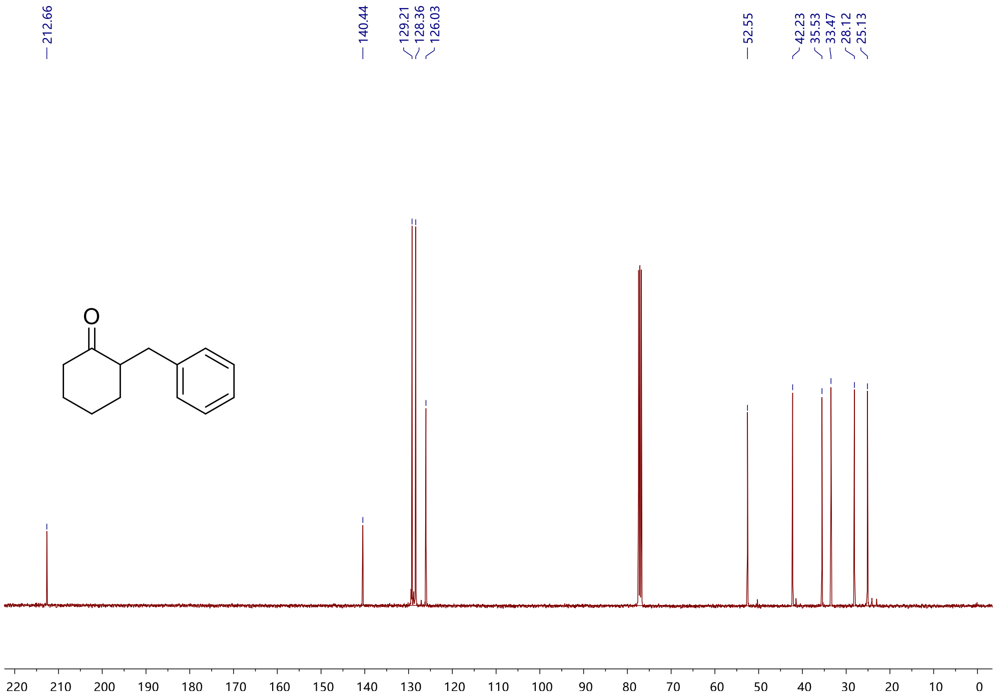
212.66
140.44
129.21
128.36
126.03
52.55
42.23
35.53
33.47
28.12
25.13
O
220
210
200
190
180
170
160
150
140
130
120
110
100
90
80
70
60
50
40
30
20
10
0

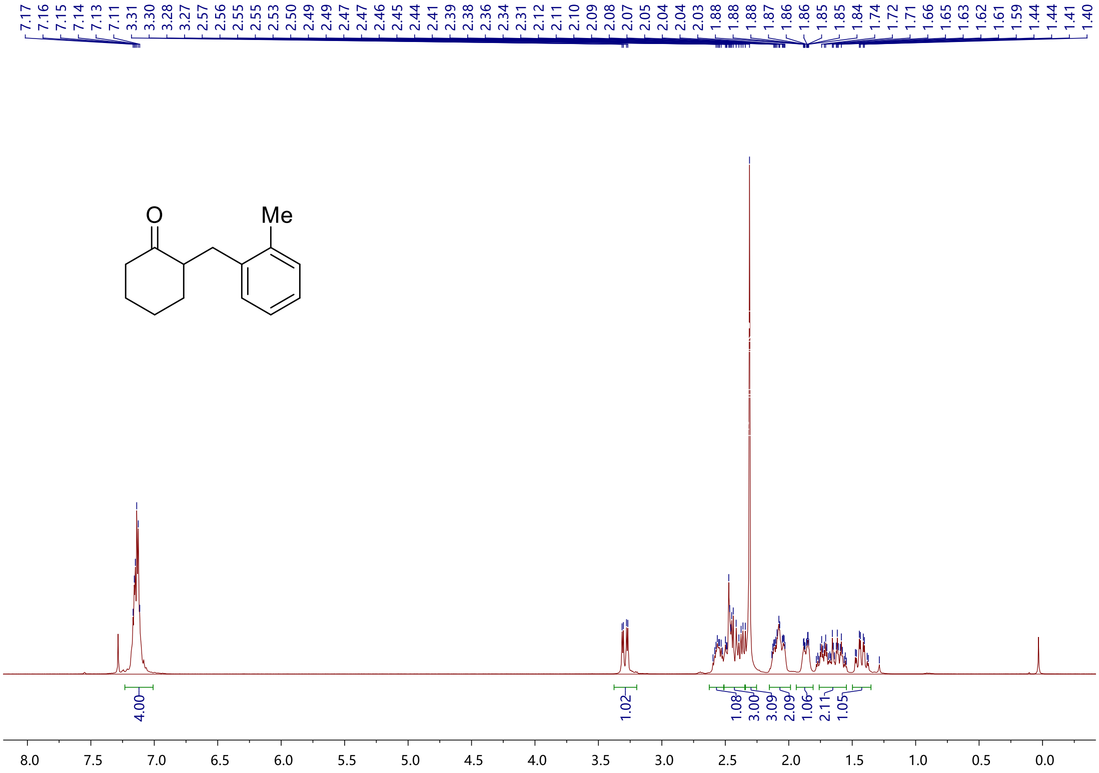

7.17
7.16
7.15
7.14
7.13
7.11
3.31
3.30
3.28
3.27
2.57
2.56
2.55
2.55
2.53
2.50
2.49
2.49
2.47
2.46
2.45
2.44
2.41
2.39
2.38
2.36
2.34
2.31
2.12
2.11
2.10
2.09
2.08
2.07
2.05
2.04
2.04
2.03
1.88
1.88
1.87
1.86
1.86
1.85
1.85
1.84
1.74
1.72
1.71
1.66
1.65
1.63
1.62
1.61
1.59
1.44
1.41
1.40
O
Me
4.00
1.02
1.08
3.00
3.09
2.09
1.06
2.11
1.05
8.0
7.5
7.0
6.5
6.0
5.5
5.0
4.5
4.0
3.5
3.0
2.5
2.0
1.5
1.0
0.5
0.0

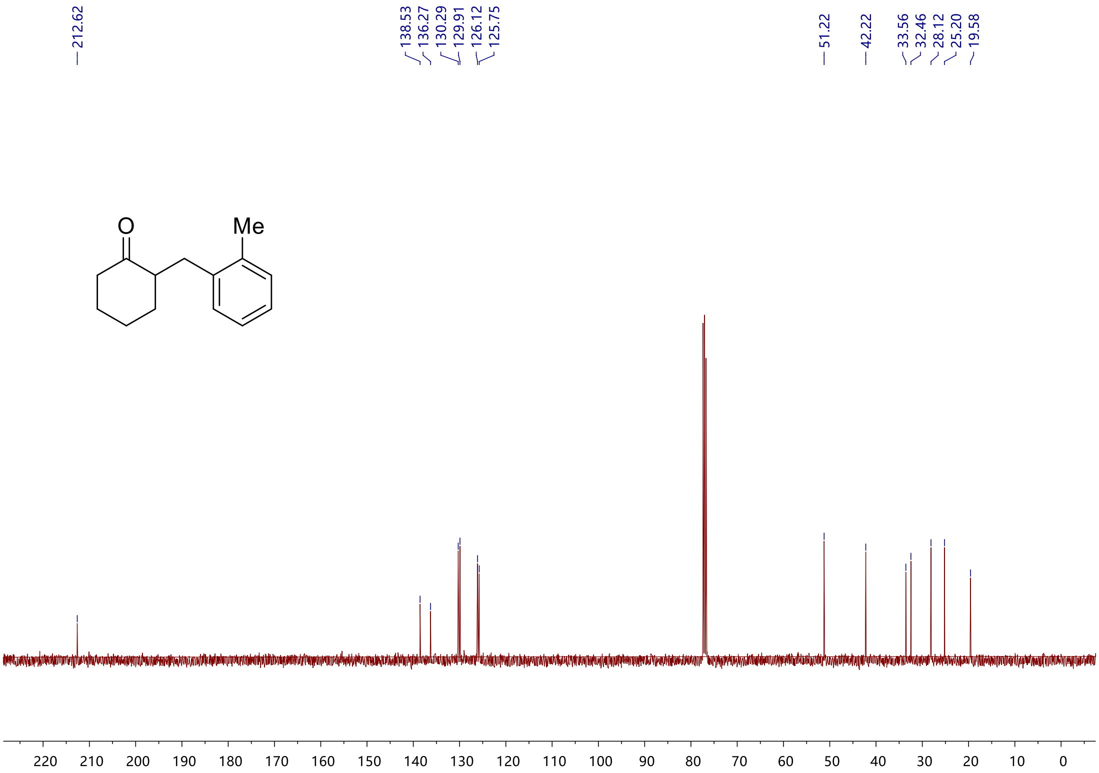

212.62
138.53
136.27
130.29
129.91
126.12
125.75
51.22
42.22
33.56
32.46
28.12
25.20
19.58
O
Me
220
210
200
190
180
170
160
150
140
130
120
110
100
90
80
70
60
50
40
30
20
10
0

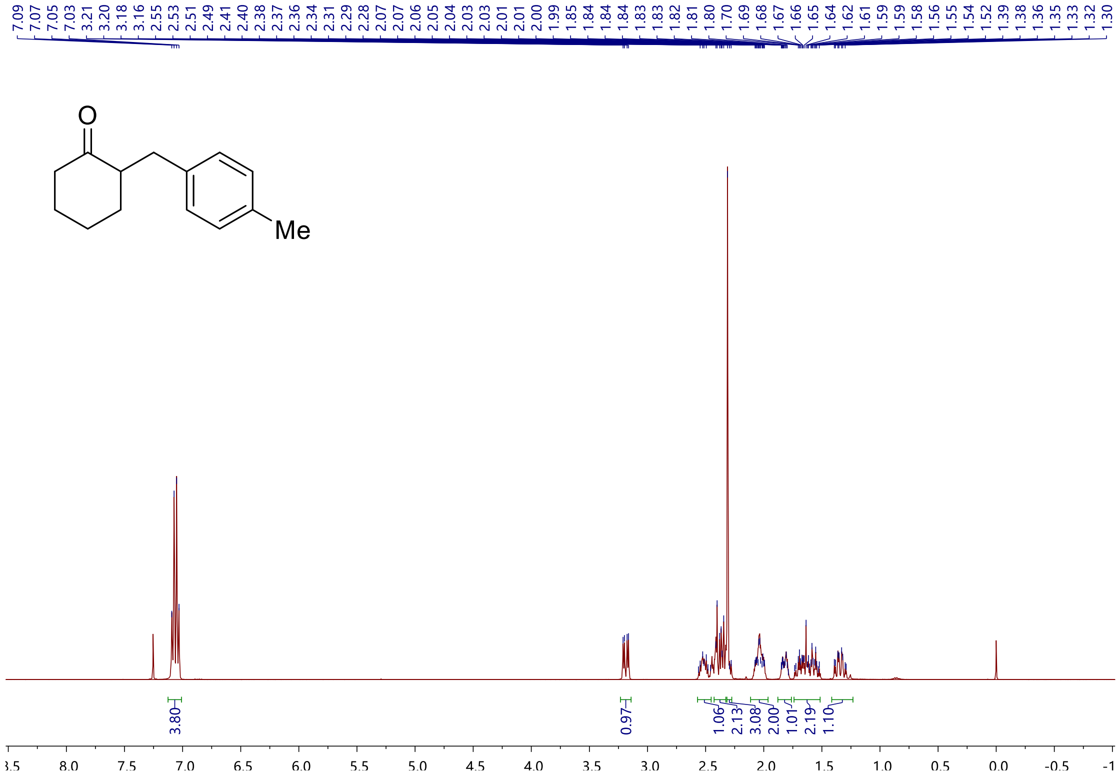

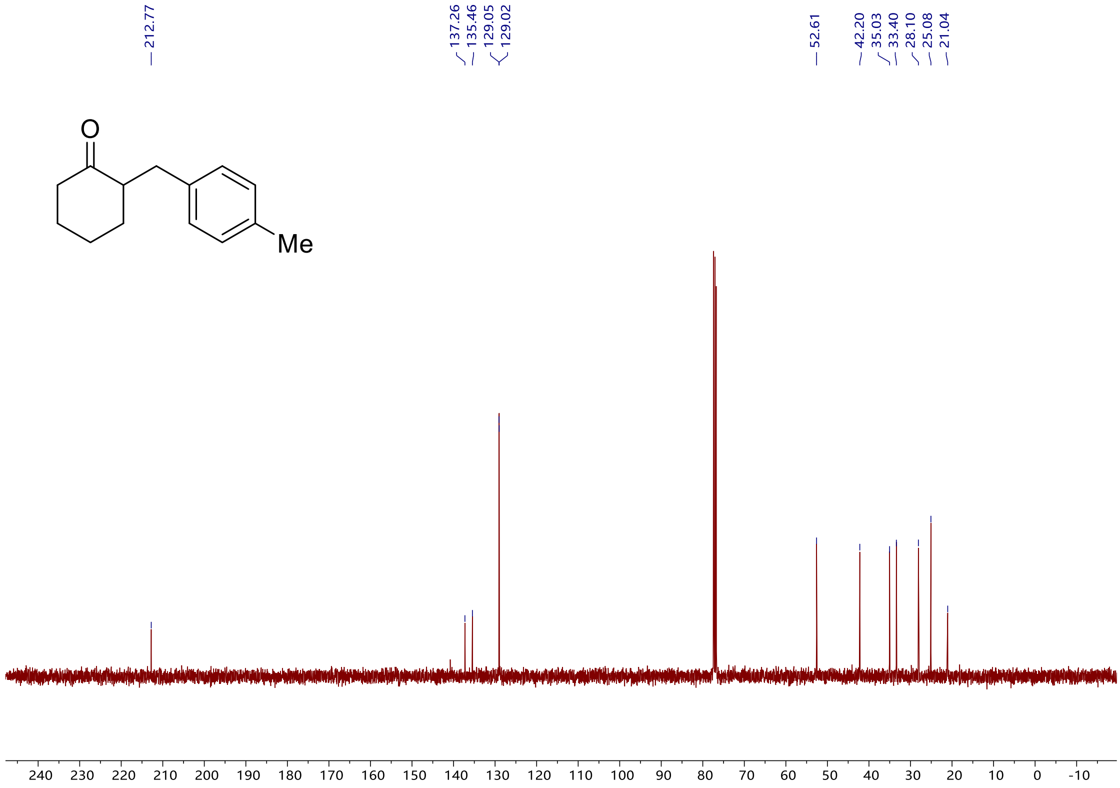

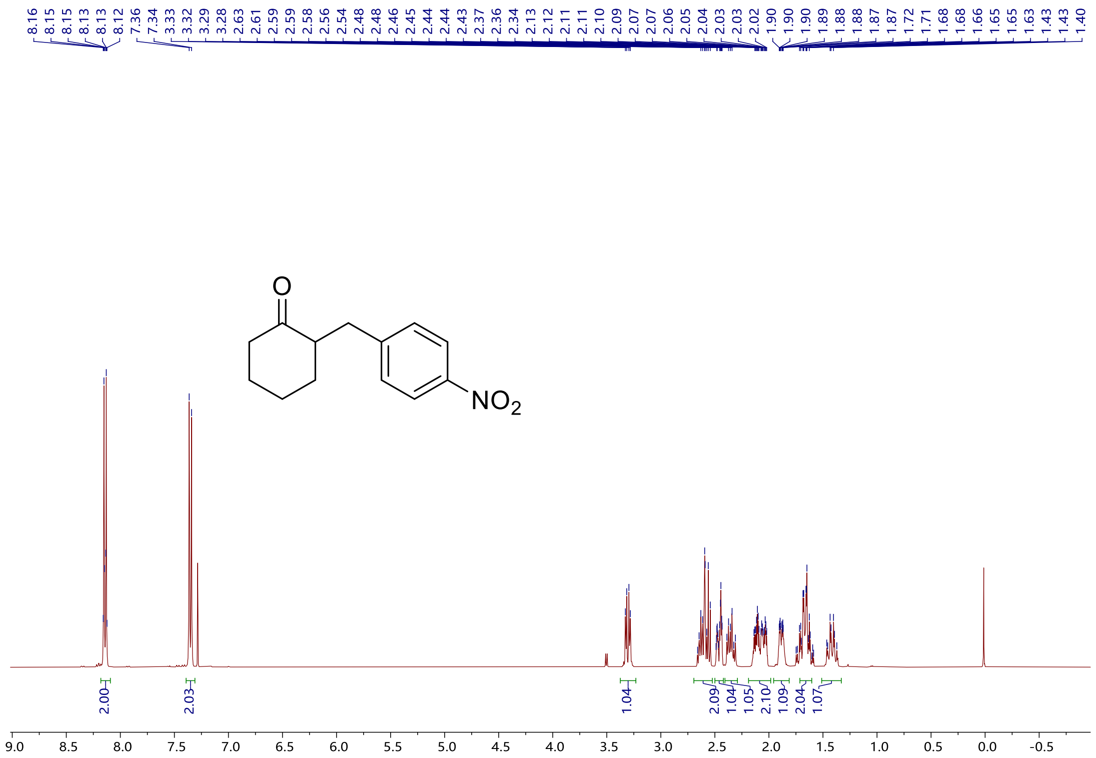

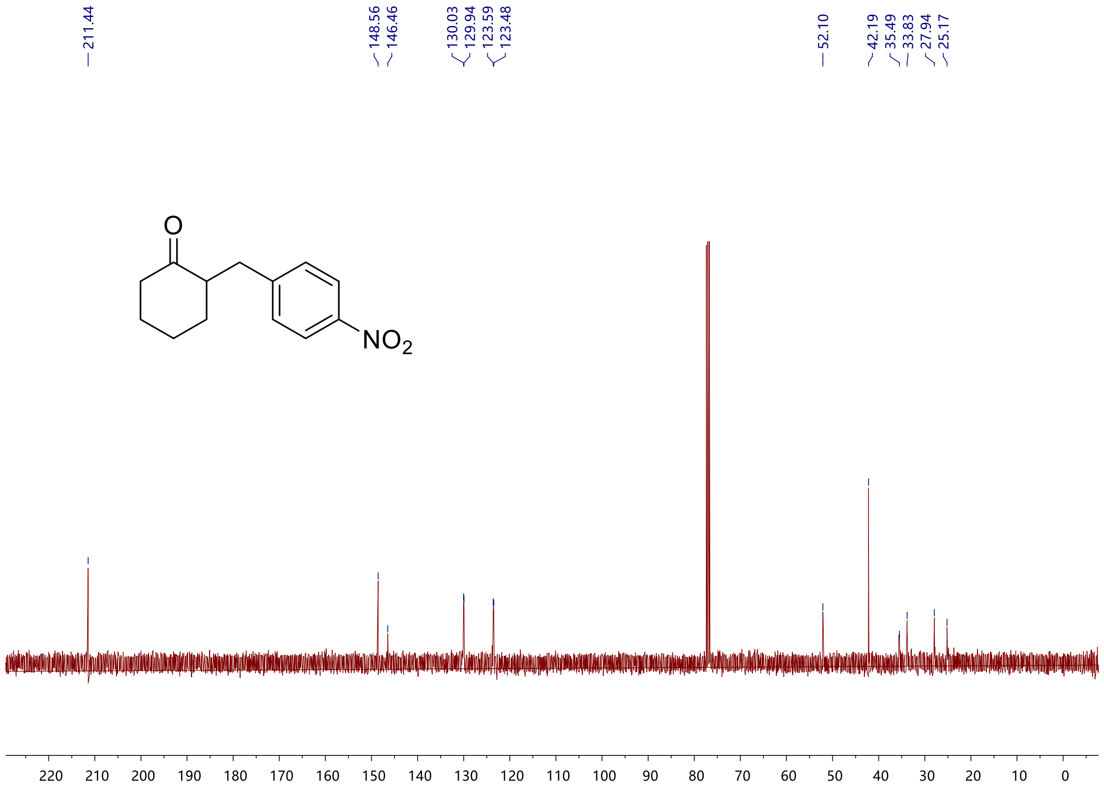

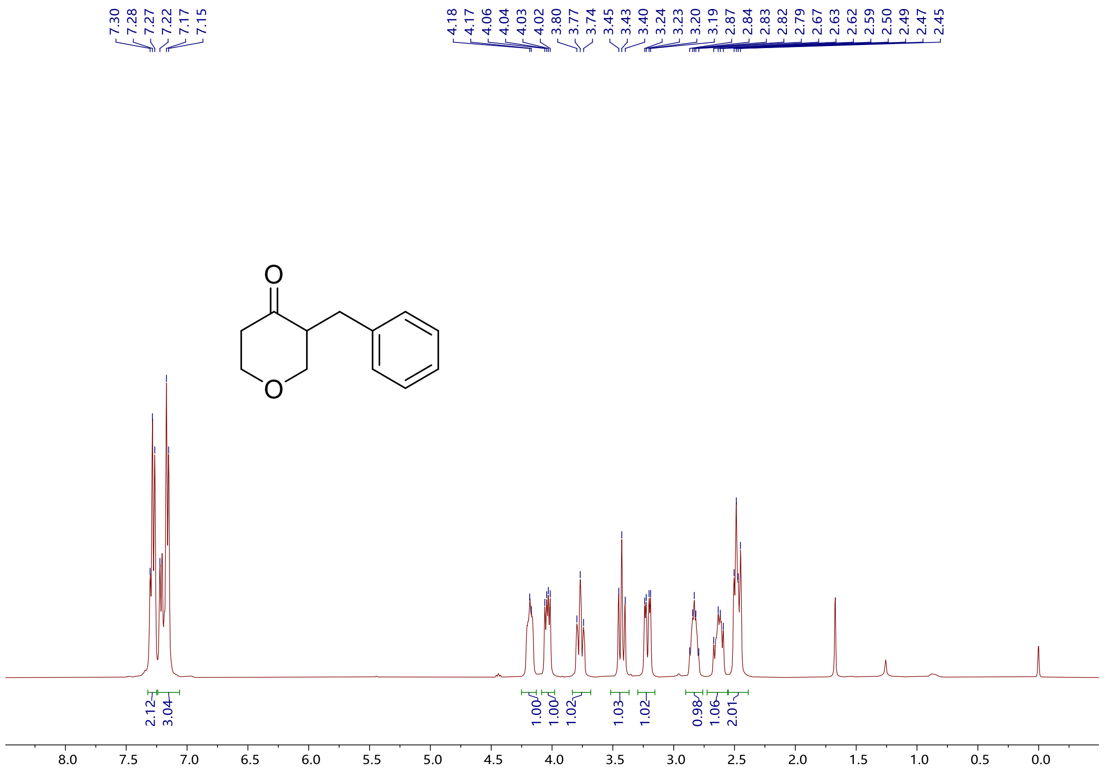

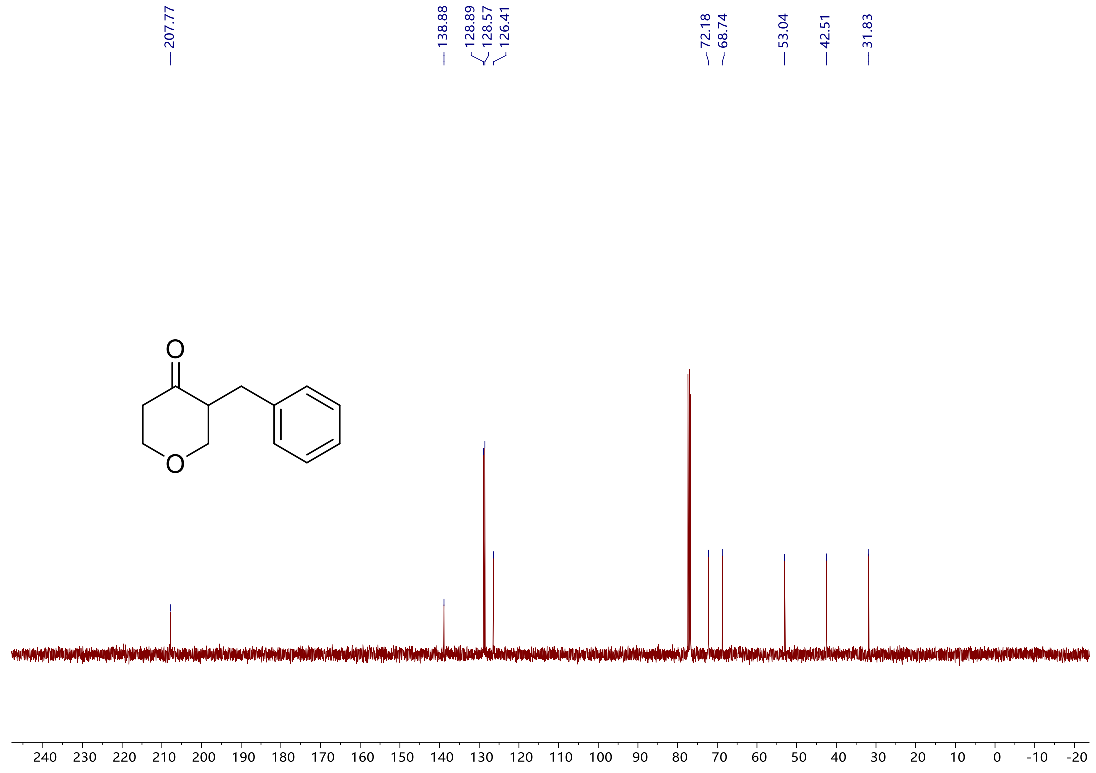

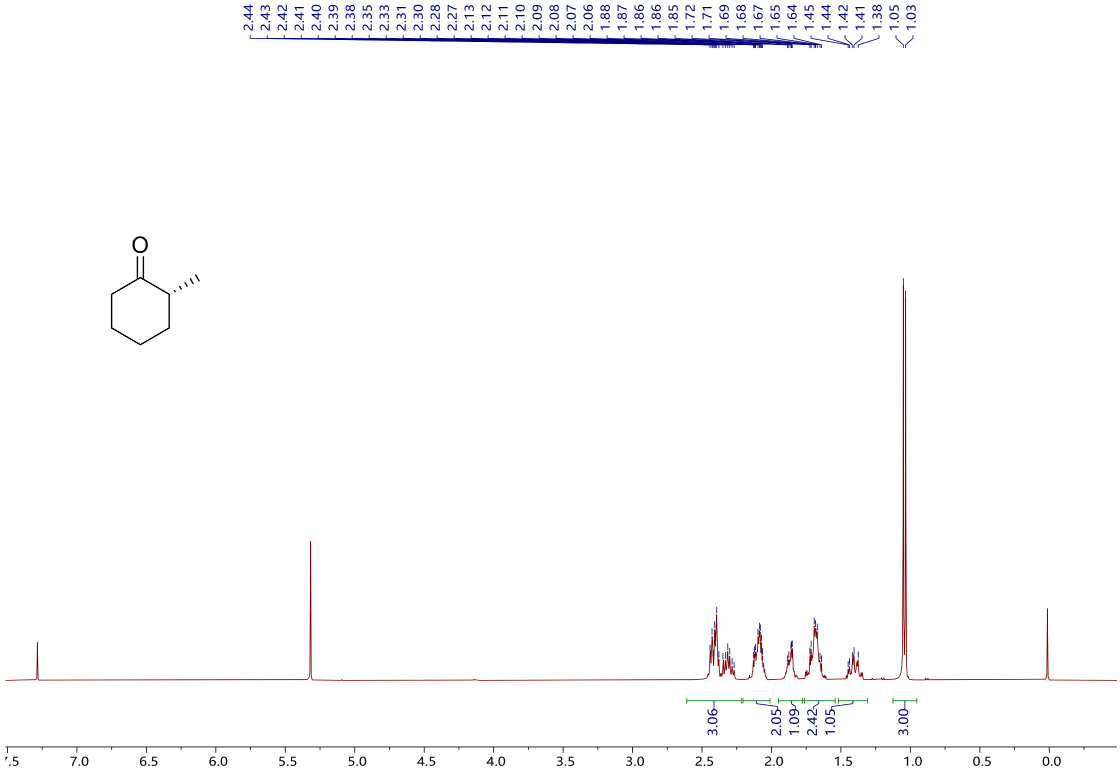
2.44
2.43
2.42
2.41
2.40
2.39
2.38
2.35
2.33
2.31
2.30
2.28
2.27
2.13
2.12
2.11
2.10
2.09
2.08
2.07
2.06
1.88
1.87
1.86
1.86
1.85
1.72
1.71
1.69
1.68
1.67
1.65
1.64
1.45
1.44
1.42
1.41
1.38
1.05
1.03
O
3.06
2.05
1.09
2.42
1.05
3.00
7.5
7.0
6.5
6.0
5.5
5.0
4.5
4.0
3.5
3.0
2.5
2.0
1.5
1.0
0.5
0.0

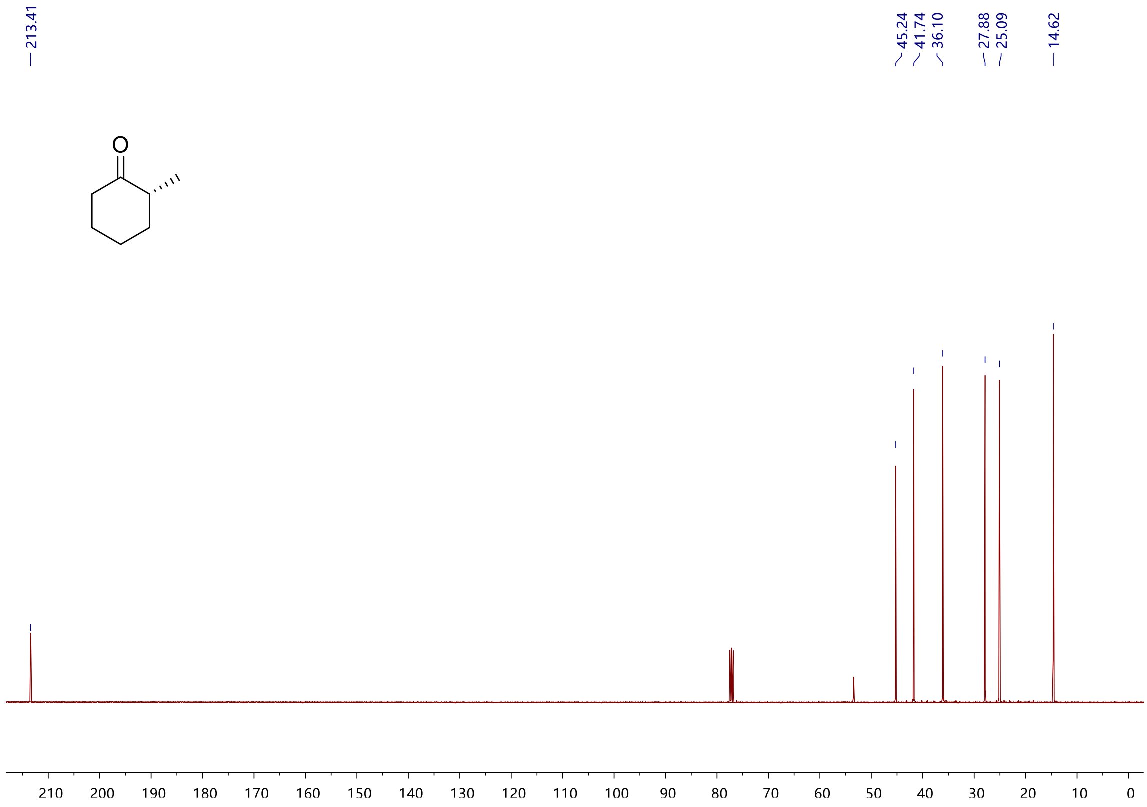
213.41
45.24
41.74
36.10
27.88
25.09
14.62
O
210
200
190
180
170
160
150
140
130
120
110
100
90
80
70
60
50
40
30
20
10
0

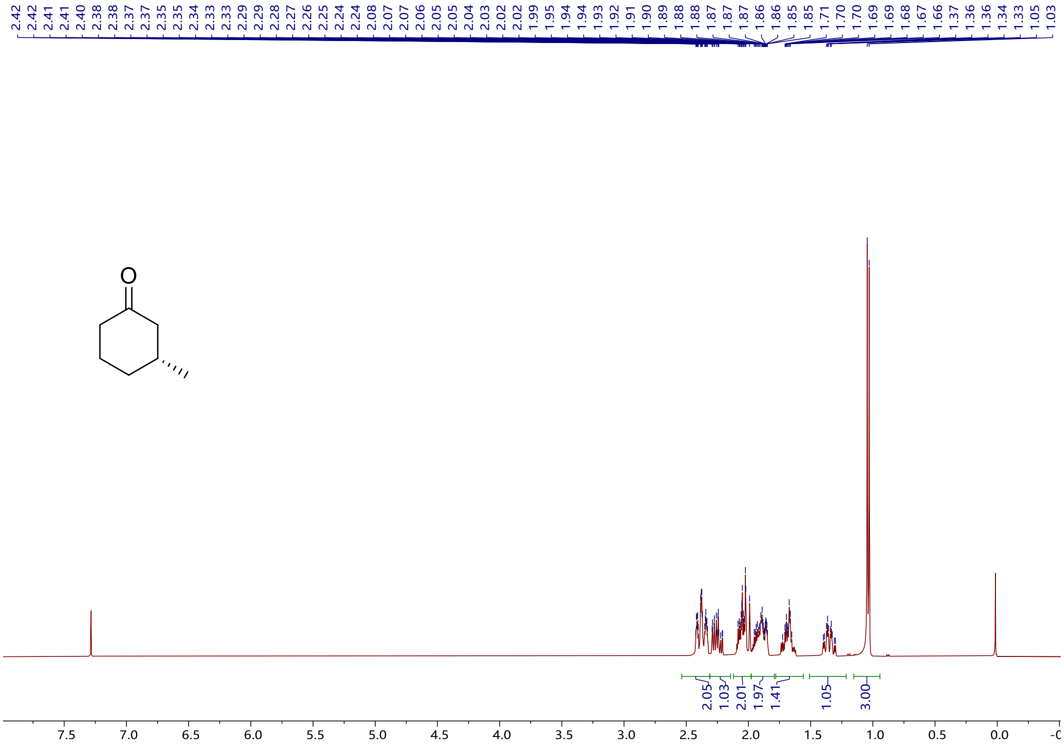
2.42
2.42
2.41
2.40
2.38
2.38
2.37
2.37
2.35
2.35
2.34
2.33
2.29
2.29
2.28
2.27
2.26
2.25
2.24
2.24
2.08
2.07
2.07
2.06
2.05
2.05
2.04
2.03
2.02
2.02
1.99
1.95
1.94
1.94
1.93
1.92
1.91
1.90
1.89
1.88
1.88
1.87
1.87
1.87
1.86
1.86
1.85
1.85
1.71
1.70
1.70
1.69
1.69
1.68
1.67
1.66
1.37
1.36
1.36
1.34
1.33
1.05
1.03
O
2.05
1.03
2.01
1.97
1.41
1.05
3.00
7.5
7.0
6.5
6.0
5.5
5.0
4.5
4.0
3.5
3.0
2.5
2.0
1.5
1.0
0.5
0.0

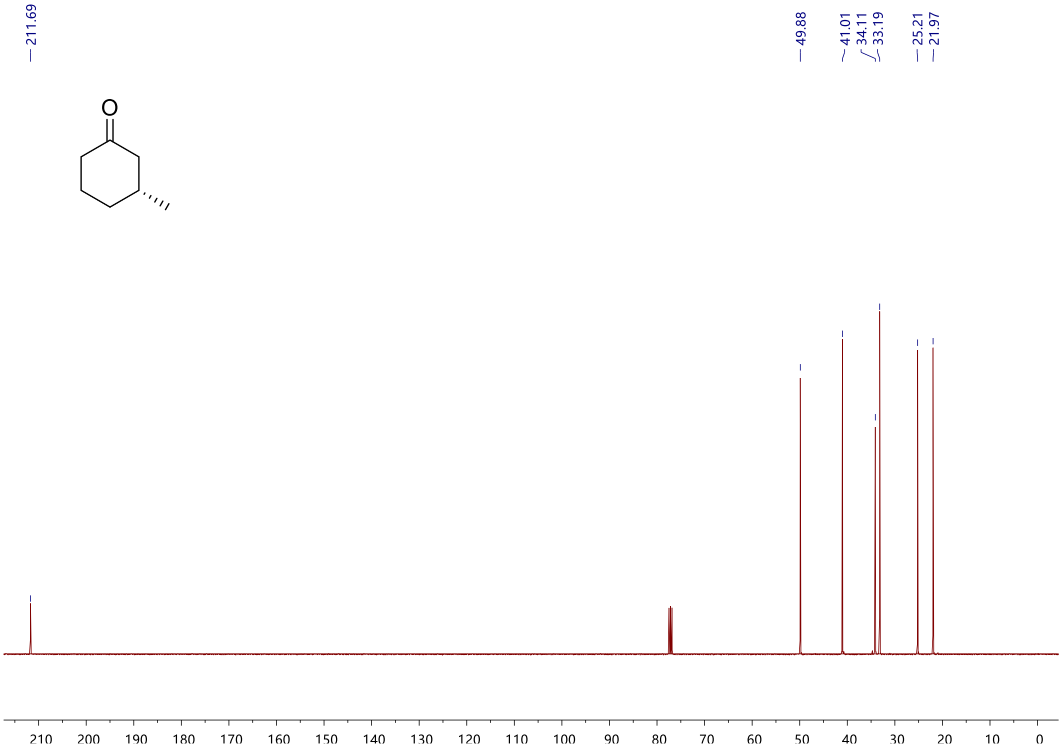
211.69
49.88
41.01
34.11
33.19
25.21
21.97
O
210
200
190
180
170
160
150
140
130
120
110
100
90
80
70
60
50
40
30
20
10
0

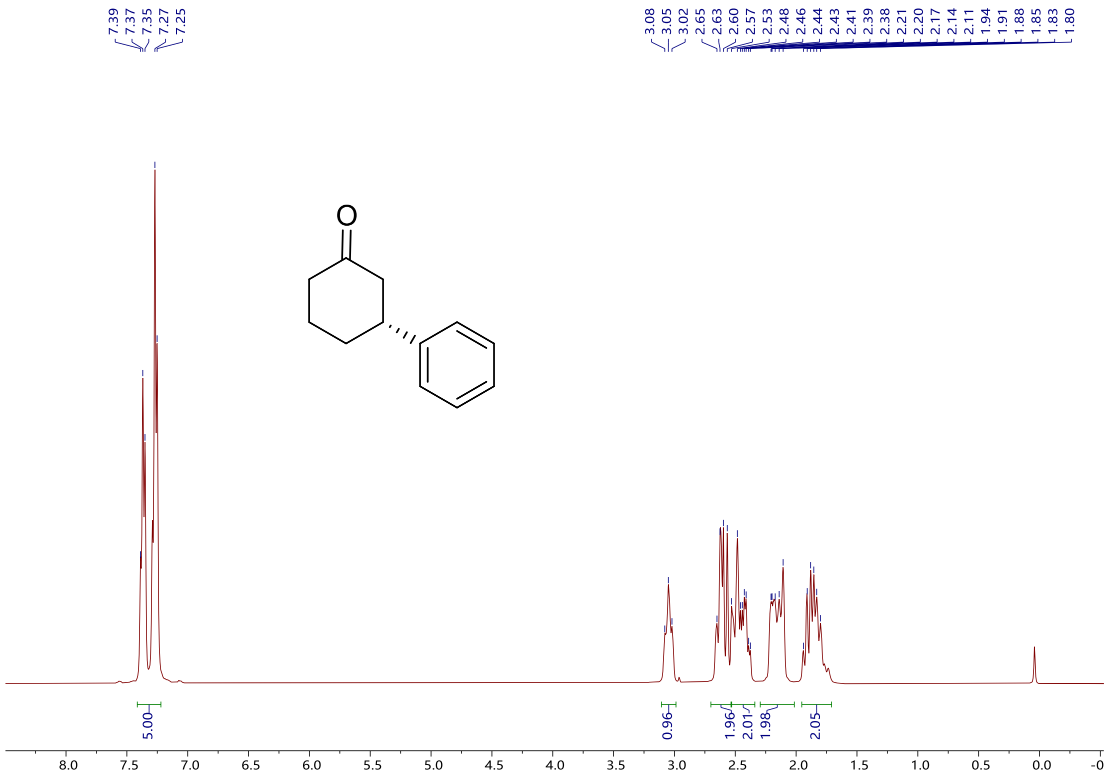

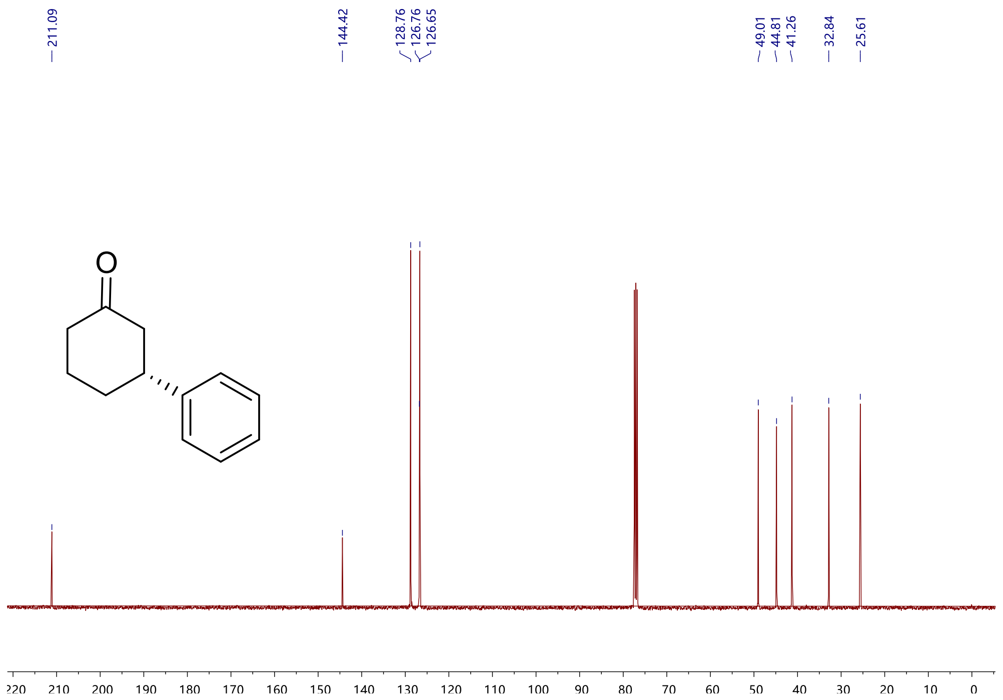

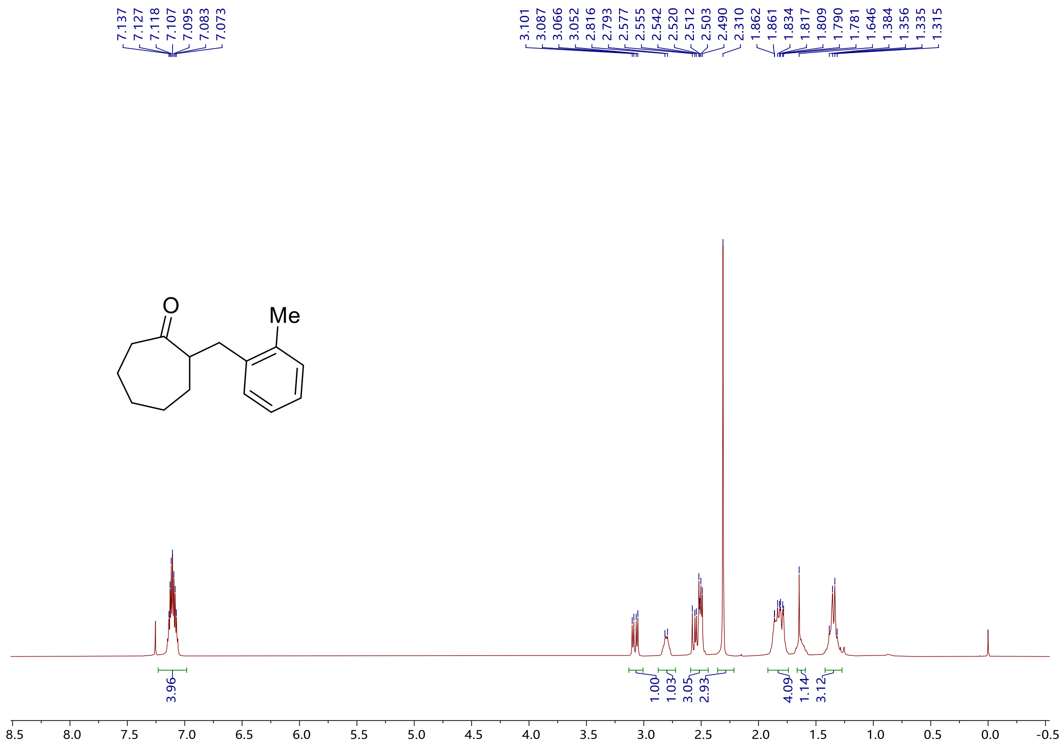
7.137
7.127
7.118
7.107
7.095
7.083
7.073
3.101
3.087
3.066
3.052
2.816
2.793
2.577
2.555
2.542
2.520
2.512
2.503
2.490
2.310
1.862
1.861
1.834
1.817
1.809
1.790
1.781
1.646
1.384
1.356
1.335
1.315
O
Me
3.96
1.00
1.03
3.05
2.93
4.09
1.14
3.12
8.5
8.0
7.5
7.0
6.5
6.0
5.5
5.0
4.5
4.0
3.5
3.0
2.5
2.0
1.5
1.0
0.5
0.0
-0.5

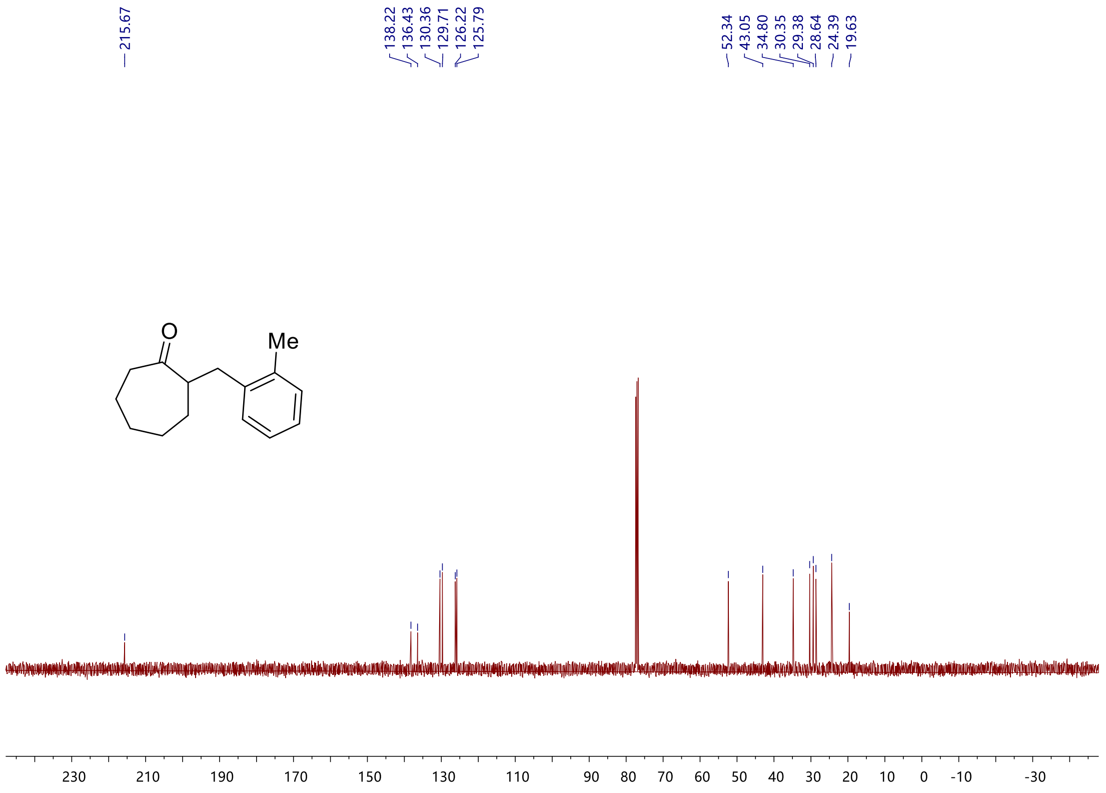
215.67
138.22
136.43
130.36
129.71
126.22
125.79
52.34
43.05
34.80
30.35
29.38
28.64
24.39
19.63
O
Me
230
210
190
170
150
130
110
90
80
70
60
50
40
30
20
10
0
-10
-30

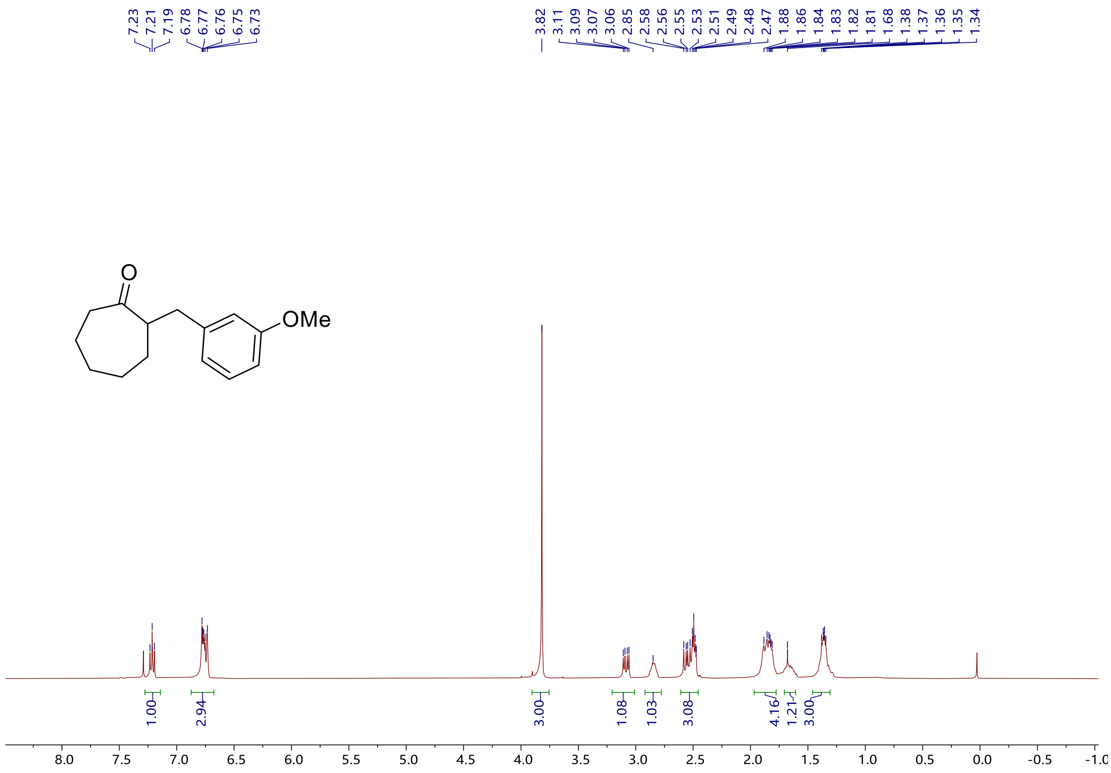
7.23
7.21
7.19
6.78
6.77
6.76
6.75
6.73
3.82
3.11
3.09
3.07
3.06
2.85
2.58
2.56
2.55
2.53
2.51
2.49
2.48
2.47
1.88
1.86
1.84
1.83
1.82
1.81
1.68
1.38
1.37
1.36
1.35
1.34
OMe
1.00
2.94
3.00
1.08
1.03
3.08
4.16
1.21
3.00

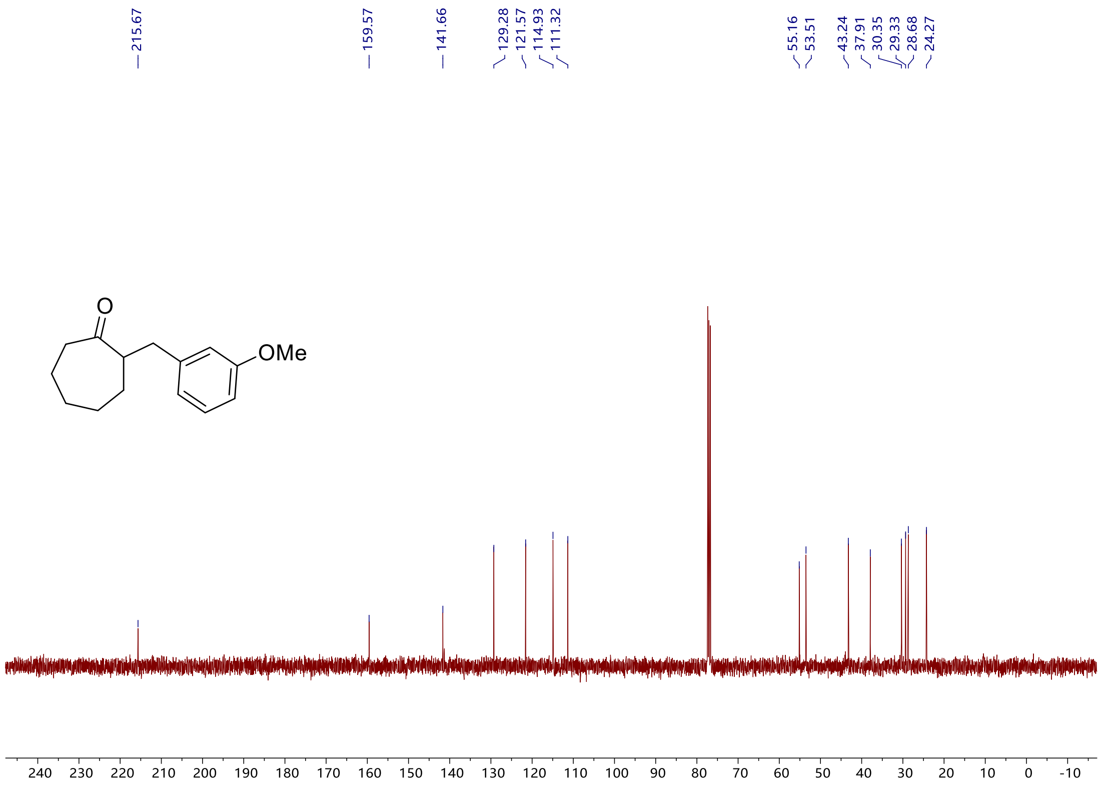
215.67
159.57
141.66
129.28
121.57
114.93
111.32
55.16
53.51
43.24
37.91
30.35
29.33
28.68
24.27
OMe

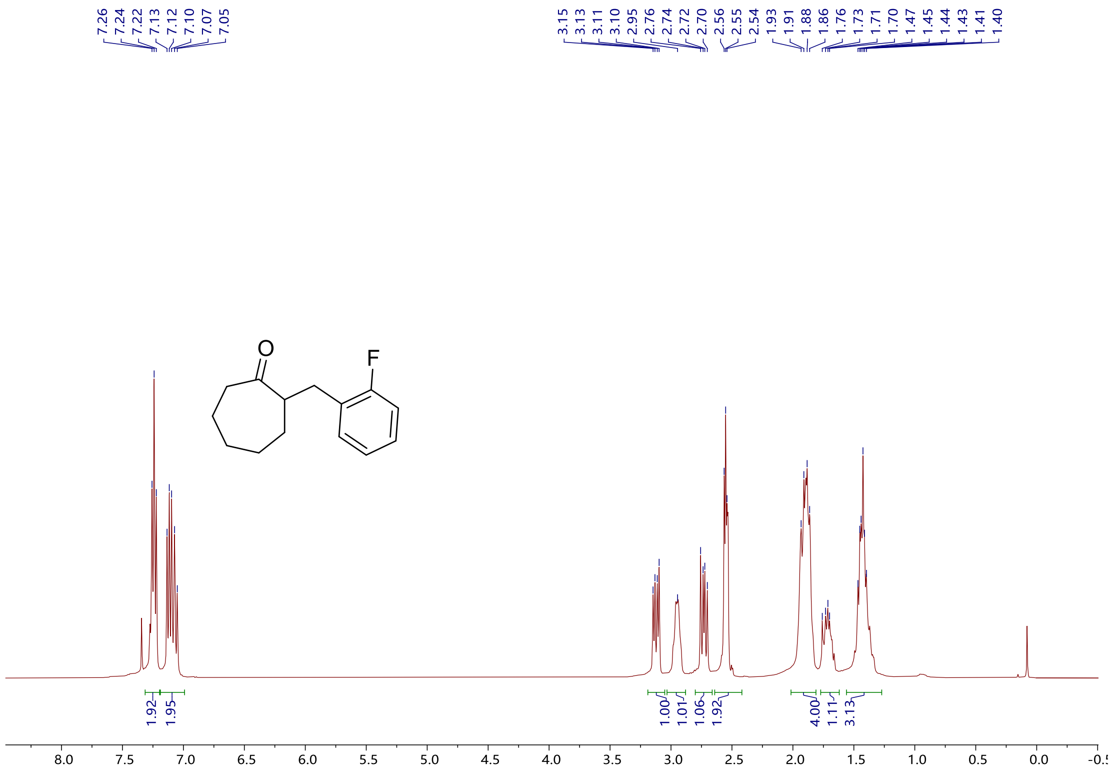
7.26
7.24
7.22
7.13
7.12
7.10
7.07
7.05
3.15
3.13
3.11
3.10
2.95
2.76
2.74
2.72
2.70
2.56
2.55
2.54
1.93
1.91
1.88
1.86
1.76
1.73
1.71
1.70
1.47
1.45
1.44
1.43
1.41
1.40
O
F
1.92
1.95
1.00
1.01
1.06
1.92
4.00
1.11
3.13
8.0
7.5
7.0
6.5
6.0
5.5
5.0
4.5
4.0
3.5
3.0
2.5
2.0
1.5
1.0
0.5
0.0

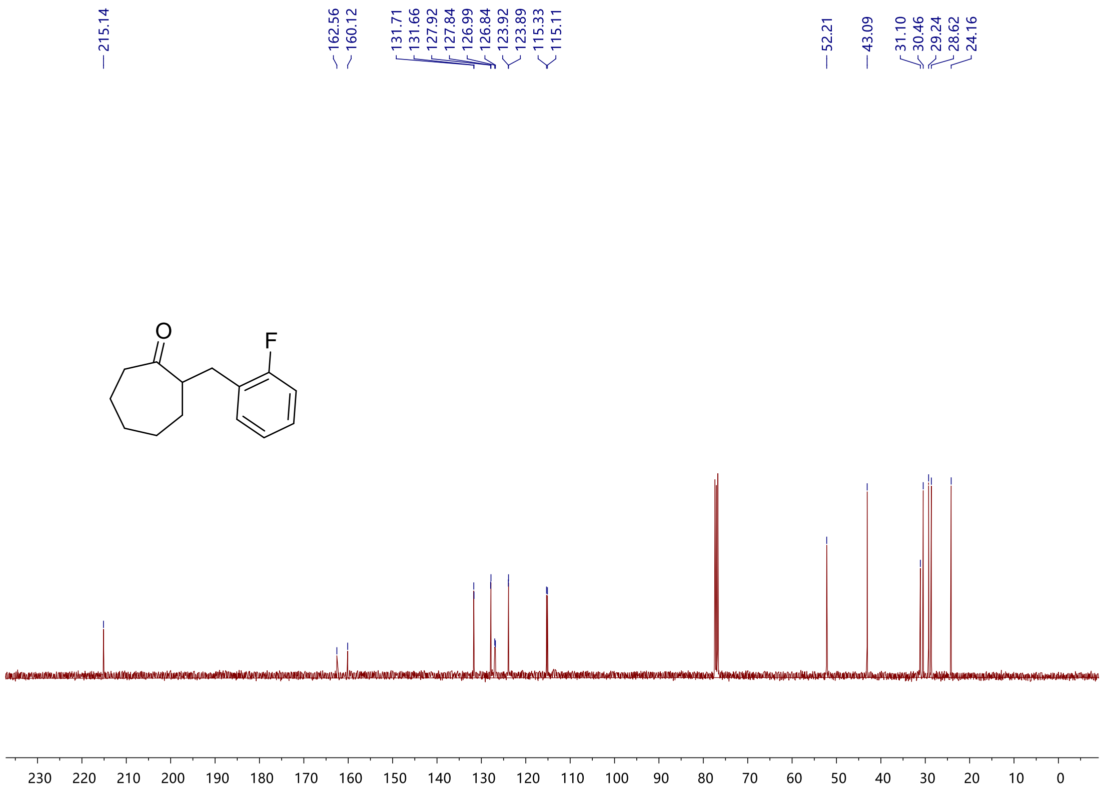
215.14
162.56
160.12
131.71
131.66
127.92
127.84
126.99
126.84
123.92
123.89
115.33
115.11
52.21
43.09
31.10
30.46
29.24
28.62
24.16
O
F
230
220
210
200
190
180
170
160
150
140
130
120
110
100
90
80
70
60
50
40
30
20
10
0

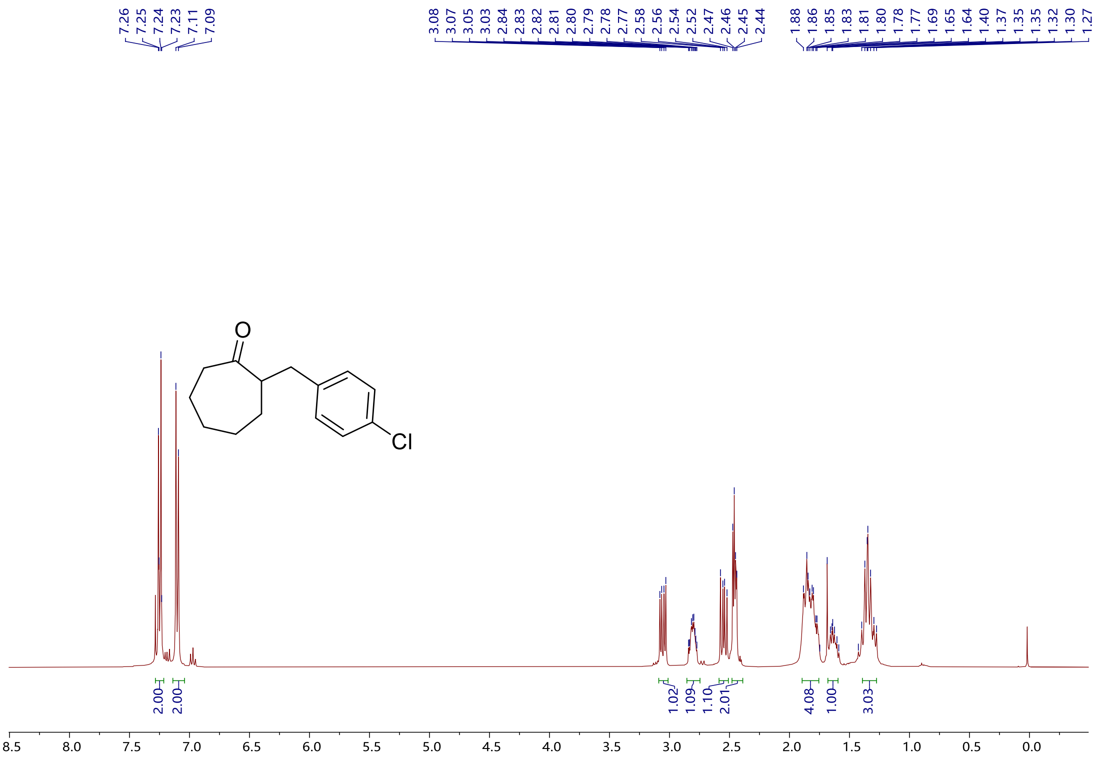

7.26
7.25
7.24
7.23
7.11
7.09
3.08
3.07
3.05
3.03
2.84
2.83
2.82
2.81
2.80
2.79
2.78
2.77
2.58
2.56
2.54
2.52
2.47
2.46
2.45
2.44
1.88
1.86
1.85
1.83
1.81
1.80
1.78
1.77
1.69
1.65
1.64
1.40
1.37
1.35
1.32
1.30
1.27
O
Cl
2.00
2.00
1.02
1.09
1.10
2.01
4.08
1.00
3.03
8.5
8.0
7.5
7.0
6.5
6.0
5.5
5.0
4.5
4.0
3.5
3.0
2.5
2.0
1.5
1.0
0.5
0.0

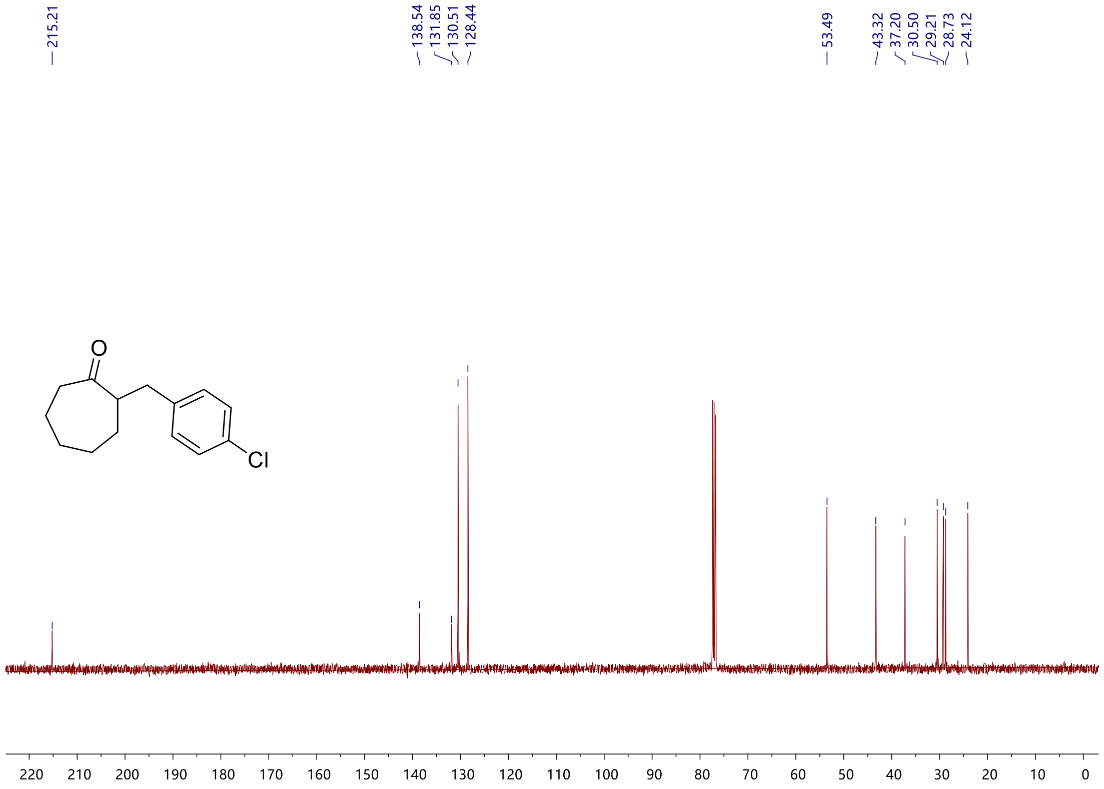

215.21
138.54
131.85
130.51
128.44
53.49
43.32
37.20
30.50
29.21
28.73
24.12
O
Cl
220
210
200
190
180
170
160
150
140
130
120
110
100
90
80
70
60
50
40
30
20
10
0

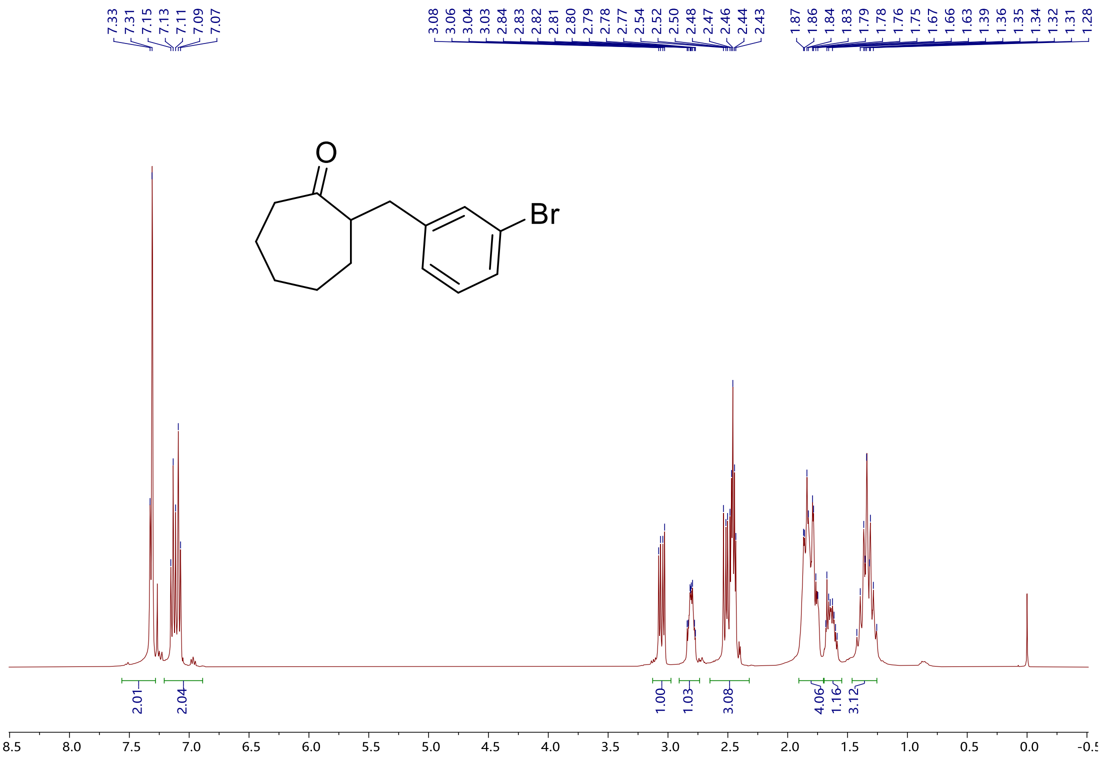
7.33
7.31
7.15
7.13
7.11
7.09
7.07
3.08
3.06
3.04
3.03
2.84
2.83
2.82
2.81
2.80
2.79
2.78
2.77
2.54
2.52
2.50
2.48
2.47
2.46
2.44
2.43
1.87
1.86
1.84
1.83
1.79
1.78
1.76
1.75
1.67
1.66
1.63
1.39
1.36
1.35
1.34
1.32
1.31
1.28
Br
2.01
2.04
1.00
1.03
3.08
4.06
1.16
3.12
8.5 8.0 7.5 7.0 6.5 6.0 5.5 5.0 4.5 4.0 3.5 3.0 2.5 2.0 1.5 1.0 0.5 0.0

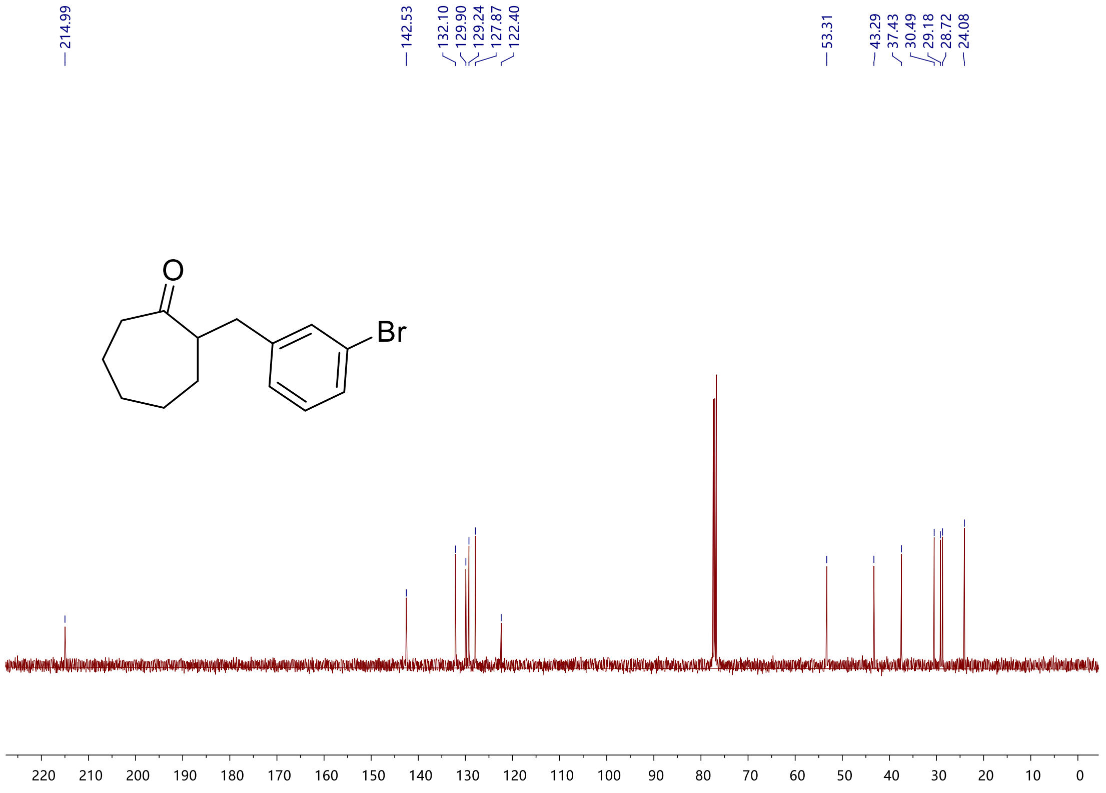
214.99
142.53
132.10
129.90
129.24
127.87
122.40
53.31
43.29
37.43
30.49
29.18
28.72
24.08
Br
220 210 200 190 180 170 160 150 140 130 120 110 100 90 80 70 60 50 40 30 20 10 0

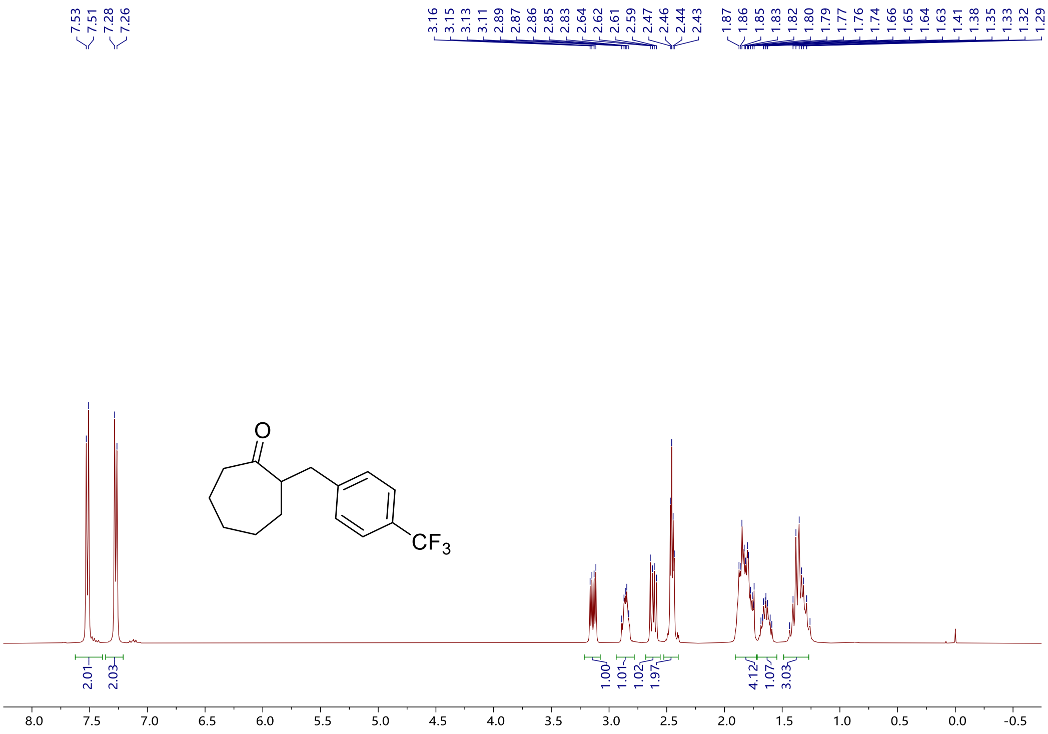
7.53
7.51
7.28
7.26
3.16
3.15
3.13
3.11
2.89
2.87
2.86
2.85
2.83
2.64
2.62
2.61
2.59
2.47
2.46
2.44
2.43
1.87
1.86
1.85
1.83
1.82
1.80
1.79
1.77
1.76
1.74
1.66
1.65
1.64
1.63
1.41
1.38
1.35
1.33
1.32
1.29
O
CF3
2.01
2.03
1.00
1.01
1.02
1.97
4.12
1.07
3.03
8.0 7.5 7.0 6.5 6.0 5.5 5.0 4.5 4.0 3.5 3.0 2.5 2.0 1.5 1.0 0.5 0.0 -0.5

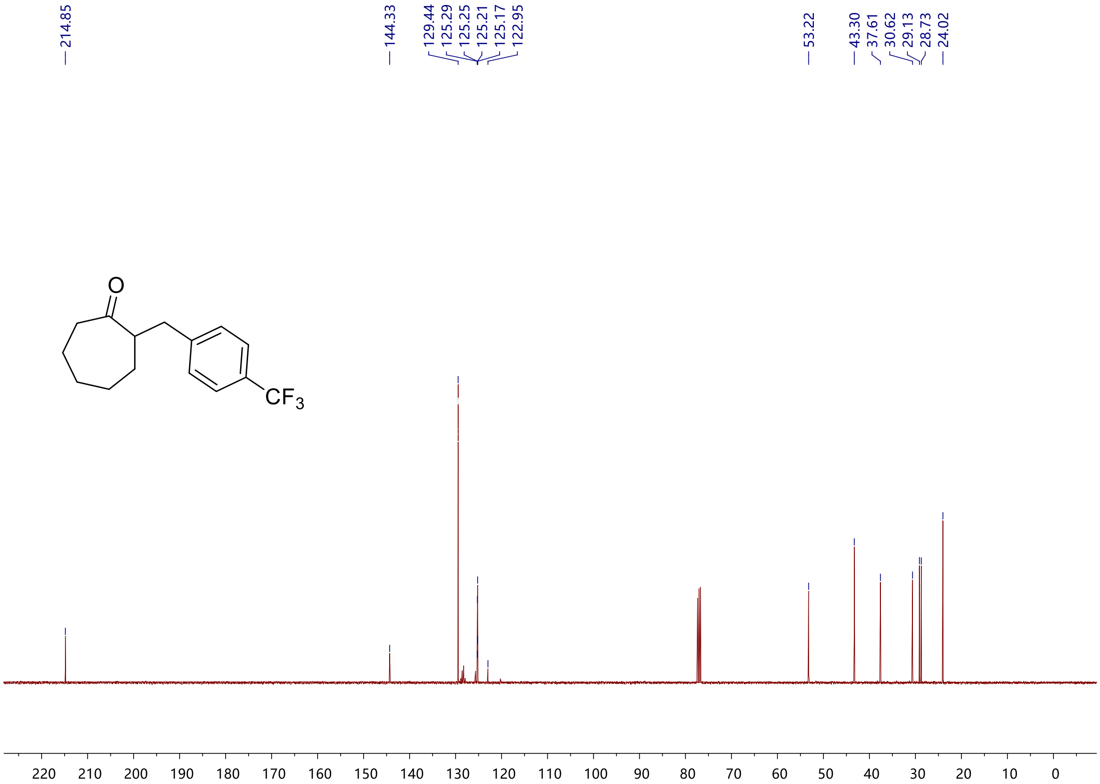
214.85
144.33
129.44
125.29
125.25
125.21
125.17
122.95
53.22
43.30
37.61
30.62
29.13
28.73
24.02
O
CF3
220 210 200 190 180 170 160 150 140 130 120 110 100 90 80 70 60 50 40 30 20 10 0

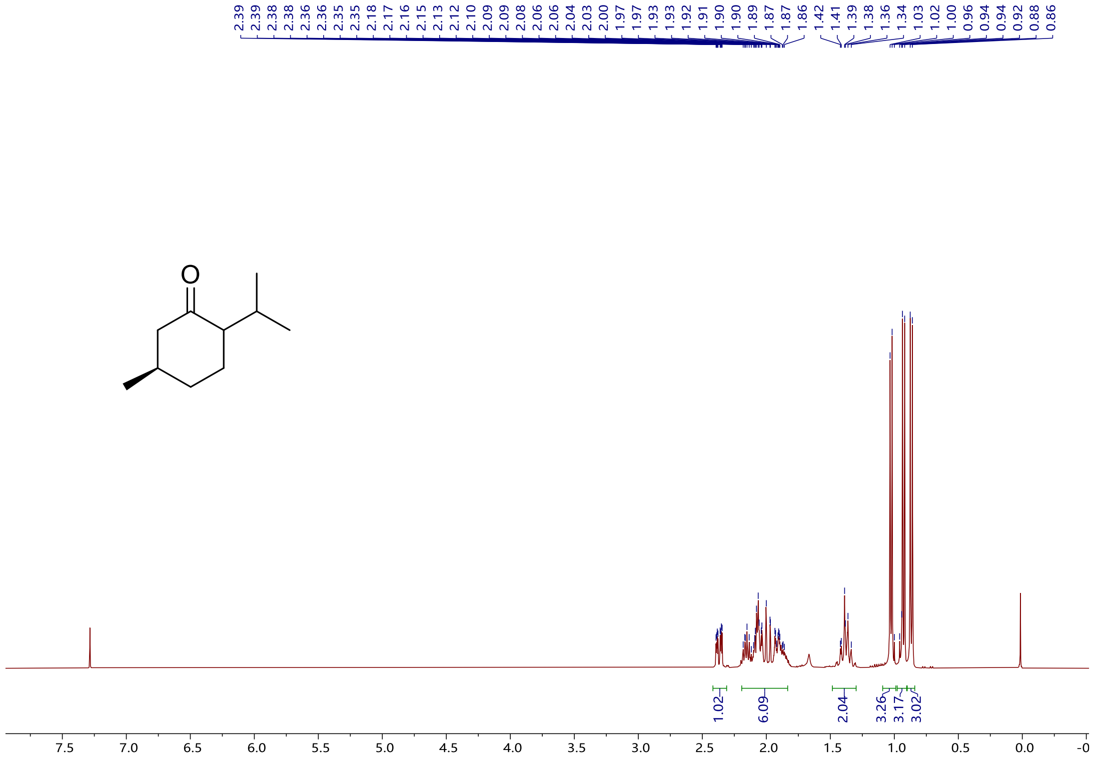

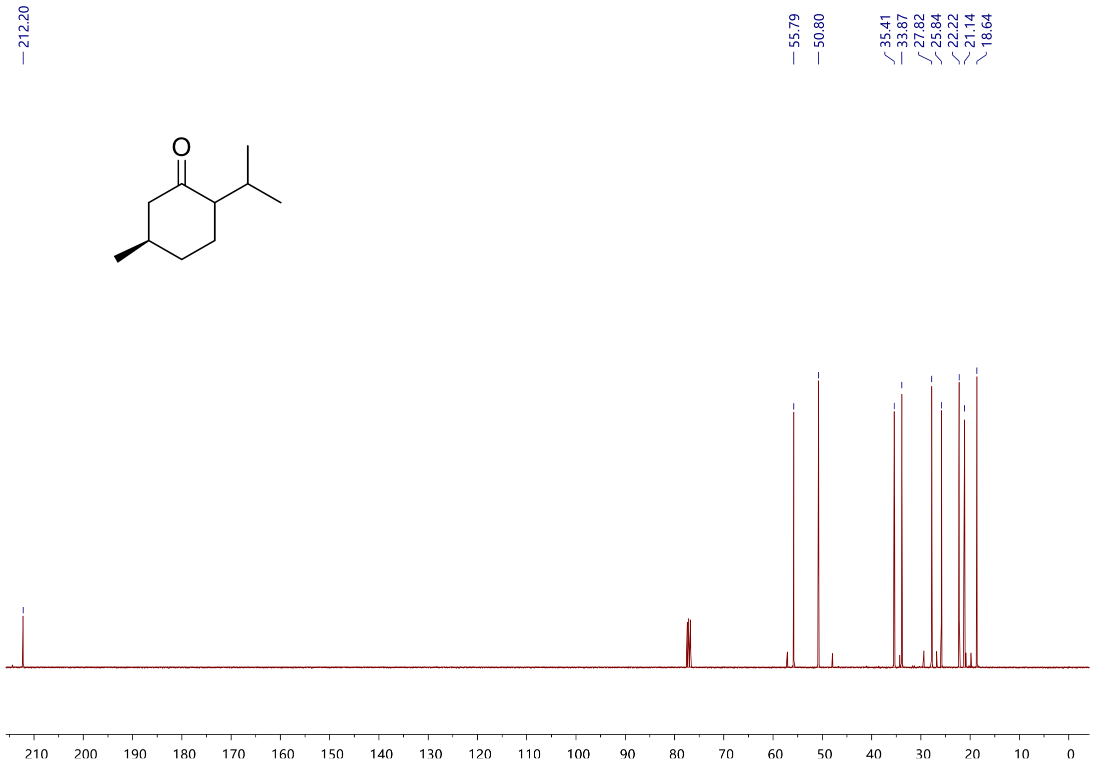

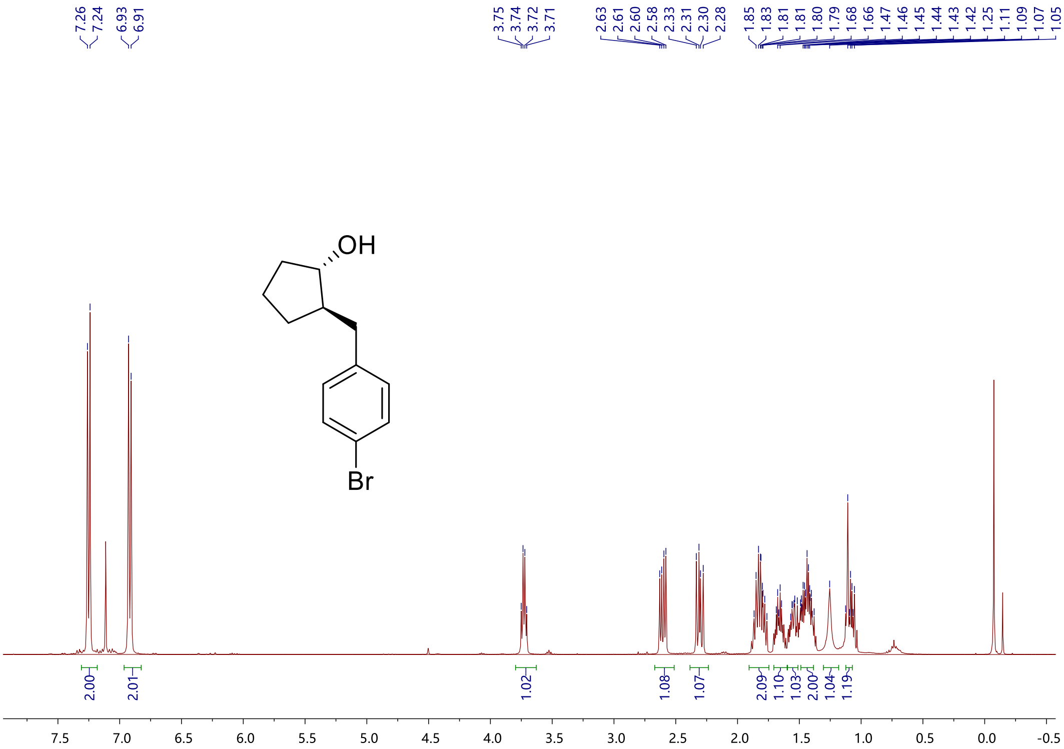
7.26
7.24
6.93
6.91
3.75
3.74
3.72
3.71
2.63
2.61
2.60
2.58
2.33
2.31
2.30
2.28
1.85
1.83
1.81
1.81
1.80
1.79
1.68
1.66
1.47
1.46
1.45
1.44
1.43
1.42
1.25
1.11
1.09
1.07
1.05
OH
Br
2.00
2.01
1.02
1.08
1.07
2.09
1.10
1.03
2.00
1.04
1.19

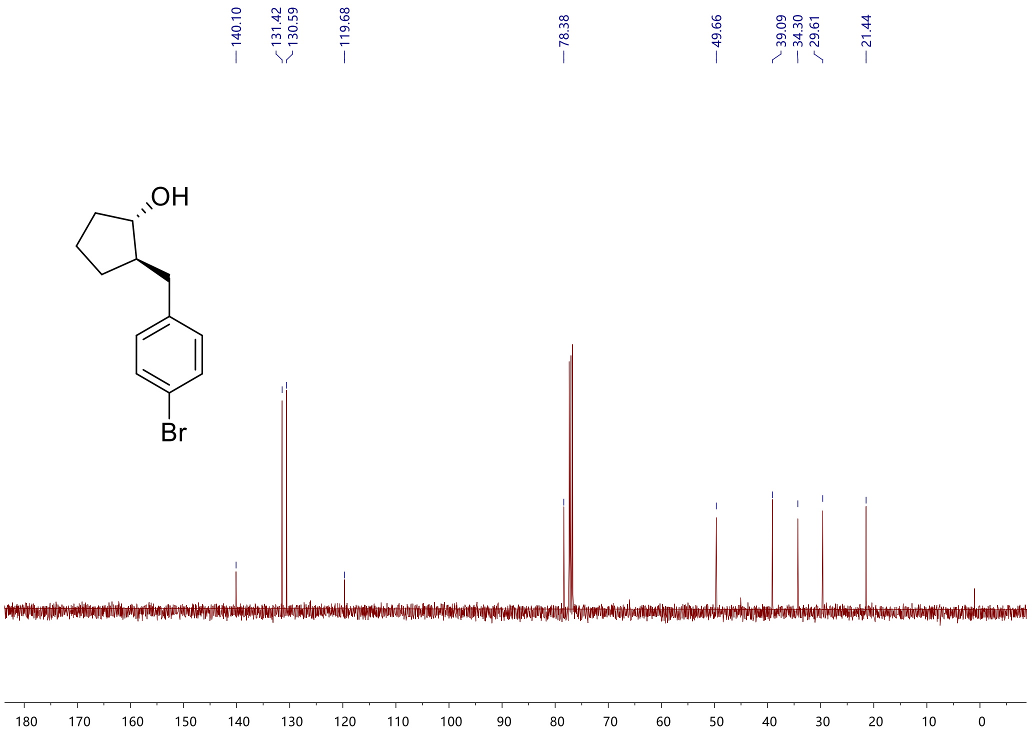
140.10
131.42
130.59
119.68
78.38
49.66
39.09
34.30
29.61
21.44
OH
Br

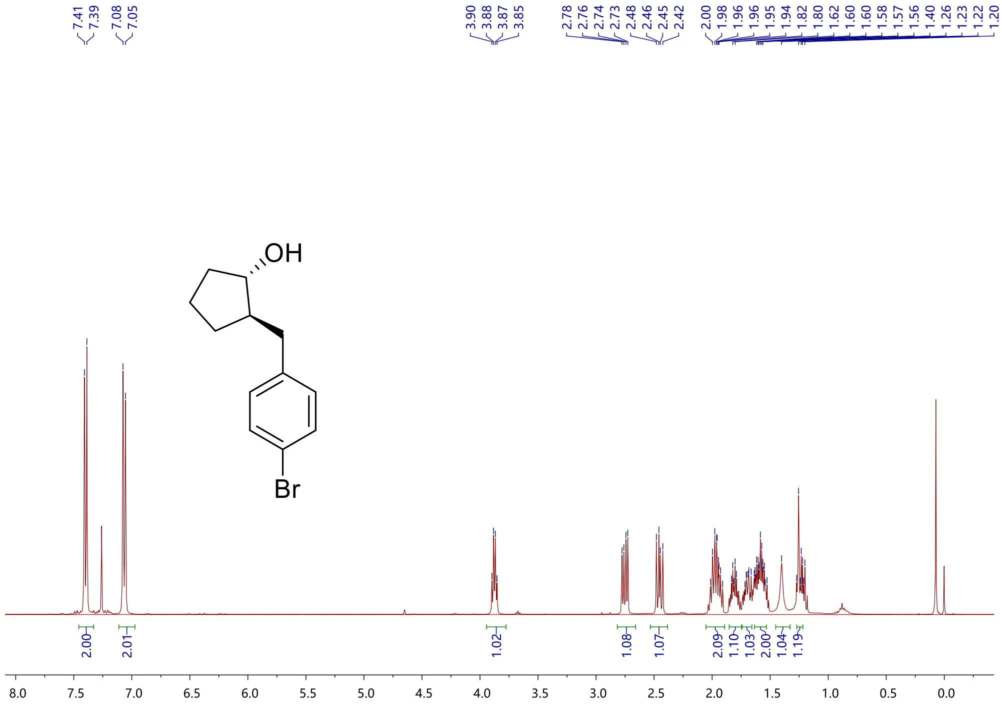
7.41
7.39
7.08
7.05
3.90
3.88
3.87
3.85
2.78
2.76
2.74
2.73
2.48
2.46
2.45
2.42
2.00
1.98
1.96
1.96
1.95
1.94
1.82
1.80
1.62
1.60
1.60
1.58
1.57
1.56
1.40
1.26
1.23
1.22
1.20
OH
Br
2.00
2.01
1.02
1.08
1.07
2.09
1.10
1.03
2.00
1.04
1.19
8.0
7.5
7.0
6.5
6.0
5.5
5.0
4.5
4.0
3.5
3.0
2.5
2.0
1.5
1.0
0.5
0.0

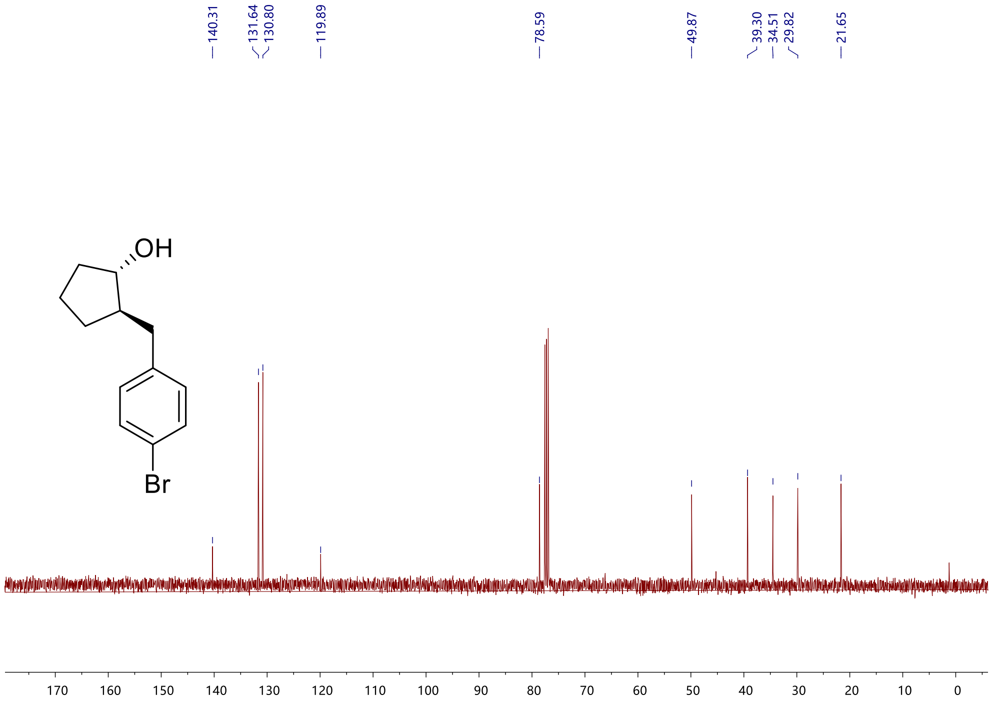
140.31
131.64
130.80
119.89
78.59
49.87
39.30
34.51
29.82
21.65
OH
Br
170
160
150
140
130
120
110
100
90
80
70
60
50
40
30
20
10
0

GC/HPLC spectra

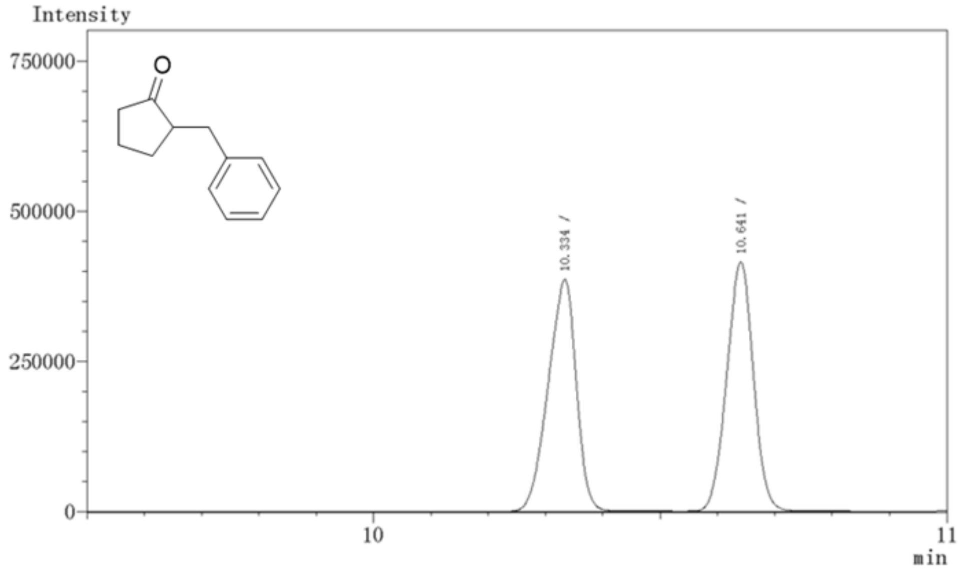

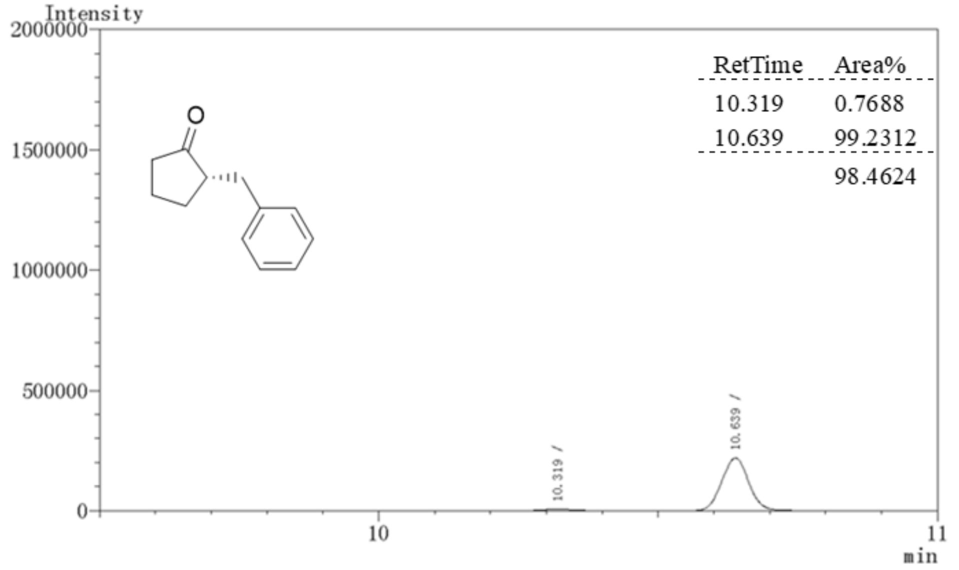

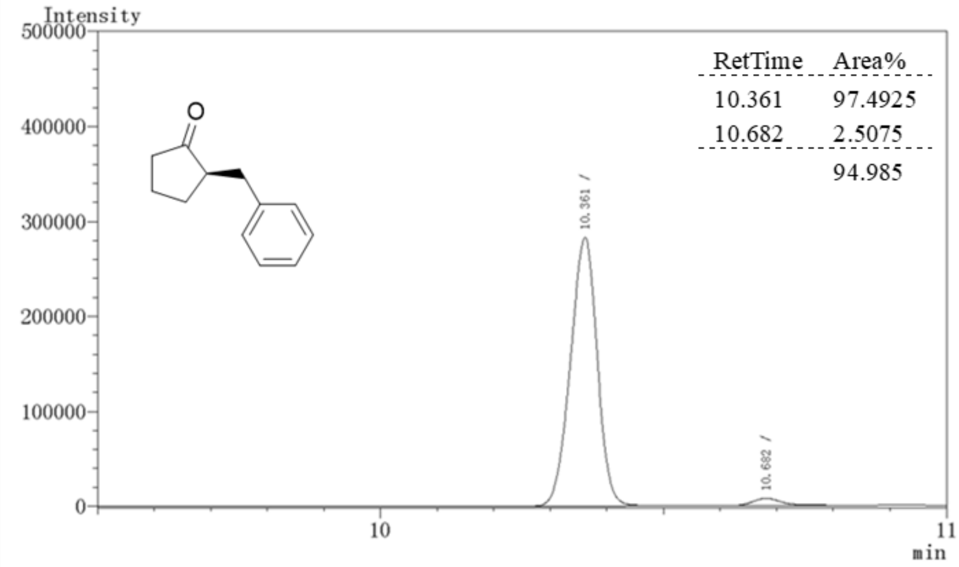

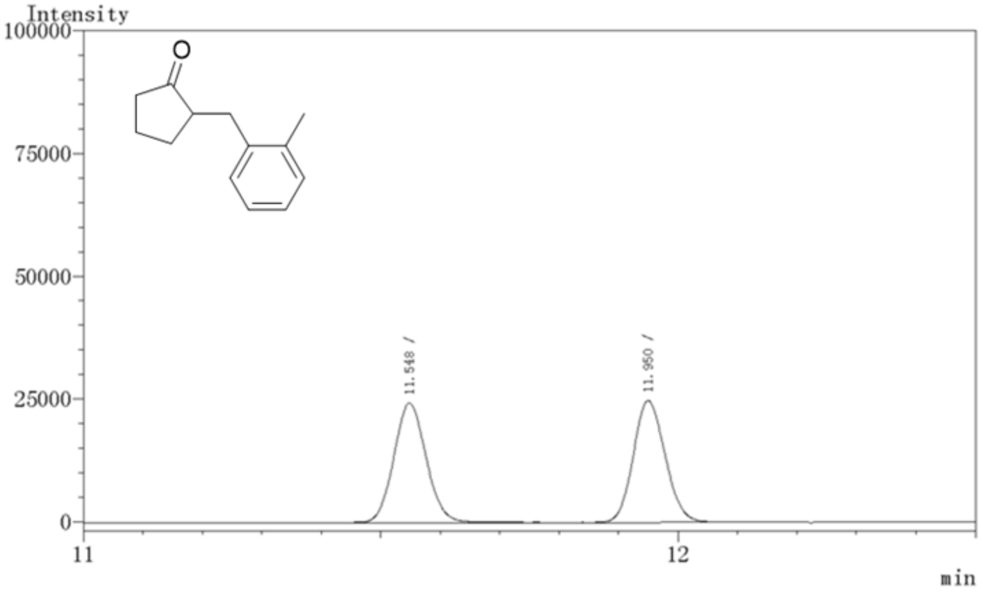
Intensity
100000
75000
50000
25000
0
11.548
11.950
11
12
min
O

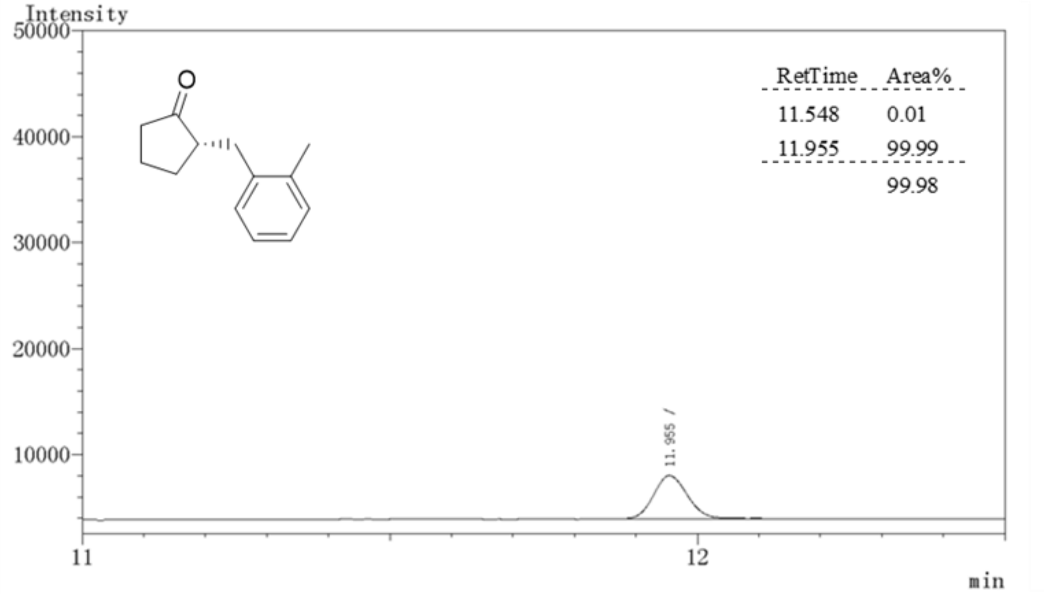
Intensity
50000
40000
30000
20000
10000
RetTime Area%
11.548 0.01
11.955 99.99
99.98
11.955
11
12
min
O

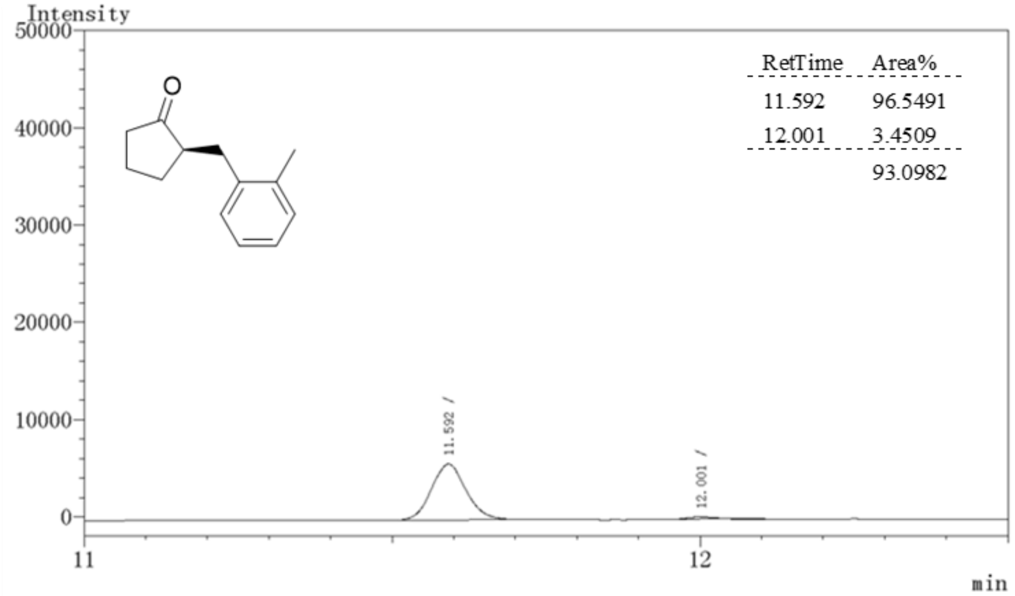
Intensity
50000
40000
30000
20000
10000
0
RetTime Area%
11.592 96.5491
12.001 3.4509
93.0982
11.592
12.001
11
12
min
O

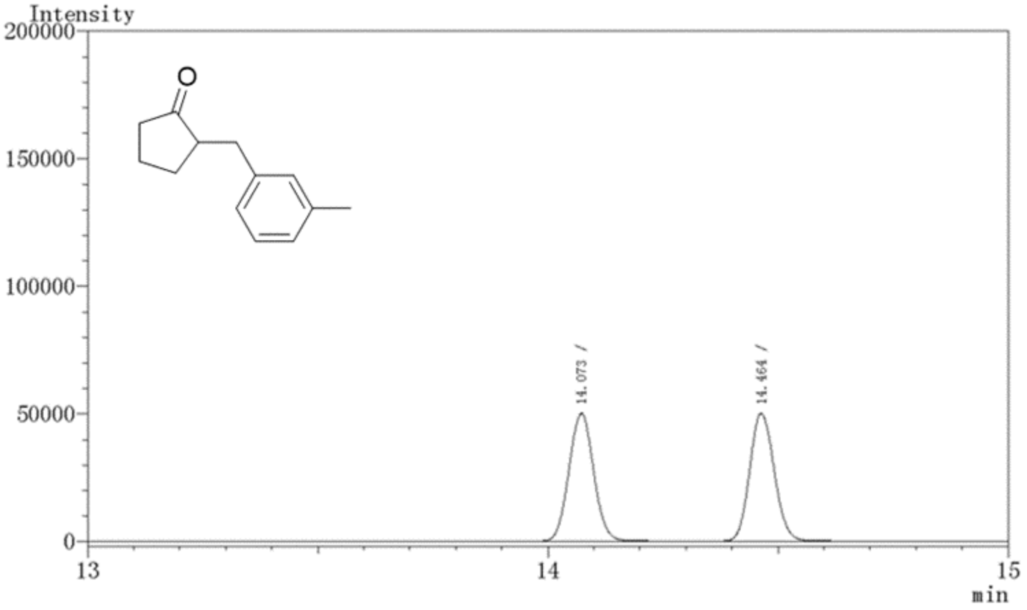
Intensity
200000
150000
100000
50000
0
14.073
14.464
13
14
15
min

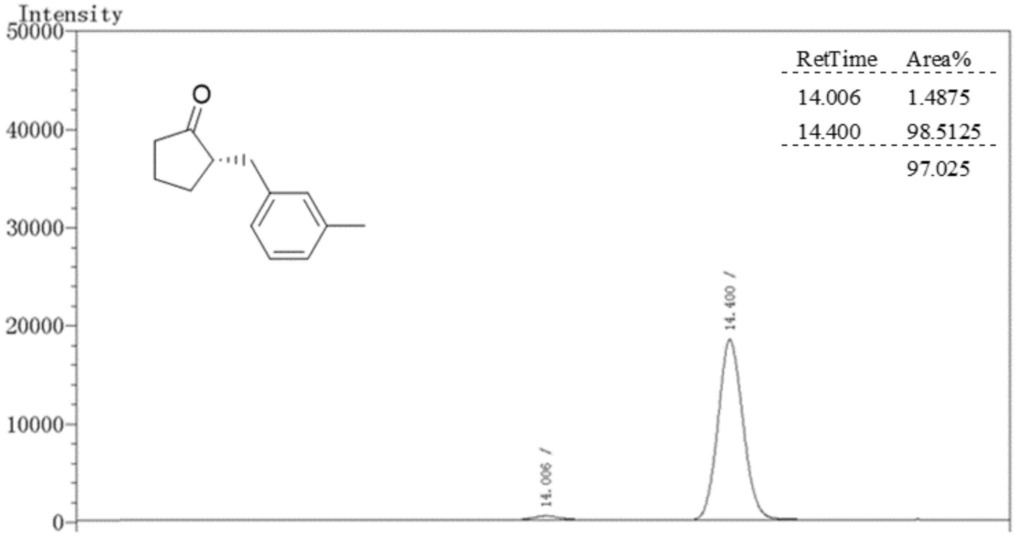
Intensity
50000
40000
30000
20000
10000
0
RetTime Area%
14.006 1.4875
14.400 98.5125
97.025
14.006
14.400

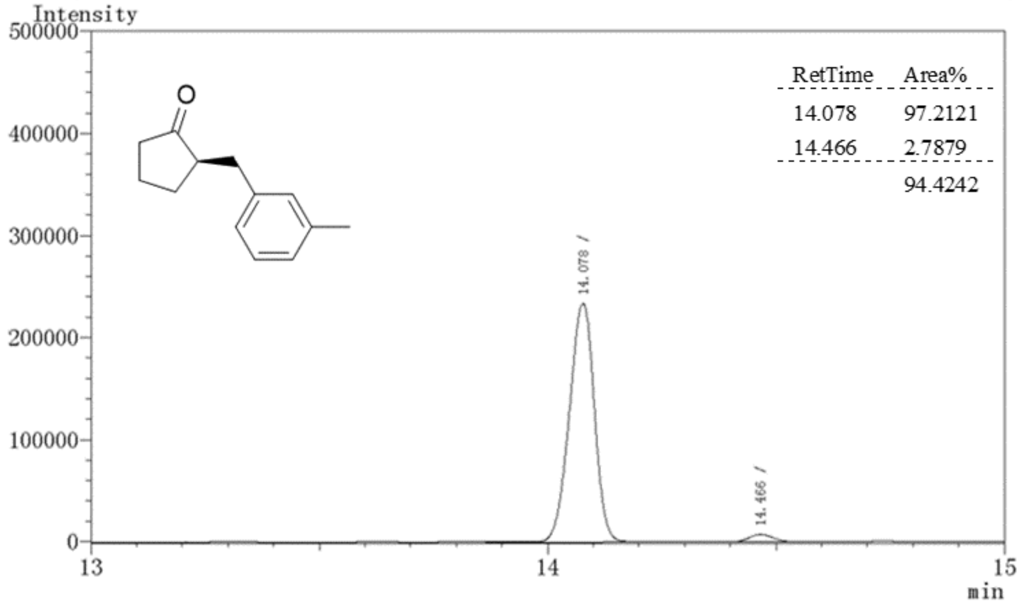
Intensity
500000
400000
300000
200000
100000
0
RetTime Area%
14.078 97.2121
14.466 2.7879
94.4242
14.078
14.466
13
14
15
min

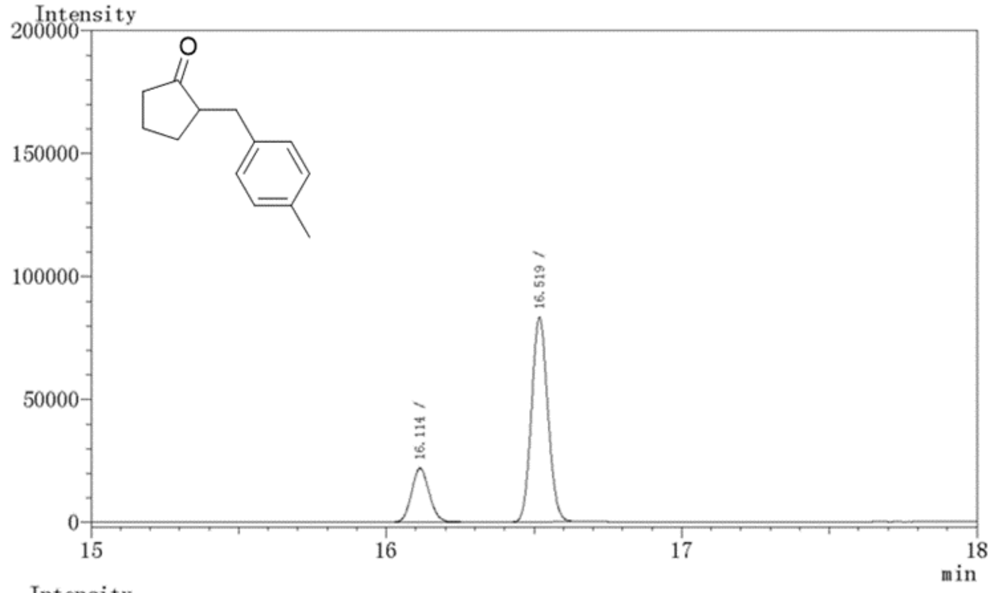

Intensity
200000
150000
100000
50000
0
16.114
16.519
15
16
17
18
min

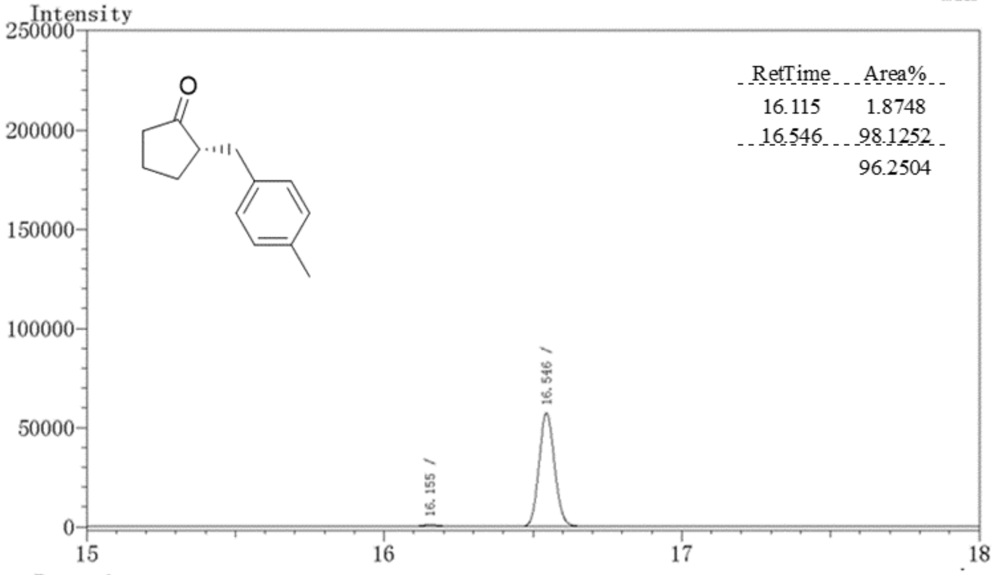

Intensity
250000
200000
150000
100000
50000
0
RetTime Area%
16.115 1.8748
16.546 98.1252
96.2504
16.155
16.546
15
16
17
18

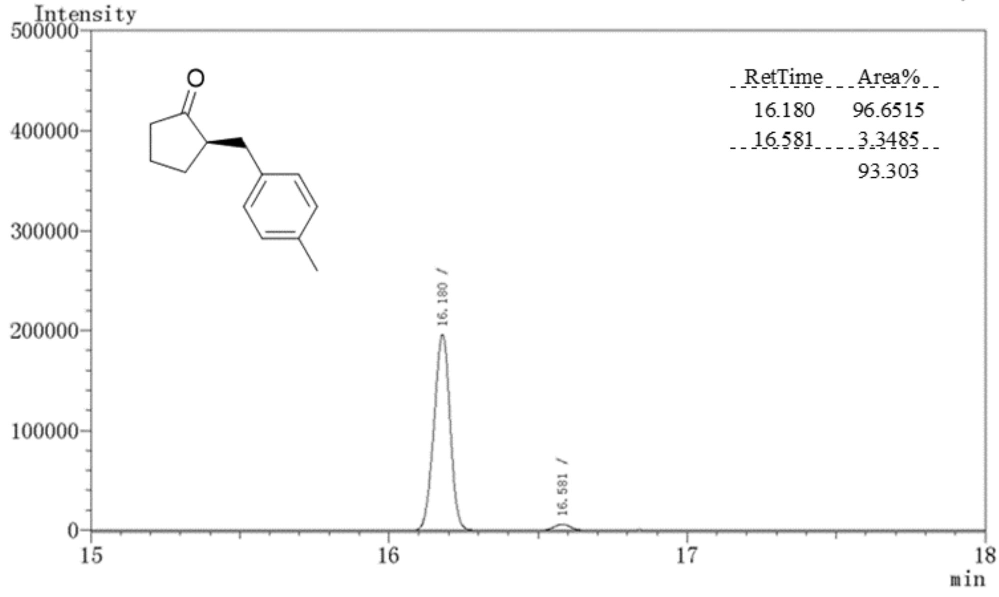

Intensity
500000
400000
300000
200000
100000
0
RetTime Area%
16.180 96.6515
16.581 3.3485
93.303
16.180
16.581
15
16
17
18
min

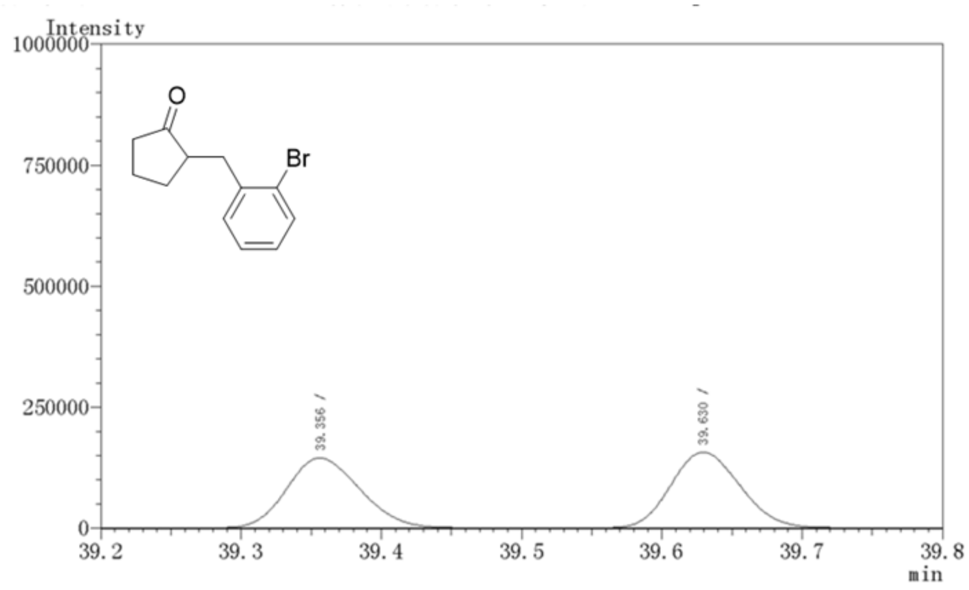
Intensity
O
Br
39.356
39.630
min

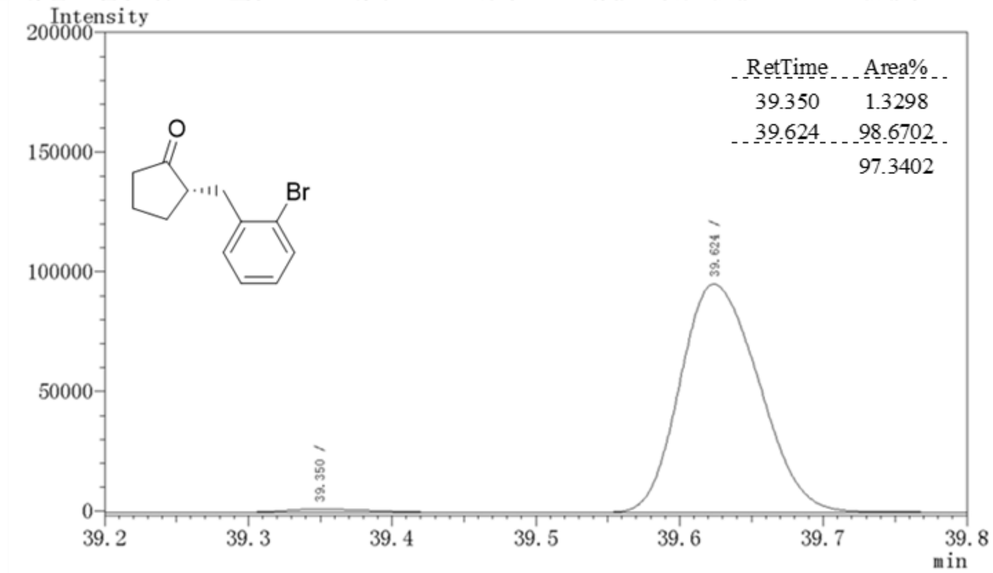
Intensity
RetTime Area%
39.350 1.3298
39.624 98.6702
97.3402
O
Br
39.624
39.350
min

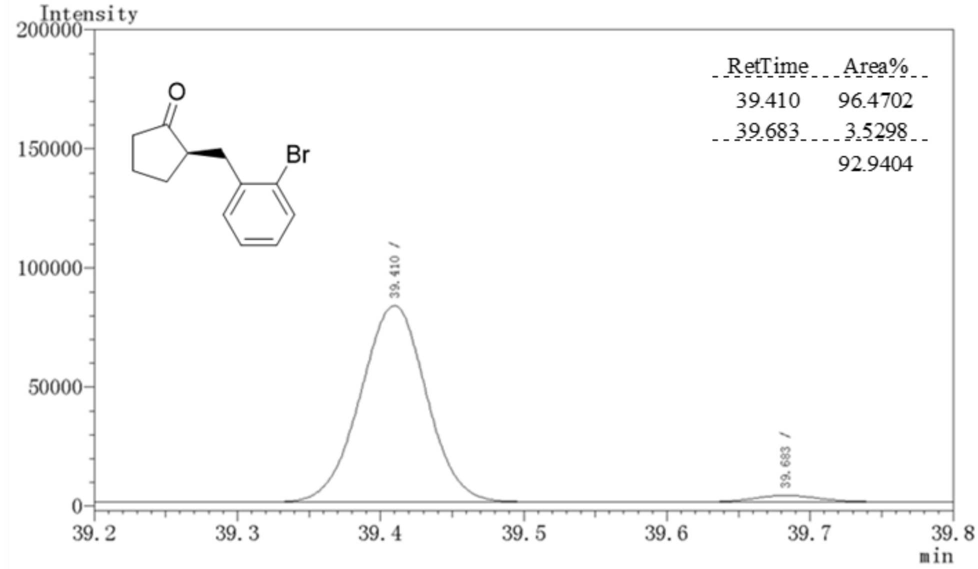
Intensity
RetTime Area%
39.410 96.4702
39.683 3.5298
92.9404
O
Br
39.410
39.683
min

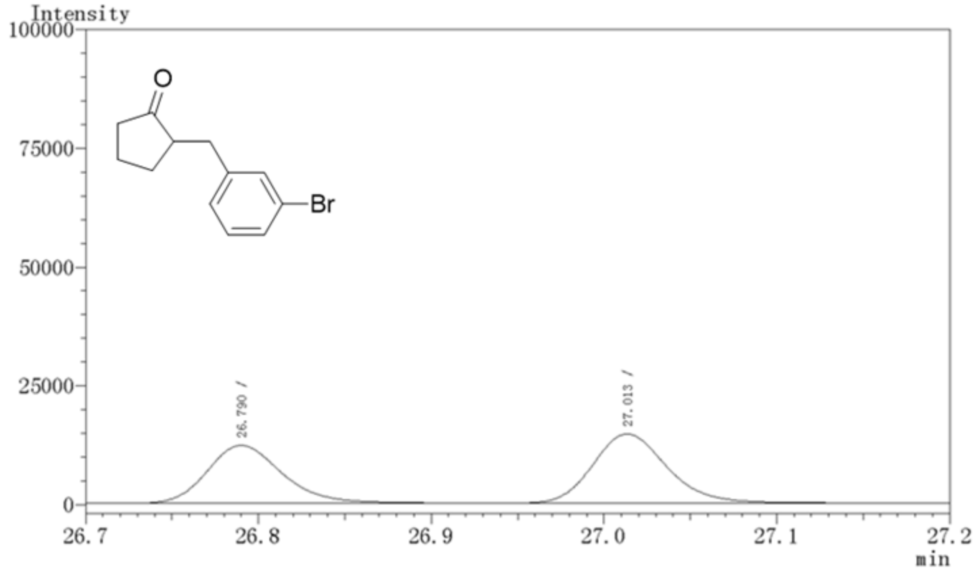
Intensity
100000
75000
50000
25000
0
26.790
27.013
Br
O
26.7
26.8
26.9
27.0
27.1
27.2
min

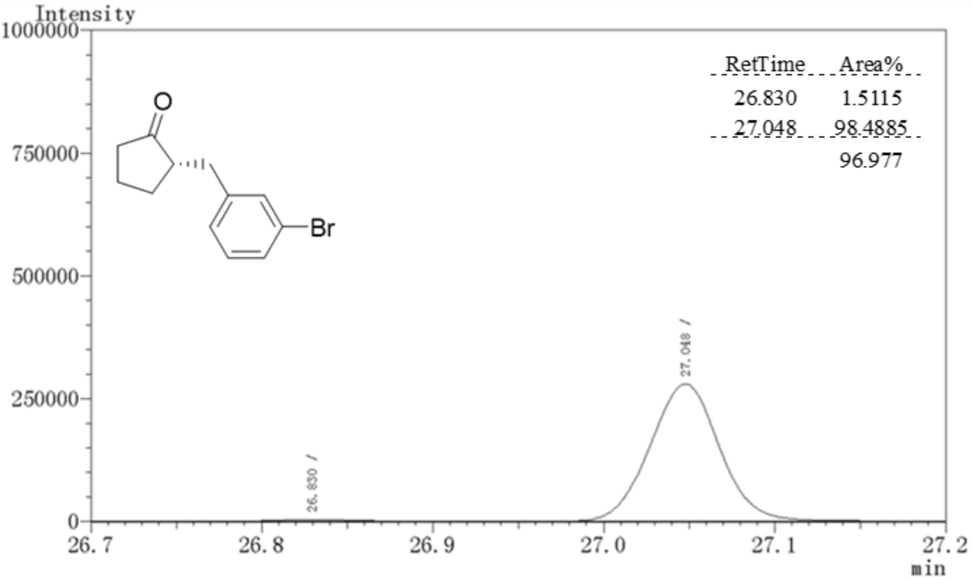
Intensity
1000000
750000
500000
250000
0
RetTime Area%
26.830 1.5115
27.048 98.4885
96.977
26.830
27.048
Br
O
26.7
26.8
26.9
27.0
27.1
27.2
min

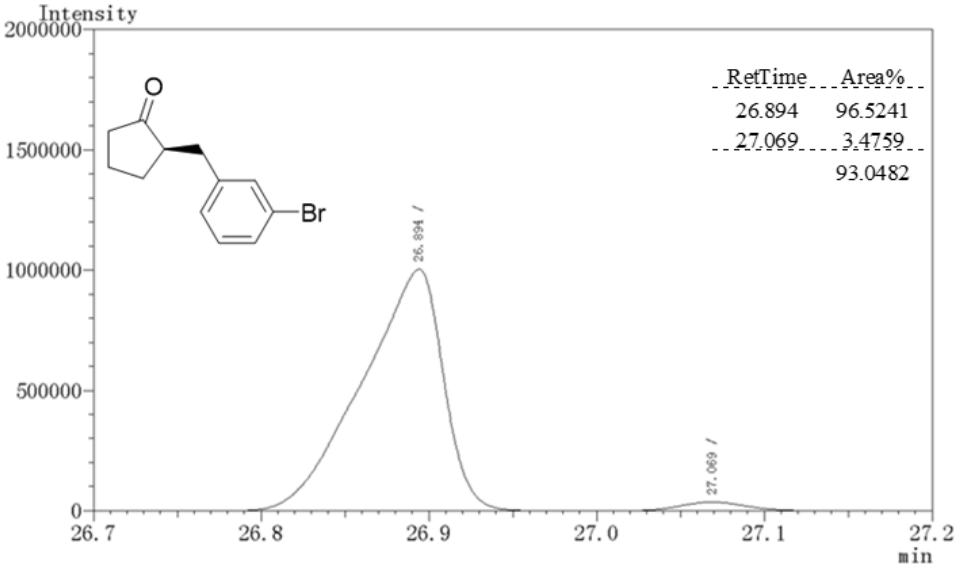
Intensity
2000000
1500000
1000000
500000
0
RetTime Area%
26.894 96.5241
27.069 3.4759
93.0482
26.894
27.069
Br
O
26.7
26.8
26.9
27.0
27.1
27.2
min

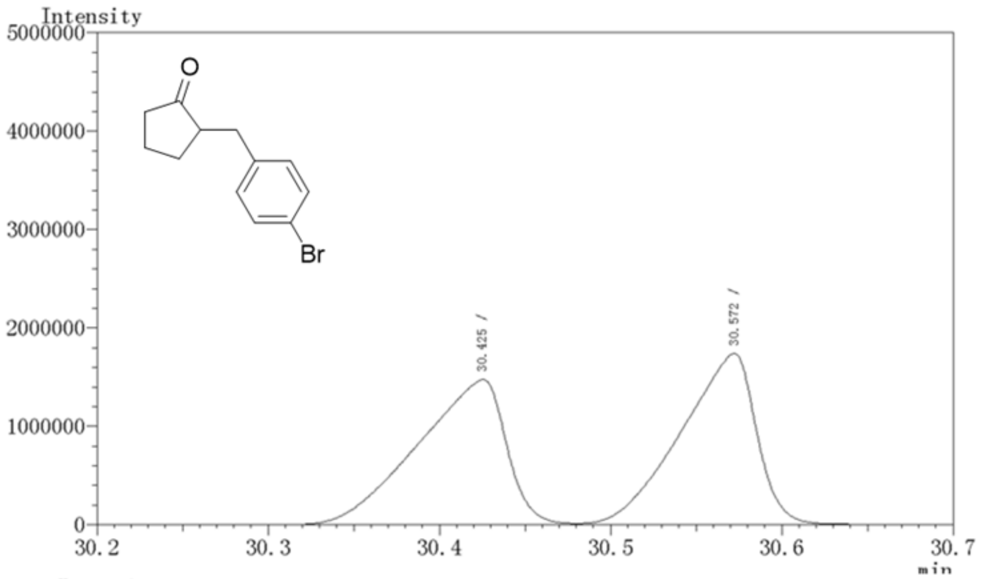
Intensity
5000000
4000000
3000000
2000000
1000000
0
O
Br
30.425
30.572
30.2
30.3
30.4
30.5
30.6
30.7
min

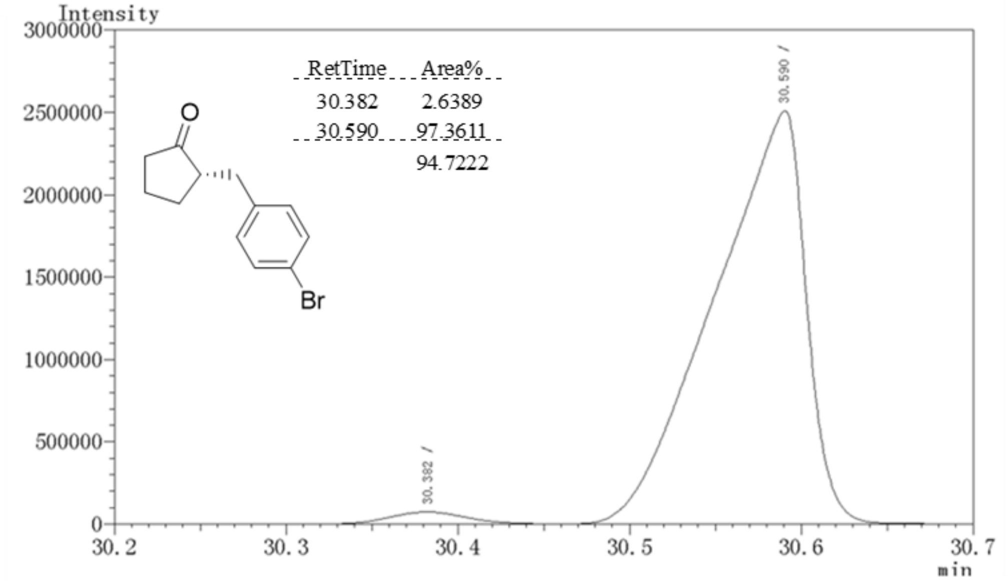
Intensity
3000000
2500000
2000000
1500000
1000000
500000
0
RetTime Area%
30.382 2.6389
30.590 97.3611
94.7222
O
Br
30.382
30.590
30.2
30.3
30.4
30.5
30.6
30.7
min

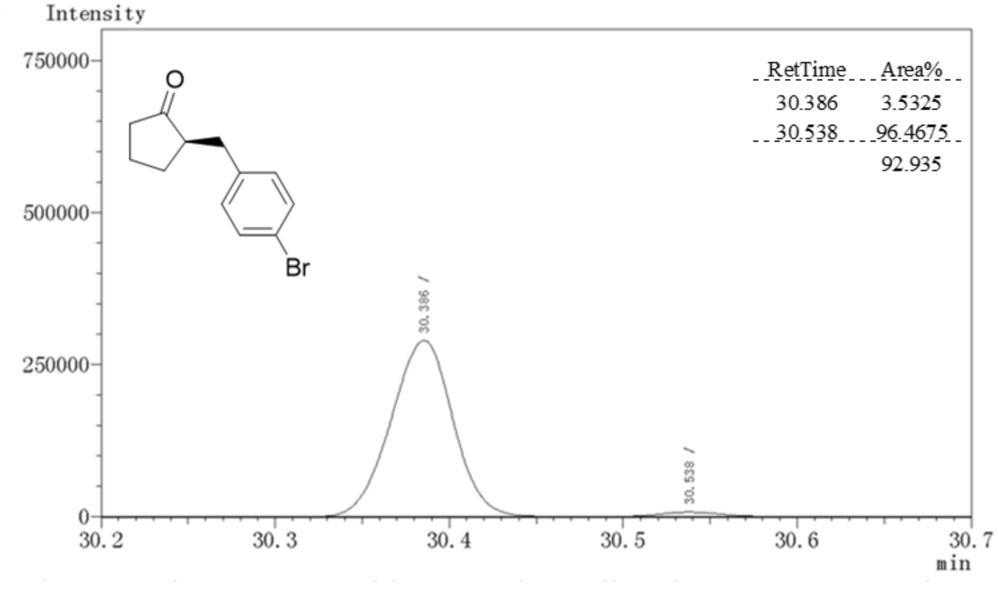
Intensity
750000
500000
250000
0
RetTime Area%
30.386 3.5325
30.538 96.4675
92.935
O
Br
30.386
30.538
30.2
30.3
30.4
30.5
30.6
30.7
min

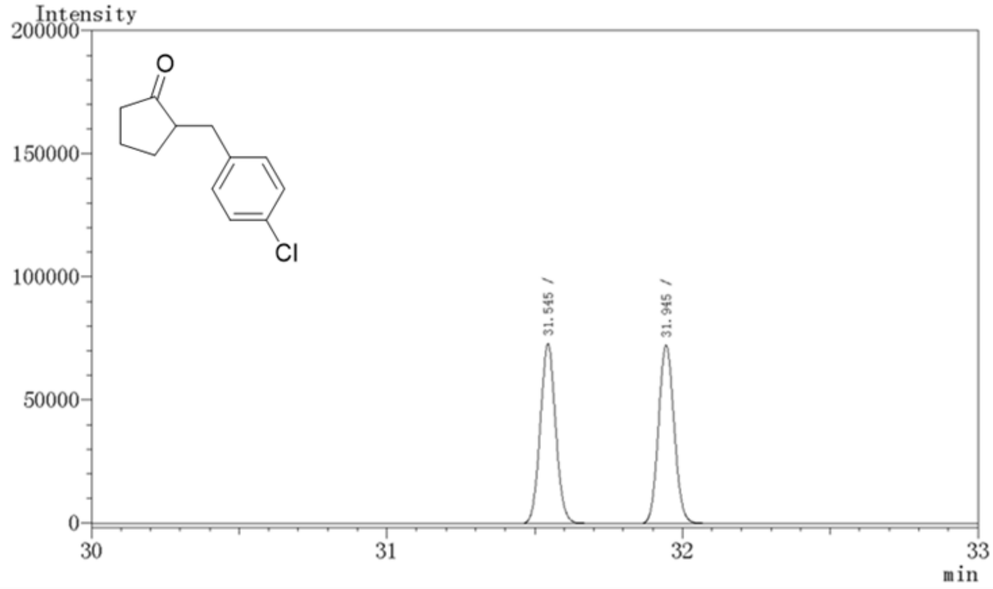
Intensity
200000
150000
100000
50000
0
O
Cl
31.545
31.945
30
31
32
33
min

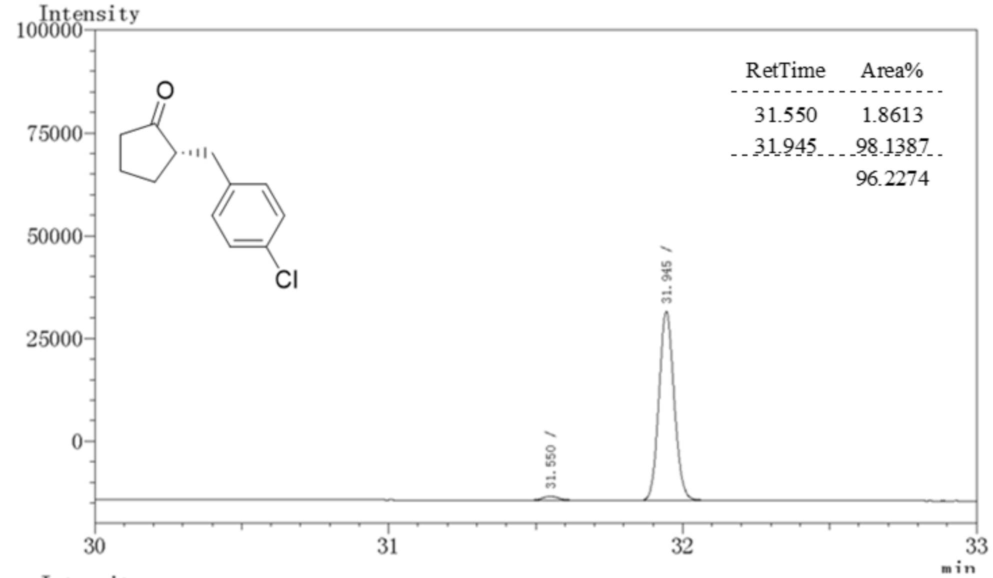
Intensity
100000
75000
50000
25000
0
O
Cl
RetTime Area%
31.550 1.8613
31.945 98.1387
96.2274
31.550
31.945
30
31
32
33
min

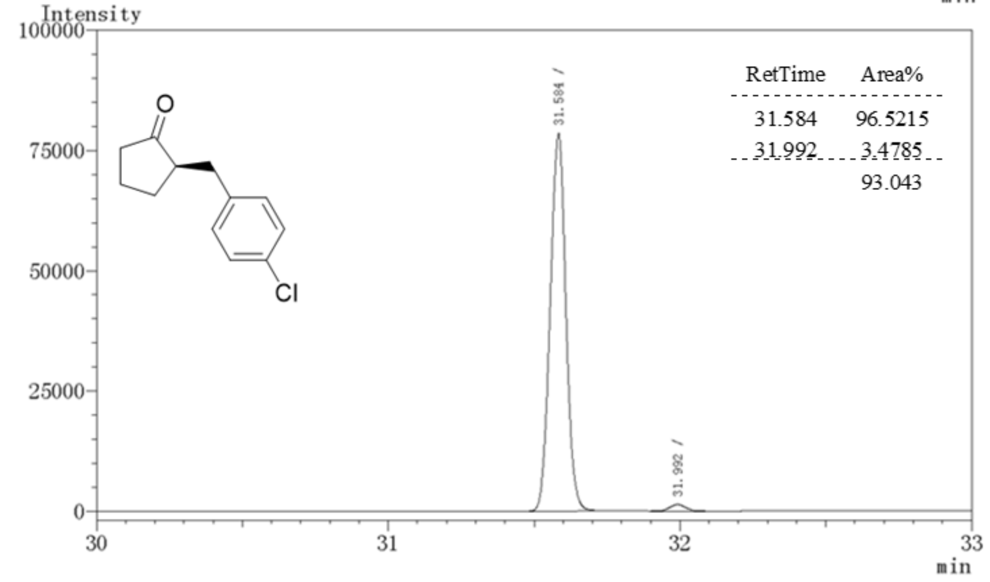
Intensity
100000
75000
50000
25000
0
O
Cl
RetTime Area%
31.584 96.5215
31.992 3.4785
93.043
31.584
31.992
30
31
32
33
min

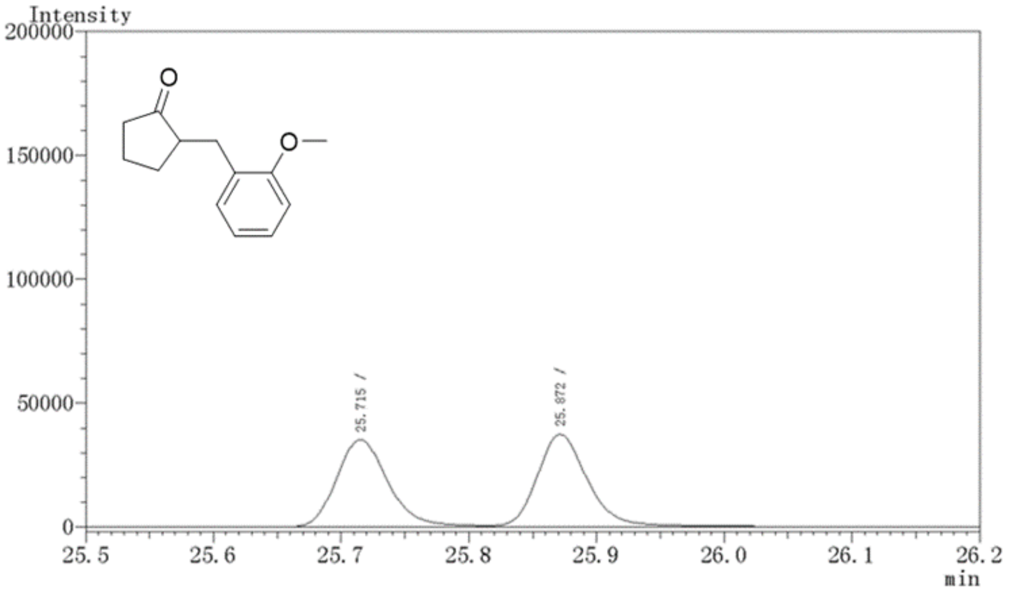

Intensity
200000
150000
100000
50000
0
25.715
25.872
25.5
25.6
25.7
25.8
25.9
26.0
26.1
26.2
min

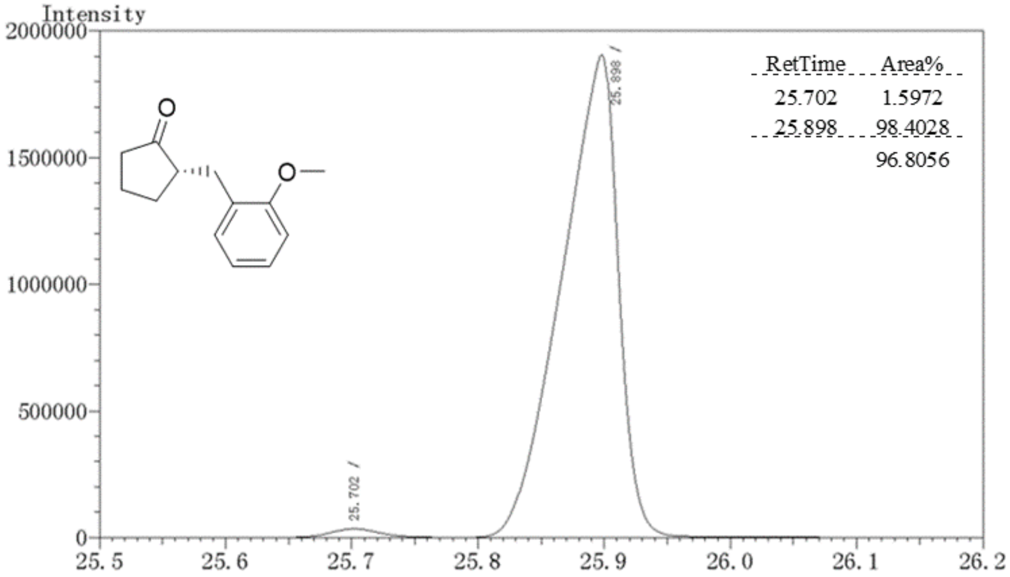

Intensity
2000000
1500000
1000000
500000
0
25.702
25.898
RetTime
Area%
25.702
1.5972
25.898
98.4028
96.8056
25.5
25.6
25.7
25.8
25.9
26.0
26.1
26.2

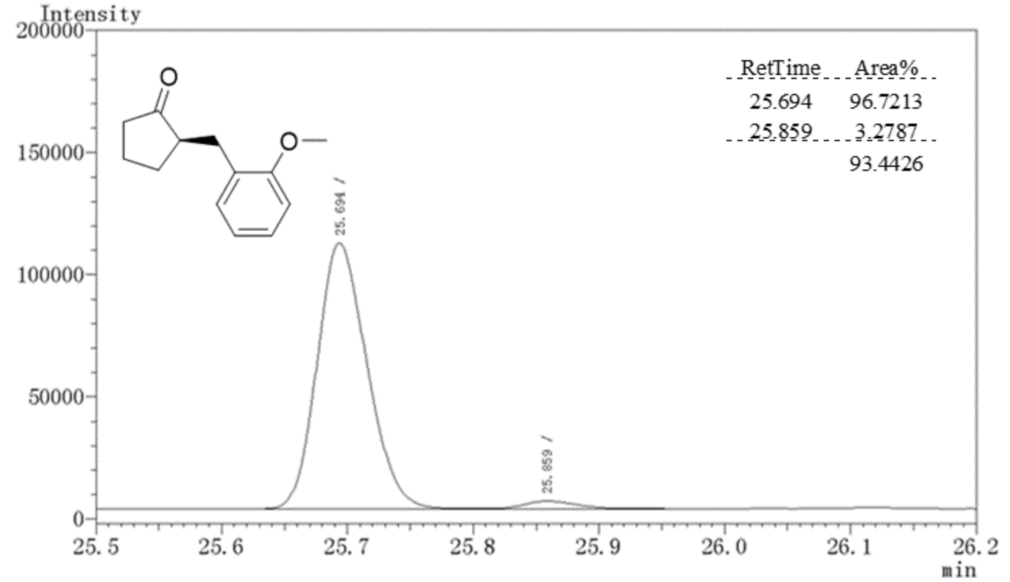

Intensity
200000
150000
100000
50000
0
25.694
25.859
RetTime
Area%
25.694
96.7213
25.859
3.2787
93.4426
25.5
25.6
25.7
25.8
25.9
26.0
26.1
26.2
min

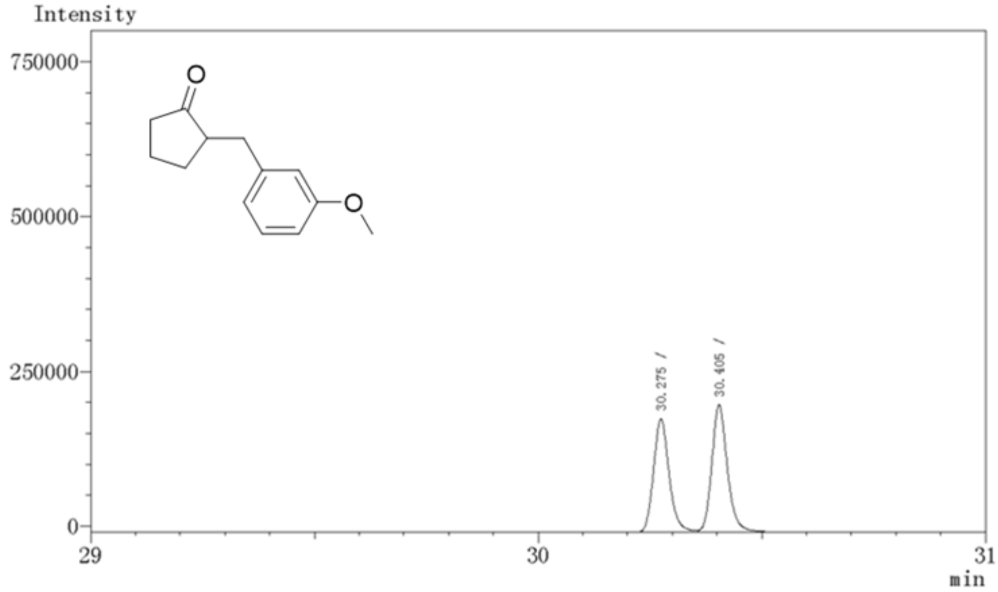
Intensity
750000
500000
250000
0
30.275
30.405
29
30
31
min

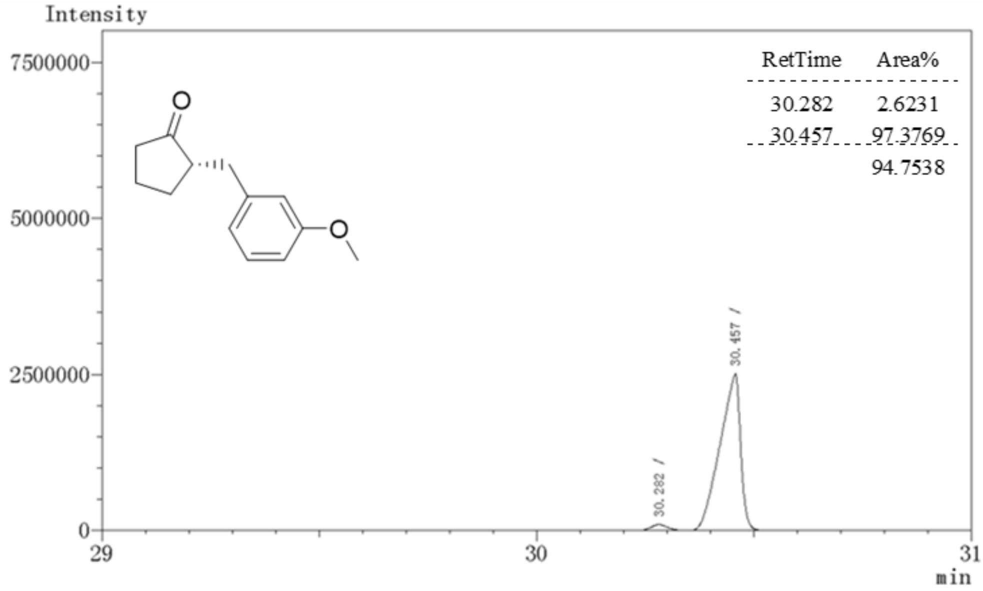
Intensity
7500000
5000000
2500000
0
RetTime Area%
30.282 2.6231
30.457 97.3769
94.7538
30.282
30.457
29
30
31
min

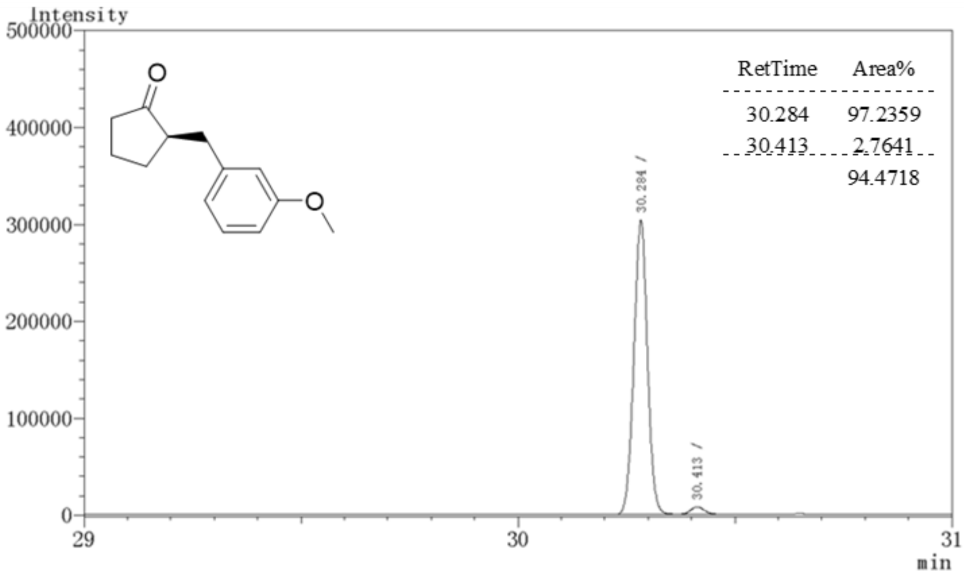
Intensity
500000
400000
300000
200000
100000
0
RetTime Area%
30.284 97.2359
30.413 2.7641
94.4718
30.284
30.413
29
30
31
min

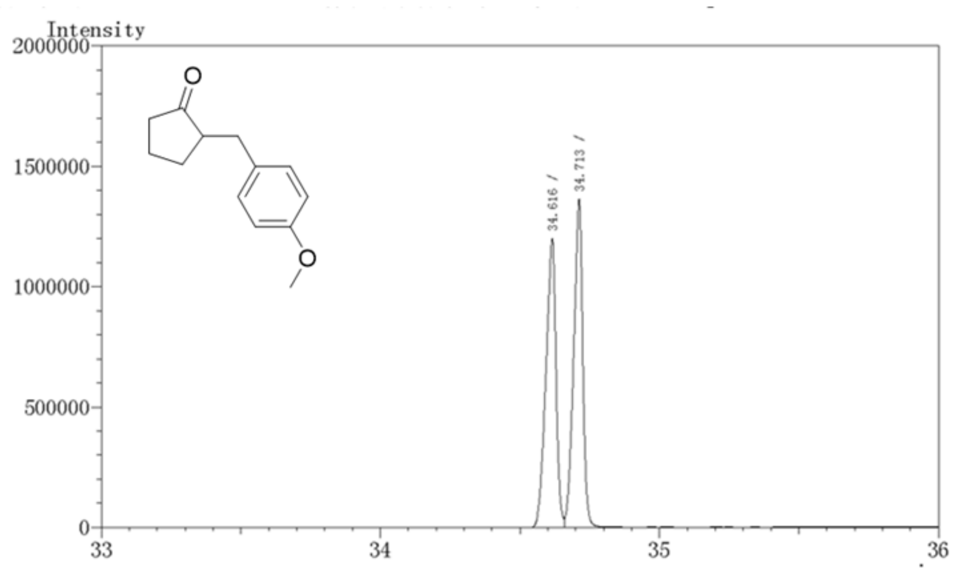
Intensity
2000000
1500000
1000000
500000
0
34.616
34.713
33
34
35
36

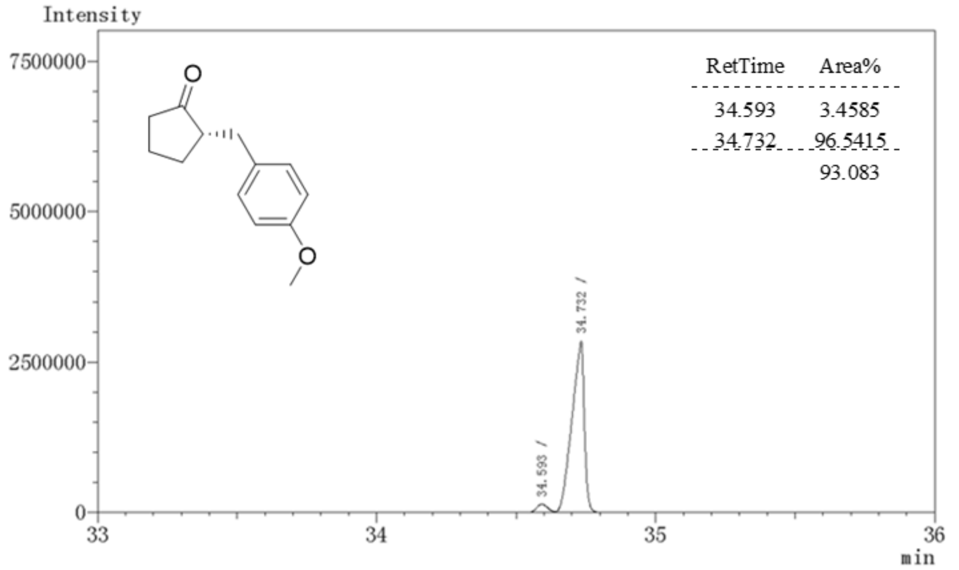
Intensity
7500000
5000000
2500000
0
RetTime Area%
34.593 3.4585
34.732 96.5415
93.083
34.593
34.732
33
34
35
36
min

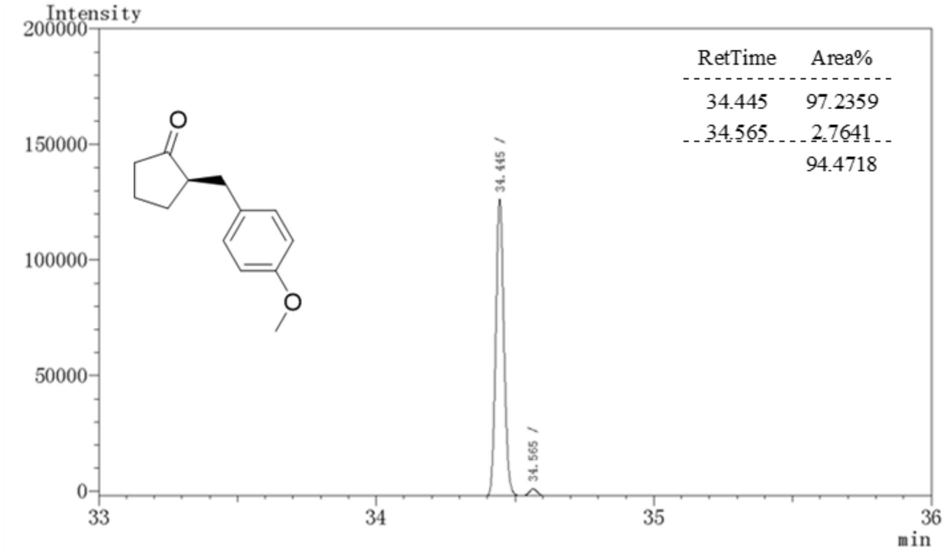
Intensity
200000
150000
100000
50000
0
RetTime Area%
34.445 97.2359
34.565 2.7641
94.4718
34.445
34.565
33
34
35
36
min

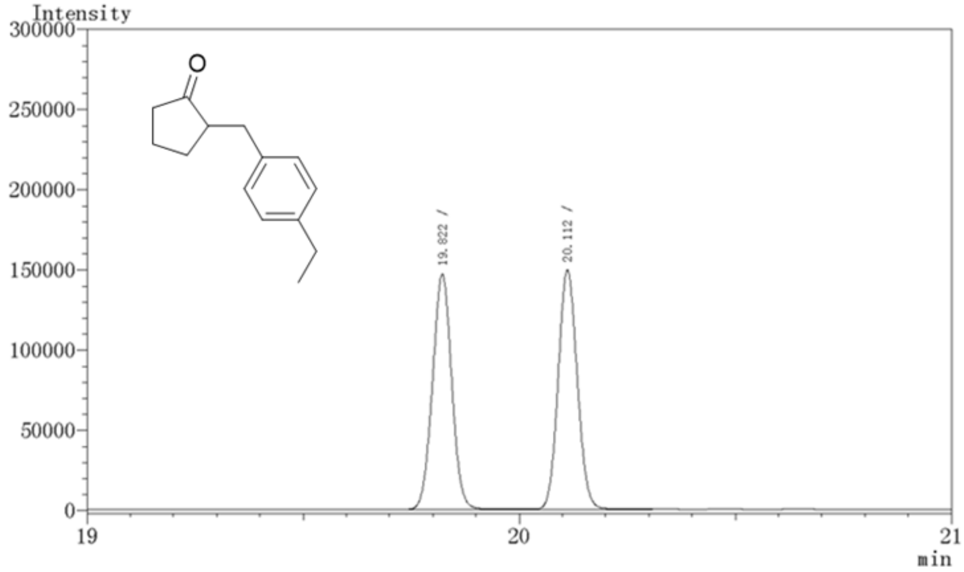

Intensity
300000
250000
200000
150000
100000
50000
0
19.822
20.112
19
20
21
min

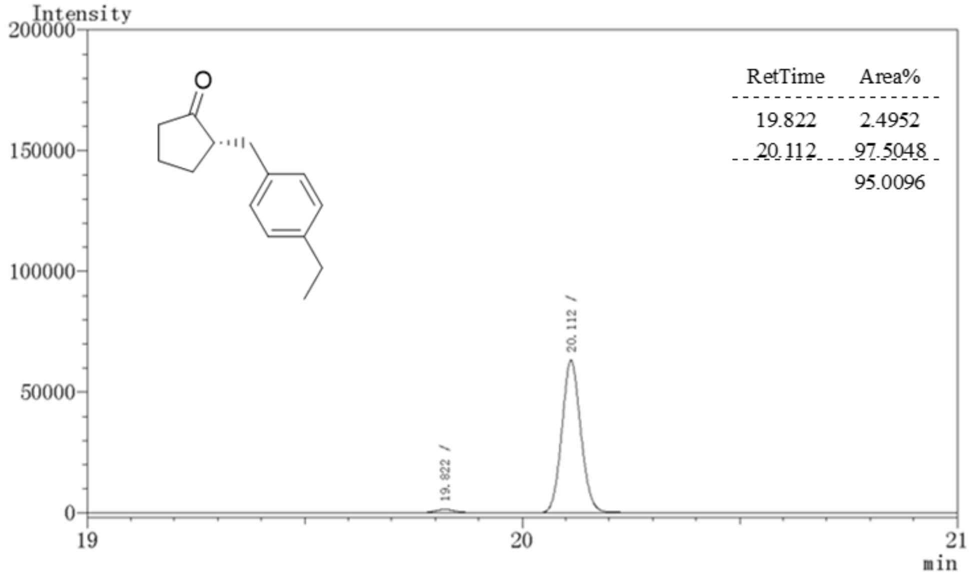

Intensity
200000
150000
100000
50000
0
RetTime Area%
19.822 2.4952
20.112 97.5048
95.0096
19.822
20.112
19
20
21
min

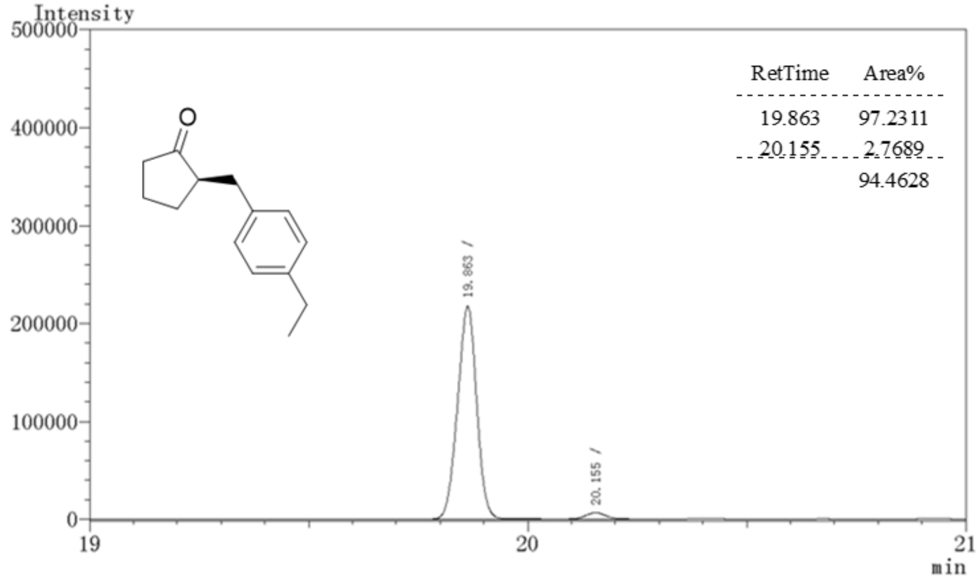

Intensity
500000
400000
300000
200000
100000
0
RetTime Area%
19.863 97.2311
20.155 2.7689
94.4628
19.863
20.155
19
20
21
min

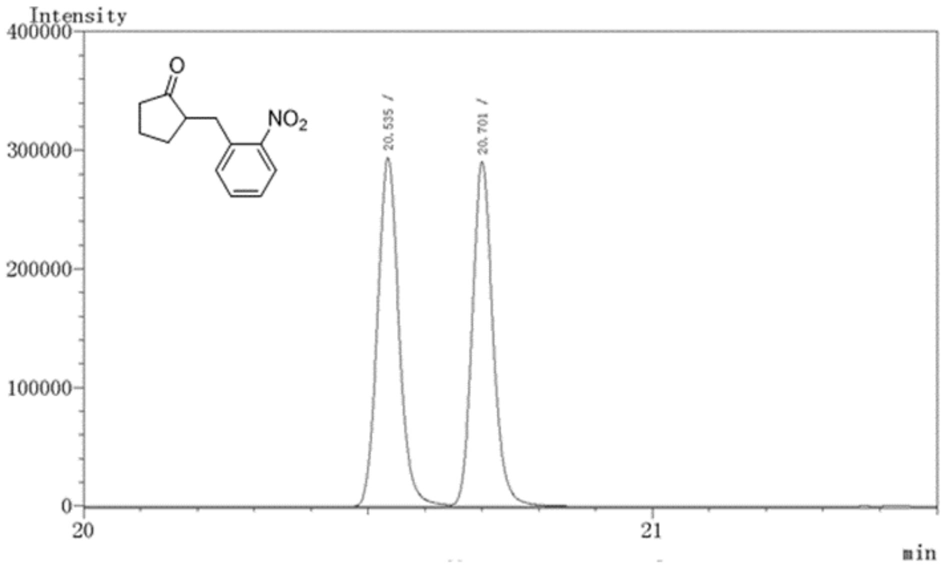
Intensity
400000
300000
200000
100000
0
20
21
min
NO2
O
20.535
20.701

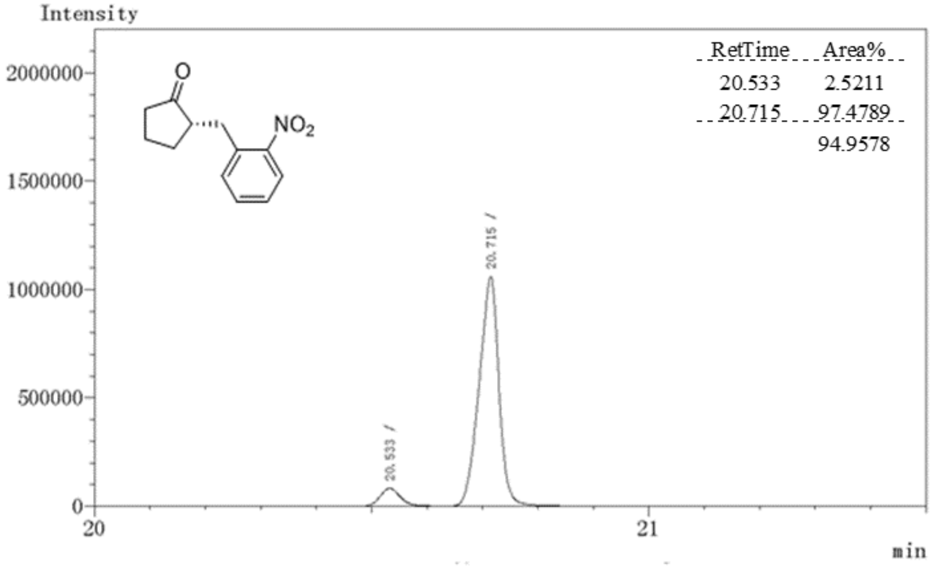
Intensity
2000000
1500000
1000000
500000
0
20
21
min
O
NO2
RetTime Area%
20.533 2.5211
20.715 97.4789
94.9578
20.715
20.533

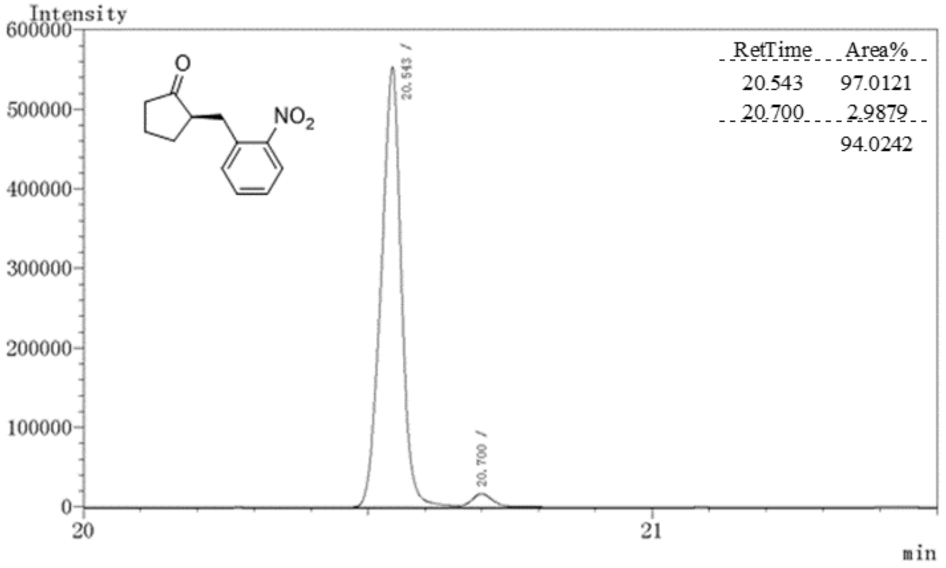
Intensity
600000
500000
400000
300000
200000
100000
0
20
21
min
O
NO2
RetTime Area%
20.543 97.0121
20.700 2.9879
94.0242
20.543
20.700

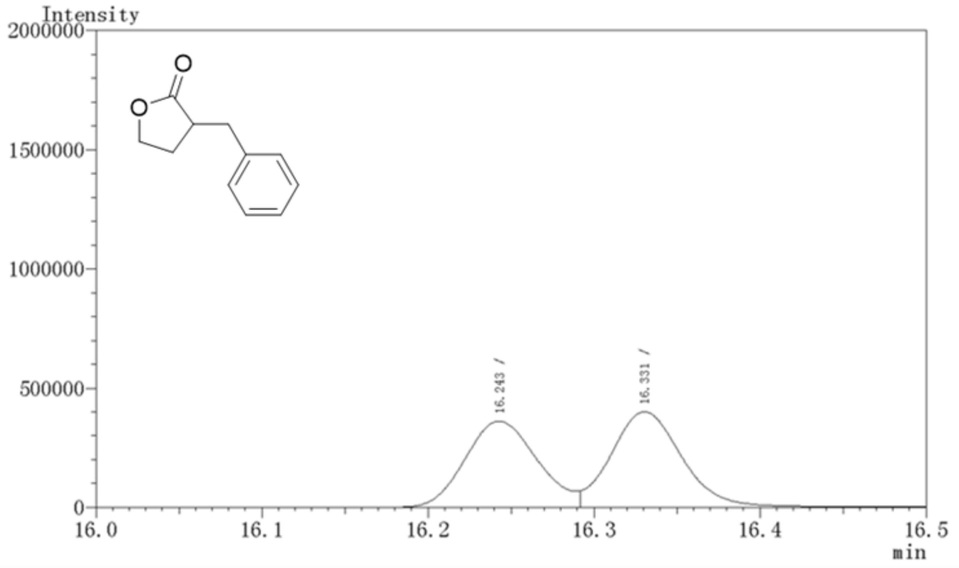
Intensity
2000000
1500000
1000000
500000
0
16.243
16.331
16.0
16.1
16.2
16.3
16.4
16.5
min

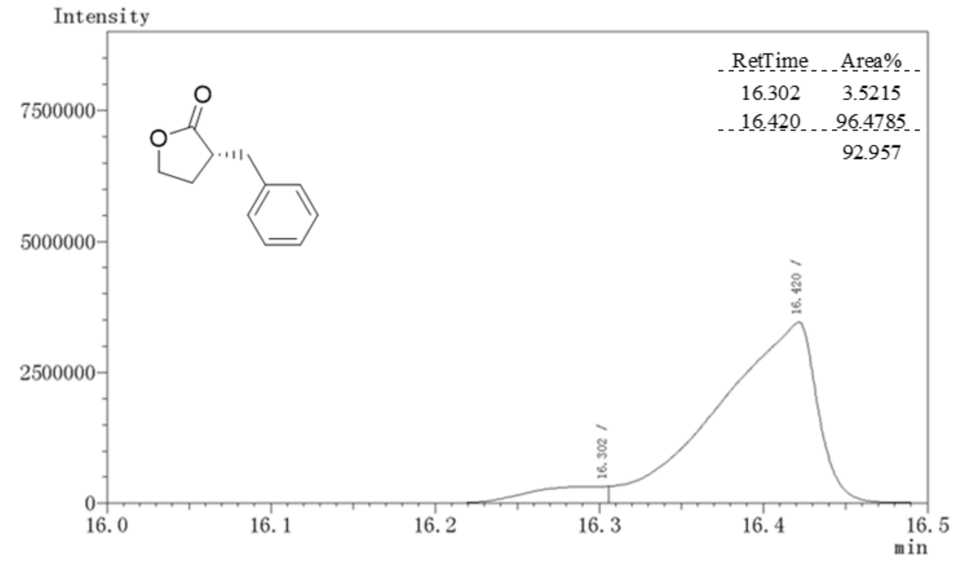
Intensity
RetTime Area%
16.302 3.5215
16.420 96.4785
92.957
7500000
5000000
2500000
0
16.302
16.420
16.0
16.1
16.2
16.3
16.4
16.5
min

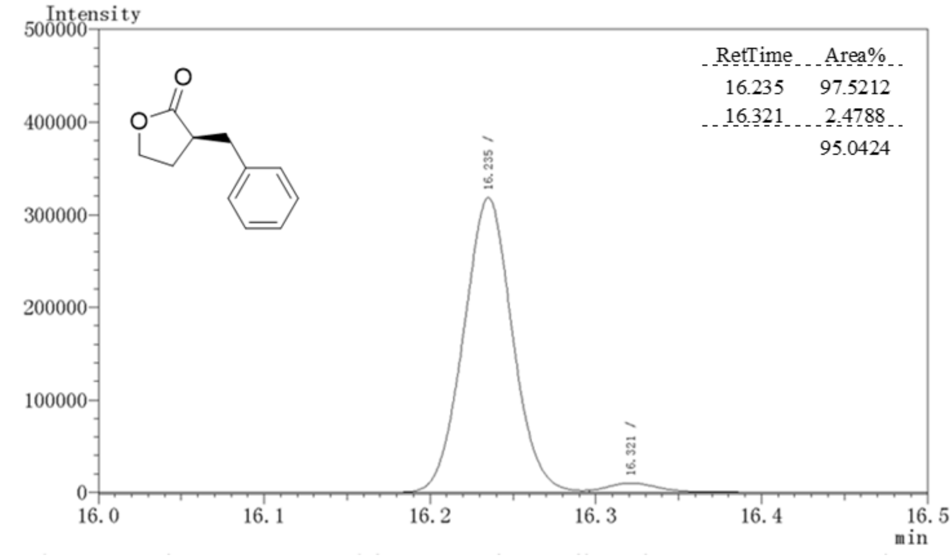
Intensity
RetTime Area%
16.235 97.5212
16.321 2.4788
95.0424
500000
400000
300000
200000
100000
0
16.235
16.321
16.0
16.1
16.2
16.3
16.4
16.5
min

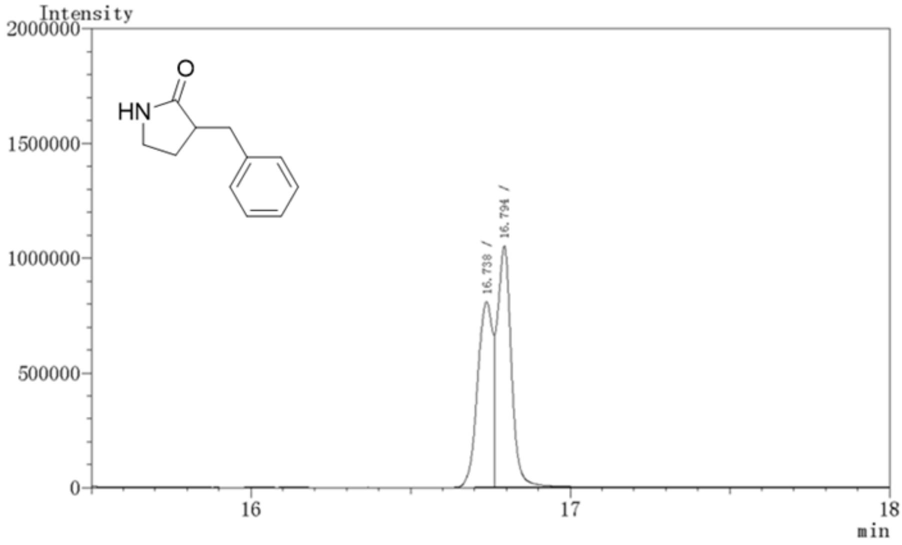
Intensity
2000000
1500000
1000000
500000
0
HN
O
16.738
16.794
16
17
18
min

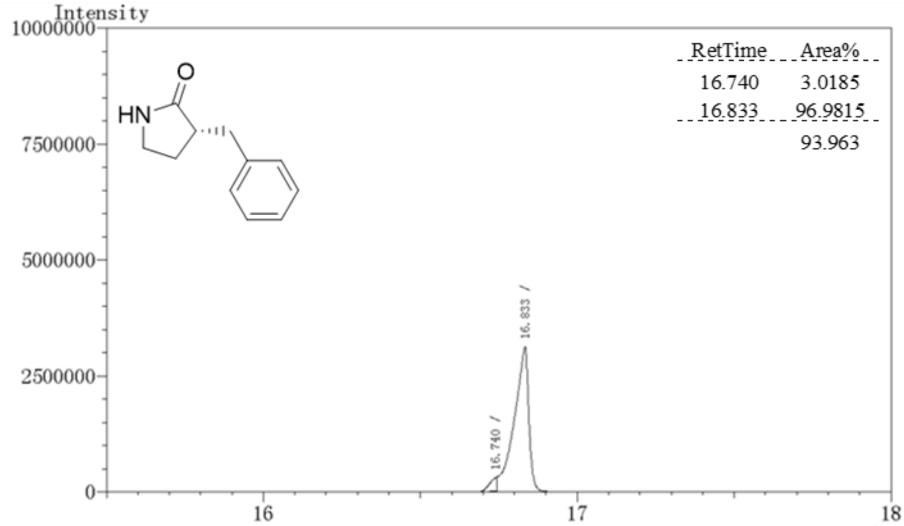
Intensity
10000000
7500000
5000000
2500000
0
HN
O
RetTime Area%
16.740 3.0185
16.833 96.9815
93.963
16.740
16.833
16
17
18

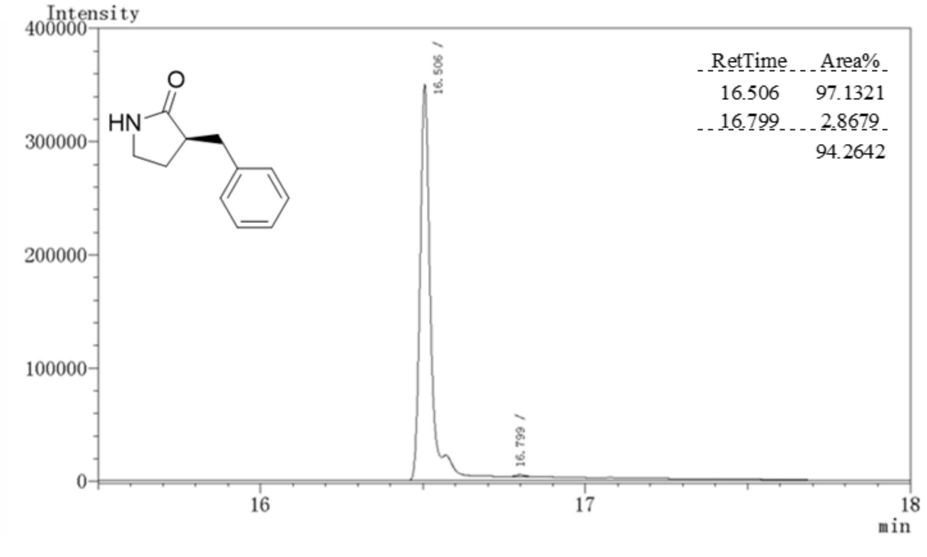
Intensity
400000
300000
200000
100000
0
HN
O
RetTime Area%
16.506 97.1321
16.799 2.8679
94.2642
16.506
16.799
16
17
18
min

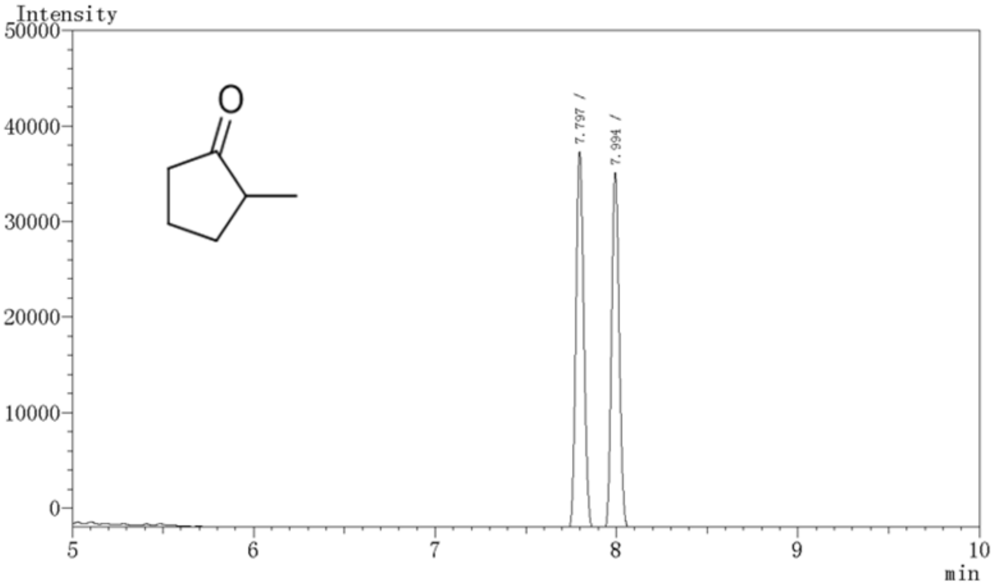

Intensity
50000
40000
30000
20000
10000
0
5
6
7
8
9
10
min
7.797
7.994

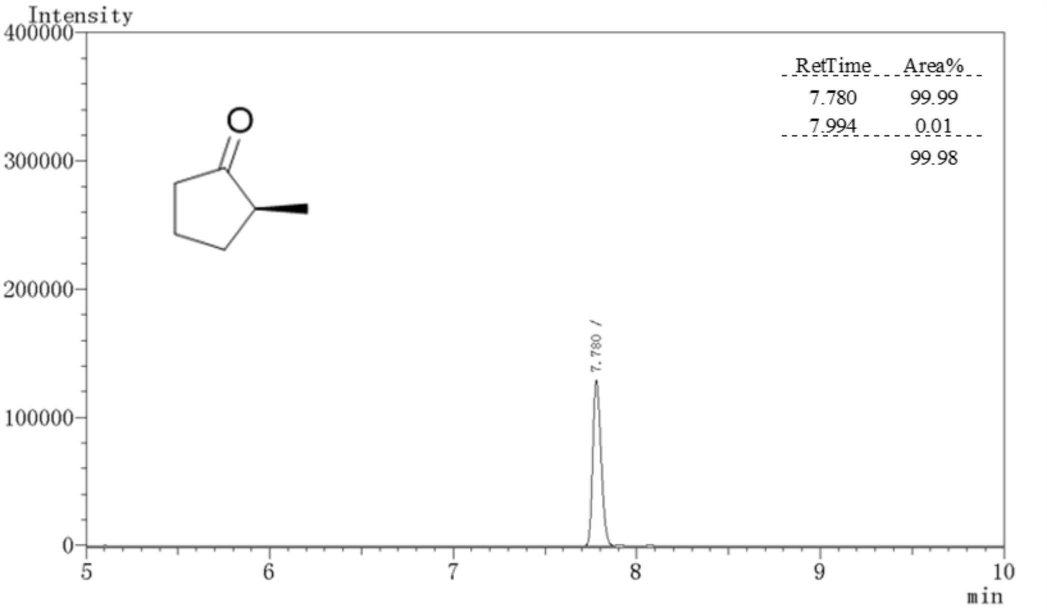

Intensity
400000
300000
200000
100000
0
5
6
7
8
9
10
min
7.780
RetTime Area%
7.780 99.99
7.994 0.01
99.98

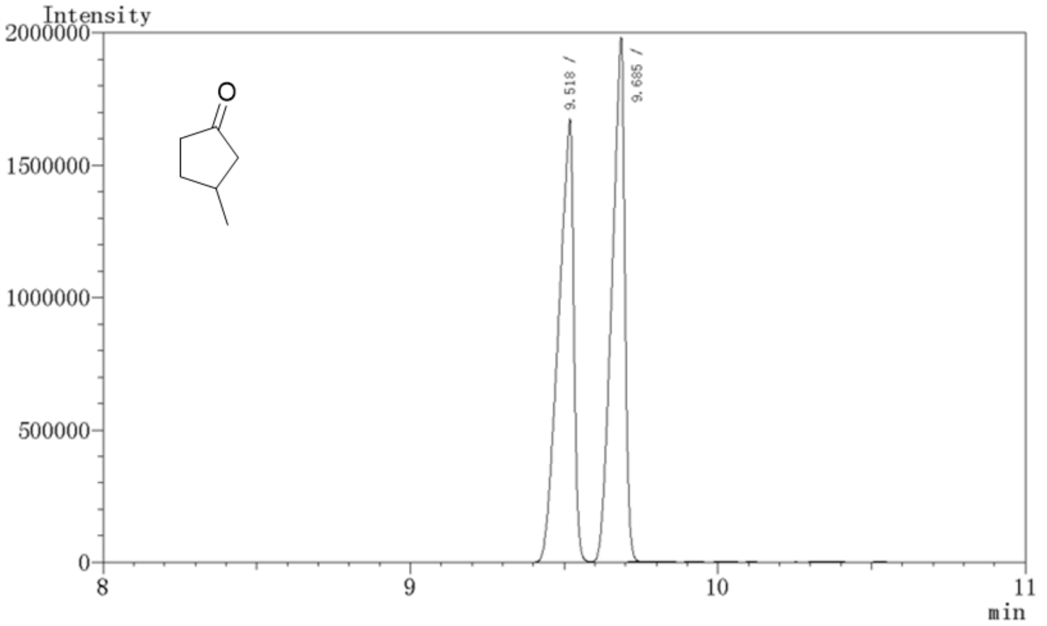
Intensity
2000000
1500000
1000000
500000
0
9.518
9.685
8
9
10
11
min

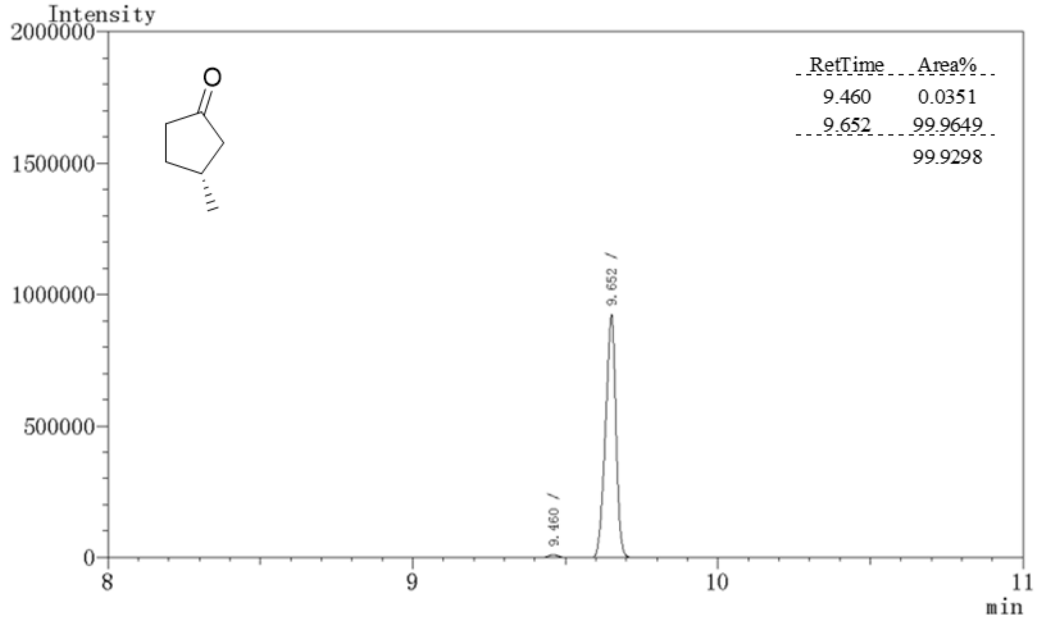
Intensity
2000000
1500000
1000000
500000
0
RetTime Area%
9.460 0.0351
9.652 99.9649
99.9298
9.652
9.460
8
9
10
11
min

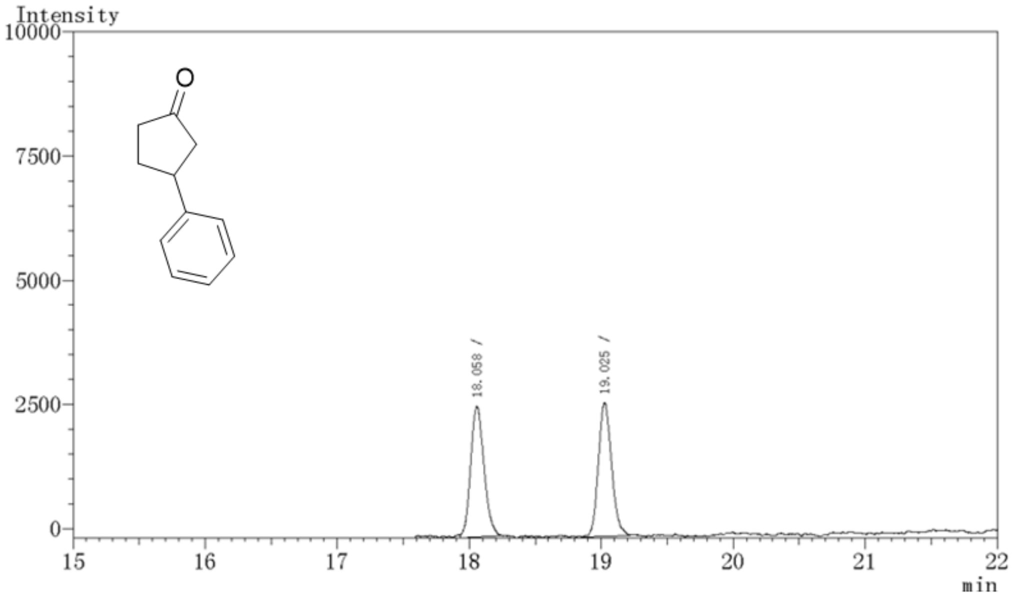
Intensity
18.058
19.025
min

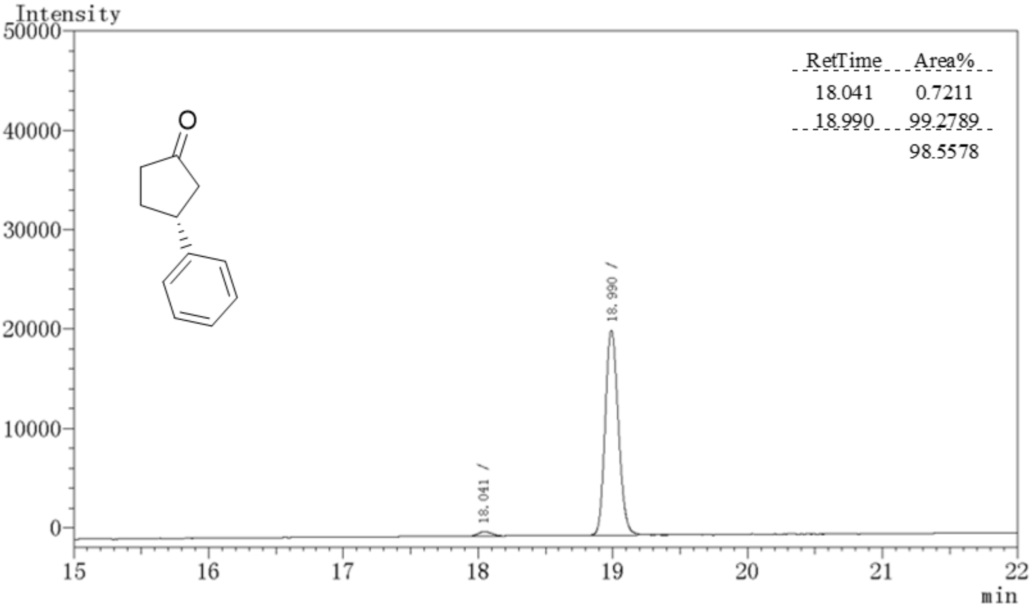
Intensity
RetTime Area%
18.041 0.7211
18.990 99.2789
98.5578
18.041
18.990
min

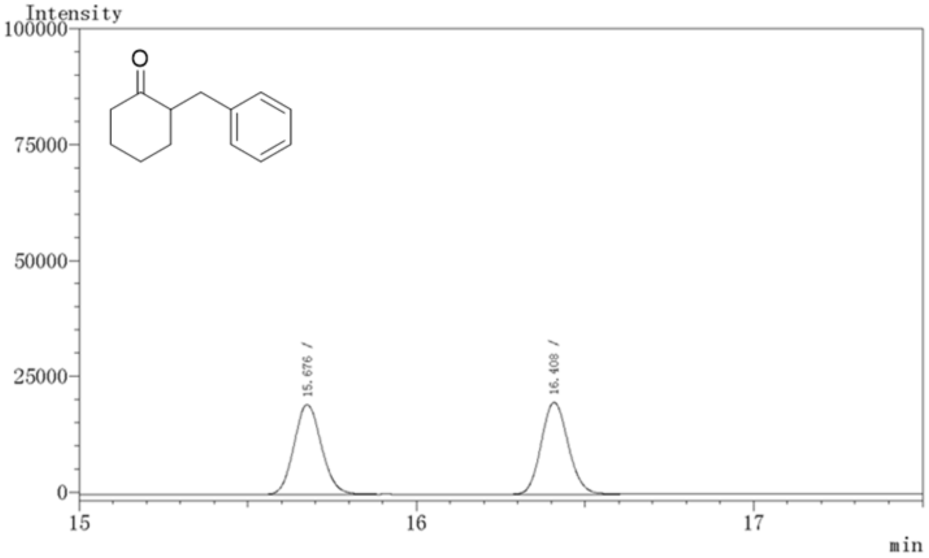
Intensity
100000
75000
50000
25000
0
15.676
16.408
15
16
17
min
O

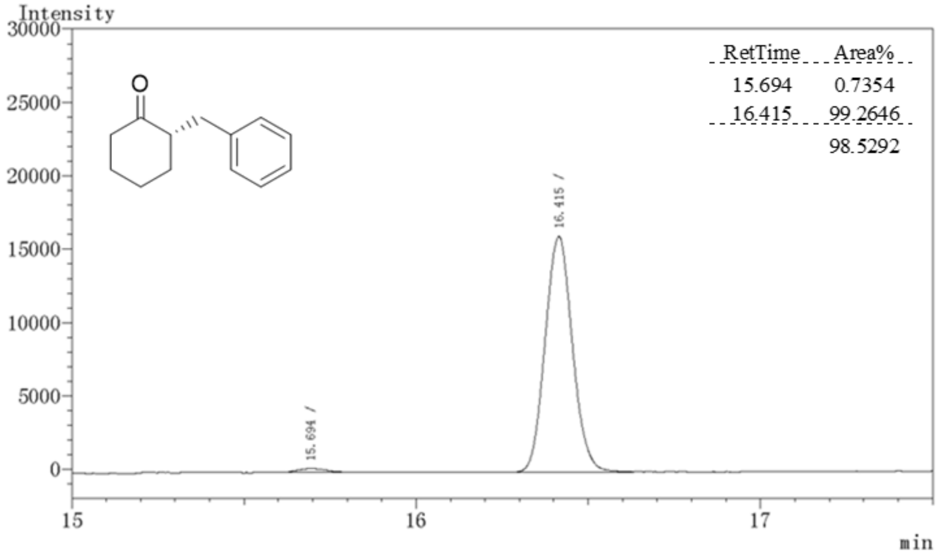
Intensity
30000
25000
20000
15000
10000
5000
0
RetTime Area%
15.694 0.7354
16.415 99.2646
98.5292
15.694
16.415
15
16
17
min
O

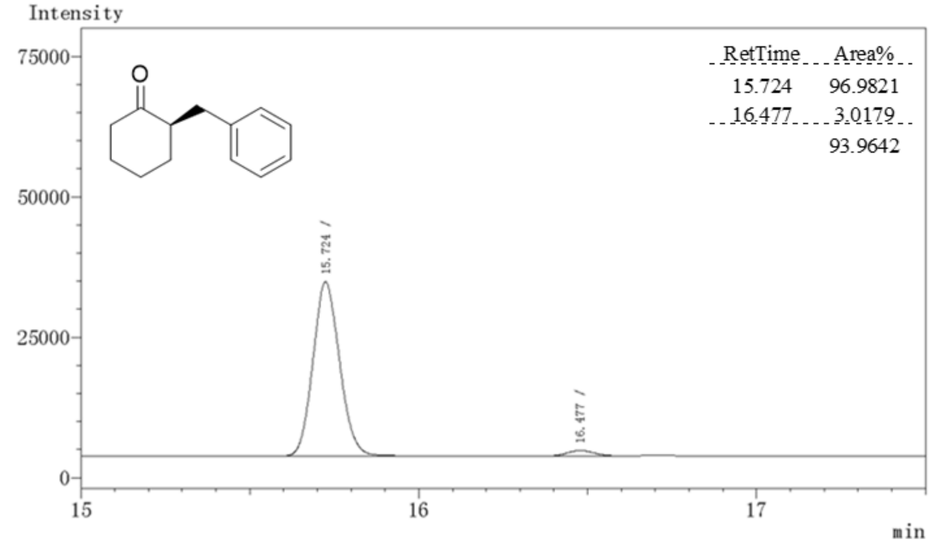
Intensity
75000
50000
25000
0
RetTime Area%
15.724 96.9821
16.477 3.0179
93.9642
15.724
16.477
15
16
17
min
O

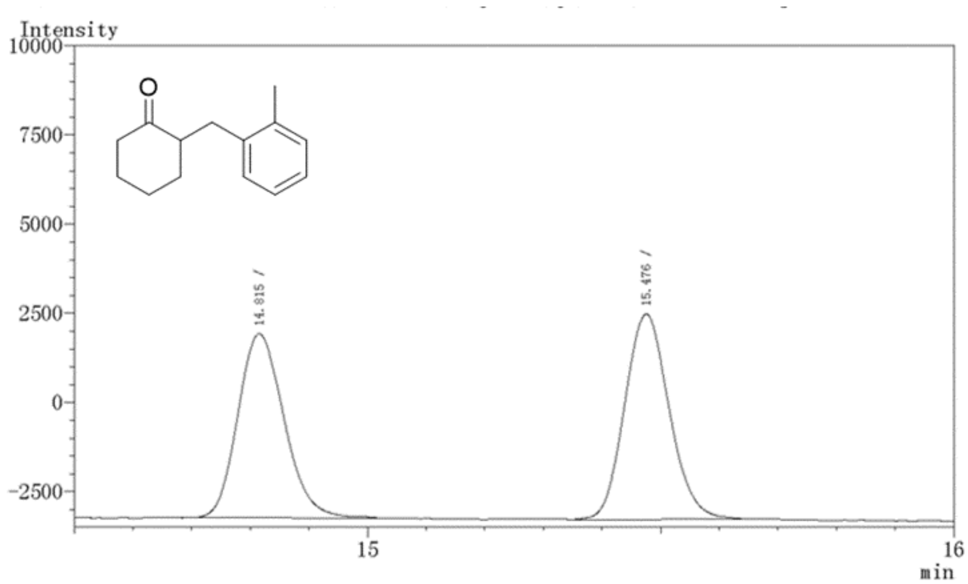
Intensity
10000
7500
5000
2500
0
-2500
14.815
15.476
15
16
min

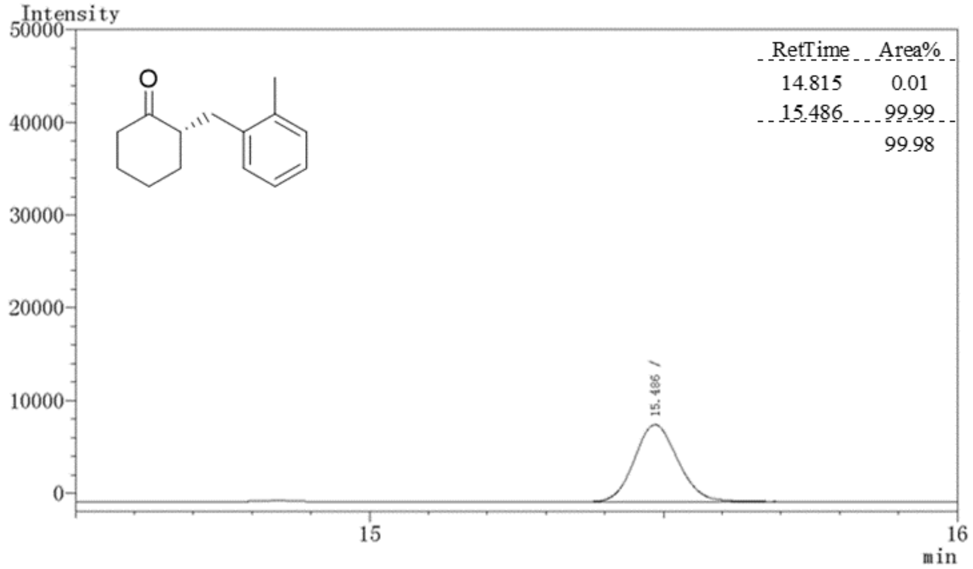
Intensity
50000
40000
30000
20000
10000
0
RetTime Area%
14.815 0.01
15.486 99.99
99.98
15.486
15
16
min

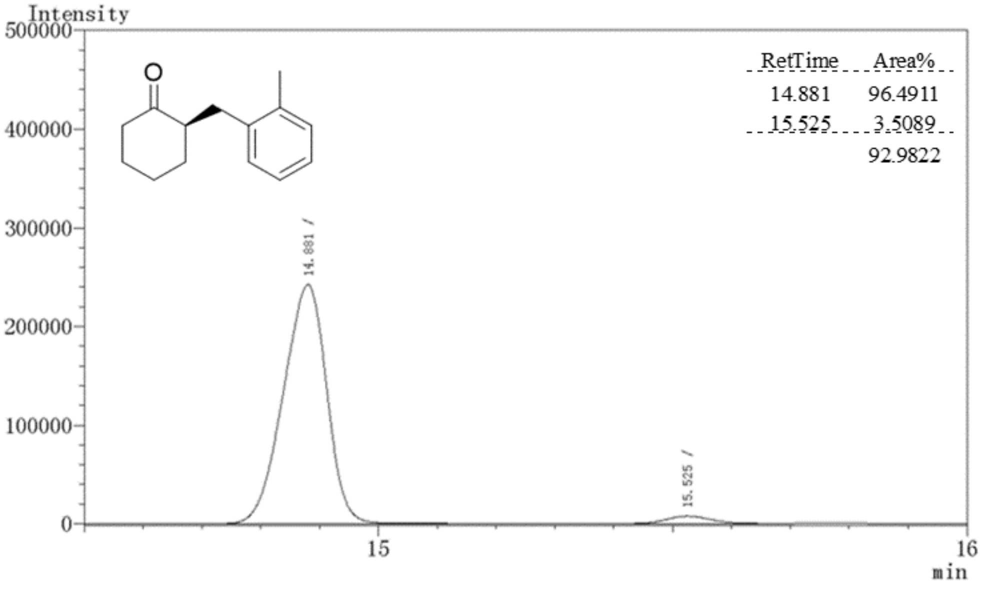
Intensity
500000
400000
300000
200000
100000
0
RetTime Area%
14.881 96.4911
15.525 3.5089
92.9822
14.881
15.525
15
16
min

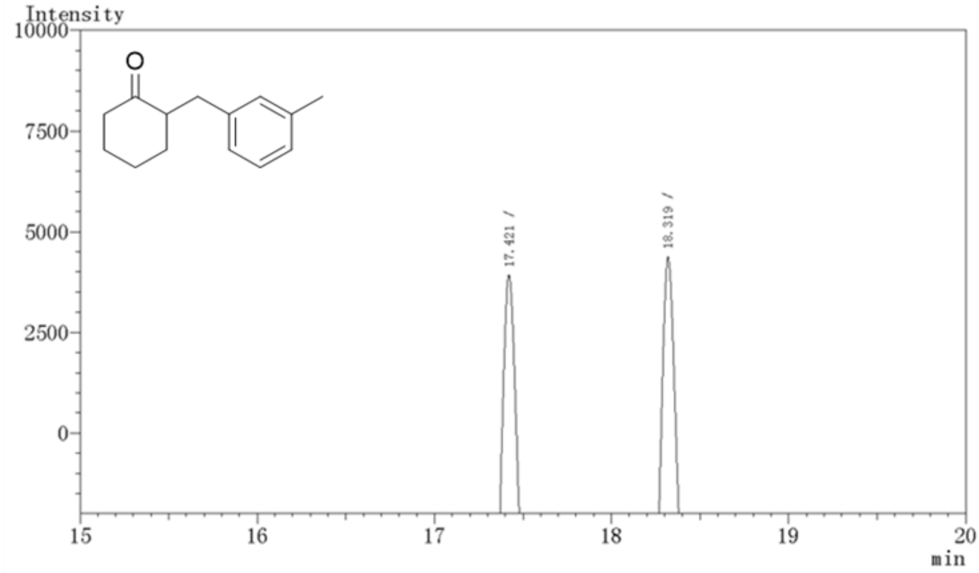
Intensity
10000
7500
5000
2500
0
17.421
18.319
15
16
17
18
19
20
min

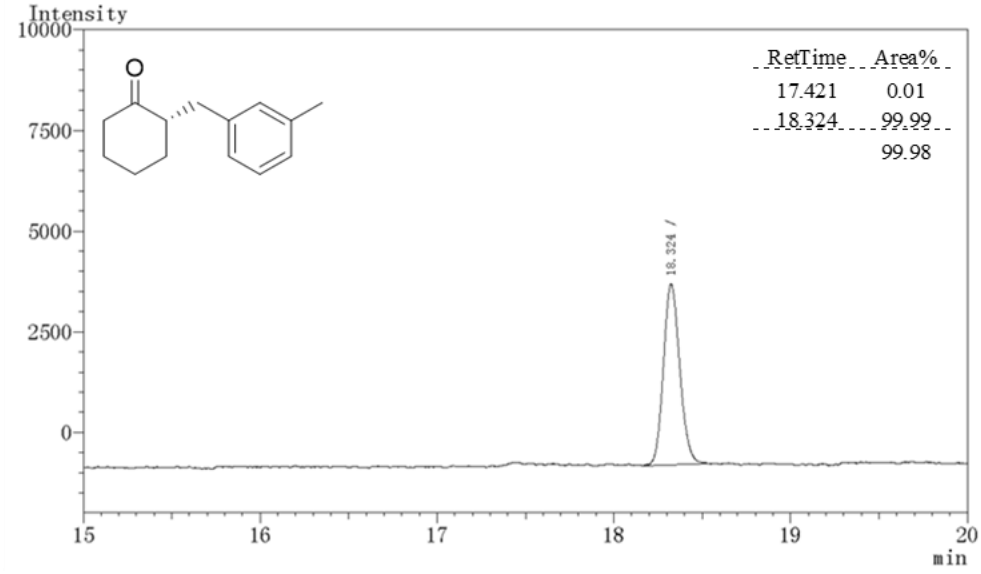
Intensity
10000
7500
5000
2500
0
RetTime Area%
17.421 0.01
18.324 99.99
99.98
18.324
15
16
17
18
19
20
min

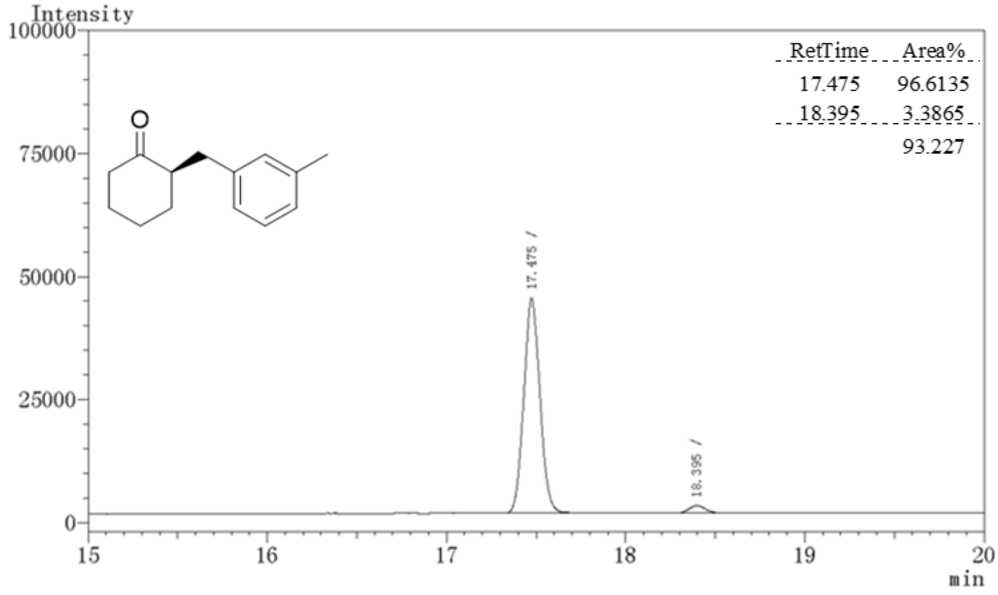
Intensity
100000
75000
50000
25000
0
RetTime Area%
17.475 96.6135
18.395 3.3865
93.227
17.475
18.395
15
16
17
18
19
20
min

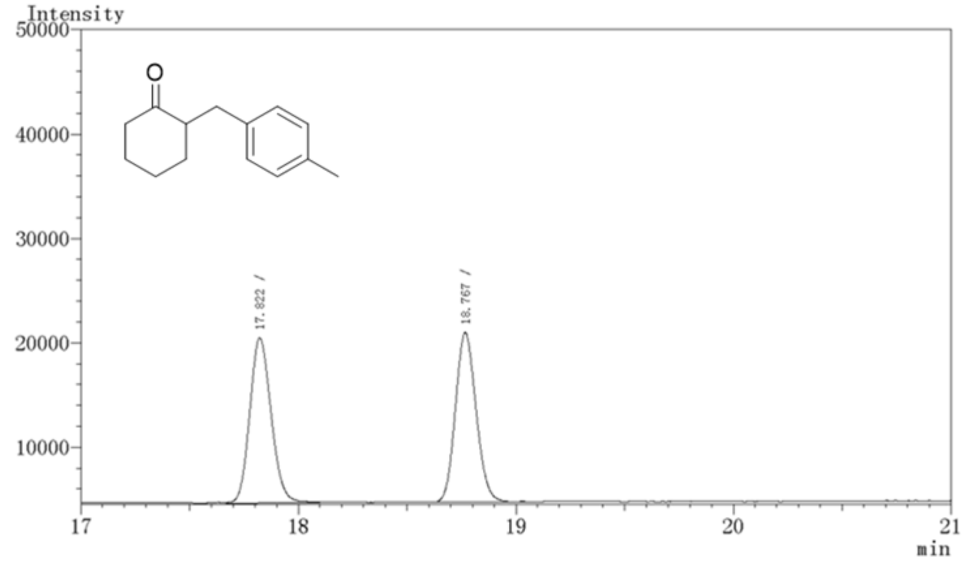
Intensity
50000
40000
30000
20000
10000
17.822
18.767
17
18
19
20
21
min

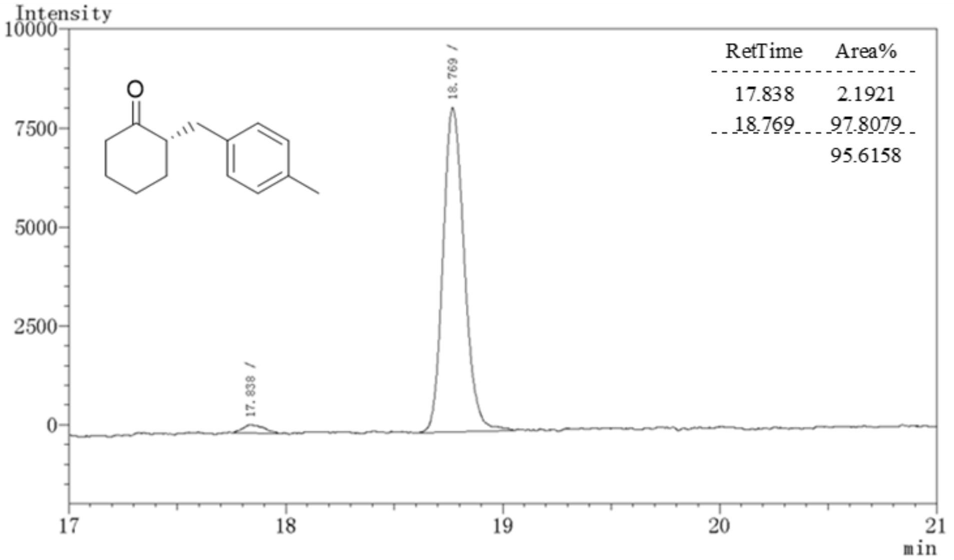
Intensity
10000
7500
5000
2500
0
RetTime Area%
17.838 2.1921
18.769 97.8079
95.6158
18.769
17.838
17
18
19
20
21
min

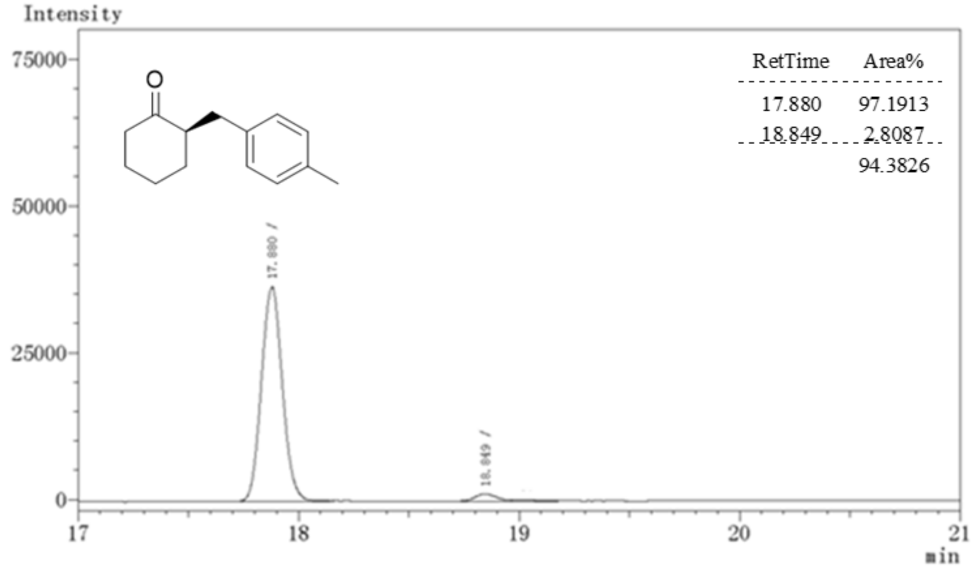
Intensity
75000
50000
25000
0
RetTime Area%
17.880 97.1913
18.849 2.8087
94.3826
17.880
18.849
17
18
19
20
21
min

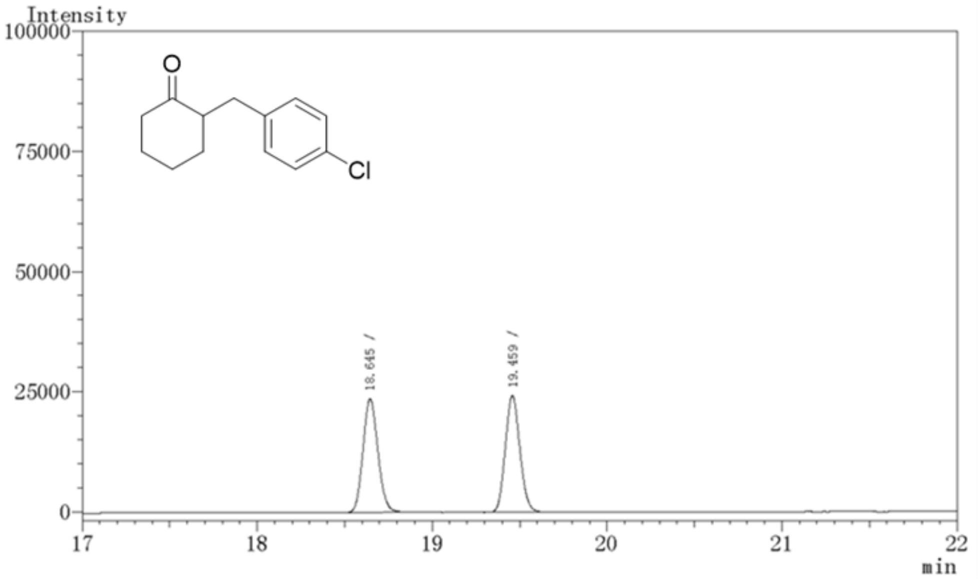
Intensity
100000
75000
50000
25000
0
18.645
19.459
O
Cl
17
18
19
20
21
22
min

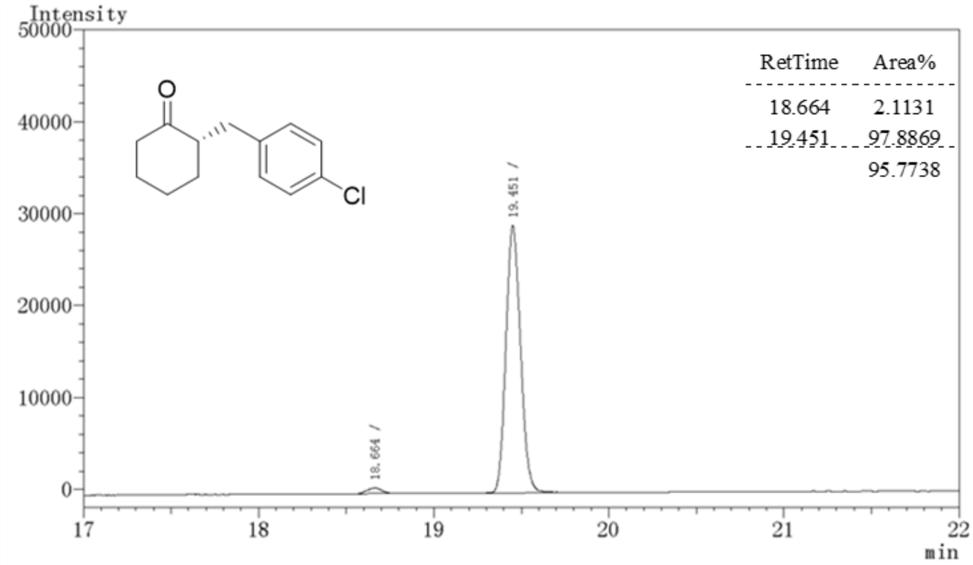
Intensity
50000
40000
30000
20000
10000
0
RetTime Area%
18.664 2.1131
19.451 97.8869
95.7738
19.451
18.664
O
Cl
17
18
19
20
21
22
min

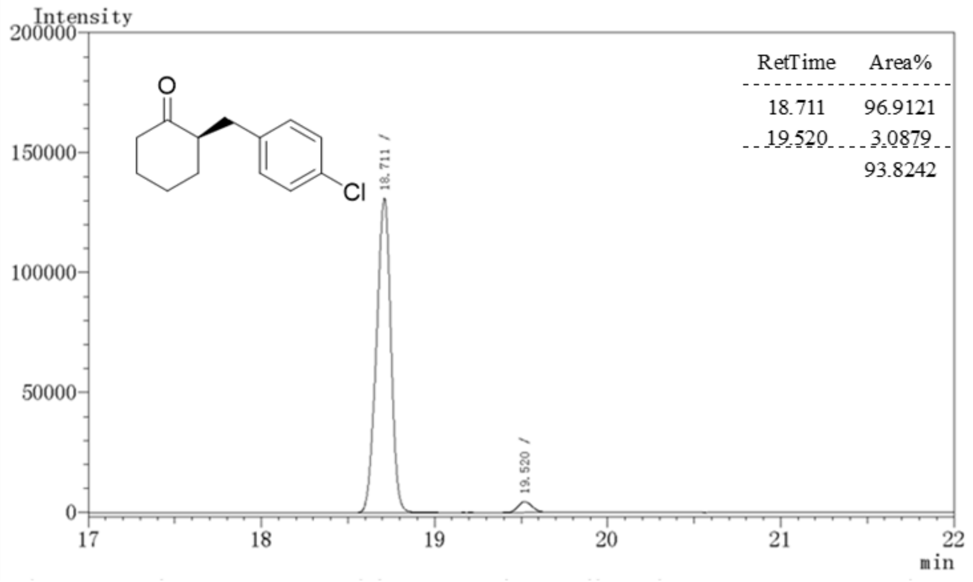
Intensity
200000
150000
100000
50000
0
RetTime Area%
18.711 96.9121
19.520 3.0879
93.8242
18.711
19.520
O
Cl
17
18
19
20
21
22
min

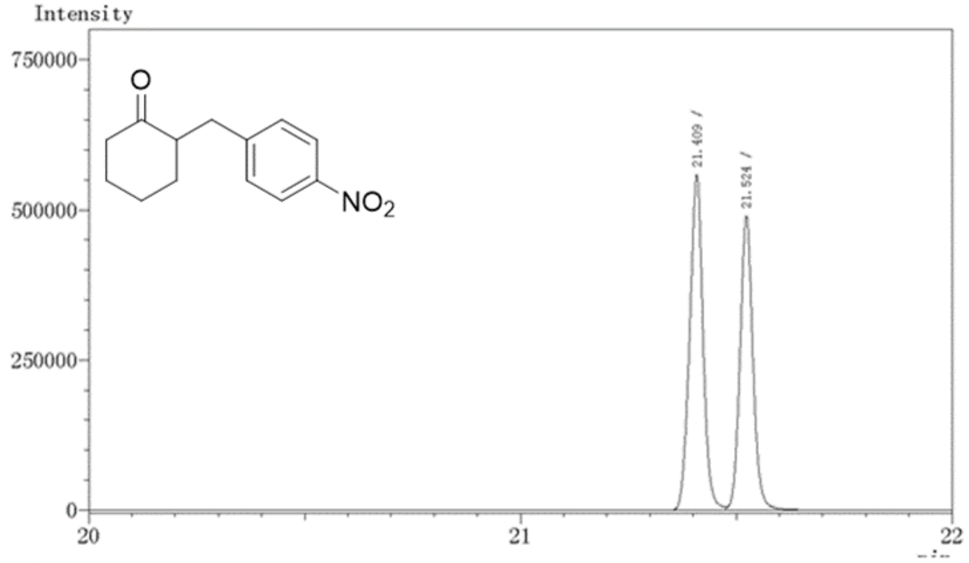
Intensity
750000
500000
250000
0
NO2
21.409
21.524
20
21
22

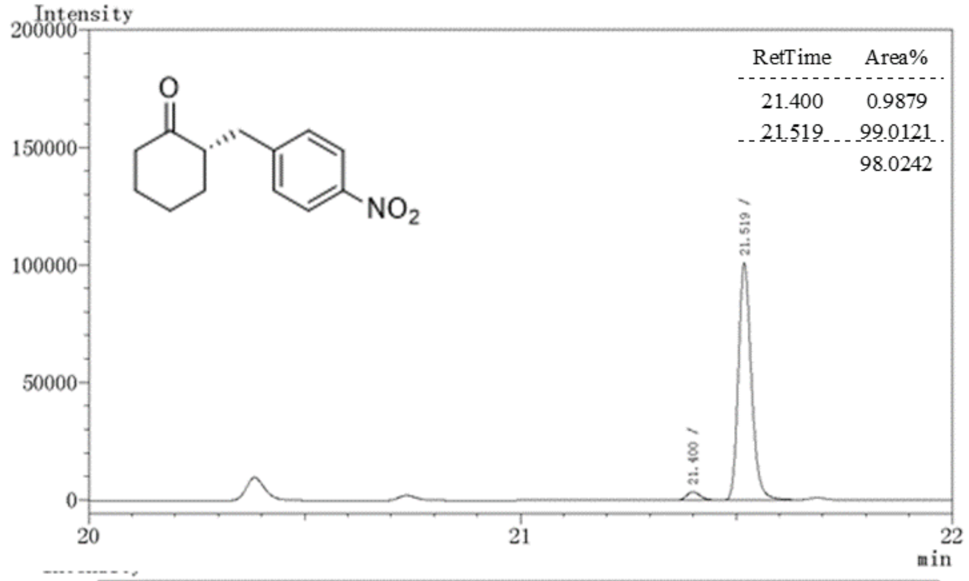
Intensity
200000
150000
100000
50000
0
NO2
RetTime Area%
21.400 0.9879
21.519 99.0121
98.0242
21.400
21.519
20
21
22
min

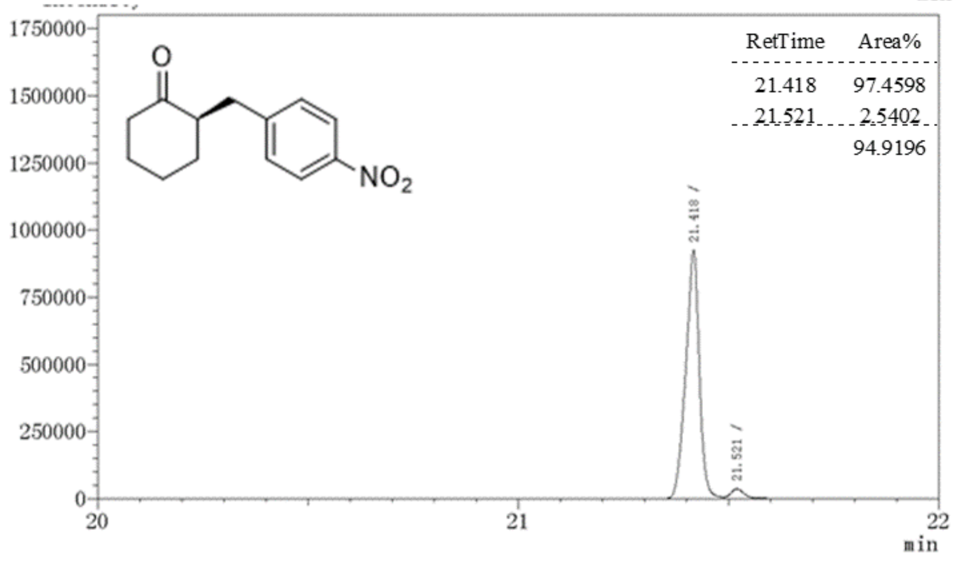
1750000
1500000
1250000
1000000
750000
500000
250000
0
NO2
RetTime Area%
21.418 97.4598
21.521 2.5402
94.9196
21.418
21.521
20
21
22
min

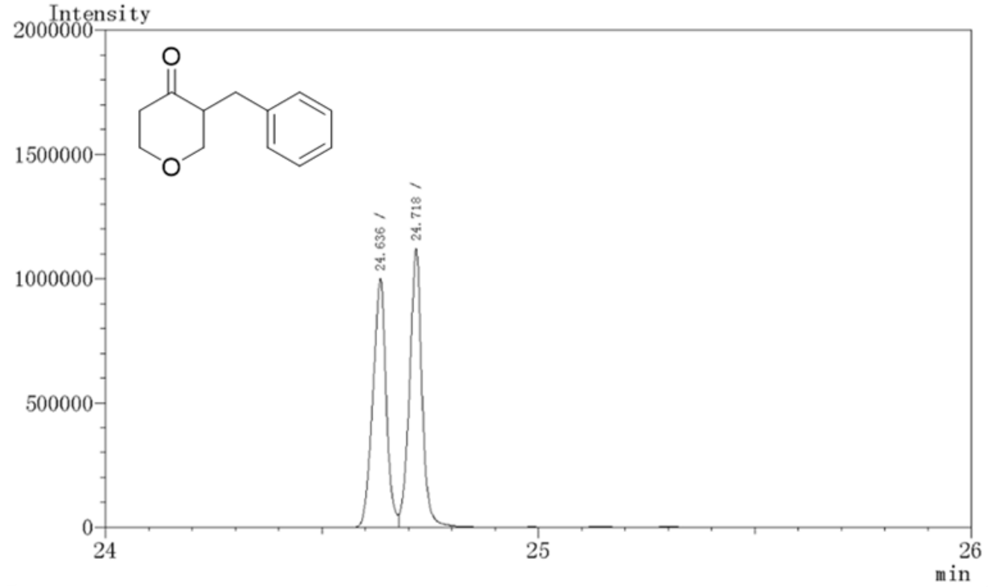
Intensity
2000000
1500000
1000000
500000
0
24
25
26
min
24.636
24.718

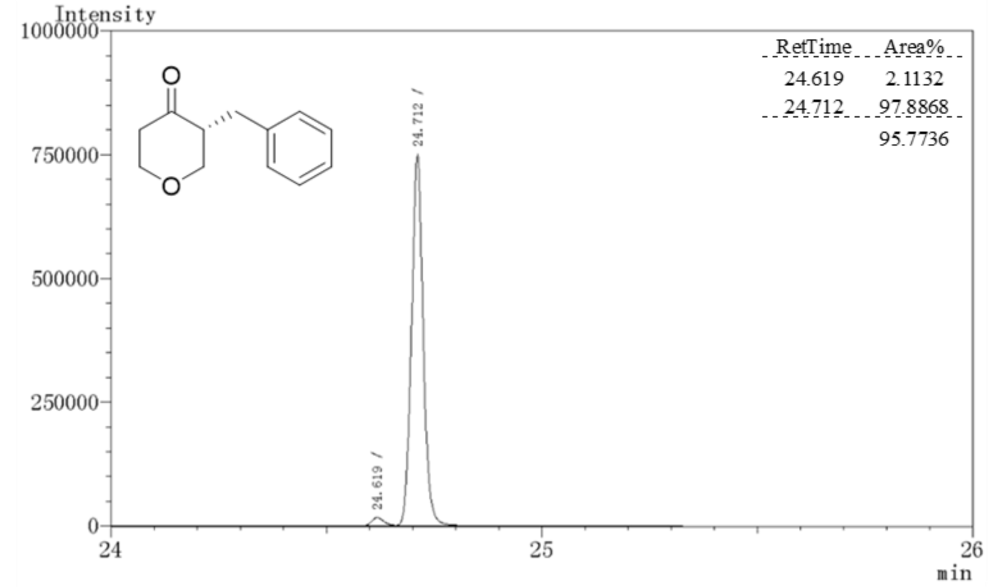
Intensity
1000000
750000
500000
250000
0
24
25
26
min
24.619
24.712
RetTime Area%
24.619 2.1132
24.712 97.8868
95.7736

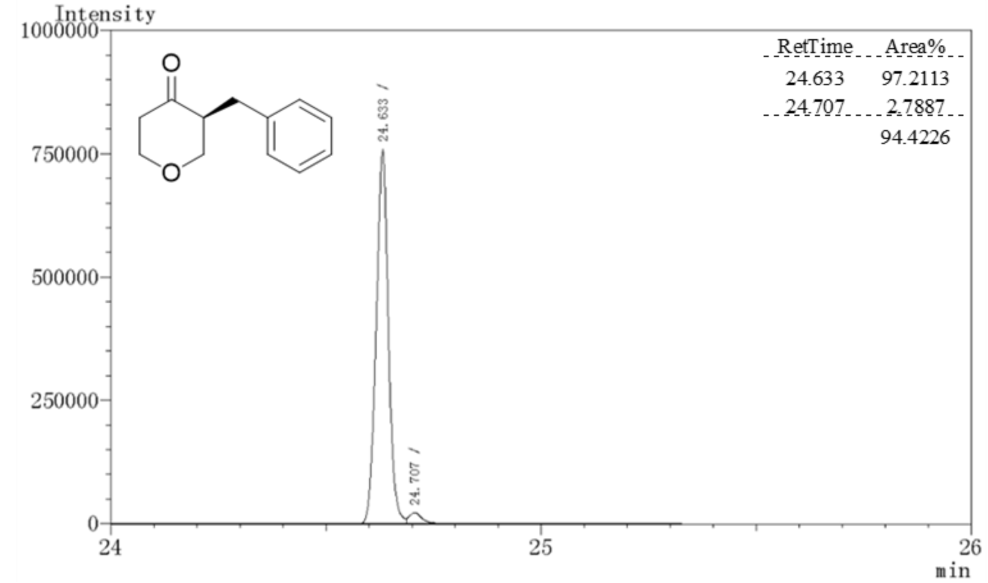
Intensity
1000000
750000
500000
250000
0
24
25
26
min
24.633
24.707
RetTime Area%
24.633 97.2113
24.707 2.7887
94.4226

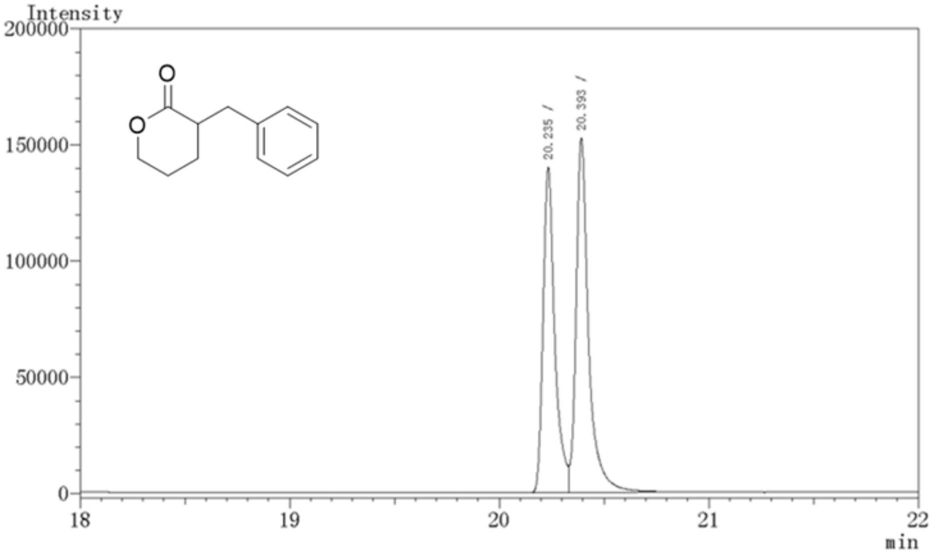
Intensity
200000
150000
100000
50000
0
20.235
20.393
18
19
20
21
22
min

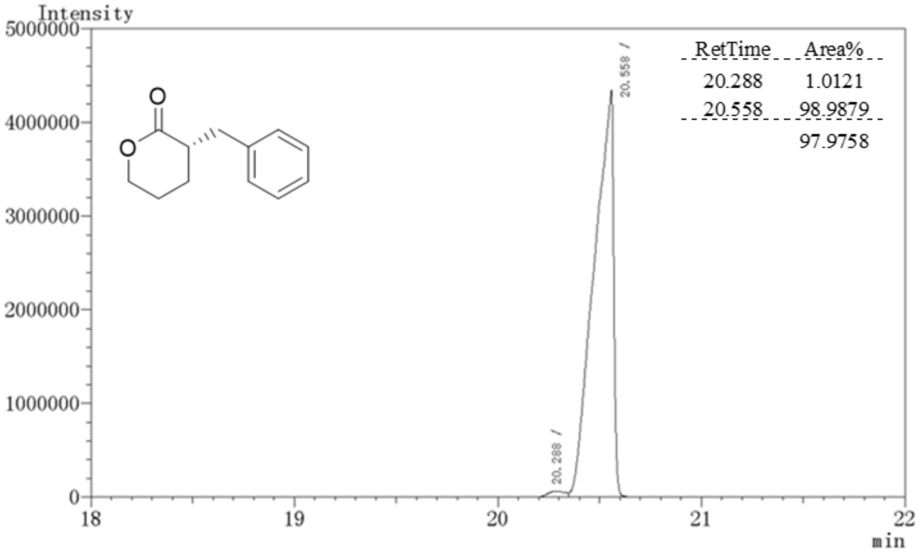
Intensity
5000000
4000000
3000000
2000000
1000000
0
RetTime Area%
20.288 1.0121
20.558 98.9879
97.9758
20.558
20.288
18
19
20
21
22
min

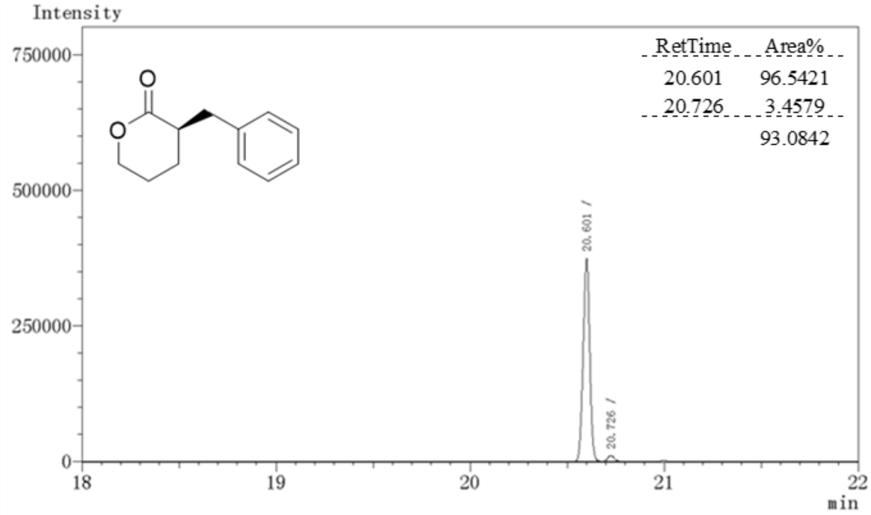
Intensity
750000
500000
250000
0
RetTime Area%
20.601 96.5421
20.726 3.4579
93.0842
20.601
20.726
18
19
20
21
22
min

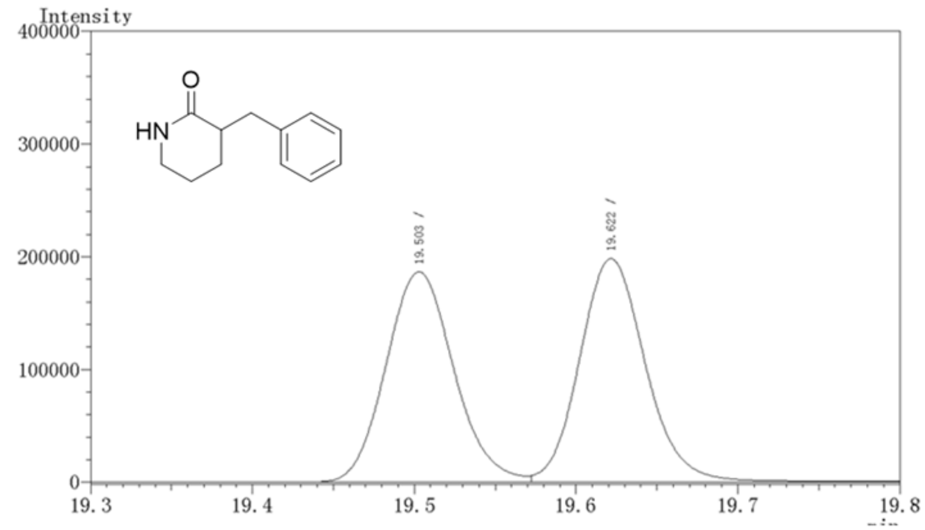
Intensity
400000
300000
200000
100000
0
HN
O
19.503
19.622
19.3
19.4
19.5
19.6
19.7
19.8

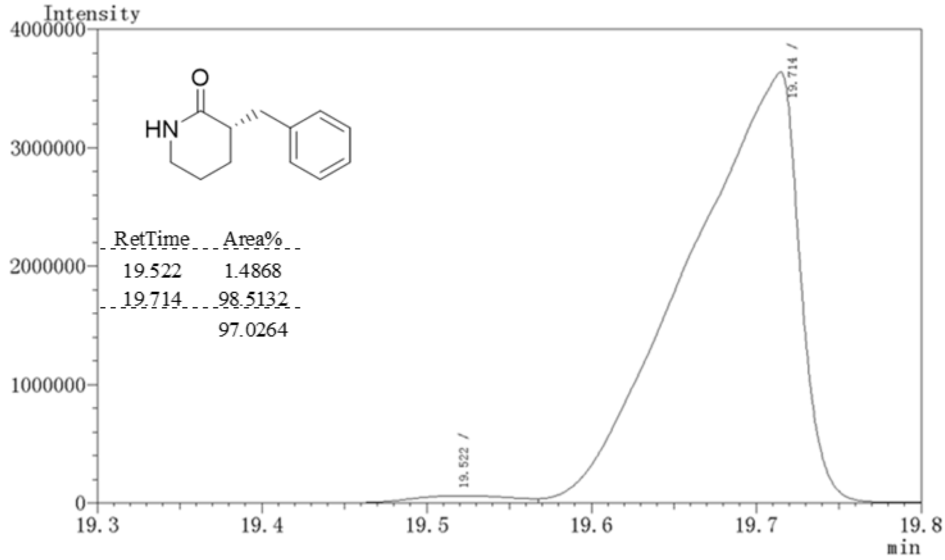
Intensity
4000000
3000000
2000000
1000000
0
HN
O
RetTime Area%
19.522 1.4868
19.714 98.5132
97.0264
19.522
19.714
19.3
19.4
19.5
19.6
19.7
19.8
min

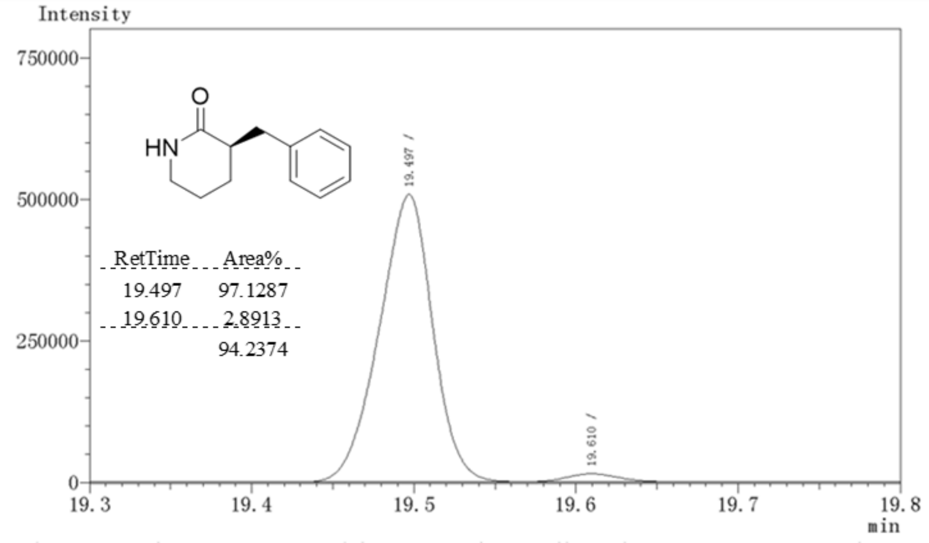
Intensity
750000
500000
250000
0
HN
O
RetTime Area%
19.497 97.1287
19.610 2.8913
94.2374
19.497
19.610
19.3
19.4
19.5
19.6
19.7
19.8
min

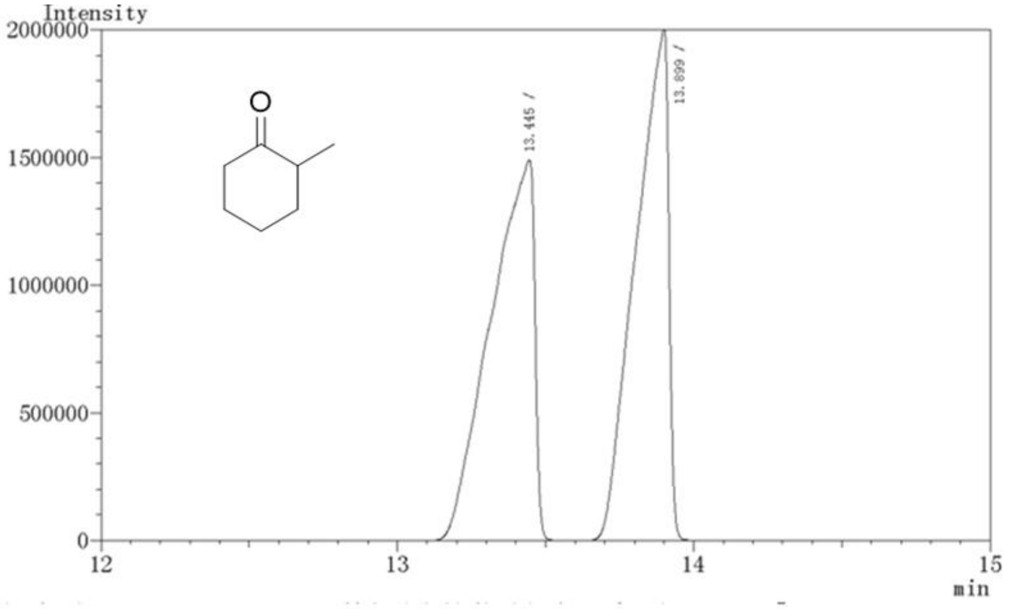
Intensity
2000000
1500000
1000000
500000
0
12
13
14
15
min
13.445
13.899
O

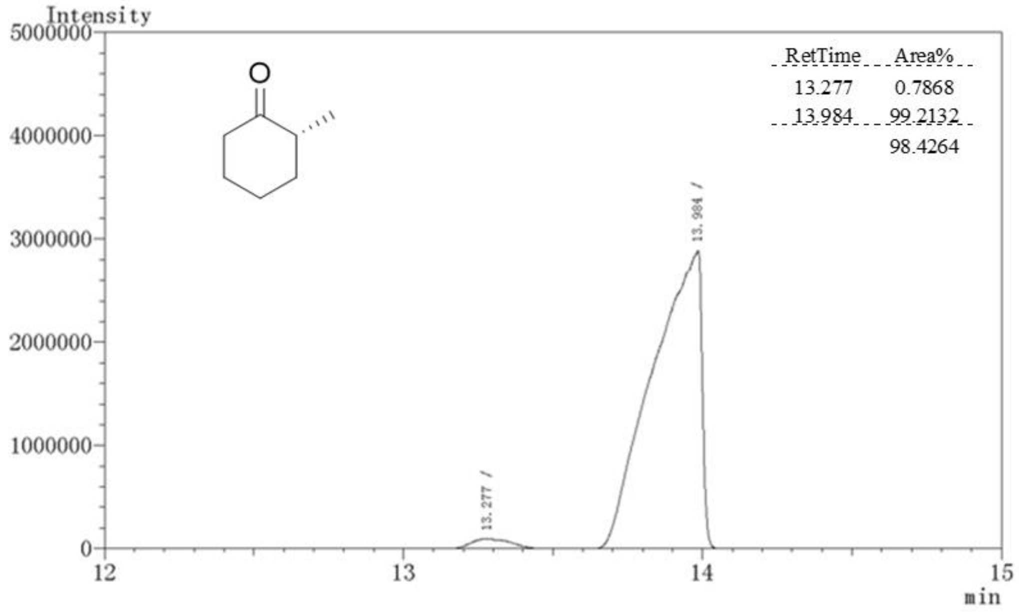
Intensity
5000000
4000000
3000000
2000000
1000000
0
12
13
14
15
min
RetTime Area%
13.277 0.7868
13.984 99.2132
98.4264
13.277
13.984
O

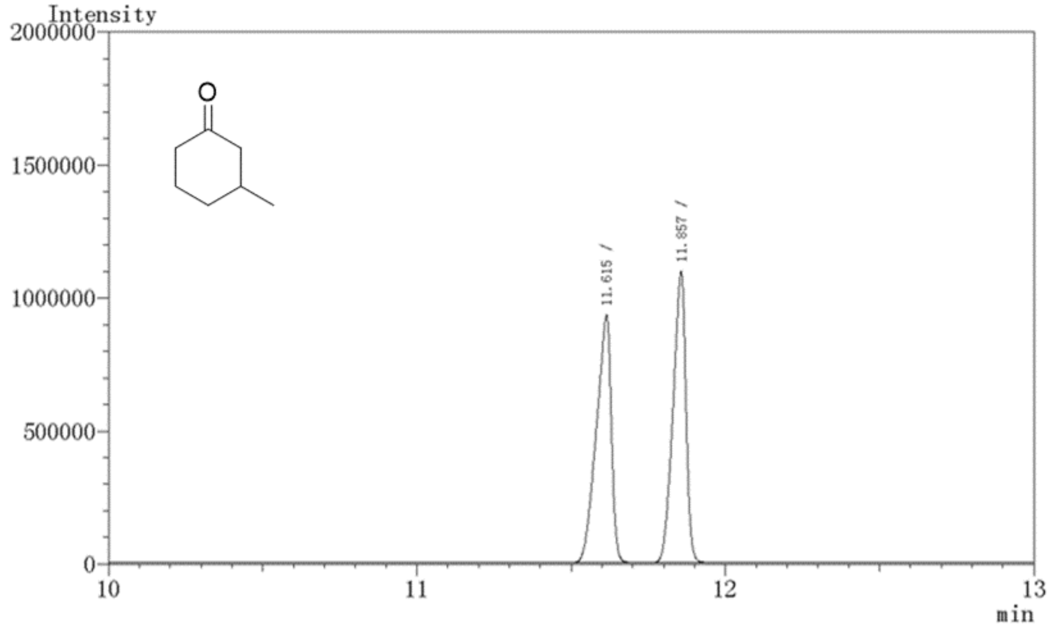
Intensity
2000000
1500000
1000000
500000
0
11.615
11.857
10
11
12
13
min

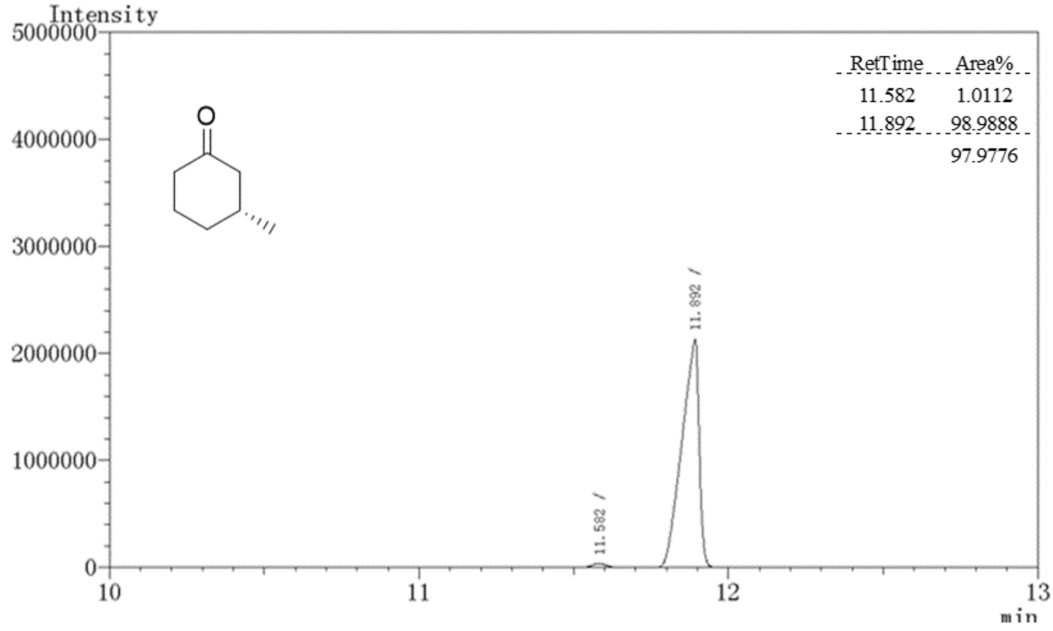
Intensity
5000000
4000000
3000000
2000000
1000000
0
RetTime Area%
11.582 1.0112
11.892 98.9888
97.9776
11.892
11.582
10
11
12
13
min

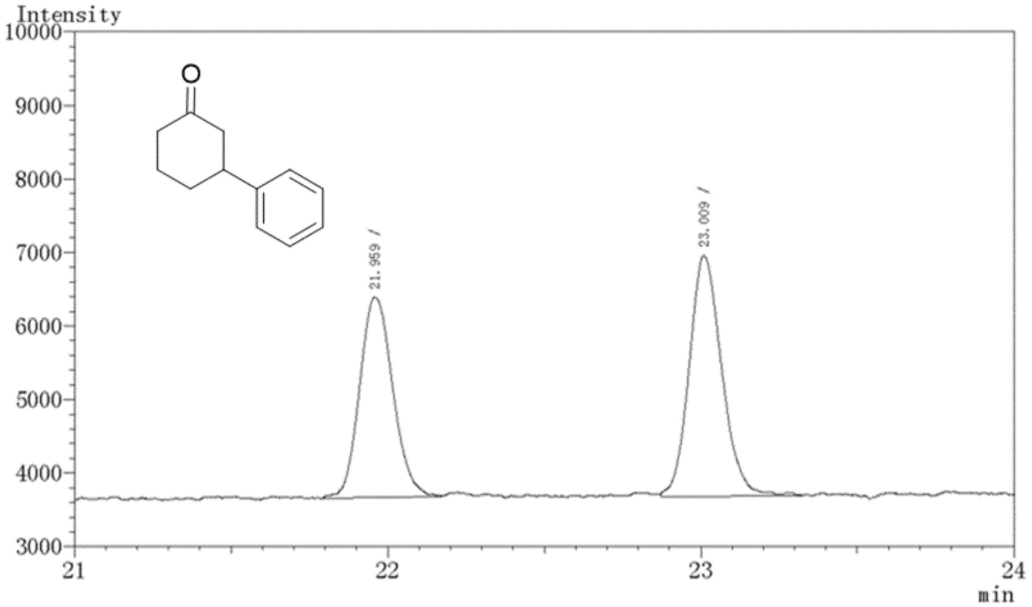
Intensity
21.959
23.009
min

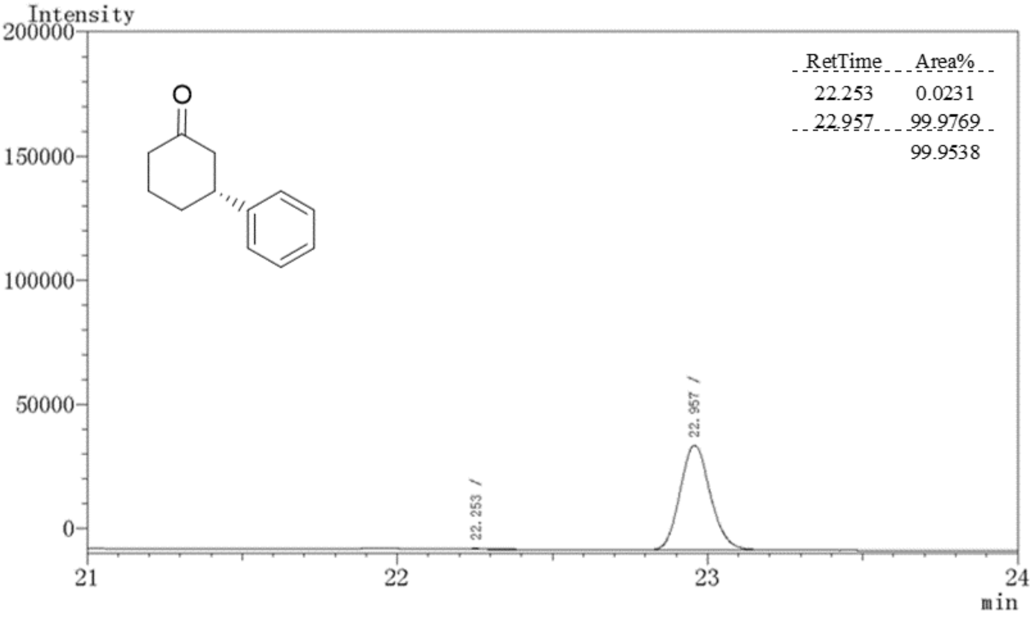
Intensity
RetTime Area%
22.253 0.0231
22.957 99.9769
99.9538
22.253
22.957
min

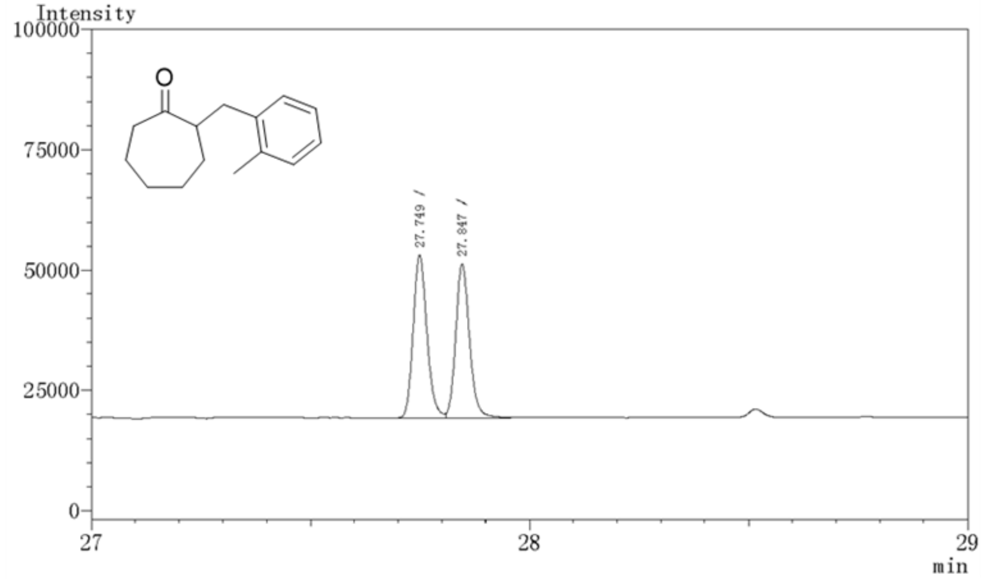
Intensity
100000
75000
50000
25000
0
27.749
27.847
27
28
29
min

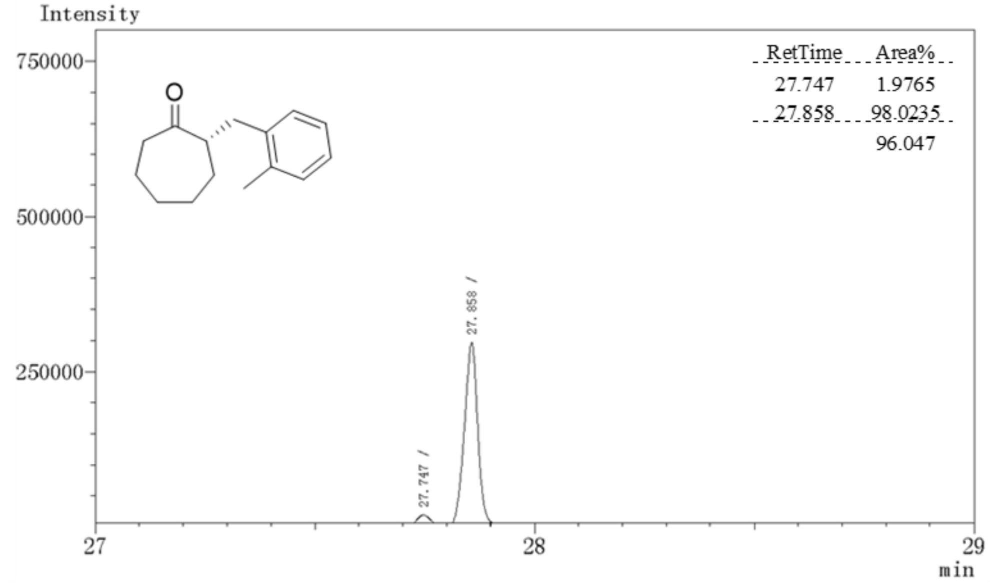
Intensity
750000
500000
250000
RetTime Area%
27.747 1.9765
27.858 98.0235
96.047
27.747
27.858
27
28
29
min

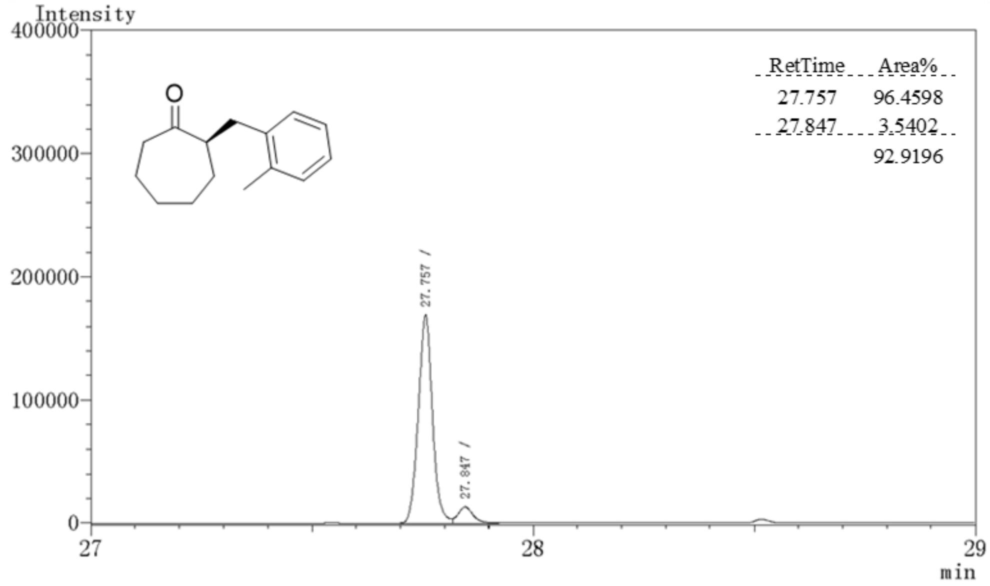
Intensity
400000
300000
200000
100000
0
RetTime Area%
27.757 96.4598
27.847 3.5402
92.9196
27.757
27.847
27
28
29
min

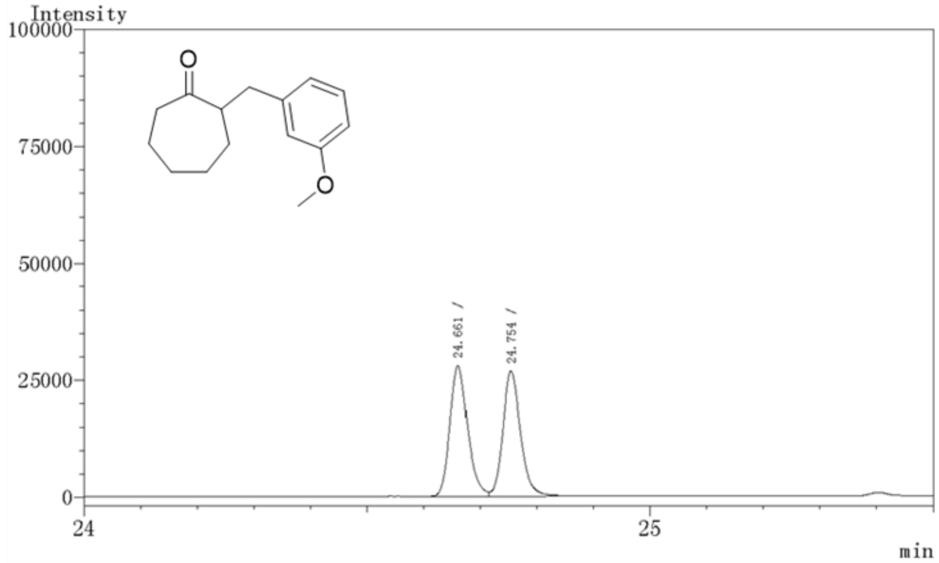
Intensity
100000
75000
50000
25000
0
24.661
24.754
24
25
min

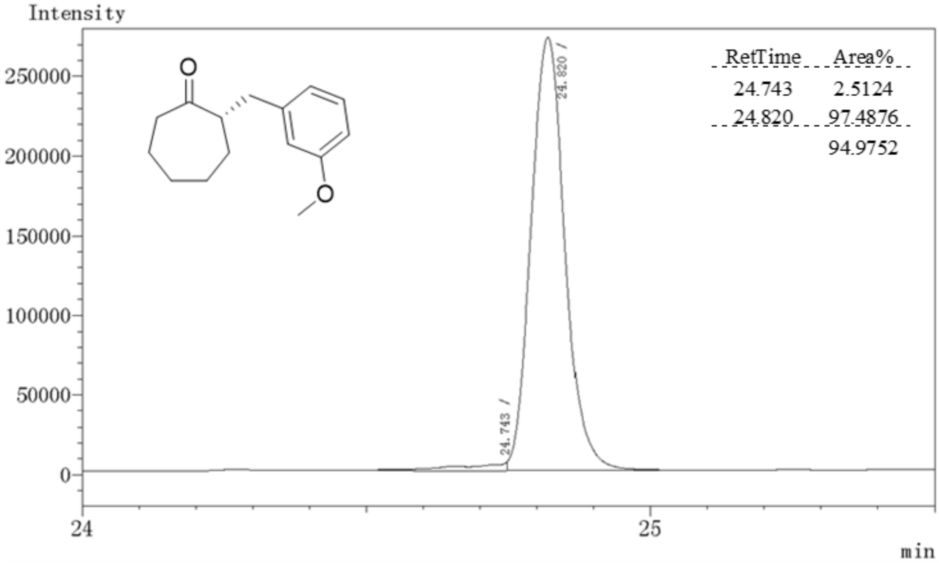
Intensity
250000
200000
150000
100000
50000
0
24.743
24.820
24
25
min
RetTime Area%
24.743 2.5124
24.820 97.4876
94.9752

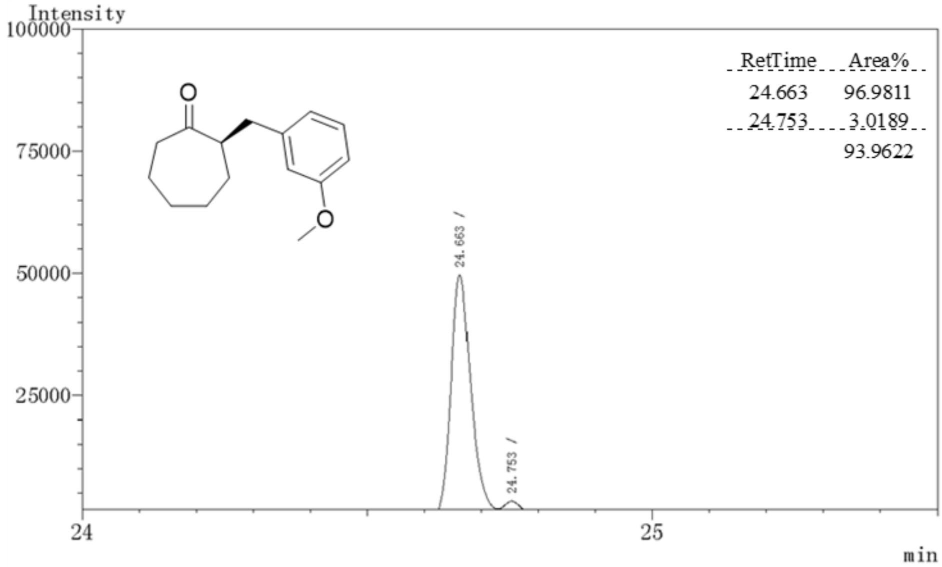
Intensity
100000
75000
50000
25000
24.663
24.753
24
25
min
RetTime Area%
24.663 96.9811
24.753 3.0189
93.9622

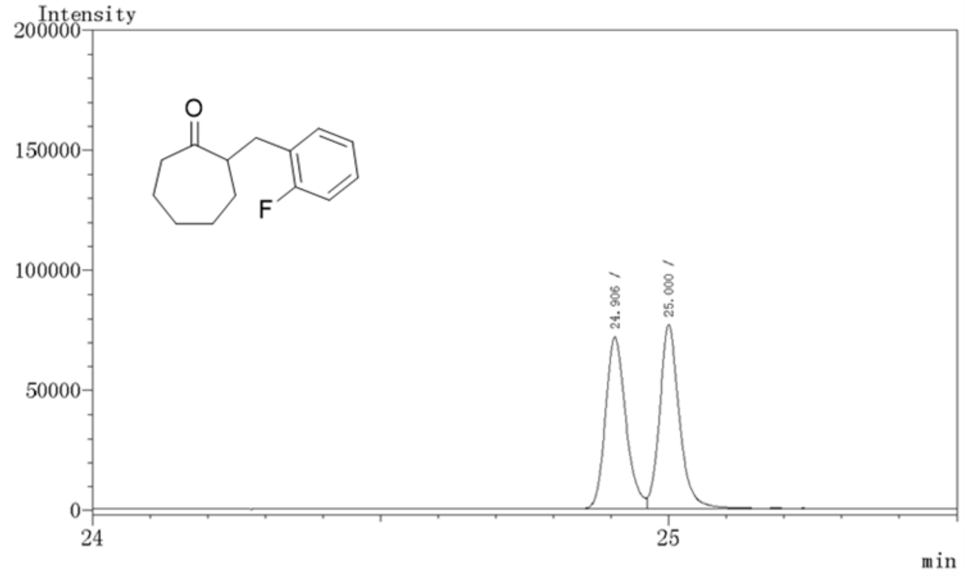
Intensity
200000
150000
100000
50000
0
O
F
24.906
25.000
24
25
min

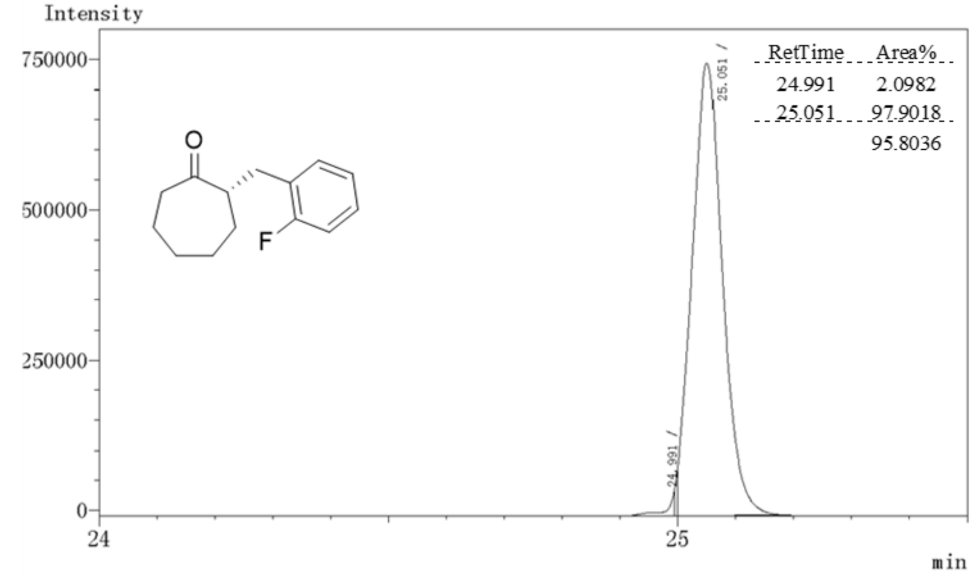
Intensity
750000
500000
250000
0
O
F
RetTime Area%
24.991 2.0982
25.051 97.9018
95.8036
25.051
24.991
24
25
min

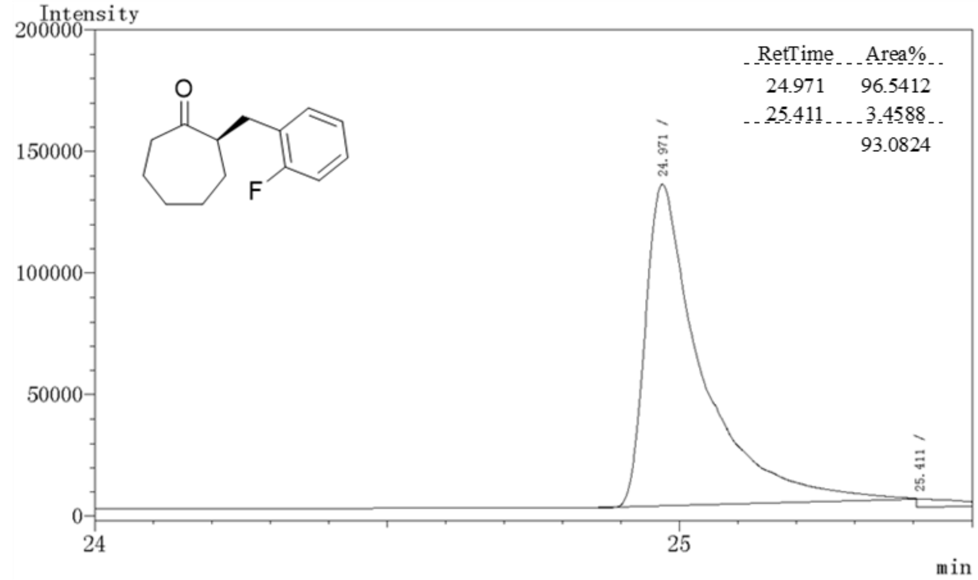
Intensity
200000
150000
100000
50000
0
O
F
RetTime Area%
24.971 96.5412
25.411 3.4588
93.0824
24.971
25.411
24
25
min

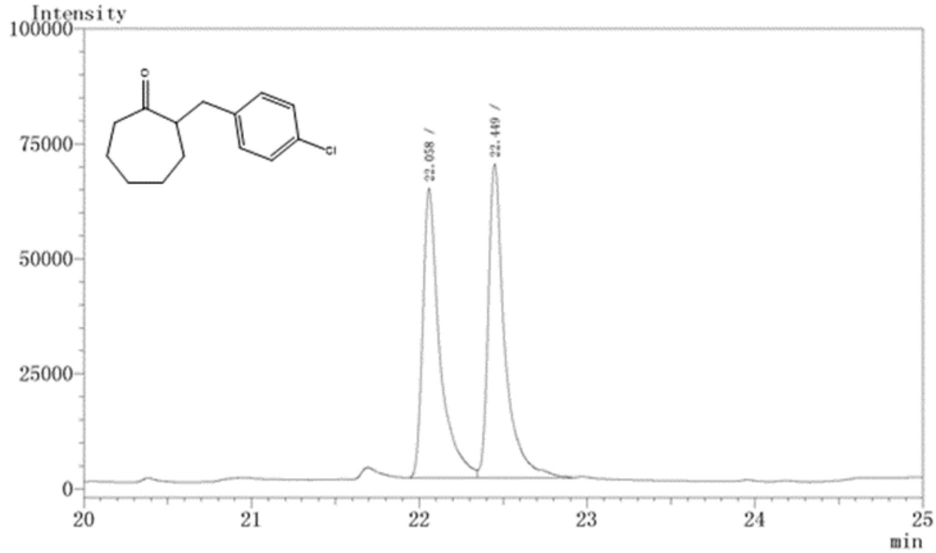
Intensity
100000
75000
50000
25000
0
22.058
22.449
Cl
20
21
22
23
24
25
min

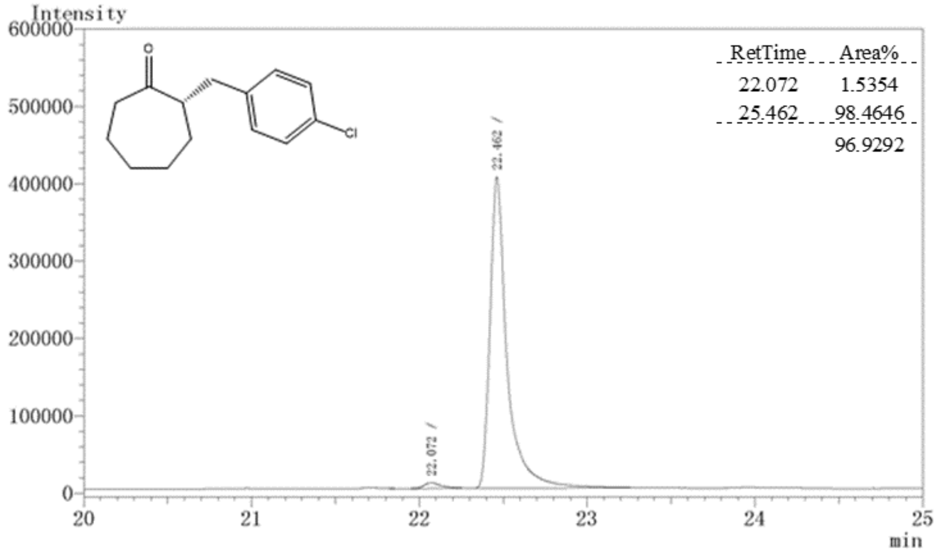
Intensity
600000
500000
400000
300000
200000
100000
0
RetTime Area%
22.072 1.5354
25.462 98.4646
96.9292
22.072
22.462
Cl
20
21
22
23
24
25
min

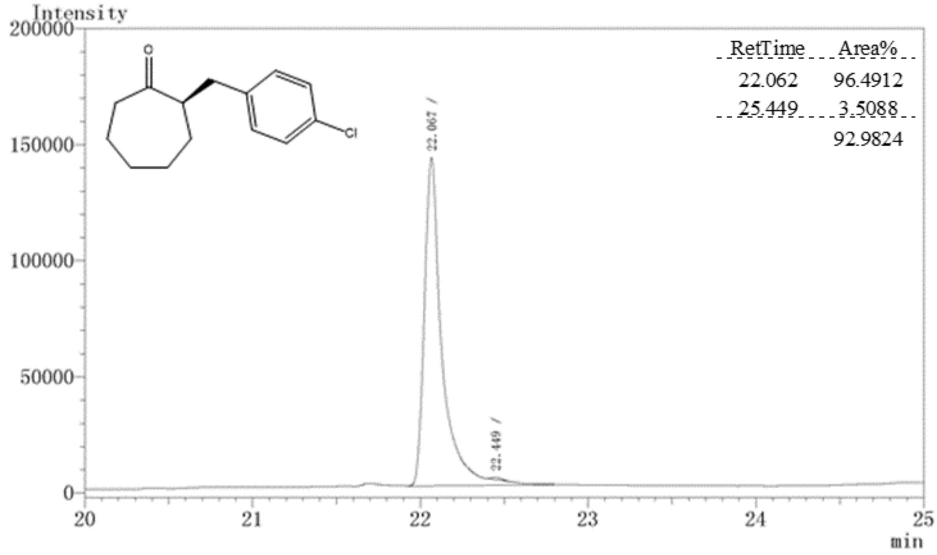
Intensity
200000
150000
100000
50000
0
RetTime Area%
22.062 96.4912
25.449 3.5088
92.9824
22.067
22.449
Cl
20
21
22
23
24
25
min

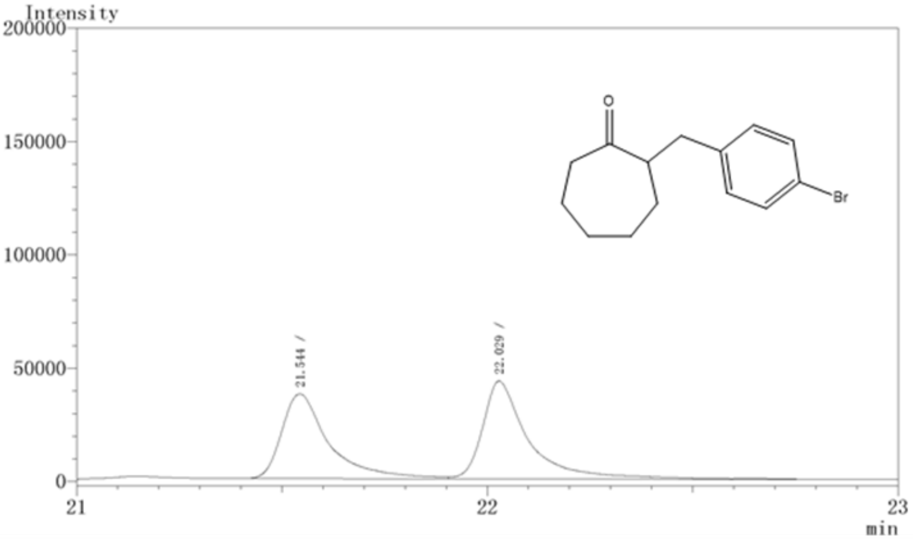
Intensity
200000
150000
100000
50000
0
21.544
22.029
O
Br
21
22
23
min

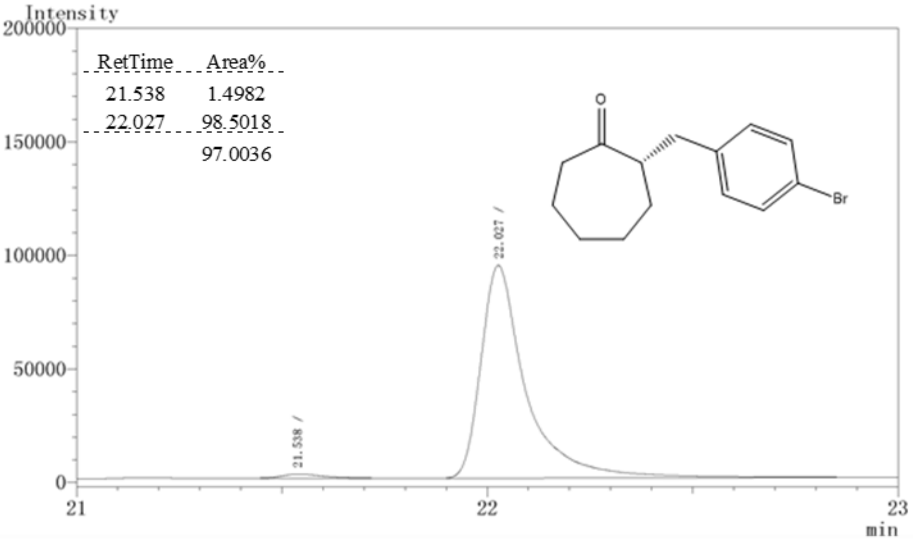
Intensity
200000
RetTime Area%
21.538 1.4982
22.027 98.5018
97.0036
150000
100000
50000
0
22.027
21.538
O
Br
21
22
23
min

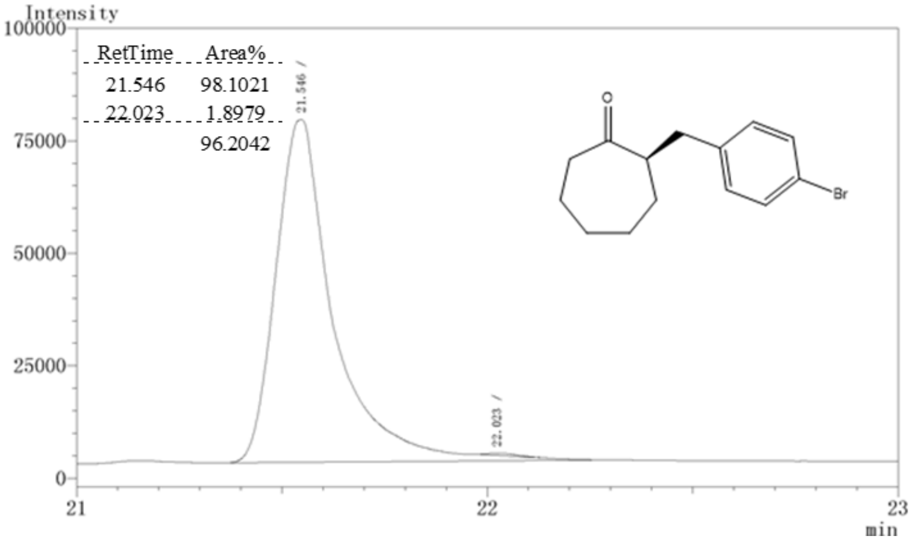
Intensity
100000
RetTime Area%
21.546 98.1021
22.023 1.8979
96.2042
75000
50000
25000
0
21.546
22.023
O
Br
21
22
23
min

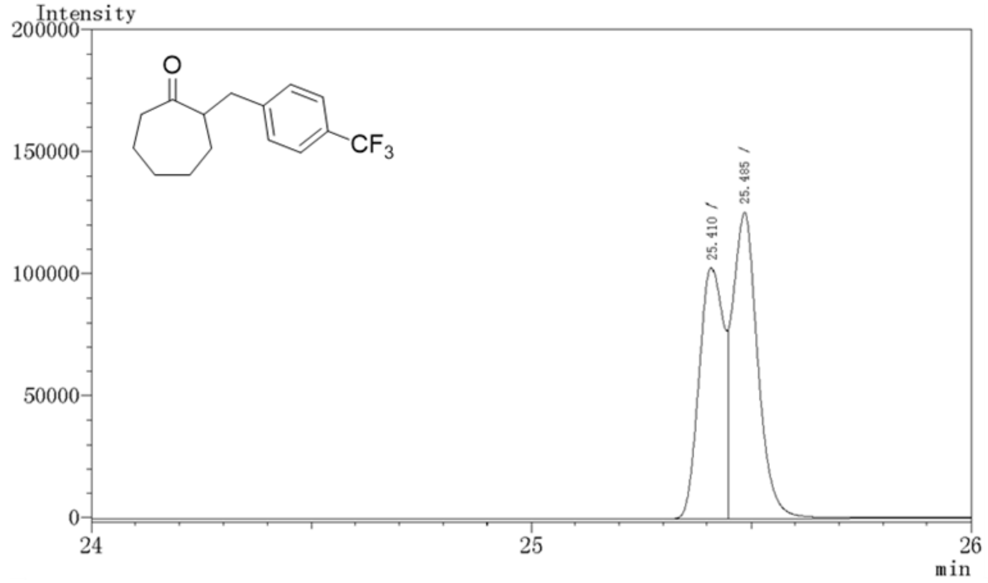
Intensity
200000
150000
100000
50000
0
24
25
26
min
25.410
25.485
O
CF3

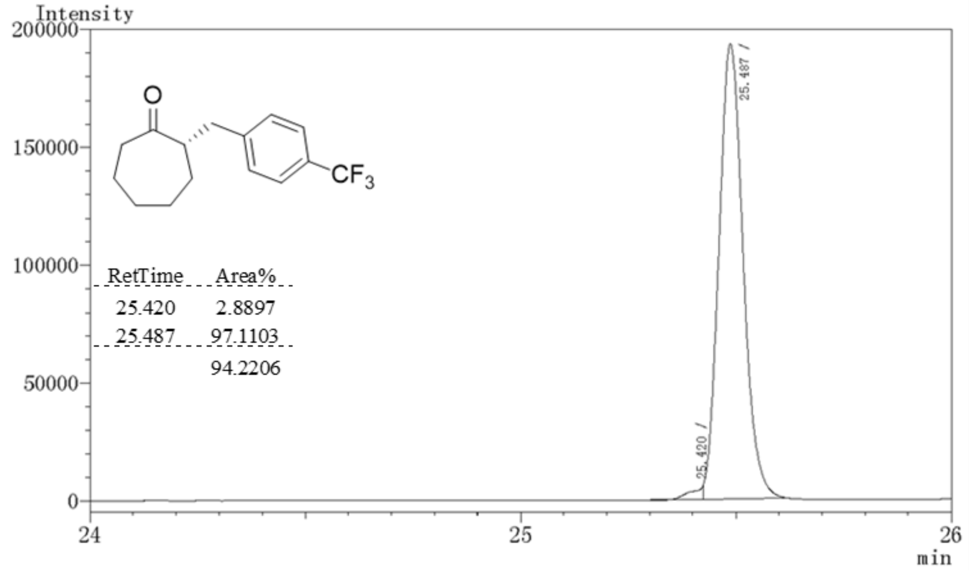
Intensity
200000
150000
100000
50000
0
24
25
26
min
25.487
25.420
O
CF3
RetTime Area%
25.420 2.8897
25.487 97.1103
94.2206

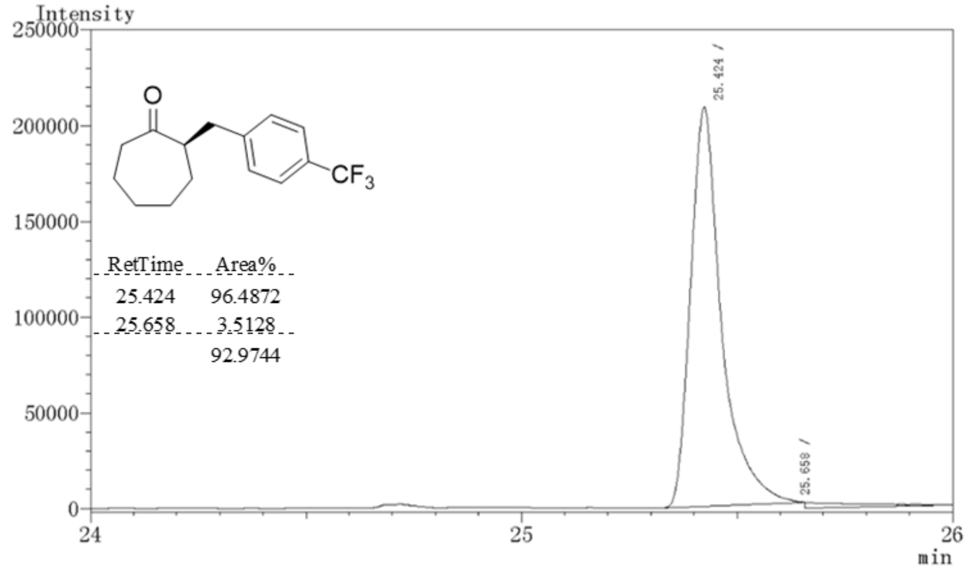
Intensity
250000
200000
150000
100000
50000
0
24
25
26
min
25.424
25.658
O
CF3
RetTime Area%
25.424 96.4872
25.658 3.5128
92.9744

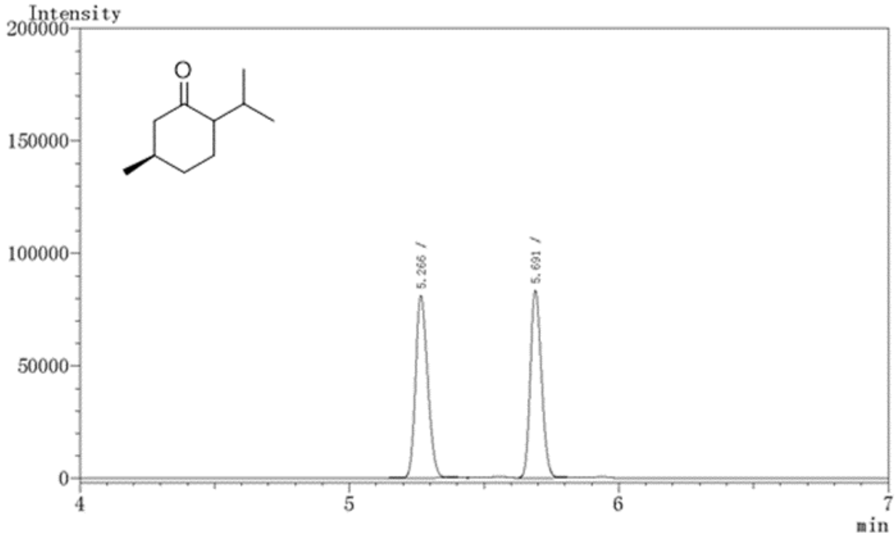
Intensity
200000
150000
100000
50000
0
4
5
6
7
min
5.266
5.691

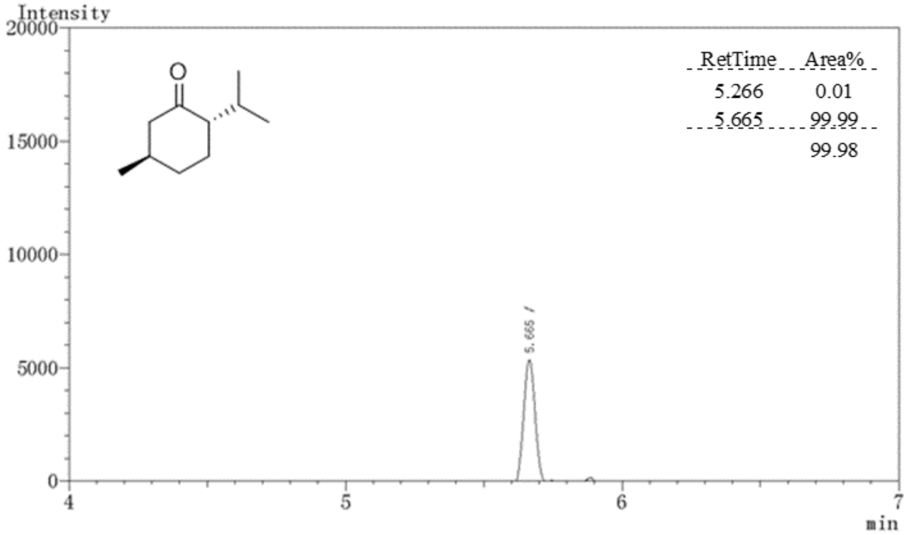
Intensity
20000
15000
10000
5000
0
4
5
6
7
min
5.665
RetTime Area%
5.266 0.01
5.665 99.99
99.98

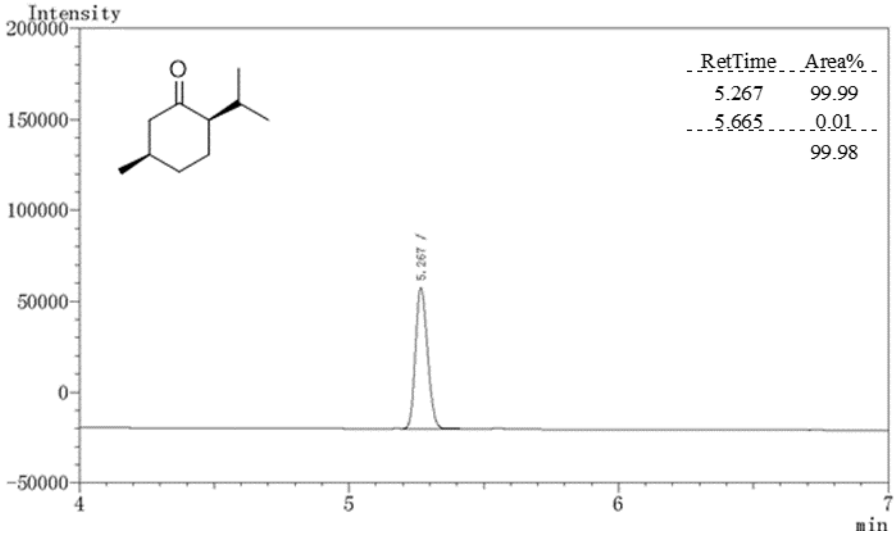
Intensity
200000
150000
100000
50000
0
-50000
4
5
6
7
min
5.267
RetTime Area%
5.267 99.99
5.665 0.01
99.98

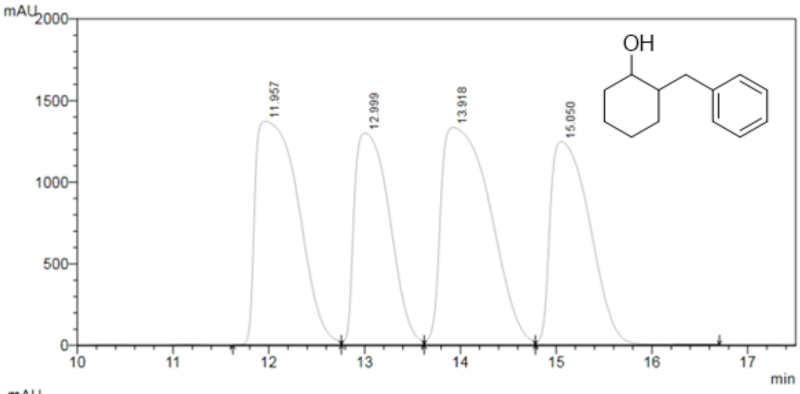
mAU
2000
1500
1000
500
0
10 11 12 13 14 15 16 17
min
11.957
12.999
13.918
15.050
OH

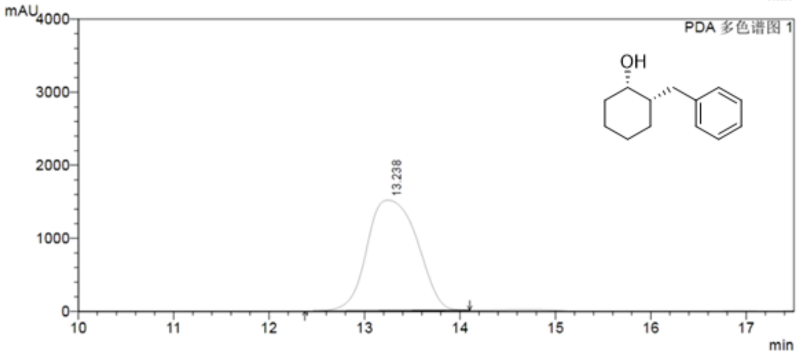
mAU
4000
3000
2000
1000
0
10 11 12 13 14 15 16 17
min
PDA 多色谱图 1
13.238
OH

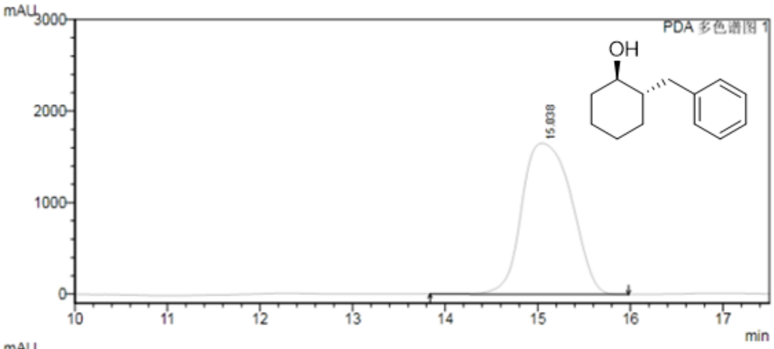
mAU
3000
2000
1000
0
10 11 12 13 14 15 16 17
min
PDA 多色谱图 1
15.038
OH

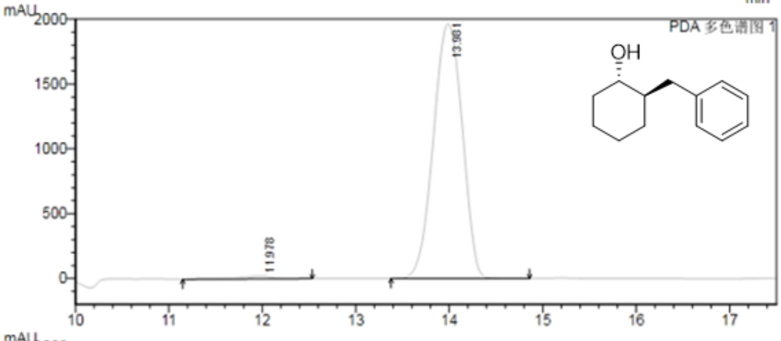
mAU
2000
1500
1000
500
0
10 11 12 13 14 15 16 17
min
PDA 多色谱图 1
11.978
13.981
OH

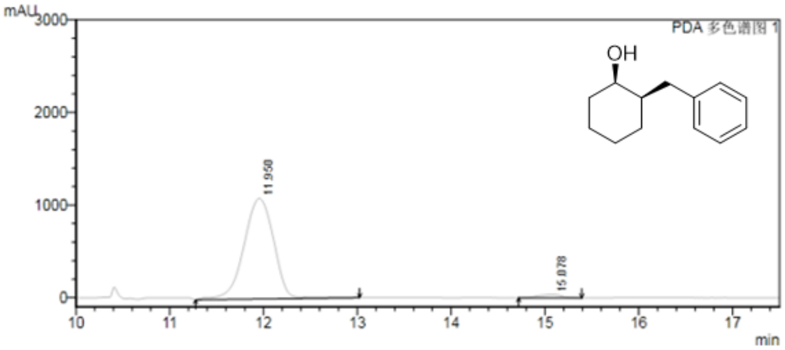
mAU
3000
2000
1000
0
10 11 12 13 14 15 16 17
min
PDA 多色谱图 1
11.950
15.078
OH

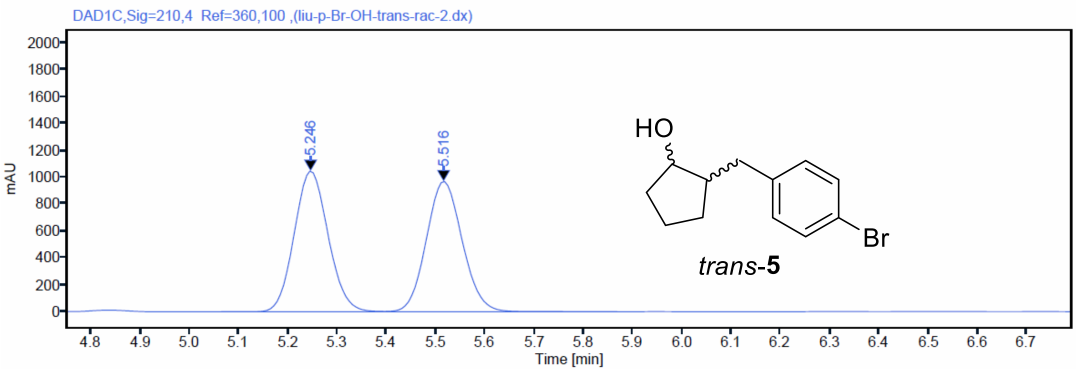

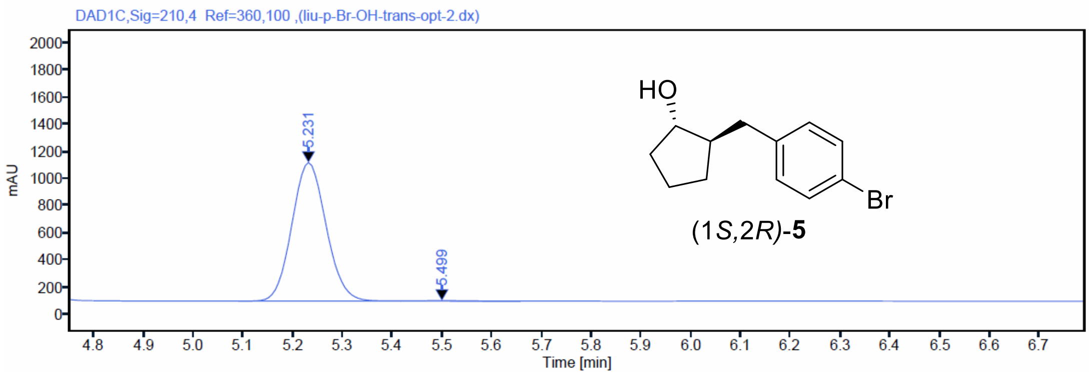

Signal: DAD1C,Sig=210,4 Ref=360,100

RT [min]	Type	Width [min]	Area	Height	Area%
5.231	BV	0.33	4877.76	1021.34	99.26
5.499	VV	0.24	36.22	4.27	0.74
		Sum	4913.97		

Supplementary References

1. Frisch, M. J. et al. Gaussian 09, revision A.1; Gaussian, Inc.: Wallingford, CT, 2009.

2. Lonsdale, R. & Reetz, M. T. Reduction of α, β-unsaturated ketones by old yellow enzymes: mechanistic insights from quantum mechanics/molecular mechanics calculations. *J. Am. Chem. Soc.* **137**, 14733-14742 (2015).

3. Marenich, A. V., Cramer, C. J. & Truhlar, D. G. Universal solvation model based on solute electron density and on a continuum model of the solvent defined by the bulk dielectric constant and atomic surface tensions. *J. Phys. Chem. B* **113**, 6378-6396 (2009).

4. Becke, A. D. Density-functional exchange-energy approximation with correct asymptotic behavior, *Phys. Rev.* **38**, 3098-3100 (1988).

5. Qu, G. et al. Computational insights into the catalytic mechanism of bacterial carboxylic acid reductase. *J. Chem. Inf. Model.* **59**, 832-841 (2019).

6. Li, X. et al. Chemoselective conjugate reduction of α,β-unsaturated ketones catalyzed by rhodium amido complexes in aqueous media. *J. Org. Chem.* **75**, 2981-2988 (2010).

7. Lu, S. M. & Bolm, C. Highly enantioselective synthesis of optically active ketones by iridium-catalyzed asymmetric hydrogenation. *Angew. Chem. Int. Ed.* **47**, 8920-8923 (2008).

8. Tian, F., Yao, D., Liu, Y., Xie, F. & Zhang, W., Iridium-catalyzed highly enantioselective hydrogenation of exocyclic α,β-unsaturated carbonyl compounds, *Adv. Synth. Catal.* **352**, 1841–1845 (2010).

9. Liu, X., Han, Z., Wang, Z. & Ding, K. Spinphox/iridium(I)-catalyzed asymmetric hydrogenation of cyclic α-alkylidene carbonyl compounds. *Angew. Chem. Int. Ed.* **53**, 1978-1982 (2014).

10. Yanga, Y., Lia, M., Cao, H., Zhang, X. & Yu, L., Unexpected Pd/C-catalyzed room temperature and atmospheric pressure hydrogenation of 2-methylenecyclobutanones, *Mol. Catal.* **474,** 110450 (2019).

11. Casavant, B. J., Khoder, Z. M., Berhane, I. A. & Chemler, S. R., Copper(II)-promoted cyclization/difunctionalization of allenols and allenylsulfonamides: synthesis of heterocycle-functionalized vinyl carboxylate esters, *Org. Lett.* **17**, 24, 5958-5961 (2015).

12. Benjamin, S. L., Karagiannidis, L., Levason, W., Reid, G. & Rogers, M. C. Hybrid dibismuthines and distibines: preparation and properties of antimony and bismuth oxygen, sulfur, and nitrogen donor ligands. *Organometallics* **30**, 895-904 (2011).
13. Zhang, X. W. et al. Synthesis, structure, and in vitro antiproliferative activity of cyclic hypervalent organobismuth(III) chlorides and their triphenylgermylpropionate derivatives. *J. Organomet. Chem.* **694**, 3019-3026 (2009).
14. Qiu, R. et al. Facile separation catalyst system: direct diastereoselective synthesis of (*E*)-α,β-unsaturated ketones catalyzed by an air-stable Lewis acidic/basic bifunctional organobismuth complex in ionic liquids. *Green Chem.* **12**, 1767-1771 (2010).
15. Qiu, R. et al. Highly efficient and selective synthesis of (*E*)-α,β-unsaturated ketones by crossed condensation of ketones and aldehydes catalyzed by an air-stable cationic organobismuth perfluorooctanesulfonate. *Adv. Synth. Catal.* **352**, 153-162 (2010).
16. Luan, P. et al. Design of *de novo* three-enzyme nanoreactors for stereodivergent synthesis of α-substituted cyclohexanols. *ACS Catal.* **12**, 7550-7558 (2022).
17. Jeanne Masson-Makdissi, J., Ching, J., Reid, C. M. & Lautens, M. Pd/Rh dual catalysis: tandem isomerization-allylation to access α-quaternary carbonyl compounds. *ACS Catal.* **12**, 15130-15134 (2022).
18. Xie, J. B. et al. Highly enantioselective hydrogenation of α-arylmethylene cycloalkanones catalyzed by iridium complexes of chiral spiro aminophosphine ligands. *J. Am. Chem. Soc.* **132**, 4538-4539 (2010).
